# Optimal pandemic control strategies and cost-effectiveness of COVID-19 non-pharmaceutical interventions in the United States

**DOI:** 10.1186/s44263-025-00189-z

**Published:** 2025-09-12

**Authors:** Nicholas J. Irons, Adrian E. Raftery

**Affiliations:** 1 Department of Statistics, Leverhulme Centre for Demographic Science, and Pandemic Sciences Institute, University of Oxford, Oxford, UK; 2 Departments of Statistics and Sociology, University of Washington, Seattle, USA

**Keywords:** COVID-19, Interventions, Social distancing, Cost-effective, Optimal control, Infectious disease

## Abstract

**Background:**

Non-pharmaceutical interventions (NPIs) in response to the COVID-19 pandemic necessitated a trade-off between the health impacts of viral spread and the social and economic costs of restrictions. Navigating this trade-off proved consequential, contentious, and challenging for decision-makers.

**Methods:**

We conduct a cost-effectiveness analysis of NPIs enacted at the state level in the United States (US) in 2020. We combine data on COVID-19 cases, deaths, policies, and the social, economic, and health consequences of infections and interventions within an epidemiological model. We estimate SARS-CoV-2 prevalence, transmission rates, effects of interventions, and costs associated to infections and NPIs in each US state. We use these estimates to quantitatively evaluate the efficacy and gross impacts of the policy schedules implemented during the pandemic. We also derive optimal cost-effective strategies that minimize aggregate costs to society.

**Results:**

We find that NPIs were effective in substantially reducing SARS-CoV-2 transmission, averting 860,000 (95% CI: 560,000–1,190,000) COVID-19 deaths in the US in 2020. Although school closures reduced transmission, their social impact in terms of student learning loss was too costly, depriving the nation of $2 trillion in 2020 US dollars (USD2020), conservatively, in future Gross Domestic Product (GDP). Moreover, this marginal trade-off between school closure and COVID-19 deaths was not inescapable: a combination of other measures would have been enough to maintain similar or lower mortality rates without incurring such profound learning loss. Optimal policies involve consistent implementation of mask mandates, public test availability, contact tracing, social distancing orders, and reactive workplace closures, with no closure of schools. Their use would have reduced the gross impact of the pandemic in the US in 2020 from $4.6 trillion to $1.9 trillion and, with high probability, saved over 100,000 lives.

**Conclusions:**

US COVID-19 school closure was not cost-effective, but other measures were. While our study focuses on COVID-19 in the US prior to vaccines, our methodological contributions and findings about the cost-effectiveness and optimal structure of NPI policies have implications for the response to future epidemics and in other countries. Our results also highlight the need to address the substantial global learning deficit incurred during the pandemic.

**Supplementary information:**

The online version contains supplementary material available at 10.1186/s44263-025-00189-z.

## Background

In the year prior to the arrival of COVID-19 vaccines and other pharmaceutical interventions, NPIs—including school and workplace closures, social distancing, masking, testing, and contact tracing—were the primary tools for mitigating the spread of SARS-CoV-2. The use of NPIs posed significant challenges to decision-makers at every level of government, who were forced to make difficult and consequential real-time decisions with limited data and amidst contentious political debate [1]. While they substantially reduced viral transmission, extended lockdowns had severe deleterious social and economic consequences globally—including disrupted economic output, job loss, and student learning loss [2, 3]—on top of the already staggering health impacts of the pandemic. These impacts—health, economic, and social—were felt disproportionately by marginalized populations [2, 4, 5].

The literature studying NPIs in response to COVID-19 and past pandemics is vast. Brodeur et al. [6] and Bloom et al. [7] provide reviews of the economics literature. Other relevant studies include: estimating associations and inferring causal effects of NPIs on viral transmission [8–19]; quantifying the gross health and economic impacts of pandemics and the associated policy response [20–26]; and modeling the (optimal) control of epidemics and the cost-effectiveness of NPIs appearing in the economics [27–37] and public health literature [38–50]. Nevertheless, high quality evidence concerning the cost-effectiveness of NPIs during the COVID-19 pandemic is sparse. Systematic reviews in the context of COVID-19 identified school closure as the most highly effective NPI in reducing transmission, with other effective measures including workplace closure, other social distancing measures, and masking [51]. Regarding cost-effectiveness, testing, contact tracing, quarantine, and social distancing were identified as highly cost-effective, but the available reviews noted a lack of quality research and conclusive evidence [52, 53].

To address the NPI planning problem in a principled way, we develop a statistical decision framework and conduct a cost-effectiveness analysis of NPI policies enacted at the state level in the US in 2020. Our analysis is composed of three steps. We first build a Bayesian epidemiological model tracking state-level SARS-CoV-2 transmission. We next estimate the effects of NPIs on viral transmission in all states jointly using a Bayesian hierarchical regression model. Finally, we couple these estimates with monetary costs associated with the social, economic, and health consequences of infection and NPIs drawn from the literature in order to quantitatively evaluate the efficacy and gross impacts of the policy schedules implemented during the pandemic, and to derive strategies that optimally navigate the trade-off between restrictions and viral spread.

We build upon the existing literature to address gaps limiting its value in informing policy. Firstly, we study the optimal control of a pandemic using statistical decision theory [54]. We take a data-driven approach to the NPI decision process, estimating and accounting for uncertainty in key parameters, including viral prevalence, reproduction numbers, and the effects of NPIs and other endogenous and exogenous factors on transmission rates. In particular, we produce probabilistic estimates of SARS-CoV-2 prevalence over time, which are necessary to properly account for the magnitude and uncertainty of costs associated with infections, based on prior work leveraging random sample testing surveys to debias clinical COVID-19 data [55]. Our model is able to capture the complex and stochastic temporal trends of SARS-CoV-2 transmission (e.g., multiple waves, super-spreader events, the introduction of new infections via travel, and random fluctuations) which can be missed by standard deterministic epidemiological models. This allows us to define and evaluate realistic counterfactual scenarios under different NPI policies conditional on what was observed during the pandemic.

Secondly, we model the costs and effects on viral transmission of multiple specific NPIs. Some previous studies have considered a limited toolkit, focusing on a minimal collection of interventions, such as a single catch-all “social distancing”, “containment", or “lockdown” policy [27–32, 34–37, 39, 42, 50]. This can fail to identify the most effective policies, as we generally have a range of tools at our disposal, and NPIs are known to be more effective in combination [38, 43–46, 56] Furthermore, if we consider only a single instrument, we may erroneously conclude that its implementation is cost-effective because we implicitly assume that other policies are not available. As we discuss in Additional file 1: Section S.1.3, the cost-effectiveness of any single intervention is context-specific and depends on the other policy options. We consider a comprehensive set of 11 NPIs. As such, our findings refine the broad qualitative guidance drawn from prior studies in the context of COVID-19—e.g., that “lockdown” is cost-effective and optimal when implemented early and stringently [34]. By evaluating a range of NPIs, we can disaggregate policies to conclude that testing, tracing, masking, reactive workplace closure, and social distancing measures (not including extended school closure) combine to form an optimal cost-effective strategy.

Thirdly, unlike many studies assessing the economic impact of school closure during pandemics, we factor in costs associated with student learning loss [39, 40, 42–49, 57–62]. While students across the country experienced extended disruptions to in-person learning, there was substantial heterogeneity across states in the duration of school closures. Schools in some states remained remote or hybrid for almost the entirety of the 2020–2021 school year, while others returned to fully in-person instruction for most of the year [63]. Many studies quantify the total cost of school closure as a sum of direct costs arising from lost productivity of school staff and workplace absenteeism of parents or childcare costs resulting from students staying home. However, the indirect costs of school closure are substantial. As noted by Haw et al. [64], conventional measures of economic value used for planning can “substantially underestimate the contribution of the education sector to national prosperity."Students suffering acute learning loss go on to become less skilled and less productive members of the workforce, which in turn leads to future losses in personal income and national GDP [65]. We account for the net present value of these future losses to society, which can be very large, based on estimates of the cost of learning loss from the education economics literature [65–67] and recent estimates of the amount of learning loss accrued during COVID-19 school closures [5, 63].

Additional methodological contributions of our approach include a novel zero-inflated negative binomial model that flexibly captures well-known reporting idiosyncrasies (e.g., consistent under-reporting of cases and deaths on holidays and weekends) and over-dispersion (e.g., due to superspreading) in clinical COVID-19 data [68–74]. As a result, our method eliminates the need for ad hoc data cleaning and smoothing procedures that can complicate the analysis pipeline, yield poorly calibrated prediction intervals, and potentially bias transmission rate estimates based on over-smoothed data. Furthermore, we implement a two-stage modeling procedure that first estimates the time-varying effective reproduction number in each US state individually, followed by a joint hierarchical model across states that estimates pooled effects of NPIs on transmission dynamics. This approach allows for efficient Bayesian computation by parallelizing model fits across states. While our results are specific to the COVID-19 pandemic, our methods can be used more widely to evaluate public health interventions against infectious disease and to understand the relationship between interventions, the behavioral response, and disease transmission.

## Methods

### Data and implementation

#### Code availability

Data cleaning was conducted in R and Python. All models were fit in R using the *CmdStanR* package, with Markov chain Monte Carlo (MCMC) convergence assessed using the diagnostics provided therein [75, 76]. Code to download and process the data and reproduce our analysis is available at the GitHub repository [77] and archived in Zenodo [78].

#### Data availability

All data used are publicly available. We obtained US state-level daily counts of confirmed COVID-19 cases and deaths in 2020 from the Johns Hopkins COVID-19 Data Repository [79]. We obtained daily state-level NPI policies from the Oxford COVID-19 Government Response Tracker (OxCGRT) [80]. We obtained daily state-level counts of SARS-CoV-2 polymerase chain reaction (PCR) tests administered from the COVID-19 Tracking Project [81].

#### COVID-19 death and case data cleaning

If a negative number of deaths or cases were reported on a given day—often due to retroactive changes in the reported cumulative death or case count for record deduplication or changes in data reporting by the state government—we assume that the cumulative death (case) count on that day was the correct one and set the number of deaths (cases) incident on prior days to zero until the overall cumulative count is non-decreasing. We begin modeling viral transmission in each state 21 days prior to the first day on which more than one death is reported.

#### NPI policy data cleaning

In converting the ordinal policy levels to numerical values, we followed OxCGRT’s methodology for calculating indices, in which ordinal levels are equally spaced numerically and a targeted (as opposed to general) intervention is treated as a half-step between ordinal levels. We rescale each policy value relative to the maximum level of stringency seen over 2020, such that their values lie between 0 and 1, with 1 denoting the most stringent policy. If a policy is not recorded on a given day, we set its value to that on the previous day on which the policy was recorded, or we set it to zero if at the beginning of the study period. We average daily policy values at the weekly level for our NPI regression model.

### Epidemiological model

We use a Bayesian epidemiological model to estimate SARS-CoV-2 prevalence and transmission rates in each US state in 2020. Our Bayesian Susceptible-Exposed-Infectious-Removed-Deceased (SEIRD) model builds on that of Irons and Raftery [55], which was used to estimate state-level SARS-CoV-2 prevalence in the first year of the pandemic based on reported cases, deaths, tests, and random testing surveys. To account for measurement error, idiosyncratic reporting, and overdispersion in viral transmission, we use a zero-inflated negative binomial likelihood for the COVID-19 death and case data linking them to the SEIRD model parameters.

For a given US state, let *S*(*t*) denote the proportion of susceptible people in the state on day *t*, *E*(*t*) the proportion exposed but not yet infectious, *I*(*t*) the proportion infectious, $R_S(t)$ the proportion recovered (survivors no longer infectious), $R_D(t)$ the proportion no longer infectious who will eventually succumb to the disease, and *D*(*t*) the proportion decedent. These quantities evolve in time according to the equations

1 $$\begin{aligned} \left\{ \begin{array}{ll} S(t+1) - S(t) & = - \beta (t)S(t)I(t) \\ E(t+1) - E(t) & = \beta (t)S(t)I(t) - \delta E(t) \\ I(t+1) - I(t) & = \delta E(t) - \gamma I(t) \\ R_S(t+1) - R_S(t) & = \gamma (1-\iota )I(t) \\ R_D(t+1)-R_D(t) & = \gamma \cdot \iota I(t) -\mu R_D(t) \\ D(t+1)-D(t) & = \mu R_D(t). \end{array}\right. \end{aligned}$$

**Fig. 1:**
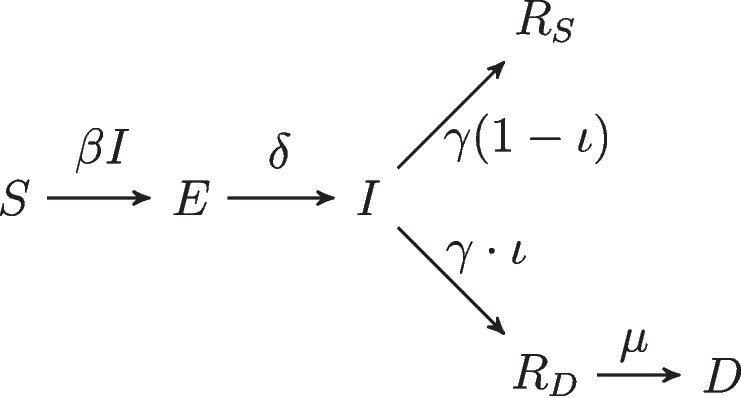
SEIRD model diagram

A model diagram of this process is depicted in Fig. 1.

Members of the population move from susceptible to exposed after contact with an infectious person with rate $\beta (t)$, which is allowed to vary in time to account for variation in exposure due to social distancing and other factors. Following the latent period (with duration $\delta ^{-1}$), exposed people become infectious and are subsequently removed at rate $\gamma$, at which point they no longer infect others. A proportion $\iota$ (the infection fatality rate, or IFR) of removed individuals die from COVID-19 at temporal rate $\mu$, and the rest remain alive.

As a simplifying approximation, our model assumes a conserved population, i.e., there are no births and no deaths due to competing risks:

$$\begin{aligned} S(t)+E(t)+I(t)+R_S(t)+R_D(t)+D(t) = 1 \end{aligned}$$

for all times *t*. Note that the time-varying basic reproduction number $R_0(t)$ and effective reproduction number $R_e(t)$, which describe rates of transmission in the initial and current population, respectively, are given by $R_0(t) = \beta (t)/\gamma$ and $R_e(t)=S(t)R_0(t)$.

We assume that $\gamma ^{-1}$, the average length in days of the infectious period, is determined by the disease and constant over time. We make the same assumption for the other biological parameters introduced above. In particular, while the IFR $\iota$ can realistically change over time, e.g., due to vaccination, the time period of our study focuses on viral transmission prior to widespread vaccine administration and circulation of novel SARS-CoV-2 strains with differential virulence. Estimates of the IFR over time in England based on regular random testing of the population found that, while the IFR did fluctuate in 2020, it hovered around 0.67% [82]. This is consistent with the IFR estimated in a systematic meta-analysis in 2020 [83], with the results of Irons and Raftery [55], and with our estimates discussed in the Results section.

As another simplifying approximation, our model does not account for waning immunity and reinfection, as acquired immunity was relatively long-lasting and reinfection within the first year of the pandemic was rare [84–93]. As those with prior infection were subject to a lower risk of death, this simplification circumvents the need to model the reduced IFR among reinfections. Also note that, as we model viral spread in the US states independently of each other, we do not explicitly track interstate transmission events.

Regarding prior specification, $R_0(t)$ is given a scaled beta-distributed random walk structure. We assume that $R_0(t)$ is constant during each week and, in an abuse of notation, write $R_0(w(t))$ to mean the value of $R_0$ in week *w*(*t*) to which day *t* belongs. We have

$$\begin{aligned} R_0(w+1)/R_0^{\max } & \sim \text {Beta}(\sigma ^2_R R_0(w)/R_0^{\max }, \sigma ^2_R(1-R_0(w)/R_0^{\max })), \\ R_0(0) & \sim \text {Uniform}(0,R_0^{\max }), \\ \pi (\log \sigma ^2_R) & \propto 1. \end{aligned}$$

The prior on $R_0(w+1)$ is centered at $R_0(w)$. We place a flat improper prior $\pi$ on the log-transformed scale parameter $\log \sigma ^2_R$. We take $R_0^{\max }=6.5$ to be the upper bound for the transmission rate based on [94]. We place a flat Dirichlet prior on the initial SEIRD components:

$$\begin{aligned} S(0) & = (1-p) + p\cdot x_0(S), \\ E(0) & = p\cdot x_0(E), \\ I(0) & = p\cdot x_0(I), \\ R_S(0) & = p\cdot (x_0(R)+x_0(D))(1-\iota ), \\ R_D(0) & = p\cdot x_0(R)\cdot \iota , \\ D(0) & = p\cdot x_0(D)\cdot \iota , \\ (x_0(S),x_0(E),x_0(I),x_0(R),x_0(D)) & \sim \text {Dirichlet}(1,1,1,1,1), \end{aligned}$$

Here $p = 0.05$ is the upper bound on the proportion of the population potentially infected at or before time 0 (the first day of the study period). The remaining parameters are detailed in Additional file 1: Table S1. We specify state-specific priors for the IFR using a normal distribution truncated to the unit interval based on the posterior median and 95% credible interval reported by Irons and Raftery [55]:

$$\begin{aligned} \iota _s \sim \text {Normal}_{[0,1]}(\hat{\iota }_s,\hat{\sigma }_{s}). \end{aligned}$$

Here $\hat{\iota }_s$ is the posterior median IFR in state *s* and $\hat{\sigma }_s$ is obtained by dividing the width of the 95% credible interval by 4 (the “range over 4” rule).

#### Likelihood on deaths

In a given US state, let *d*(*t*) and *c*(*t*) denote the number of COVID-19 deaths and cases reported in the state on day *t*, as recorded by Dong et al. [79]. To account for measurement error, idiosyncratic reporting, and overdispersion in viral transmission [68–73], we use a zero-inflated negative binomial model on *d*(*t*) and *c*(*t*). Many states inconsistently reported cases and deaths, often taking breaks over weekends and holidays, resulting in numerous spurious zeros in the data. We address this by assuming that any deaths or cases occurring on such a day are reported on the first subsequent day of accurate reporting.

Specifically, let $Z_t$ indicate the event that the number of deaths occurring on day *t* is incorrectly reported as 0. We assume that the $Z_t$ are independent and identically distributed with $P(Z_t=1)=\theta _D$. We know that $Z_t=0$ on days with reported deaths ($d(t)>0$) and our model conditions on this knowledge. Assume $t_0<t_0+k$ are days with $d(t_0)>0$ and $d(t)=0$ for all $t\in (t_0,t_0+k]$. Note that some of the zeros on days $t\in (t_0,t_0+k]$ could be due to misreporting, whereas others could be accurate reporting days on which zero deaths actually occurred. We marginalize over the unknown random variables $Z_t$ for $t\in (t_0,t_0+k]$ conditional on the assumptions that: the reported deaths $d(t_0)$ are centered at $m_D(t_0) = N\mu R_D(t_0)$, the true number of deaths on day $t_0$; in expectation, any deaths occurring on misreporting days are reported on the next day of accurate reporting. Under these assumptions, the underlying mean $\tilde{m}_D(t_0+k)$ of reported cases $d(t_0+k)$ conditional on $Z_{t_0+k}=0$, after marginalizing over $Z_t,t\in (t_0,t_0+k)$, can be expressed in terms of the true number of deaths $m_D(t)$ occurring on past days as

$$\begin{aligned} \tilde{m}_D(t_0+k) & = \mathbb {E}[d(t_0+k)|Z_{t_0+k}=0] \\ & = \mathbb {E}\left[ m_D(t_0+k)+\sum \limits _{t\in (t_0,t_0+k)}Z_t m_D(t)\right] \\ & = \sum \limits _{t=0}^{k-1} m_D(t_0+k-t)\theta _D^{t}. \end{aligned}$$

The likelihood on observed deaths is then given by the following zero-inflated negative binomial:

2 $$\begin{aligned} P(d(t) & =d\ |\ \tilde{m}_D(t),\kappa _D(t),\theta _D)\nonumber \\ & = \left\{ \begin{array}{ll} \theta _D +(1-\theta _D)\cdot \text {NegBin2}(0;\tilde{m}_D(t),\kappa _D(t)^{-1}), & d=0, \\ (1-\theta _D)\cdot \text {NegBin2}(d;\tilde{m}_D(t), \kappa _D(t)^{-1}), & d>0, \end{array}\right. \end{aligned}$$

where NegBin2$(y;\mu ,\tau )$ is parametrized to have mean $\mu$ and variance $\mu +\mu ^2/\tau$. We allow the overdispersion parameter $\kappa _D(t)$ to depend on the mean as follows:

$$\begin{aligned} \kappa _D(t)^{-1} = \kappa _D^{-1}\left( \zeta _D \tilde{m}_D(t) + (1-\zeta _D)\right) , \end{aligned}$$

where $\zeta _D\in [0,1]$ is a proportion parameter and $\kappa _D\in (0,\infty )$. We use $\text {Uniform}(0,1)$ priors on $\theta _D$ and $\zeta _D$ and a flat improper prior $\pi (\log \kappa _D)\propto 1$. Finally, with $\tilde{d}(0)$ representing the cumulative deaths reported prior to the start of the modeling window, we use the likelihood

$$\begin{aligned} \tilde{d}(0)\sim \text {Poisson}(N\cdot (R_D(0)+D(0))), \end{aligned}$$

where *N* is the state’s total population.

This model flexibly interpolates between a count distribution with a linear mean-variance relationship (as with the overdispersed Poisson) when $\zeta _D=1$ and a quadratic mean-variance relationship (as with the usual negative binomial) when $\zeta _D=0$. We found that this modification was necessary to accurately capture dispersion in clinical data across a range of states. In some states, such as Ohio and Indiana, a standard Poisson was sufficient to produce well-calibrated posterior predictive distributions. In most other states, such as Texas and Florida, a negative binomial was required. Finally, there were some states, such as New York, in which Poisson predictive intervals were too narrow and negative binomial intervals were too wide, while predictive intervals derived from the model (2) were much better calibrated. Our model (2) can handle all of these cases.

#### Likelihood on cases

Let $\nu (t)=N\beta (t)S(t)I(t)$ denote the number of new infections in the state on day *t*. We relate the true prevalence $\nu (t)$ to the number of cases *c*(*t*) reported on each day using a compartmental model that accounts for time-varying imperfect case ascertainment and delays between exposure and case confirmation via testing. We define a “number of infections waiting to be confirmed” compartment $I_C(t)$ satisfying

$$\begin{aligned} I_C(t+1) = I_C(t)(1-\tau ) + \text {CAR}(t+1)\nu (t+1), \end{aligned}$$

where $\tau ^{-1}$ is the expected delay in days from infection to case confirmation and $\text {CAR}(t)$ is the case ascertainment rate on day *t*. We use a truncated normal prior on the case confirmation delay based on [95]:

$$\begin{aligned} \tau \sim N_{(\tau _D-8.053-1.96\cdot 4.116,\infty )}(\tau _D - 8.053,4.116), \end{aligned}$$

where $\tau _D$ is the mean total time from infection to death

$$\begin{aligned} \tau _D=\delta ^{-1}+\gamma ^{-1}+\mu ^{-1} = 21.0. \end{aligned}$$

The underlying mean $\tilde{m}_C(t)$ of *c*(*t*) on misreporting days $t=t_0+k$ is analogous to that for deaths, $\tilde{m}_D(t)$, with the expected number of deaths on accurately reported days $t_0$, $N\mu R_D(t_0)$, replaced by the expected number of infections confirmed on day $t_0$, $\tau I_C(t_0)$:

$$\begin{aligned} m_C(t_0) & = \tau I_C(t_0), \\ \tilde{m}_C(t_0+k) & = \sum \limits _{t=0}^{k-1} m_C(t_0+k-t)\theta _C^{t}. \end{aligned}$$

The zero-inflated negative binomial likelihood is then

$$\begin{aligned} P(c(t) & =c\ |\ \tilde{m}_C(t),\kappa _C(t),\theta _C)\\ & = \left\{ \begin{array}{ll} \theta _C + (1-\theta _C)\cdot \text {NegBin2}(0;\tilde{m}_C(t),\kappa _C(t)^{-1}), & c=0, \\ (1-\theta _C)\cdot \text {NegBin2}(c;\tilde{m}_C(t),\kappa _C(t)^{-1}), & c>0, \\ \end{array}\right. \\ \kappa _C(t)^{-1} & = \kappa _C^{-1}\left( \zeta _C \tilde{m}_C(t) + (1-\zeta _C)\right) . \end{aligned}$$

We use $\text {Uniform}(0,1)$ priors on $\theta _C$ and $\zeta _C$ and flat improper priors $\pi (\log \kappa _C)\propto 1$ and $\pi (\log I_C(0))\propto 1$. We place a beta-distributed random walk prior on case ascertainment rates:

$$\begin{aligned} \text {CAR}(0) & \sim \text {Uniform}(0,1), \\ \text {CAR}(t+1) & \sim \text {Beta}(\sigma ^2_{\text {CAR}}\text {CAR}(t),\sigma ^2_{\text {CAR}}(1-\text {CAR}(t))), \\ \pi (\log \sigma ^2_{\text {CAR}}) & \propto 1. \end{aligned}$$

In Additional file 1: Section S.4, we plot posterior estimates of the zero-inflation parameter $\theta _C$, overdispersion parameter $\kappa _C(t)^{-1}$, and case ascertainment rate $\text {CAR}(t)$ for all US states.

### Effects of NPIs on SARS-CoV-2 transmission

Turning to our robust regression model linking NPI policies to the dynamics of viral transmission, we rely on the OxCGRT dataset, which aggregates government policy responses to the pandemic starting from January 1, 2020 [80]. OxCGRT tracks a range of containment and closure indicators with numerical values corresponding to the strength of the response on each day. Our regression model focuses on the following 11 policy indicators: school closure, workplace closure, public event cancellation, restrictions on gatherings, public transport closure, stay-at-home requirements, restrictions on internal movement, public information campaigns, testing, contact tracing, and facial covering policies.

For each US state *s*, we define $u^{(s)}(t)$, an 11-dimensional vector with entries in the interval [0, 1] denoting the strength of each NPI implemented on day *t*. So $u_k^{(s)}(t) = 0$ represents no restrictions associated to the *k*th NPI on day *t* (e.g., no school closure), whereas $u_k^{(s)}(t) = 1$ represents the strictest restrictions (e.g., full school closure). NPI implementation during the pandemic was highly correlated, which poses a challenge to teasing apart the effects of individual NPIs on SARS-CoV-2 transmission. We utilize a Bayesian hierarchical model (BHM) to jointly model the weekly time-varying basic reproduction number $R_0^{(s)}(w)$ in each US state *s* and week *w* as a function of NPIs. The BHM leverages spatiotemporal variation in NPI implementation over time across states in order to estimate their effects. It allows for spatial heterogeneity in NPI effects (e.g., due to differential adherence to government mandates) while enabling identification via partial pooling of information across the country.

Our robust regression model expresses $R_0^{(s)}(w)$ as a log-linear function of the NPIs implemented in that week, $u^{(s)}(w)$, where we obtain weekly values for the NPIs by averaging over days. To account for the endogenous behavioral response to the fear of infection, we also control for the expected population proportion of deaths $\tilde{D}^{(s)}(w-1)$, removals $\tilde{R}^{(s)}(w-1)$, and infections $\tilde{I}^{(s)}(w-1)$ incident in the prior week. We consider three models controlling for (i) deaths only; (ii) removals and deaths; and (iii) infections, removals, and deaths. Given the delay in case confirmation, model (ii), in which individual behavior responds to deaths and removals but not infections incident in the prior week, may be preferred on theoretical grounds. Nevertheless, our qualitative findings are consistent across models, with the main distinction being that controlling for more effects tends to attenuate the effect of school closure on transmission rates. For brevity, in the main text we report results for model (ii) only; estimates for the other models are provided in Additional file 1: Section S.2.

For notational convenience, we first define the linear predictor

$$\begin{aligned} \log \hat{R}_0^{(s)}(w) & := \log R_0^{(s)} + \beta ^{(s)}_u\cdot u^{(s)}(w) \\ & \quad + \beta ^{(s)}_I\tilde{I}^{(s)}(w-1) + \beta ^{(s)}_R\tilde{R}^{(s)}(w-1) \\ & \quad + \beta ^{(s)}_D\tilde{D}^{(s)}(w-1), \end{aligned}$$

where $R_0^{(s)}$ is the initial state-specific basic reproduction number under no restrictions, $\beta _u^{(s)}$ is a vector of state-specific random NPI effects of size $p=11$, $\beta ^{(s)}_D$ denotes the random effect of deaths, and similarly for $\beta ^{(s)}_I,\beta ^{(s)}_R$. Models (i) and (ii) assume $\beta ^{(s)}_I=\beta ^{(s)}_R=0$ and $\beta ^{(s)}_I=0$, respectively. Our final model on the observed transmission rate $R_0^{(s)}(w)$, which is output by the SEIRD model, also includes an autoregressive component:

3 $$\begin{aligned} \log R_0^{(s)}(w) & = \log \hat{R}_0^{(s)}(w)\nonumber \\ & \quad + \varphi \left( \log R_0^{(s)}(w-1)-\log \hat{R}_0^{(s)}(w-1)\right) \nonumber \\ & \quad + \varepsilon ^{(s)}(w), \end{aligned}$$

where $\varphi$ is the AR(1) parameter and $\varepsilon ^{(s)}(w)$ is a Student-*t* distributed error term with $\nu _\varepsilon$ degrees of freedom:

$$\begin{aligned} \varepsilon ^{(s)}(w)\sim \text {Student-}t(\nu _\varepsilon ,\sigma ^2_\varepsilon ). \end{aligned}$$

We considered AR(*q*) models for $q=1,2,3$. We selected $q=1$ via leave-one-out cross validation (LOO-CV) [96]. Similar model specifications have appeared elsewhere in the literature [8, 10, 97]. The error terms account for unpredictable and heavy-tailed exogenous shocks that may have sustained effects on transmission (as modeled through the AR(1) term), such as the start of a new wave due to a super-spreader event or the introduction of new infections from an external source (e.g., due to travel into the state). The use of Student-*t* distributed errors ensures that the regression model is robust to outliers, which prevents over-fitting the effects of NPIs to the transmission data. We use a flat improper prior on $\log \nu _\varepsilon$ and find that the posterior of $\nu _\varepsilon$ concentrates between 2 and 3, indicating that a heavy-tailed error distribution is appropriate.

We control for deaths $\tilde{D}$, removals $\tilde{R}$, and infections $\tilde{I}$ incident in the prior week following the identification strategy of a number of other studies estimating the causal effects of NPIs [15, 16, 60, 61, 98–101]. The existence of substantial voluntary social distancing and its pronounced economic effects in the US and elsewhere have been well-documented in numerous empirical analyses [15, 98, 102–109] and derived from first principles in microeconomic modeling [27, 28, 30, 110]. In response to SARS-CoV-2 outbreaks, people began social distancing (and, potentially, other protective measures, e.g., mask-wearing) prior to the onset of restrictions and subsequently increased social activity separate from the lifting of restrictions. As a result, declines in mobility, consumer spending, and hours worked cannot be fully attributed to the effects of NPI policies [111, 112]. Including prior deaths, removals, and infections as covariates in the regression model accounts for changes in protective behaviors by individuals responding to the risk of infection.

**Fig. 2:**
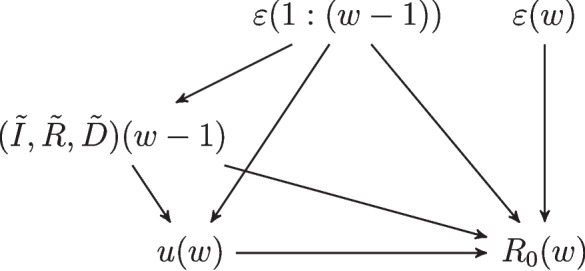
NPI regression model DAG

Figure 2 depicts a directed acyclic graph (DAG) representing our causal model for the outcome $R_0(w)$ in each week. We suppress the state *s* for compactness of notation. Deaths, removals, and infections incident in week $w-1$ may affect the policy response *u*(*w*) and the transmission rate $R_0(w)$ in the following week, with the latter effect representing the endogenous behavioral response (including social distancing and other protective measures) to the fear of infection. The transmission rate $R_0(w)$ is also a function of NPI policies *u*(*w*) and exogenous shocks $\varepsilon (1:w):=\{\varepsilon (v):v=1,\ldots ,w\}$. We allow for past shocks $\varepsilon (1:w-1)$ to affect past deaths, removals, and infections and the current policy response *u*(*w*). In our regression model (3), the lagged and attenuating effect of past shocks on the transmission rate is captured by the AR(1) term:

$$\begin{aligned} \varphi \left( \log R_0^{(s)}(w-1)-\log \hat{R}_0^{(s)}(w-1)\right) = \sum \limits _{v=1}^{w-1} \varphi ^{w-v}\varepsilon ^{(s)}(v). \end{aligned}$$

Given the DAG in Fig. 2, we see that controlling for $(\tilde{I}(w-1),\tilde{R}(w-1),\tilde{D}(w-1))$ and $\varepsilon (1:w-1)$—as we do in (3)—blocks all back-door paths from *u*(*w*) to $R_0(w)$. As such, the effect of NPIs is identified in this model following the back-door criterion [113]. This model was selected by LOO-CV among models controlling for both deaths and cases in the past *x* weeks, where *x* was fixed at 1, 2, 4, 6, 8, 10, 12, 16 and also allowed to vary as a parameter in the model.

We assume that the NPIs in place in week *w* directly affect transmission rates in week *w* (a zero-week lag). This is in line with a number of other studies estimating the effects of NPIs (e.g., Flaxman et al. [8], Brauner et al. [9], and Sharma et al. [10]), which do not account for lagged effects of NPIs. This is not an issue for closure policies (e.g., school, business, and transit closures), as they take effect immediately. For other policies, it is reasonable to assume that behavioral responses occur quickly. For example, Alexander and Karger [114] find that mobility and consumer spending declined consistently within 2 days of when US counties enacted stay-at-home orders. To assess the effect of lagging the NPIs, we conduct a covariance analysis in Additional file 1: Section S.2. We find that the magnitude of the covariance between NPIs and transmission rates is maximized by a zero-week lag, which suggests that it is appropriate.

Regarding prior specification for the regression (3), we use a hierarchical model for the state-specific coefficients $\xi ^{(s)}=(\beta ^{(s)}_u,\beta ^{(s)}_I,\beta ^{(s)}_R,\beta ^{(s)}_D,R_0^{(s)})\in \mathbb {R}^{p+4}$, which enables partial pooling of information. Here $p=11$, the number of NPIs in the model, is the length of the vector $\beta ^{(s)}_u$ of NPI effects. With $\xi$ denoting the global pooled effects, we have

$$\begin{aligned} \xi ^{(s)} & \sim \text {Normal}(\xi ,V)\prod _{k=1}^p I\left( \xi ^{(s)}(k)\le 0\right) ,\nonumber \\ V & = D(\lambda )\Omega D(\lambda ), \nonumber \\ \Omega & \sim \text {LKJ}(\zeta =1), \nonumber \\ \lambda (j) & \sim \text {Student-}t_{[0,\infty )}(0,2.5^2,3), \quad j=1,\ldots ,p+4, \nonumber \\ \sigma _\varepsilon & \sim \text {Student-}t_{[0,\infty )}(0,2.5^2,3), \nonumber \\ \pi (\log \nu _\varepsilon ) & \propto 1,\nonumber \\ \varphi & \sim \text {Uniform}(-1,1). \end{aligned}$$

The truncated normal prior on the state-level random effects $\xi ^{(s)}$ assumes that they are centered around the pooled effects $\xi$ and that NPIs cannot increase the transmission rate ($\xi ^{(s)}(k)\le 0$), in line with the results of numerous studies estimating the effects of NPIs [8–13, 17, 18, 106, 115]. (Note that $\xi ^{(s)}(k)=\beta ^{(s)}_u(k)$ is the *k*th NPI effect.) For the remaining parameters, we use the default prior specification for multilevel models used in the *brms* R package [116]. The covariance matrix *V* of the random effects is decomposed as the product of a correlation matrix $\Omega$ given an LKJ prior with parameter $\zeta =1$ (specifying a uniform prior on correlation matrices), and a diagonal matrix $D(\lambda )$ with entries $\lambda (j)$ given Student-*t* priors with 3 degrees of freedom truncated to be non-negative. The error standard deviation $\sigma _\varepsilon$ is given the same truncated Student-*t* prior. The log degrees of freedom $(\log \nu _\varepsilon )$ for the Student-*t* error terms $\varepsilon ^{(s)}(w)$ is given a flat improper prior. The AR(1) parameter $\varphi$ is given a $\text {Uniform}(-1,1)$ prior.

#### Accounting for seasonality of transmission

To account for potential seasonality of SARS-CoV-2 transmission [117–119], we also included average daily temperature measurements reported for the largest population centers in each state as a covariate. However, models with temperature as a covariate were excluded based on model selection with LOO-CV [96]. We obtained daily average surface temperature data for the largest city in each state using Python’s *Meteostat* package [120].

#### Propagating uncertainty

Let $\mathcal {D}$ denote the observed case and death data, $\mathcal {U}$ the NPI data, $\mathcal {S}$ the SEIRD model parameters, and $\eta$ the NPI regression model parameters. Our two-stage estimation procedure appropriately propagates uncertainty such that the resulting estimates approximate the posterior distribution $\pi (\eta |\mathcal {D},\mathcal {U})$ that would be obtained from combining the epidemiological and regression stages into a single model. Indeed, we have

$$\begin{aligned} \pi (\eta |\mathcal {D},\mathcal {U}) & = \int \pi (\eta ,\mathcal {S}|\mathcal {D},\mathcal {U}) d\mathcal {S} \\ & = \int \pi (\eta |\mathcal {S},\mathcal {D},\mathcal {U})\pi (\mathcal {S}|\mathcal {D},\mathcal {U}) d\mathcal {S} \\ & = \int \pi (\eta |\mathcal {S},\mathcal {D},\mathcal {U})\pi (\mathcal {S}|\mathcal {D}) d\mathcal {S}\\ (\text {SEIRD} & \ \text {parameters}\ \mathcal {S}\ \text {can be inferred from clinical data}\ \mathcal {D}\ \text {alone})\\ & \approx \frac{1}{M}\sum \limits _{i=1}^M \pi (\eta |\mathcal {S}^{(i)},\mathcal {D},\mathcal {U}), \end{aligned}$$

where $\mathcal {S}^{(i)}$ denotes the *i*th of *M* posterior trajectories of the SEIRD parameters (e.g., the time-varying basic reproduction number $R_0^{(s)}(w)$ in each state *s*) derived from the first-stage transmission model posterior $\pi (\mathcal {S}|\mathcal {D})$. Here we use $M=100$ randomly sampled trajectories of $R_0^{(s)}(w)$, which define the dependent variable in the NPI regression model. For each $i=1,\ldots ,M$ we generate samples from the NPI model posterior $\pi (\eta |\mathcal {S}^{(i)},\mathcal {D},\mathcal {U})$. We then aggregate these samples to obtain our final estimate of the full posterior $\pi (\eta |\mathcal {D},\mathcal {U})$.

### Evaluating and optimizing the costs of NPIs

The SEIRD model combined with the NPI regression model (3) define a simulator for the trajectories of infections and deaths under counterfactual NPI policies, conditional on the parameters estimated using the NPI and clinical data, $\mathcal {U}$ and $\mathcal {D}$, respectively. Evaluating the cost-effectiveness of NPIs and determining optimal strategies requires accounting for the aggregate costs incurred in implementing policies and their consequent health impacts.

We take the standard approach within statistical decision theory [54] of using a cost function given by the expected risk (akin to expected utility in game theory), which averages over uncertainty in: the effectiveness of NPIs; the costs of infections and interventions; and the trajectories of incident infections and deaths. Namely, for an NPI policy $\textbf{u}=\{u(t)\}_{t=1}^T$ implemented in a state on the days $t=1,\ldots ,T$, we define its associated cost $\mathcal {C}(\textbf{u})$ as the sum of the posterior expected costs incurred by infections and NPI implementation:

4 $$\begin{aligned} \mathcal {C}(\textbf{u}) = \mathbb {E}\left[ c_{\text {NPI}}(\textbf{u}) + \frac{1}{N} \left. \sum \limits _{t=1}^T c_\nu \nu (t)\right| \mathcal {D},\mathcal {U}\right] , \end{aligned}$$

where $\nu (t)=N\beta (t)S(t)I(t)$ is the number of new COVID-19 infections in the state incident on day *t* under the policy $\textbf{u}$. Here $c_\nu$ is the average cost in USD2020 associated to a COVID-19 infection and

$$\begin{aligned} c_{\text {NPI}}(\textbf{u}) = \sum \limits _{k=1}^p c_k(\textbf{u}_k) \end{aligned}$$

is the average per capita cost in USD2020 associated with implementing the policy $\textbf{u}=(\textbf{u}_1,\ldots ,\textbf{u}_p)$, where $c_k$ is the cost of the *k*th NPI. We define these quantities below. In practice, we cannot directly evaluate the expectation (4). Instead, we use posterior trajectories of infections $\nu (t)$ to approximate (4) via Monte Carlo. Finally, as our cost function averages over uncertainty in the parameters, note that we obtain a single optimal control strategy over time for each state rather than a posterior distribution of strategies. Given the relatively short duration of our study period (i.e., the first year of the pandemic), we do not adjust the cost function for temporal discounting.

As we detail below, we quantify the average cost of a COVID-19 infection as a sum of average life costs (due to COVID-19 deaths), medical costs (incurred by treatment), productivity costs (due to worker absenteeism), and costs associated to voluntary social distancing in response to the fear of infection [104, 121–129]. We quantify the cost of workplace closures and social distancing measures through their effects on employment, which have been thoroughly studied in the COVID-19 economics literature [37, 99, 100, 102, 103, 114, 130, 131]. The cost of school closures is a sum of productivity loss due to worker absenteeism (as parents of children out of school miss work to care for their kids) and learning loss resulting from students missing school and receiving lower quality education through distance learning [5, 57–59, 63, 65, 66]. Finally, we quantify the procurement costs associated to COVID-19 testing, tracing, and masking [81, 127, 132–138].

We used the *optimParallel* R package for NPI policy optimization [139]. To determine the optimal NPI strategy in each state, we used a combination of 8 random and hand-specified parameter initializations and kept the policy yielding the smallest value of the cost function. In practice, we found that the results of the optimization were robust to the initial parameters, which is reflected in our results and sensitivity analysis.

#### Cost of infections

The average cost of a COVID-19 infection is a sum of average life costs (due to COVID-19 deaths), medical costs (incurred by treatment), productivity costs (due to worker absenteeism), and costs associated with voluntary social distancing in response to the fear of infection.

The life cost associated with a COVID-19 infection deserves some discussion, as it dominates the aggregate cost of infection and it requires ascribing a dollar value to death, which can be a contentious issue. In cost-benefit analysis, the standard approach to quantifying mortality risk reductions as a result of public policy in monetary terms is through the value of a statistical life (VSL), commonly estimated at about $11 million in USD2020 [21]. In our context, the relevant quantity is the value of a statistical COVID-19 death (VSCD). As Robinson et al. [126] note, COVID-19 deaths are concentrated in the oldest age groups and the decision to adjust the VSCD for the age profile of COVID-19 mortality or not can vastly alter the conclusions of a cost-benefit analysis. As such, we conduct sensitivity analysis of our results using high and low estimates of the VSCD reported in [126]. In line with a number of other studies in the COVID-19 economics literature [21, 22, 24, 27], we use as our baseline the low estimate of $4.47 million, which is based on the average years of life lost to a COVID-19 death and a constant value per statistical life year. (That is, outside of our sensitivity analysis in Additional file 1: Section S.1, all estimated costs are based on the low VSCD.) The high estimate of $10.63 million assumes that the VSCD equals the population average VSL (i.e., it does not adjust the VSL for the age pattern of COVID-19 deaths). Additional file 1: Table S2 records the value of this and other economic parameters used in our study. Denoting the VSCD by $$c_{\text {VSCD}}$$, the average life cost per COVID-19 infection in state *s* is then $$c_{\text {VSCD}}\cdot \tilde{\iota }^{(s)}$$, where $$\tilde{\iota }^{(s)}$$ is the posterior average state-level IFR.

To quantify the cost associated with voluntary social distancing, we rely on the results of Aum et al. [104], who find that a one per thousand increase in the COVID-19 case rate caused a 2.68% drop in employment in South Korea in the spring of 2020, with similar (although non-causal) estimates in the US and UK. We adjust their numbers for under-reporting of cases based on the number of deaths and cases in South Korea in their period of study reported by Our World in Data [140]. By February 29, 2020, South Korea had 556 cumulative confirmed cases, and by March 7 it had 3526. The cumulative COVID-19 deaths 3 weeks later on March 21 were 75, and by March 28 were 104. With an IFR of 0.68% [83], we would expect between 11,029 and 15,294 infections with this number of deaths. This suggests a case ascertainment rate between 5% and 23% in that period. We average these two numbers, assuming 15.5% case ascertainment, which yields a $$0.155\cdot 2.68 \approx 0.42\%$$ drop in employment resulting from a one per thousand increase in COVID-19 prevalence. In line with Chetty et al. [2], Aum et al. [104] find that these impacts were felt most acutely among low-wage workers. As such, we phrase this increase in unemployment due to an infection—which is assumed to last the average duration of the infectious period ($$\gamma ^{-1}=5.0$$ days)—in monetary terms using the state’s median income. (We use national and state-level personal income data reported by U.S. Bureau of Economic Analysis [122]. To approximate state-level median income, we multiply the US median personal income by the state’s per capita income divided by the US per capita income.) In sum, we find that the fear cost per COVID-19 infection ranges from $1491 to $3199 across states with a median of $1999.

For the remaining parameters, we take $3045 as the average medical cost of a COVID-19 infection based on the estimates of Bartsch et al. [128] and DeMartino et al. [129]. We assume the average productivity cost of a COVID-19 infection is equal to one week of sick days at a state’s median wage, which yields costs similar to those reported by Skarp et al. [127]. These range from $505 to $1084 across states with a median of $677.

#### Cost of workplace closures and social distancing measures

Beginning in the spring of 2020, consumer spending dropped significantly in response to health concerns and government-mandated business closures and social distancing policies. This reduction in consumer spending—primarily on in-person services—was responsible for a large majority of the decline in US GDP in the second quarter of 2020. Declining business revenue led to substantial layoffs with subsequent unemployment increases concentrated among low-wage workers [2]. As such, we quantify the cost of workplace closures and social distancing measures through their effects on employment, which have been thoroughly studied in the COVID-19 economics literature [37, 99, 100, 102, 103, 114, 130, 131]. As above, we convert employment rate decreases in each state to monetary losses using the state’s median personal income, which reflects the wage distribution of pandemic job loss.

The effects of COVID-19 workplace closures on unemployment and consumer spending in the US were estimated by Barrot et al. [37], Crucini and O’Flaherty [100], and Gupta et al. [130], who arrive at broadly similar conclusions. Crucini and O’Flaherty [100] found that non-essential business closures led to a 1–2 percentage point decline in expenditures. Gupta et al. [130] found that 60% of the 12 percentage point decline in the employment rate between January and April 2020 was due to state policies, with government-mandated business closures and stay-at-home orders each accounting for half of those 7.2 percentage points. (Although drops in consumer foot traffic are not directly comparable to employment rate decreases, Goolsbee and Syverson [98] found that general shelter-in-place orders reduced overall consumer visits by 7 percentage points.) Barrot et al. [37] found that a 10 percentage point increase in the share of restricted labor was associated with a 3 percentage point decline in April 2020 employment. Given these findings, we define low, middle, and high scenarios in which workplace closures cause 2%, 4%, and 6% declines in the employment rate. We take the middle scenario as our baseline and consider the low and high scenarios in our sensitivity analysis in Additional file 1: Section S.1.

We define social distancing measures as the combination of the following six NPIs tracked by OxCGRT: stay-at-home orders, restrictions on gatherings, restrictions on internal movement, public information campaigns, public transit closures, and public event cancellations. We bundle these policies for a number of reasons: their implementation was highly correlated in time and space; there is a paucity of information on the individual economic effects of most of these interventions as the COVID-19 economics literature tends to focus on “social distancing” or “lockdown” measures broadly defined (likely due to their synchronous adoption); and they are blanket policies acting as relatively blunt instruments with their primary direct effects on the economy stemming from a common mechanism—namely, reduction in consumer spending on in-person services with consequent unemployment.

The effects of social distancing measures on unemployment and consumer spending in the US were studied by Coibion et al. [99], Crucini and O’Flaherty [100], Bodenstein et al. [103], Gupta et al. [130], and Baek et al. [131]. Drawing on survey responses, Coibion et al. [99] found that individuals in counties under lockdown were 2.8 percentage points less likely to be employed relative to other survey participants, had a 1.9 percentage point lower labor-force participation, and had a 2.4 percentage point higher unemployment rate. Crucini and O’Flaherty [100] found that stay-at-home orders caused a 4 percentage point decrease in consumer spending and hours worked. Bodenstein et al. [103] found that the combined effect of voluntary and mandatory social distancing could explain 6–8 percentage points of the 12% drop in US GDP in the second quarter of 2020 and that stay-at-home orders could account for a 2 percentage point increase in the unemployment rate. As mentioned above, Gupta et al. [130] found that stay-at-home orders led to a 3.6 percentage point decline in employment rates through April 2020. Similarly, Baek et al. [131] found that each week of stay-at-home order exposure between March 14 and April 4, 2020 yielded an increase in a state’s weekly unemployment insurance claims corresponding to 1.9% of its employment level. As Bartik et al. [102] note, nearly all employment declines occurred within the 2-week period March 14–28, which implies a cumulative 3.8% drop in the employment rate based on the findings of Baek et al. [131]. Hence, we assume that social distancing measures cause a 4% decline in the employment rate. In our sensitivity analysis, we do not vary the cost of social distancing measures as we are primarily interested in assessing the robustness of the optimal strategy and the relative costs of various policies rather than variation in the total cost incurred by each policy, which means that we are free to leave the value of one term in the cost function (4) fixed.

We note that the combined economic effects of workplace closures and social distancing measures used here are on par with trends in aggregate economic output in the US and elsewhere observed in 2020 and in prior pandemics. Congressional Budget Office [141] estimates a 3.5% year-over-year decline in real US GDP from 2019 to 2020. Analyzing trends in annual global GDP, Kaplan et al. [24] estimate a 7% decline from 2019 to 2020 due to COVID, which equates to a loss of $10 trillion. Demirguüç-Kunt et al. [142] find that national lockdowns led to a 10% decline in economic activity across Europe and Central Asia in the spring of 2020. Studying the 1918 Spanish flu pandemic, in which social distancing measures were the primary tools used to curtail viral spread, Barro et al. [143] estimate a cumulative loss in GDP per capita of 6% over 3 years.

#### Cost of school closures

The cost of school closures is a sum of productivity loss due to worker absenteeism (as parents of children out of school miss work to care for their kids) and learning loss resulting from students missing school and receiving lower quality education through distance learning.

Sadique et al. [57] and Lempel et al. [58] estimated the magnitude of direct GDP loss due to worker absenteeism resulting from extended school closures in the US and UK, respectively, and arrived at nearly identical numbers. They find that 4 weeks of school closure would cost 0.1–0.3% of GDP in the US and 0.1–0.4% in the UK. For our study, we use 0.2%. Similar estimates based on modeling studies are reviewed in Viner et al. [59].

Notably, Lempel et al. [58] also estimate the healthcare impacts of a 4-week school closure in the US, finding that it would lead to a reduction of 6% to 19% in key healthcare personnel. Similarly, Bayham and Fenichel [144] find that 15% of the healthcare workforce would be in need of childcare during a school closure and find that their absence from work could cause a greater number of COVID-19 deaths than school closures prevent. Pricing these health impacts is not straightforward, so we omit these considerations when defining the cost function. As such, we believe that our accounting of the costs of school-closure-related worker absenteeism is conservative.

While learning loss due to school closure can be viewed as a social cost, it can lead to substantial downstream economic costs as cohorts of students that missed significant schooling eventually enter the labor-force as less skilled and productive workers. Education economists have extensively studied the connections between time spent in school, performance on standardized tests, and subsequent impacts on lifetime earning and GDP with findings that are consistent across contexts. Hanushek and Woessmann [65] and Psacharopoulos et al. [66]—whose assessments of the cost of learning loss we use—provide discussion and references. As our high scenario, we use the estimate of Hanushek and Woessmann [65], who find that cohort learning loss equivalent to one-third of a school-year has a staggering net present value equal to 69% of current-year GDP. Psacharopoulos et al. [66] arrive at a much smaller number, finding a 9% GDP loss arising from 0.33 years of lost schooling, which forms our low scenario and also our baseline value. We note that the results of Psacharopoulos et al. [66] are predicated on the assumption that remote learning is 90% as effective as in-person school, which is a likely source of the large discrepancy between the two estimates. While distance learning certainly mitigated some learning loss [5], and keeping schools open during the pandemic would have also incurred some learning loss due to student and teacher illness-related absenteeism, we believe that this assumption leads to a conservative estimate of the cost of learning loss associated to in-person school closure. Nevertheless, we find that optimal NPI strategies based on this low estimate involve no closure of schools beyond the usual 16 weeks of break per year.

In their systematic review and meta-analysis, Betthäuser et al. [5] find a substantial and consistent learning deficit of 0.35 school-years of learning loss across 15 high- and middle-income countries, which accrued early in the pandemic. This learning gap persisted but ceased to grow beyond 0.35 school-years, which suggests that remote learning did mitigate learning loss with greater efficacy (relative to in-person schooling) as time went on. In our cost function, we account for the improving quality of remote learning over time by assuming that the amount of learning loss incurred by 1 week of school closure equates to one school-week initially and decreases linearly to 0 as a function of the cumulative number of past weeks spent under school closure, such that 0.35 school-years is the maximal cumulative amount of learning loss possible. Furthermore, our cost function only accounts for marginal learning loss (i.e., beyond what would be expected after summer break, for example) by assuming that learning loss only begins to accrue once the duration of school closure exceeds 16 weeks.

#### Cost of testing, tracing and masking

We quantify the per capita cost of a week-long mask mandate as the price of supplying an individual with masks for a week. Following Bartsch et al. [132], we assume personal mask expenditure of $0.32 per day or, equivalently, $2.24 per week, which approximates the cost of one surgical mask per day or one N95 mask per week [127].

In the first year of the pandemic, US states steadily ramped up the number of SARS-CoV-2 PCR tests administered each day at a consistent linear pace. Indeed, after running least-squares regression of the cumulative number of tests administered in a state on each day against time (squared) using test data obtained from the COVID-19 Tracking Project [81], we obtain $$R^2$$ values above 0.97 for all states. Across states, the linear rate of testing capacity increase varies from an additional 7 to 40 tests per million population per day. Our cost function accounts for this by assuming that the number of tests administered in a given week under a testing policy is a linear function of the cumulative number of past weeks spent with test availability, with the slope given by the state-specific rate of testing capacity increase obtained from the regression. This yields the total number of tests administered in a state in any given week under the specified testing policy. We convert this quantity to a dollar value assuming that each test costs $100 based on Skarp et al. [127], Lo et al. [133], and Sharfstein [134], which includes the cost of procuring the test as well as labor for sample extraction and diagnostic lab testing.

We similarly assume that, while contact tracing policies are in place, tracing capacity ramps up at a linear pace. This is in line with increases over time in capacity reported in wide-scale assessments of US contact tracing programs [137, 138], as well as general increases over time in state-level hiring of contact tracers reported in media [145, 146]. Fitting a log-normal model to data from Lash et al. [137], we estimate the mean number of cases interviewed per week per 100,000 population to be 95.0 during their period of study, June–October 2020. Similarly, based on data from Rainisch et al. [138], we estimate a mean of 170.5 cases interviewed per week per 100,000 population during November 2020–January 2021. With 4 months separating August and December 2020 (the midpoints of the respective study periods), we therefore assume an increase in capacity of $$(170.5 - 95.0)/16 \approx 4.72$$ cases interviewed per 100,000 population per week while contact tracing policies are active. We convert this number to a dollar value based on the average cost of contact tracing per index case. Fields et al. [135] report the hourly cost of contact tracing at $$\$107.22/4.16 \approx \$25.77$$. According to Spencer [136], the median caseload per investigator during their 2-week evaluation period was 31. Assuming a 40-h work week, this implies a cost per case of $$\$25.77\times 80/31 \approx \$66.50$$. This number, which we take as our cost of contact tracing per index case, is near the midpoint of the interval reported in Skarp et al. [127] ($40.73–$93.59) based on different data. Additional file 1: Table S3 records the testing, tracing, and masking cost parameters with references.

## Results

### Estimating transmission dynamics

**Fig. 3:**
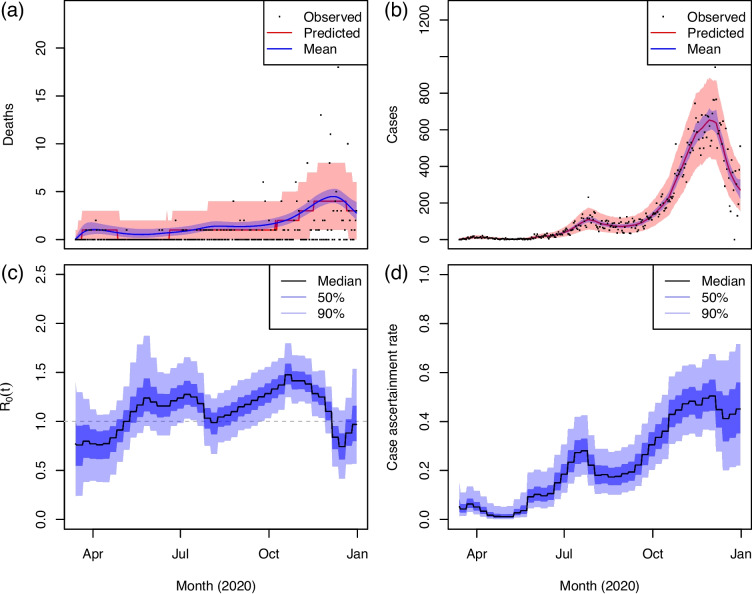
SEIRD model fit to COVID-19 data in Alaska. Top panels: observed (**a**) deaths *d*(*t*) and (**b**) cases *c*(*t*) are plotted in black. Median and 90% credible intervals of the posterior predictive distributions of *d*(*t*) and *c*(*t*) are in red. Posterior median and 90% credible intervals of the underlying mean parameters $m_D(t)$ and $m_C(t)$ are in blue. Bottom panels: posterior median, 50%, and 90% credible intervals for (**c**) the basic reproduction number $R_0(t)$ and (**d**) the case ascertainment rate $\text {CAR}(t)$

We estimate time-varying SARS-CoV-2 prevalence and transmission rates in all US states in 2020 using our Bayesian SEIRD model detailed in Methods section. Figure 3 displays model fits to clinical COVID-19 data for a specific state, Alaska, as well as estimates of the time-varying basic reproduction number $R_0$ and case ascertainment rate. Fits for the remaining states are included in Additional file 1: Section S.4. As a small state by population with noisy, zero-inflated death data, Alaska demonstrates the performance of our statistical model, which provides both (i) well-calibrated predictive intervals for the observed data and (ii) sensible, smooth estimates of the dynamics of the underlying mean parameters, even when the data appear extremely noisy, by accounting for zero-inflation, overdispersion, and idiosyncratic reporting.

**Fig. 4:**
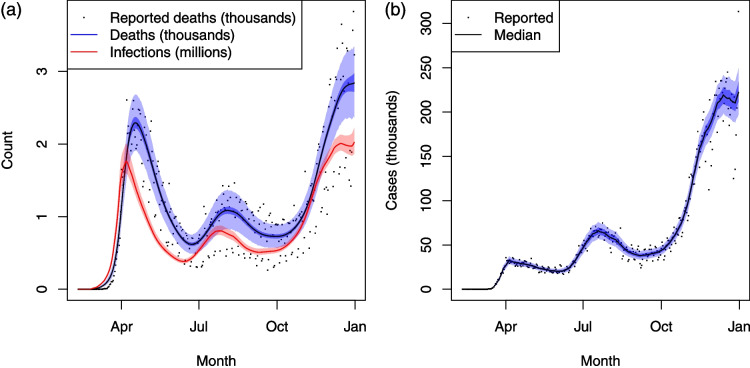
State-specific SEIRD results aggregated to the US. **a** Observed deaths *d*(*t*) are in black. Posterior median and 90% credible intervals of the underlying mean $m_D(t)$ are in dark blue. 90% credible intervals of the posterior predictive distribution of *d*(*t*) are in light blue. Posterior median and 90% credible intervals for active viral prevalence *I*(*t*) are in red. **b** Reported cases *c*(*t*) (scatterpoints) and posterior predictive median of *c*(*t*) (line) are in black; posterior 50% and 90% predictive intervals for *c*(*t*) are shaded in dark and light blue, respectively

Aggregating the state-level results, we estimate that there were 58.5 (55.7–62.7) million COVID-19 infections in the US in 2020, representing about 18% of the population. Weighting the posterior state-level IFR estimates by the proportion of 2020 US SARS-CoV-2 infections occurring in each state, we obtain a national IFR of 0.73% (0.68%–0.77%). Our findings are on par with the systematic meta-analysis of Meyerowitz-Katz and Merone [83], who estimated an IFR of 0.68% (0.53%–0.82%) for COVID-19 in 2020 based on 24 studies from a range of countries, as well as Eales et al. [82], who estimated the IFR in England in 2020 based on a series of nationally representative testing surveys at 0.67% (0.65%–0.70%). Similarly, Ward et al. [147] estimated the IFR in England in October 2020 at 0.74% (0.48%–1.40%). Figure 4 displays aggregate deaths, active viral prevalence, and cases in the US in 2020. While the posterior predictive distributions of deaths are well-calibrated at the state-level (as in Fig. 3, for example), when aggregated to the US as a whole they exhibit under-coverage. This is because we model the states independently and do not explicitly account for the “weekend effect”, i.e., consistent under-reporting of deaths on weekends which leads to highly correlated residuals across states on those days. As we evaluate and optimize NPI policies at the state-level, however, this does not pose an issue for our downstream analysis. Furthermore, we note that our model is able to account for under-reporting of deaths early in the pandemic. As evidenced by Fig. 4, our estimates of the underlying true number of COVID-19 deaths in the US ramp up before the observed death count does. This is because the likelihood on deaths accounts for and integrates over uncertainty in the accuracy of reporting.

### Estimating the effects of NPIs

We estimate the state-level effects of NPIs on the transmission rate $R_0$ using the robust log-linear hierarchical regression model detailed in Methods section. In Additional file 1: Section S.2, we compare our results to others in the literature.

We estimate a pooled baseline $R_0$ under no interventions of 2.3 (2.0–2.7). This is on par with the systematic review of Liu et al. [94], who report $R_0$ estimates for wild-type SARS-CoV-2 with median 2.79 and interquartile range 1.16. The pooled total effect of NPIs, which represents the effect of “full lockdown”, yields a reduction of $R_0$ by 50.0% (38.5%–59.3%) to 1.14 (1.03–1.28). By comparison, the pooled effects of deaths and removals incident in the prior week, when evaluated at the mean weekly rates of deaths and removals across states in 2020, yield a combined 11.2% (6.5%–16.7%) reduction in $R_0$ from voluntary social distancing and other protective measures due to fear of infection.

**Fig. 5:**
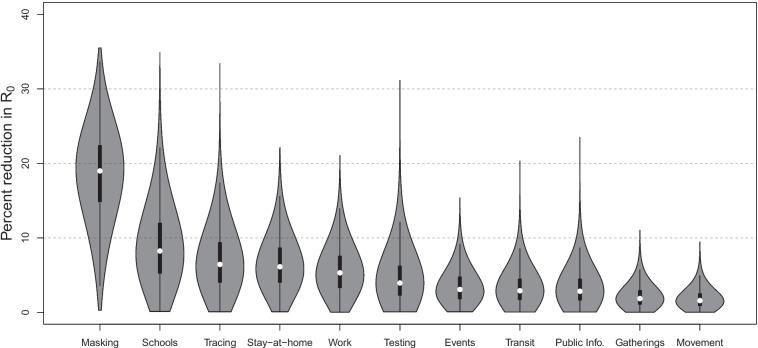
Posterior violin plots of global NPI effects in terms of percent reduction in $R_0$

Figure 5 shows the pooled effect of each NPI, quantified as a percent reduction in $R_0$. Mask mandates are the most effective intervention, reducing $R_0$ by 19.0% (6.1–28.5%). Second, school closure reduces the transmission rate by 8.2% (1.5–20.2%). Workplace closure reduced transmission by 5.3% (0.9–12.5%). Social distancing measures—defined here as the combination of stay-at-home orders, restrictions on gatherings, restrictions on internal movement, public information campaigns, public transit closures, and public event cancellations—yield a 18.9% (10.9–28.1%) reduction in $R_0$. Finally, testing and tracing policies reduce $R_0$ by 3.9% (0.6–12.3%) and 6.4% (1.1–16.0%), respectively.

### Evaluating the cost-effectiveness of NPI policies

Based on our estimates of the effects of NPIs on SARS-CoV-2 transmission, we evaluate their cost-effectiveness using costs associated with infections and NPIs from the economics and health literature as detailed in Methods section. We also derive optimal NPI strategies that minimize the gross health, economic, and social impacts of a policy. We compare our results to other relevant studies in Additional file 1: Section S.1.

**Fig. 6:**
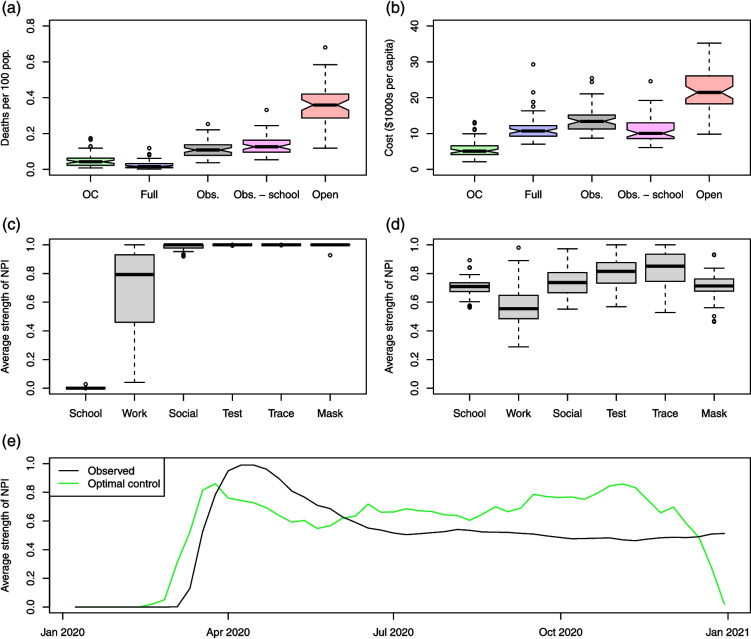
Top panels: Boxplots of the following quantities across states: (**a**) posterior median of deaths per 100 population under the OC, Full, Obs., Obs.−school, and Open policies; (**b**) expected total cost in thousands of USD2020 per capita incurred under each policy. Middle panels: the average strength over time of each NPI in the (**c**) OC and (**d**) Obs. strategies across states. Bottom panel: (**e**) the average strength of workplace closures across states in each week under the Obs. and OC policies

Figure 6 summarizes the results of our policy evaluation and optimization under the baseline scenario, which uses a cost function based on: the medium value of the cost of workplace closure; the lower value of the VSCD, which adjusts the VSL for the age pattern of COVID-19 deaths; and the lower cost of learning loss, which assumes distance learning is 90% as effective as in-person schooling. The relevant cost parameters are listed in Additional file 1: Table S2. In Additional file 1: Section S.1, we discuss sensitivity analysis of our results over a range of NPI regression model and cost function specifications. In terms of the cost function, we conduct sensitivity analysis with respect to the VSCD, cost of learning loss, and cost of workplace closure. Our qualitative findings about the structure of optimal policies are robust across scenarios, with the main quantitative distinction being the optimal strength of workplace closure. We also find that the relative ranking by cost of the policies considered can vary across cost function specifications.

Figure 6a, b displays boxplots of the COVID-19 death rate and per capita cost (including life costs) incurred under various policies across states. We consider the following five policy strategies: optimal control (OC), also referred to as the optimal strategy, which is the strategy optimizing the cost function; full lockdown (Full), which assumes that all NPIs are enforced at their strictest level for the entire year; observed (Obs.), the policy actually implemented; the observed policy minus school closure (Obs.−school); and the open policy (Open), which assumes no use of NPIs. Note that, as our cost function (4) averages over uncertainty in the parameters, we obtain a single optimal control strategy over time for each state rather than a posterior distribution of strategies.

Among these strategies, Full leads to the fewest deaths followed by OC, then Obs., then Obs.−school, and finally Open. Regarding overall costs, OC is the least expensive policy (as expected), followed by Obs.−school, Full, Obs., and finally Open. Figure 6c, d displays boxplots of the average strength of each NPI in the optimal control and observed strategies over the year across states. Strength here is defined relative to the maximum level of stringency seen over 2020. An average strength of 1 implies that the intervention is implemented in its strictest sense without interruption for the entire year, while an average strength of 0 implies that the intervention is never implemented. Figure 6e displays the average strength of workplace closure across states over time for the Obs. and OC strategies.

**Fig. 7:**
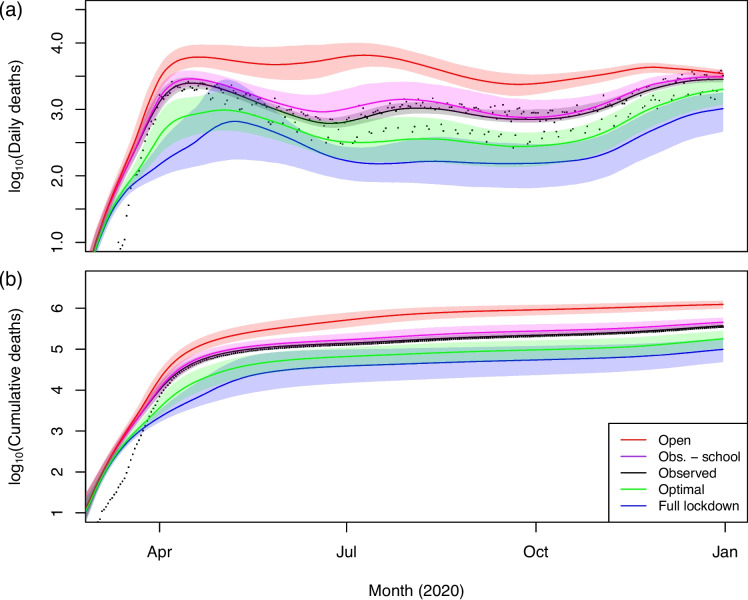
Posterior median and 95% credible intervals of (**a**) daily deaths and (**b**) cumulative deaths in the US under various strategies. Scatterpoints plot reported deaths.

Figure 7 displays the result of aggregating the state-level policy outcomes to the US as a whole. The Open strategy leads to the most deaths and is the most expensive by far, with its cost arising entirely from infections (including the impact of voluntary social distancing). We estimate that 1.24 (0.94–1.58) million COVID-19 deaths would have occurred in 2020 in the absence of public health interventions—lower than the 2.2 million projected by Ferguson et al. [26], who did not account for the endogenous social response to the virus. The burden of infections under Open yields an expected cost per US inhabitant of $21,000 in 2020 US dollars, which translates to a gross impact of $6.9 trillion—about 32% of US GDP in 2019 [125]. On the other hand, under Full, we would have observed only 98,900 (41,500–201,500) COVID-19 deaths and a cost to society of $11,900 per capita, which are surpassed by the 374,600 (337,300–445,200) deaths and $13,800 per capita ($4.6 trillion total, or 21% of 2019 GDP) lost under Obs. Note, however, that Full can become as expensive as Obs. and Open, or more so, if we assume a high cost of learning loss (see Additional file 1: Section S.1). The posterior median mortality rate under Full is 300 deaths per million, which is about 80% of the COVID-19 mortality rate observed in Canada in 2020 [140]. Obs.−school would have led to 456,700 (371,000–626,500) deaths, which is larger than the 374,600 (337,300–445,200) deaths under Obs. Nevertheless, the expected per capita cost to society of Obs.−school (including life costs), $10,300, is lower than that of Obs., $13,800. Our model estimates that school closures saved approximately 77,200 (12,300–236,000) lives, but at the expense of $2 trillion in lost learning, or $25.9 (8.4–156.4) million per COVID-19 death. However, the OC policy, which involves no school closures in 2020 beyond the usual 16 weeks of break, would have led to 179,700 (95,700–309,900) deaths, 195,500 (67,400–300,500) fewer than under Obs., at an expected cost to society of only $5,800 per capita. Most of the savings relative to Obs. stem from the cost of infections and school closures.

## Discussion

Combining data on COVID-19 cases, deaths, and policies within an epidemiological model, we estimated SARS-CoV-2 prevalence, transmission rates, effects of interventions, and costs associated to infections and NPIs in each US state in 2020. We used these estimates to determine the cost-effectiveness of various NPI policies and to derive optimal strategies.

We find that NPIs were effective in substantially reducing SARS-CoV-2 transmission, averting 860,000 (560,000–1,190,000) COVID-19 deaths in the US in 2020. Although school closures reduced transmission, their social impact in terms of student learning loss was too costly, depriving the nation of $2 trillion, conservatively, in future GDP. Moreover, this marginal trade-off between school closure and COVID-19 deaths was not inescapable: a combination of other measures would have been enough to maintain similar or lower mortality rates without incurring such profound learning loss. Optimal policies involve consistent implementation of mask mandates, public test availability, contact tracing, social distancing orders, and reactive workplace closures, with no closure of schools. Their use would have reduced the gross impact of the pandemic in the US in 2020 from $4.6 trillion to $1.9 trillion and, with high probability, saved over 100,000 lives.

Our estimates of SARS-CoV-2 infections incident in 2020 leverage prior work based on random sample testing [55]—a putatively unbiased measure of viral prevalence—and, as noted in Results section, produce a reasonable national IFR estimate similar to others reported in the literature. Nevertheless, we note that our findings concerning policy evaluation and optimization are robust to sensible variations in the IFR. This is because the costs of infections are dominated by COVID-19 deaths, which are identified from the clinical data we use here and, therefore, are outputs of our model not substantially affected by the IFR parameter (which only varies the estimated number of infections incident per death).

Our estimate of the total percent reduction in $R_0$ due to NPIs, 50.0% (38.5%–59.3%), is more conservative than others reported in the literature. With a pooled baseline $R_0$ under no interventions of 2.3 (2.0–2.7), neither NPIs nor the endogenous behavioral response in isolation would be likely to control transmission ($R_0 < 1$) at the start of the outbreak for a population with these parameter values. However, the combination of stringent NPIs, voluntary protective measures, and acquired immunity would be enough to effectively suppress viral spread, at least in the absence of exogenous shocks.

Flaxman et al. [8], Brauner et al. [9], and Banholzer et al. [13], respectively, find 81% (75–87%), 77% (67–85%), and 67% (64–71%) reductions in transmission in the initial spring 2020 wave. Studying the second wave, Sharma et al. [10] report a combined NPI effect of 66% (61–69%). We note that none of these studies control for confounding (e.g., endogenous social distancing), which may account for the discrepancy with our estimates. Indeed, when we add the effect of deaths and removals incident in the prior week to that of NPIs, the combined reduction in $R_0$ approaches these higher estimates. Another possible explanation is the context: we study the US whereas Flaxman et al. [8], Brauner et al. [9], Sharma et al. [13], and Banholzer et al. [10] focus primarily on European countries, which may have implemented stricter NPIs or practiced greater adherence to restrictions, and which exhibited higher $R_0$ values (3.3–3.8), perhaps due to earlier introduction of the virus to European countries or higher levels of social mixing on average.

The structure of optimal NPI strategies is remarkably simple and consistent across US states, with minimal spatial or temporal variation in the optimal strength of social distancing measures, testing, tracing, masking, and school closure. However, we find that workplace closure more closely straddles the cost-effectiveness threshold, which was also noted by Barrot et al. [37]. Rather than continuously implementing workplace closures at full strength or not at all, OC policies involve ramping them up to combat new waves of infections, with implementation peaking in response to the spring, summer, and fall waves of 2020 (Fig. 6e). Relative to OC, the average strength of workplace closure in Obs. ramped up and peaked later in spring and then remained constant at a (lower) moderate level throughout the year. As a result, the OC policy exhibits a flatter prevalence curve, with summer and fall waves that are muted relative to what was observed (see Fig. 7 and Additional file 1: Fig. S1).

We note that, for practical purposes and due to lack of available data, our model of SARS-CoV-2 transmission does not account for a number of complexities. In future work, more detailed modeling strategies—such as agent-based or more fine-grained compartmental models—coupled with higher resolution data may prove useful.

We do not explicitly account for the age structure of a state’s population and its infections, although these are reflected in the state-specific IFR and NPI effect estimates used in our model. (The latter can account, for example, for differential effects of school closure across states owing to younger or older populations.) As such, our reported effects of NPIs on transmission and costs associated to infections and NPIs should be interpreted as aggregate measures. Similarly, as we model aggregate rather than individual data, our conclusions about the effectiveness of NPIs do not necessarily indicate where transmission events were concentrated. For example, we find that school closures yielded greater transmission reductions than workplace closures, but this does not imply that more transmission took place in schools than in workplaces.

We do not model state-level hospital capacity and potential excess costs or deaths arising from an overwhelmed medical system. In principle, doing so would serve to increase the cost associated with infections. Estimates of the IFR in England, which experienced COVID-19 death rates similar to the US in 2020 [140], are fairly constant over the year [82], suggesting that COVID-19 mortality outcomes—the dominant term in the cost of infection—were not highly sensitive to fluctuations in the burden on hospitals. Similarly, in an analysis of the first pandemic wave in France, Glemain et al. [148] found no evidence for hospital overload explaining an increased risk of death for infections in higher incidence areas; rather, the inflated IFR is likely due to higher ages among infected individuals in hotspots. We note, however, that these observed death rates are conditional on the NPI policies implemented, which served to limit the burden on hospitals. It is possible that, in the absence of NPIs, the IFR would increase due to hospital overload.

In a similar vein, we note that our representation of voluntary social distancing within the model is a simplification. For example, we assume that the total cost of voluntary social distancing (associated to the fear of infection), which is proportional to the number of people socially distancing, is linear in the number of incident infections. In reality, voluntary social distancing may only commence once an outbreak crosses a threshold. Furthermore, when government restrictions are already in place, the marginal effect of voluntary social distancing (both on the economy and on viral transmission) may dwindle.

We do not account for mental health costs arising from lockdowns and from the fear of infection, which may be substantial [23, 149]. While there is some recent work estimating the causal effects of stay-at-home orders and school closure on mental health outcomes [150], the effects of other relevant exposures, including workplace closure, other NPIs, and the pandemic itself, have not been ascertained. Considering other indirect costs of school closure, we note that they can cause significant healthcare worker absenteeism, potentially negating some or all of the mortality benefits from school-closure-related reductions in SARS-CoV-2 transmission [58, 144]. Nevertheless, we do not account for health impacts related to healthcare personnel absenteeism as quantifying their cost is challenging. For these and other reasons, which we discuss in Methods section, we believe that our accounting of the costs of school closure is conservative.

Given the highly correlated implementation of NPIs and that we are already accounting for spatial heterogeneity of their effects in our hierarchical model, we do not also model temporal variation in NPI effects (e.g., arising from “pandemic fatigue”), which has been documented in some studies [10, 151, 152], although with mixed evidence, as it would be difficult to identify from the data. While Ge et al. [152] find that the overall effect of NPIs in Europe increased over time in 2020, Petherick et al. [151] observe increasing use of masks but declining adherence to physical distancing measures across countries over the year. Sharma et al. [10] estimate a reduced effect of school closure in the second wave in Europe. In relation to our results, increasing the efficacy of masking policies and decreasing the efficacy of school closures would not reverse the conclusion that mask mandates are highly cost-effective whereas school closures are not. However, substantial decreases in the efficacy of workplace closure and social distancing measures could impact their cost-effectiveness.

Similarly, we do not include interaction effects in our model, although we note that NPIs have multiplicative effects on the transmission rate in our log-linear regression model. This can be considered an interaction effect in a certain sense: multiple NPIs in combination are assumed to have diminishing marginal returns, i.e., the combination of two NPIs that each reduce $R_0$ by 50% would yield a 75% rather than 100% reduction in transmission.

Finally, as we model viral spread in the US states independently of each other, we do not account for spillover effects of intervention policies between states, which may play a role in overall trends in transmission [153]. (Though we can estimate the effect of a state’s restrictions on travel within the state itself, as our model includes data tracking restrictions on internal movement.) We would also like to emphasize that our model estimates the *total effect* of the *stated NPI policy* on the transmission rate (which is identified, given our modeling assumptions). Therefore, for example, if individuals respond to public event cancellations by refraining from holding informal gatherings (an endogenous response beyond the behavioral changes required by the policy), which in turn reduces the transmission rate, this reduction in $R_0$ would be attributed to the effect of public event cancellation policies in our model, rather than the effect of public events themselves or the *indirect effect* of public event policy on transmission rates through its impact on realized informal gatherings, which have not been measured. This is appropriate, as our analysis operates from the perspective of a state-level decision-maker concerned primarily with determining public policy.

While the use of NPIs involves health, economic, and social trade-offs, it is not a zero-sum game. As others have noted [24], appropriately implemented restrictions can simultaneously limit deaths and the aggregate costs to society incurred during a pandemic. Our methodology enables us to derive optimal NPI strategies, which consist of timely, enduring, and stringent use of testing, tracing, and masking policies, social distancing measures, and reactive workplace closure, with no closure of schools.

This last finding is salient as schools were closed for extended periods in the US and in many other countries throughout the pandemic. Growing evidence suggests that the impacts on school children are substantial and long-term [3, 154]. As we show in Additional file 1: Section S.1, our conclusion that school closures were not cost-effective is robust to plausible variation in the cost function. Furthermore, given that we estimate school closure to be one of the interventions most effective in reducing transmission, our results would not be easily reversed based on different modeling assumptions. If we have overestimated the effect of school closure on transmission reduction for most of 2020, getting closer to the truth would only strengthen our findings. Relative to the first COVID-19 wave in Europe [9], Sharma et al. [10] found that school closure was substantially less effective in reducing transmission, hypothesizing that the effect attenuated from the first to the second wave because many schools in Europe reopened without substantial increases in transmission. Some have argued that, with adequate health protocols in place, US schools that remained closed through the 2020–2021 academic year could have resumed in-person learning safely [154, 155].

Fahle et al. [63] provide a comprehensive review and analysis of the data on school closures, remote learning, and learning loss during the pandemic in the US. In line with Betthäuser et al. [5], they find consistent learning loss of at least 0.35 years (as measured by grade school math scores), with larger losses in school districts that spent more time in remote or hybrid learning and in poorer communities, which were more likely to remain remote longer. A more detailed assessment of the links between remote learning policies, access to digital infrastructure, and learning and health outcomes may be warranted. Accounting for heterogeneity in access to quality remote learning is important when making decisions about when and where to close schools, and where to distribute resources to address learning lost during infectious disease outbreaks. Additionally, such a study could determine the cost-effectiveness of hybrid learning options relative to fully remote or in-person instruction. As the OxCGRT dataset does not track hybrid learning policies specifically, we are unable to conclude whether hybrid instruction–a combination of in-person and remote schooling–is more or less cost-effective than in-person schooling. Fahle et al. [63] found that, as with fully remote learning, US school districts that spent more time in hybrid instruction experienced greater learning losses relative to districts with more in-person schooling. Their results show that any time spent not in-person is detrimental to learning. However, this still leaves open the question of whether hybrid instruction is less cost-effective than in-person schooling. On the other hand, given our finding that in-person schooling is cost-effective relative to fully remote learning, we expect that hybrid instruction is also cost-effective relative to remote learning, as it provides some in-person instruction. While we find that extended school closure is not cost-effective, a relevant (and potentially cost-effective) counterfactual would have been a reactive school closure of limited duration at the beginning of the pandemic (i.e., spring of 2020) compensated by an extended school year stretching into the summer. Such a strategy would likely have incurred less student learning loss by merely shifting summer break toward spring to allow for an urgent response to the initial outbreak.

In our review of the literature, we found only two cost-benefit analyses of school closure that account for learning loss [33, 50]. Studying pandemic flu, Xue et al. [50] find that school closures are not cost-effective for mild strains (such as the 2009 H1N1 virus), but they generate net benefits in the context of more severe pandemics, such as the 1918 Spanish flu. Similarly, studying historical outbreaks of influenza, gastroenteritis, and chickenpox in France, Adda [33] determines that school closures were not cost-effective, but that they would become beneficial for slightly more lethal epidemics. Notably, Xue et al. [50] model school closure in isolation, i.e., they do not consider the availability of other interventions, and Adda [33] considers only school and public transport closure. Their results concur with a number of other studies (not accounting for learning loss) finding that extended school closures are cost-effective for severe pandemics [39, 43–45, 60]. To the contrary, we conclude that COVID-19 school closures were not cost-effective.

## Conclusions

We have developed a statistical decision framework in order to conduct a cost-effectiveness analysis of NPIs in the US during COVID-19. We find that NPIs were effective in substantially reducing SARS-CoV-2 transmission. Cost-effective policies that minimize aggregate costs to society involve consistent implementation of mask mandates, public test availability, contact tracing, social distancing orders, and reactive workplace closures, with no closure of schools beyond the usual 16 weeks of break per year. More broadly, our methodological framework can be used to evaluate the cost-effectiveness of public health interventions against infectious disease and to understand the relationships between interventions, the behavioral response, and disease transmission.

While our study focuses on COVID-19 in the US prior to the arrival of vaccines, our findings about the cost-effectiveness and optimal structure of NPI policies shed light on NPI implementation in other settings. Masking, testing, and tracing are relatively cheap and likely to remain cost-effective universally: for severe and relatively mild pandemics; in lower resource settings; and after effective pharmaceutical interventions become available. After the arrival of vaccines and antiviral treatments, workplace closures and social distancing measures should be enacted more sparingly. Although school closures were not cost-effective, evidence suggests that distance learning helped to mitigate learning loss. Consequently, extended school closures are likely to be relatively more costly in low- and middle-income countries with younger populations and less capacity to provide effective education remotely [3]. Likewise, with fewer opportunities for remote work and less online economic activity, workplace closures, stay-at-home orders, and other social distancing measures may be more costly in these countries [156]. For less virulent diseases with a similar age pattern of death, extended school closures are unlikely to be justifiable, and extended workplace closures and social distancing measures should be mandated with care. If possible, more targeted interventions should be used [29].

## Supplementary information

Additional file 1. Supplement containing: further results and discussion for the cost-effectiveness of interventions, including sensitivity analysis of optimal control strategies and discussion of the use of incremental cost-effectiveness ratios (ICERs) in infectious disease (Section S.1); further results and discussion for the NPI regression model (Section S.2); epidemiological and economic parameter Tables S1–S3 (Section S.3); and plots of state-specific epidemiological model estimates (Section S.4).

## Data Availability

All data used are publicly available. We obtained US state-level daily counts of confirmed COVID-19 cases and deaths in 2020 from the Johns Hopkins COVID-19 Data Repository [79]. We obtained daily state-level NPI policies from OxCGRT [80]. We obtained daily state-level counts of SARS-CoV-2 PCR tests administered from the COVID-19 Tracking Project [81]. All data and code to reproduce our analysis are available at the GitHub repository and archived in Zenodo [78].

## Abbreviations

BHM: Bayesian hierarchical model
COVID-19: Coronavirus disease 2019
DAG: Directed acyclic graph
DALY: Disability-adjusted life-year
Full: Full lockdown NPI policy
GDP: Gross domestic product
ICER: Incremental cost-effectiveness ratio
IFR: Infection fatality rate
LOO-CV: Leave-one-out cross validation
MAP: *Maximum a posteriori*
MCMC: Markov chain Monte Carlo
NPI: Non-pharmaceutical intervention
Obs.: Observed NPI policy
Obs.–school: Observed NPI policy minus school closures
OC: Optimal control NPI policy
Open: Fully open NPI policy
OxCGRT: Oxford COVID-19 government response tracker
PCR: Polymerase chain reaction
QALY: Quality-adjusted life-year
SARS-CoV-2: Severe acute respiratory syndrome coronavirus 2
SEIRD: Susceptible-Exposed-Infectious-Removed-Deceased
SIR: Susceptible-Infectious-Removed
US: United States
USD: US dollars
VSCD: Value of a statistical COVID-19 death
VSL: Value of a statistical life
