# Author Correction: Optimal pandemic control strategies and cost-effectiveness of COVID-19 non-pharmaceutical interventions in the United States

**DOI:** 10.1186/s44263-025-00220-3

**Published:** 2025-11-04

**Authors:** Nicholas J. Irons, Adrian E. Raftery

**Affiliations:** 1 Department of Statistics, Leverhulme Centre for Demographic Science, and Pandemic Sciences Institute, University of Oxford, Oxford, UK; 2 Departments of Statistics and Sociology, University of Washington, Seattle, USA

**Author Correction: BMC Glob. Public Health 3, 76 (2025)**

**https://doi.org/10.1186/s44263-025–00189-z**

Following publication of the original article an error was reported in the Results section.

**Table:** 

Original version	Corrected version	Description of error
*In the section “Results: Estimating Transmission Dynamics”* Weighting the posterior state-level IFR estimates by the proportion of 2020 US COVID-19 deaths occurring in each state, we obtain a national IFR of 0.78% (0.74%–0.82%)	Weighting the posterior state-level IFR estimates by the proportion of 2020 US SARS-CoV-2 infections occurring in each state, we obtain a national IFR of 0.73% (0.68%–0.77%)	In the original version, the national infection fatality rate (IFR) was miscalculated, incorrectly using deaths instead of infections to write the national IFR as a weighted average of state-level IFR estimates. Indeed, we have $$\begin{array}{cc}{{IFR}}^{(USA)}& =\frac{deaths^{(USA)}}{{{infections}}^{(USA)}}\\ & =\frac{{\sum }_{s\in USA}{{deaths}}^{(s)}}{{{infections}}^{(USA)}}\\ & =\frac{{\sum }_{s\in USA}\frac{{{deaths}}^{(s)}}{{{infections}}^{(s)}}\cdot {{infections}}^{(s)}}{{{infections}}^{(USA)}}\\ & =\frac{{\sum }_{s\in USA}{{IFR}}^{(s)}\cdot {{infections}}^{(s)}}{{{infections}}^{(USA)}}\\ & =\sum_{s\in USA}{{IFR}}^{(s)}\cdot \frac{{{infections}}^{(s)}}{{{infections}}^{(USA)}}\end{array}$$

The original article has been updated.

