# Supplementary material for: Optimal pandemic control strategies and cost-effectiveness of COVID-19 non-pharmaceutical interventions in the United States
Source: BMC Glob Public Health. 2025 Sep 12;3:76. doi: 10.1186/s44263-025-00189-z (PMC12427114; doi:10.1186/s44263-025-00189-z)
Supplement: Supplementary file 1 — Additional file 1. Supplement containing: further results and discussion for the cost-effectiveness of interventions, including sensitivity analysis of optimal control strategies and discussion of the use of incremental cost-effectiveness ratios (ICERs) in infectious disease (Section S.1); further results and discussion for the NPI regression model (Section S.2); epidemiological and economic parameter Tables S1–S3 (Section S.3); and plots of state-specific epidemiological model estimates (Section S.4). [file 44263_2025_189_MOESM1_ESM.pdf]

Additional file 1 for "Optimal pandemic control strategies and cost-effectiveness of COVID-19 non-pharmaceutical interventions in the United States."

Nicholas J. Irons*, Adrian E. Raftery†

This supplement contains: further results and discussion for the cost-effectiveness of interventions, including sensitivity analysis of optimal control strategies and discussion of the use of ICERs in infectious disease (Section S.1); further results and discussion for the NPI regression model (Section S.2); epidemiological and economic parameter Tables S1–S3 (Section S.3); and plots of state-specific epidemiological model estimates (Section S.4).

S.1 Evaluating and optimizing costs

S.1.1 Baseline scenario

Dynamic workplace closure strategies. Figure S1 plots the average strength of workplace closure across states over time and the resulting viral prevalence for the OC, Obs., and Obs.−school strategies. Generally, the OC strategy involves ramping up workplace closure to combat new waves of infections, with implementation peaking in response to the spring, summer, and fall waves of 2020. On the other hand, relative to OC, the average strength of workplace closure in Obs. ramped up and peaked later in spring and

*Department of Statistics, Leverhulme Centre for Demographic Science, and Pandemic Sciences Institute, University of Oxford, Oxford, UK. Corresponding author. Email: nicholas.irons@stats.ox.ac.uk.

†Departments of Statistics and Sociology, University of Washington, Seattle, USA.

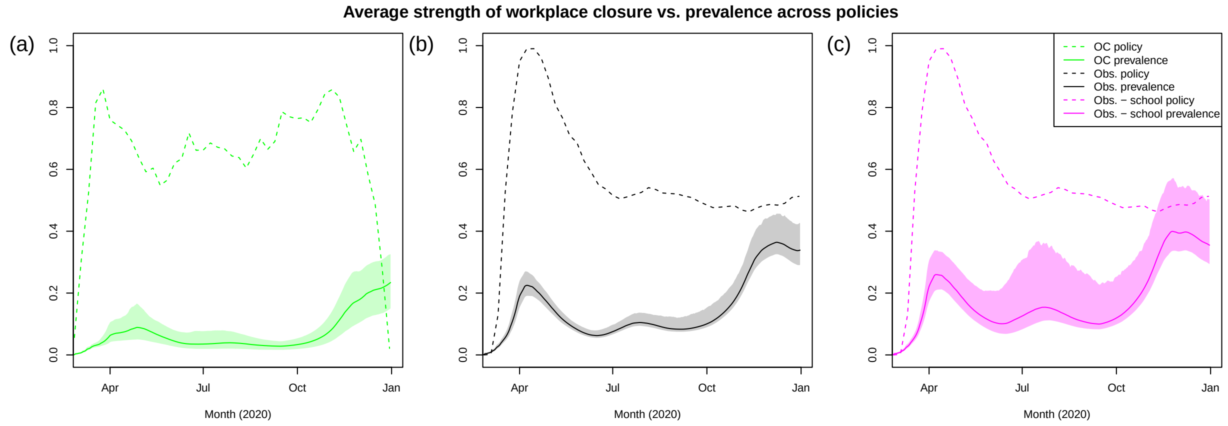

Figure S1: Average strength of workplace closure across states over time (dashed line) and the resulting viral prevalence (posterior median with 90% credible interval) for the (a) OC, (b) Obs., and (c) Obs.−school strategies

then remained constant at a (lower) moderate level throughout the year. As a result, the OC policy exhibits a flatter prevalence curve, with summer and fall waves that are muted relative to Obs. and Obs. - school.

Comparison to the literature. We can compare our estimates of the gross impacts of COVID-19 and NPIs to others in the literature. Cutler & Summers [1] and Bruns & Teran [2] project the total cost of the pandemic in the US over its full duration at $16 trillion, which is about 3.5 times our estimate of $4.6 trillion in losses observed during 2020. Flaxman *et al.* [3] estimated that NPIs averted 3.1 (2.8–3.5) million deaths up to May 2020 in 11 countries totaling 375 million population. Ferguson *et al.* [4] predicted that NPIs would prevent 1.1 million deaths in the US over the course of the pandemic. Greenstone & Nigam [5] projected that moderate social distancing would save 1.7 million lives in the US by October 1, with mortality benefits of $8 trillion, or $24,000 per capita, based on the same VSCD used in our baseline scenario. Highlighting the inter-generational transfer of wealth stemming from the implementation of social distancing measures, they note that the vast majority of the monetized benefits of social distancing accrue to people age 50 or older. More conservatively, Eichenbaum *et al.* [6] find that containment policies, if implemented optimally, would save about half a million lives in the US based on low values of key epidemiological parameters—specifically, they use an IFR of 0.5% and an R_0 of 1.45 for their

baseline model. Thunström *et al.* [7] estimated that social distancing in the US would save 1.24 million lives at a cost of \$7.2 trillion in lost GDP, which implies that social distancing measures would yield net losses for any VSCD below $7.2/1.24 \approx 5.8$ million dollars. To the contrary, we find that NPIs (and social distancing measures in particular) are cost-effective for a VSCD of \$4.5 million. Assuming a vaccine arrives (stochastically) after a year to end the pandemic, Farboodi *et al.* [8] estimate the per capita cost of the optimal policy at \$8,100, comparable to our \$5,781. They find that the *laissez-faire* equilibrium (i.e., in the absence of government intervention) would only incur a cost of \$12,700 per person, as compared to our \$20,978. Undertaking a cost-benefit analysis of confinement policies targeted toward mitigation and (strict) suppression, Gollier [9] finds that both strategies incur a total cost—combining economic and life costs—equating to 15% of annual GDP, or about \$10,000 per capita, which is comparable to our estimates of the total costs of the various containment strategies in Results. Jones *et al.* [10] estimate that, under an optimal social distancing policy, GDP declines by 12% and 0.17% of the population (about 560,000) die from COVID-19 in the first 26 weeks of the pandemic. In a simulation study, Keogh-Brown *et al.* [11] estimate that containment strategies in the UK to suppress COVID-19 through the end of 2020 would incur health costs of 1.7% of GDP and economic costs of 29.2% of GDP, with 7.3 percentage points coming from workplace absenteeism of parents affected by school closure and 21.9 percentage points from business closure. Their total cost is comparable to our estimate for the US in 2020 (i.e., under Obs.), which is 21% of GDP. However, relative to the results of Keogh-Brown *et al.* [11], we find that health impacts are a much larger portion of the total, costs related to business closure are much smaller, and costs related to school closure are somewhat larger.

S.1.2 Sensitivity analysis of optimal control strategies

Figures S2–S7 display the results of our sensitivity analysis across regression models (i)–(iii) and cost function specifications. Overall, our qualitative findings about the structure of OC policies are robust across scenarios, with the main quantitative distinction being the

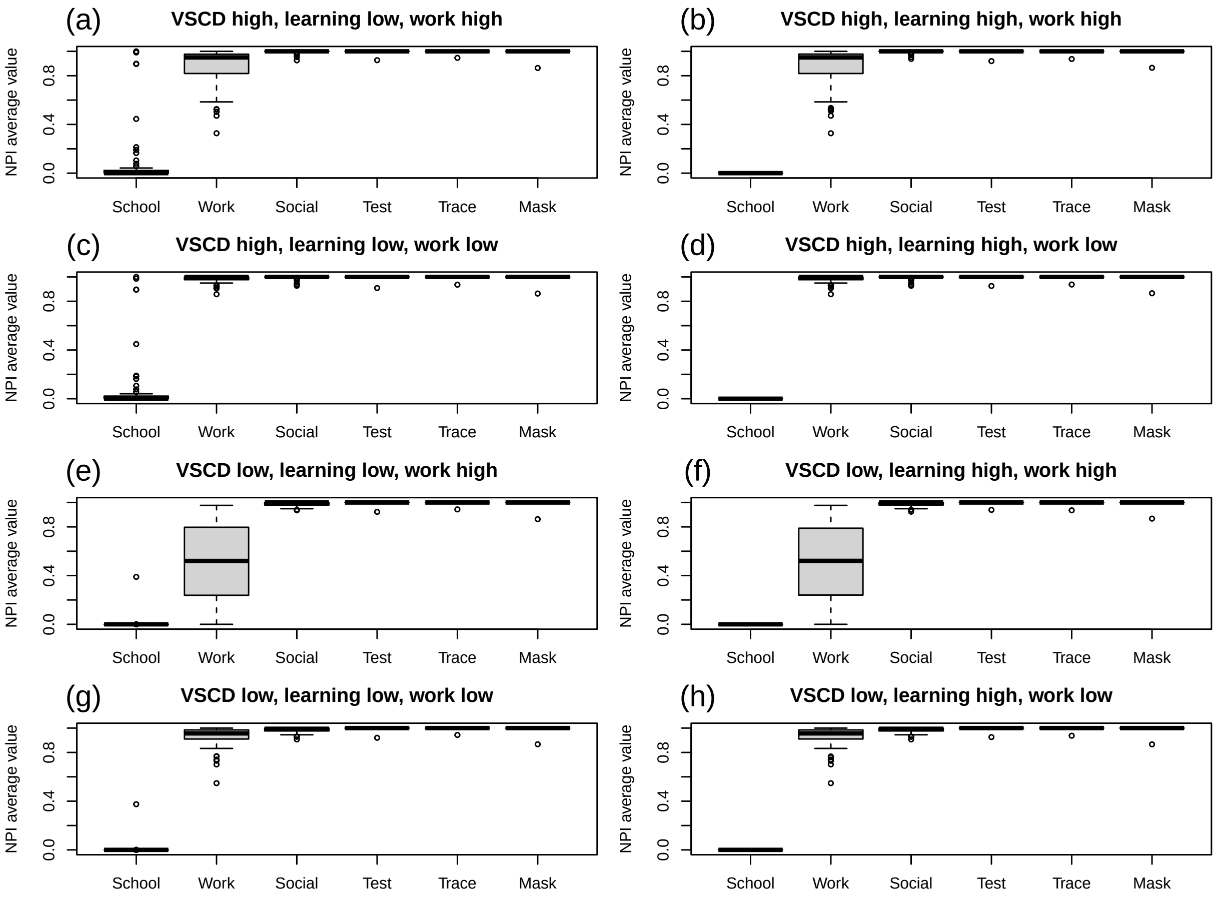

Figure S2: Sensitivity analysis for the optimal control results under regression model (i). Boxplots of the average value of the optimal NPI policy over the year in each state.

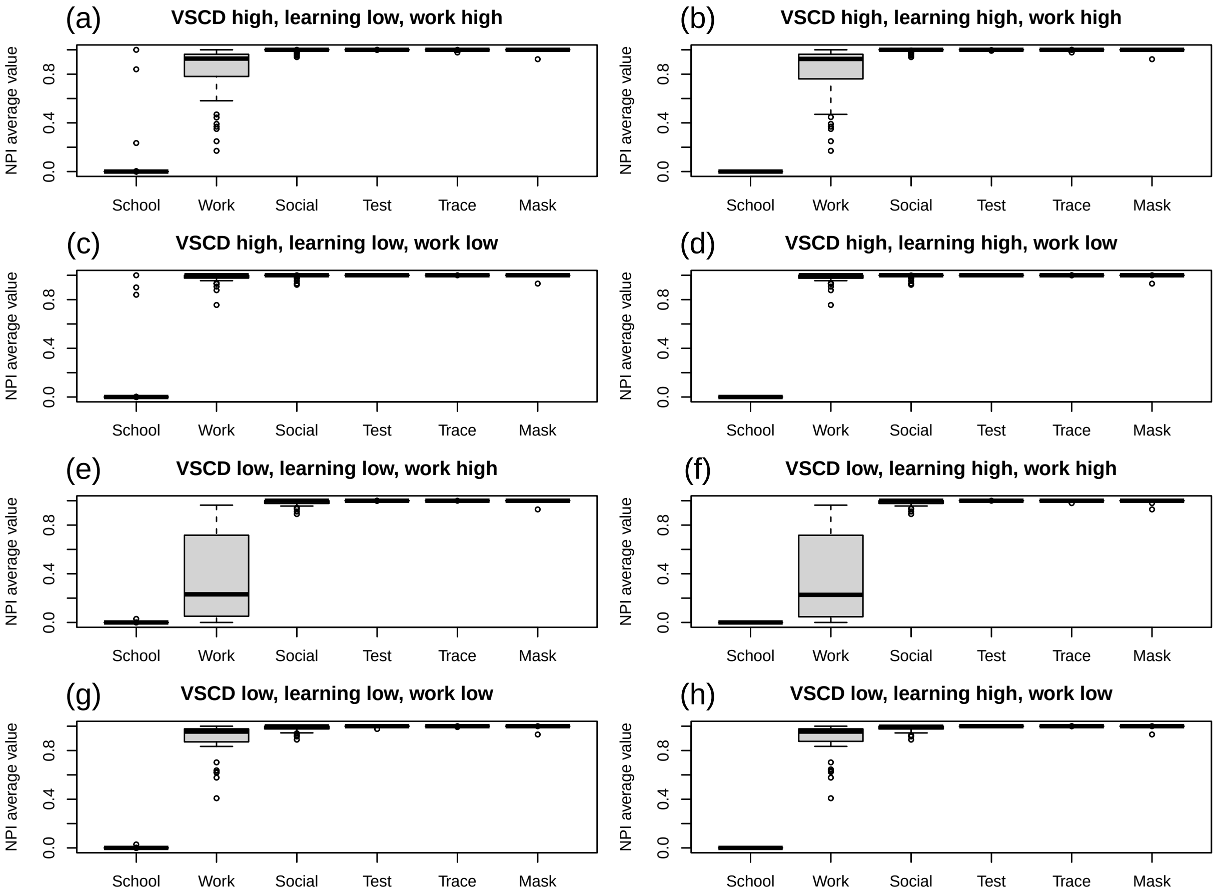

Figure S3: Sensitivity analysis for the optimal control results under regression model (ii). Boxplots of the average value of the optimal NPI policy over the year in each state.

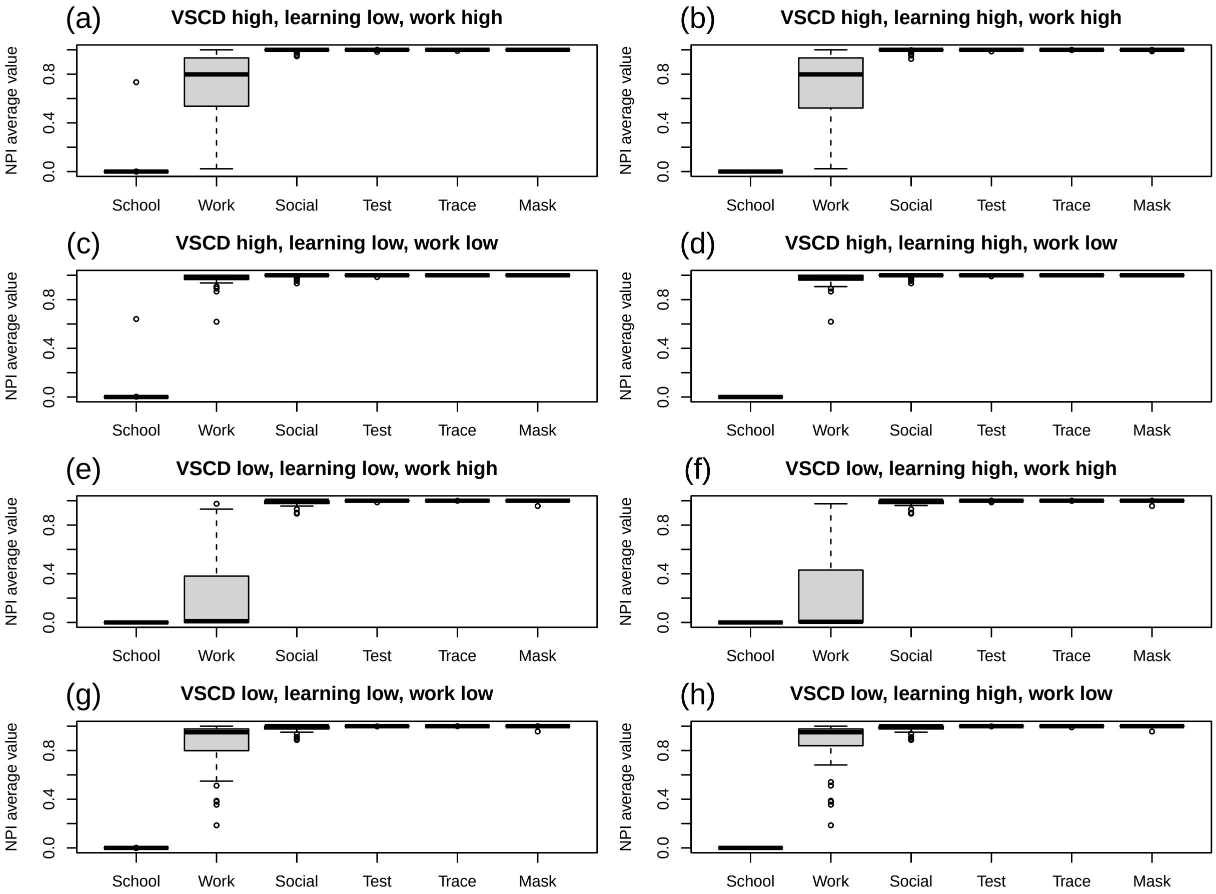

Figure S4: Sensitivity analysis for the optimal control results under regression model (iii). Boxplots of the average value of the optimal NPI policy over the year in each state.

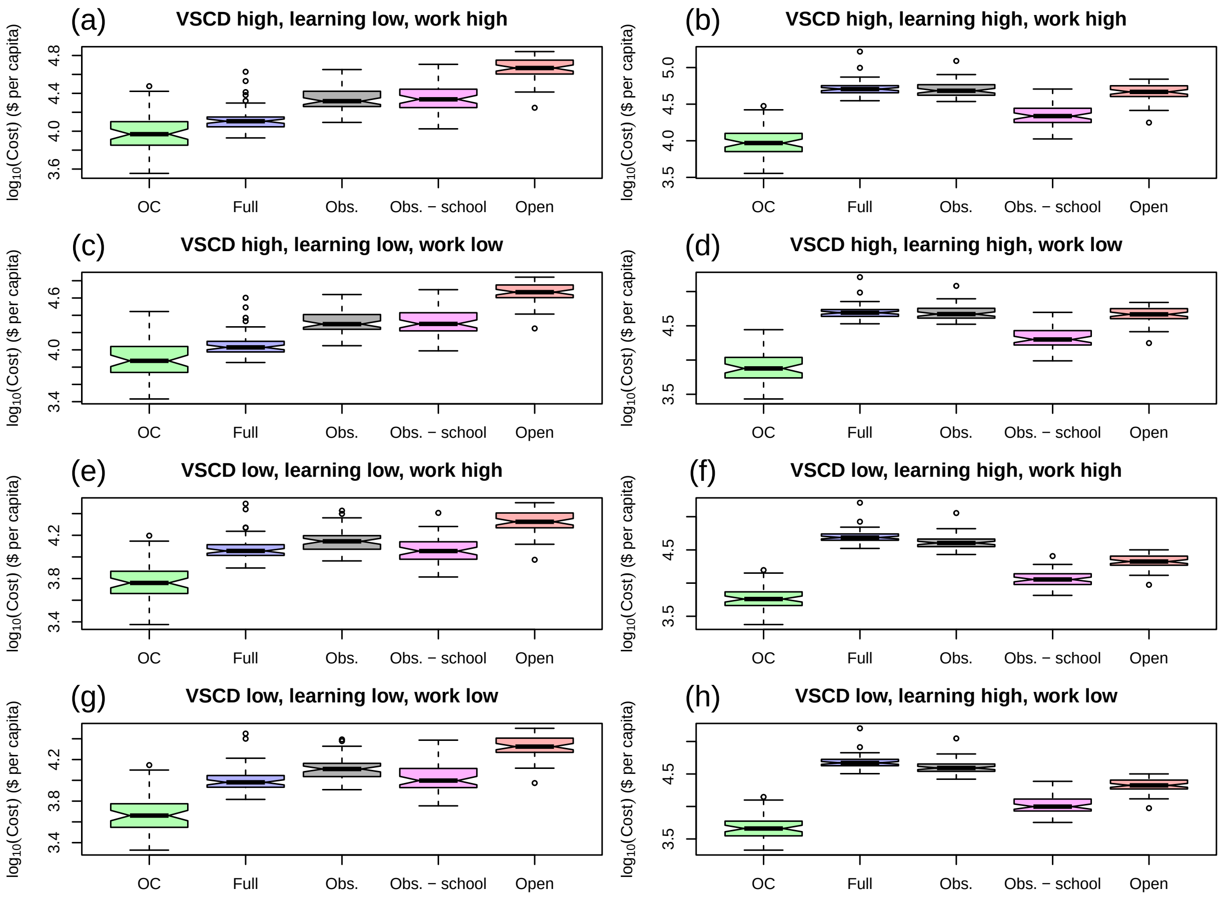

Figure S5: Sensitivity analysis for the costs of various policies under regression model (i). Boxplots of the log-scale total cost in USD2020 per capita incurred by the OC, Full, Obs., Obs.−school, and Open policies across states.

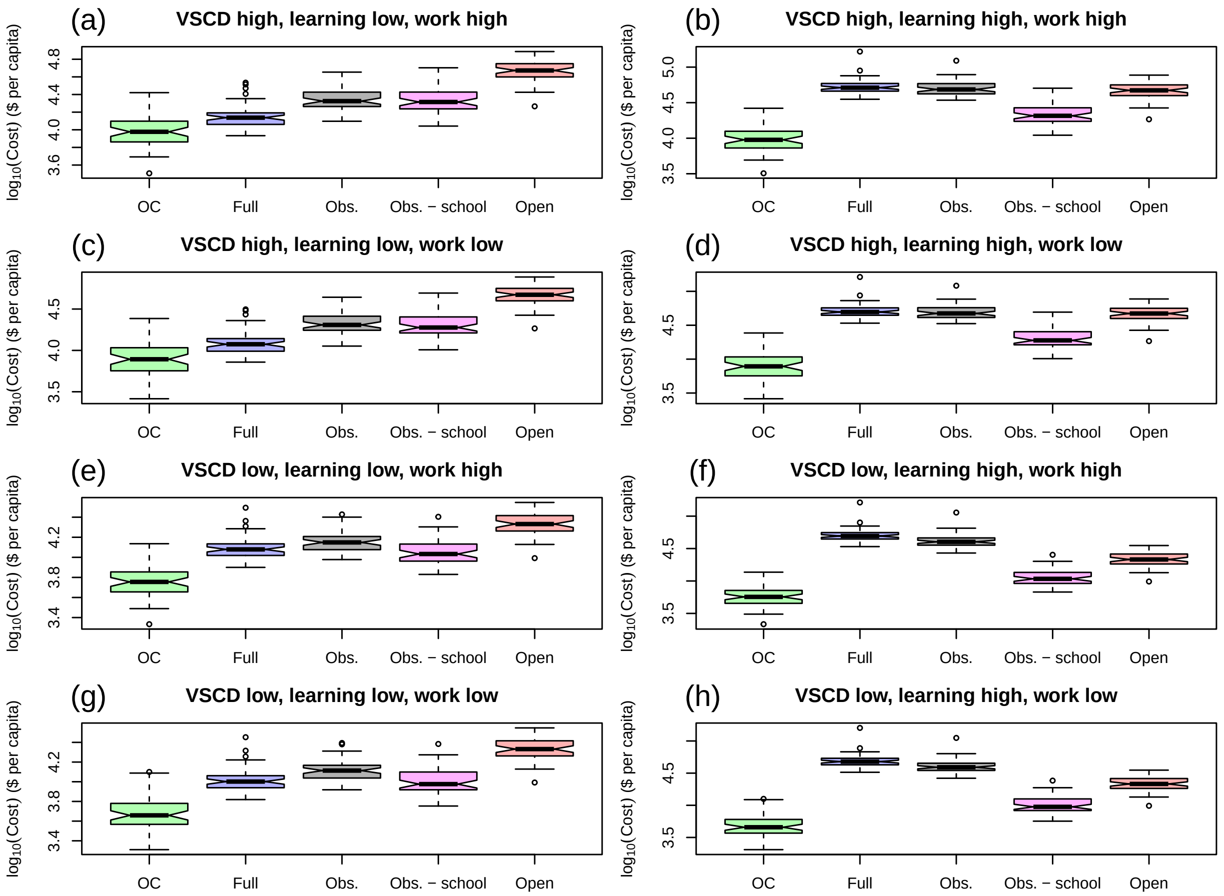

Figure S6: Sensitivity analysis for the costs of various policies under regression model (ii). Boxplots of the log-scale total cost in USD2020 per capita incurred by the OC, Full, Obs., Obs.−school, and Open policies across states.

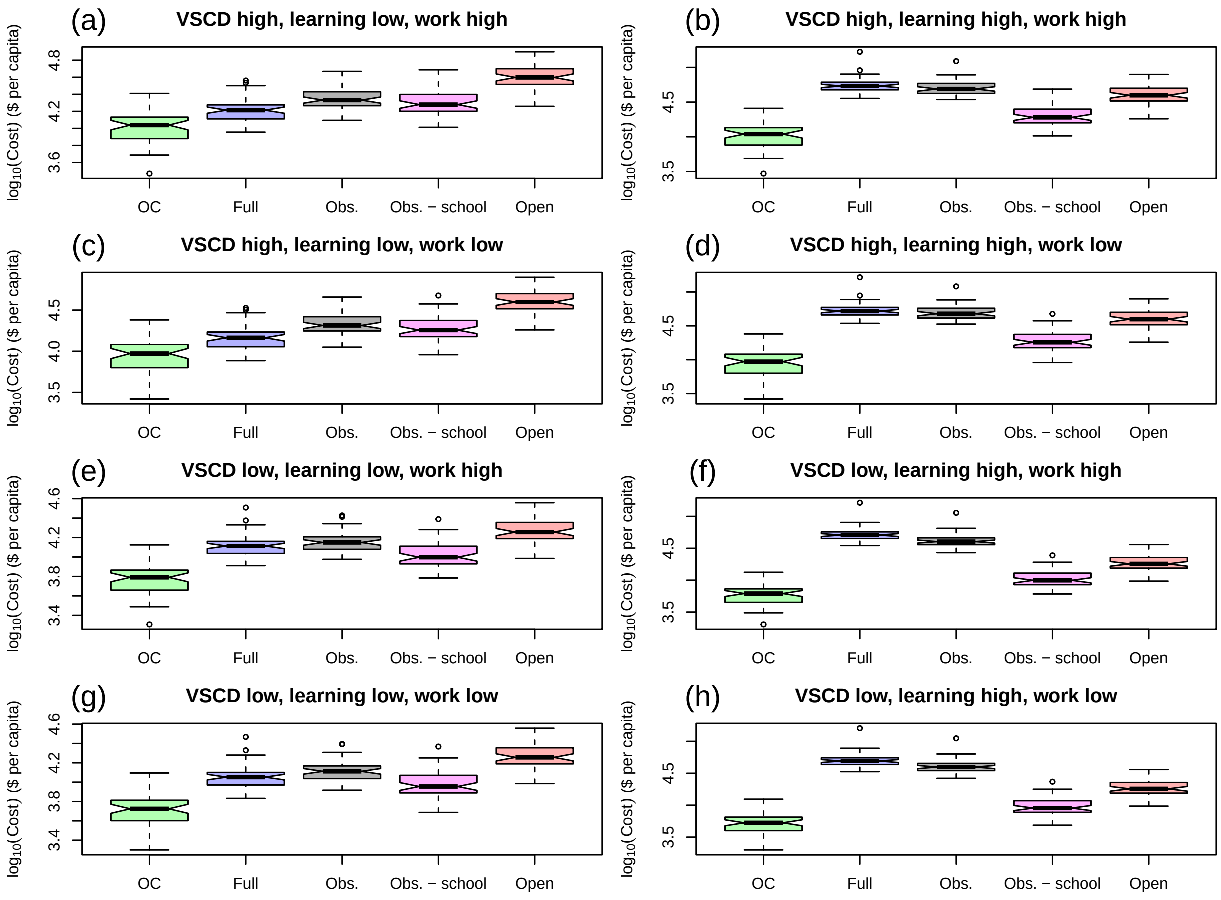

Figure S7: Sensitivity analysis for the costs of various policies under regression model (iii). Boxplots of the log-scale total cost in USD2020 per capita incurred by the OC, Full, Obs., Obs.−school, and Open policies across states.

optimal strength of workplace closure. We also find that the relative ranking by cost of the policies considered can vary across cost function specifications.

We vary the VSCD, the cost of learning loss, and the cost of workplace closures across plausible ranges, which are given in Table S2. As noted in Methods, we do not vary the cost of social distancing measures as we are primarily interested in assessing the robustness of the OC strategy and the relative costs of various policies rather than variation in the total cost incurred by each policy. We also omit sensitivity analysis for the costs of testing, tracing, and masking as they are highly cost-effective interventions under any reasonable variation in their costs. Indeed, these measures are all at least somewhat effective in reducing transmission and extremely cheap compared to infections, school and workplace closures, and social distancing mandates.

Figures S2–S4 exhibit boxplots of the average value of each NPI in the OC strategy across states for models (i)–(iii), respectively. We see that, barring a few outliers in some scenarios, school closures are never implemented in the OC policy. This is the case even when we assume both a high VSCD equal to the VSL ($10.63 million USD2020)—which does not adjust the VSCD for the age profile of COVID-19 mortality—and a low cost of learning loss (9% of GDP per 0.33 years)—which assumes that distance learning is 90% as effective as in-person schooling—which are shown in the upper left panels. Across scenarios, workplace closures are implemented fairly consistently, but their optimal duration decreases as the cost of workplace closure increases or the VSCD decreases. In particular, in Figure S4 (model (iii)), we see that the median optimal strength of workplace closure across states is 0 when we use a low VSCD and high cost of workplace closure. These conclusions are comparable to the results of Barrot *et al.* [12], who find that the cost-effectiveness of business closures (i.e., whether they produce a net benefit or loss) is sensitive to modeling assumptions and particularly the assumed value of a life-year.

Figures S5–S7 show boxplots of the costs of various policies across states. Notably, when the cost of learning loss is high (right column, subplots (b), (d), (f), and (h)), the total cost of Obs.−school is substantially lower than Obs. and Full. If we further assume

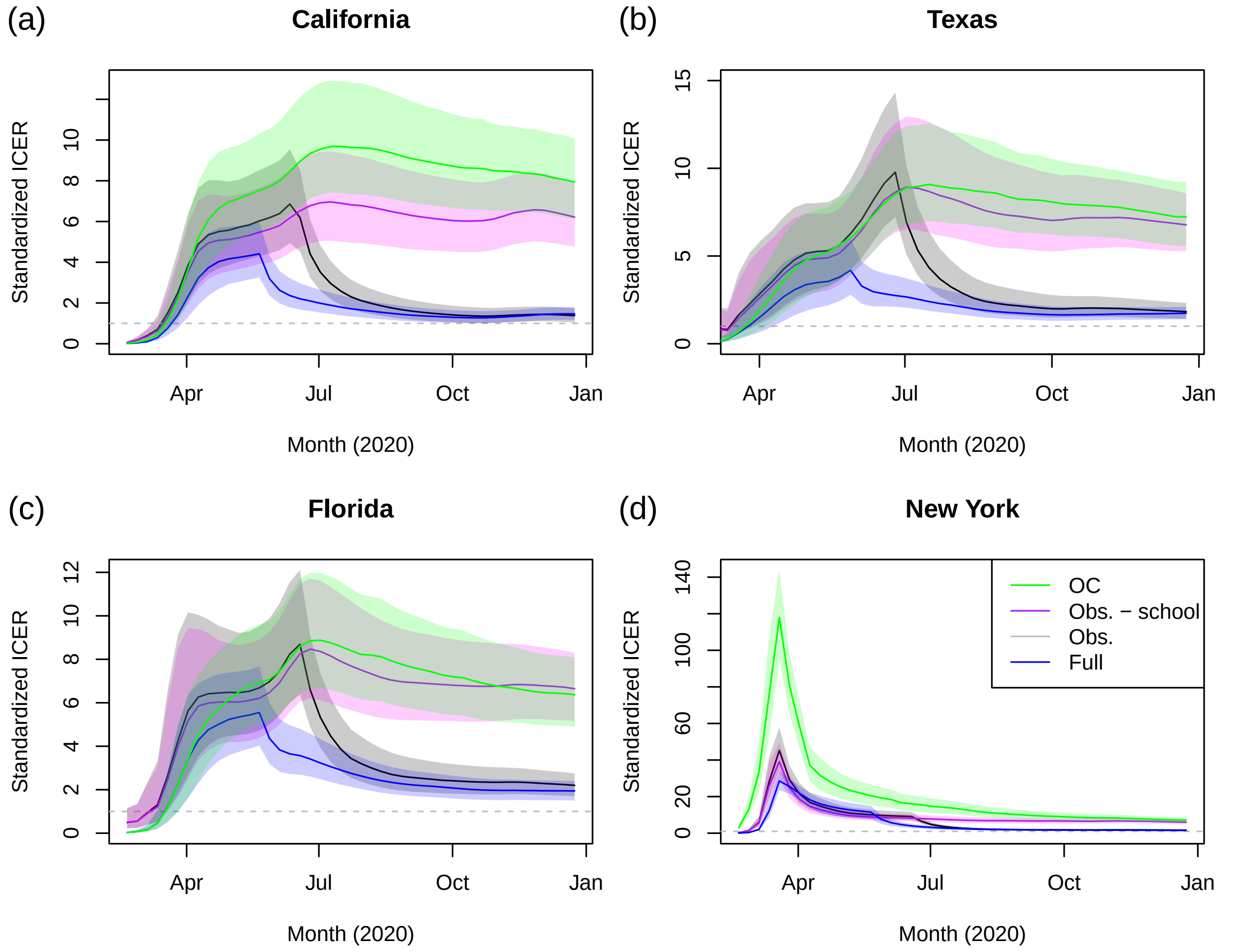

Figure S8: Posterior median and 50% credible intervals for the cumulative standardized ICER of various policies in the four most populous states.

that the VSCD is low (bottom right panels, subplots (f) and (h)), Full exceeds the cost of Obs., which exceeds the cost of Open. If both VSCD and the cost of learning loss are low (bottom left panels, subplots (e) and (g)), Obs.−school becomes cheaper than Obs. and on par with Full. These patterns are consistent across models (i)–(iii).

S.1.3 ICERs in infectious disease

The ICER is a quantity widely used in the economic evaluation of health interventions. The ICER is defined as the monetary cost of an intervention divided by the benefit it produces (as measured by some target outcome) relative to a baseline, with a larger ICER often (mis)interpreted to mean that an intervention is less cost-effective [13]. In public

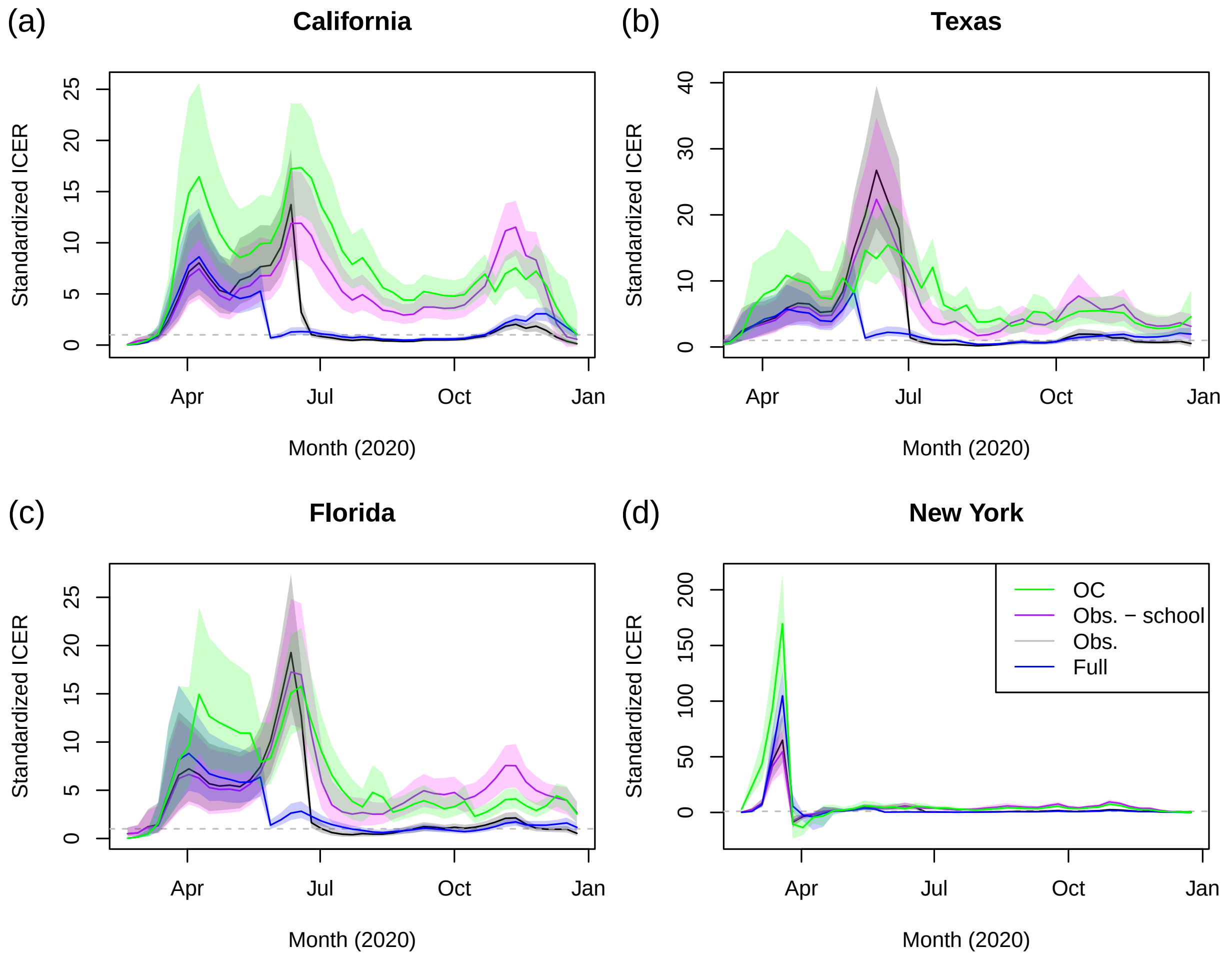

Figure S9: Posterior median and 50% credible intervals for the weekly standardized ICER of various policies in the four most populous states.

health and infectious disease, outcomes of interest include the number of quality-adjusted life years (QALYs), disability-adjusted life-years (DALYs), infections, hospitalizations, or deaths averted by the intervention. Despite their ubiquity in the health economics literature, ICERs can be difficult to interpret and are often defined, calculated, and reported inconsistently, which severely limits their practical value in cost-effectiveness analysis [13, 14]. As a result, ICER estimates for the same strategy can vary by orders of magnitude across studies, and rankings of interventions based on their reported ICERs can yield counter-intuitive results, as evidenced by a recent systematic review of economic evaluations of COVID-19 interventions [15]. For example, Podolsky *et al.* [15] find that the median ICER of school closure across studies in their review is exceeded by that of vaccination, testing, facial covering, and stay-at-home policies. (This may be due to the fact that many cost-effectiveness analyses considering school closure fail to account for costs associated to student learning loss.) Furthermore, the median ICER of mask mandates exceeds that of school closure by an order of magnitude, and the median ICER of stay-at-home orders exceeds that of school closure by nearly 3 orders of magnitude. To address these issues, some have argued instead for the use of a policy's net benefit in decision-making [13, 16, 17]. We take a similar approach, reporting the expected total cost of various strategies, which facilitates straightforward comparison of their cost-effectiveness. Nevertheless, our methodology can shed light on the appropriate use of ICERs in the economic evaluation of interventions targeting infectious disease transmission.

We argue that ICERs in the context of infectious disease interventions should be: data-driven and account for uncertainty; not based solely on (poorly calibrated, overly simplistic, or deterministic) simulation models; reported based on well-defined interventions; and calculated using the intervention's (causal) effect on the effective reproduction number $R_e(t)$, the relevant quantity governing infectious disease transmission, which is interpreted as the expected number of secondary cases resulting from an infection and can be estimated from clinical data in real time. Related to this last point, we note that, for a given intervention, the ICER is a time- and context-specific quantity. Outcomes, such as infections averted by

an intervention, depend also on the implementation of other interventions (their timing, strength, duration) as well as other factors affecting disease transmission (e.g., the duration of the pandemic, the baseline R_0 value, other epidemiological parameters, voluntary social distancing and protective measures, exogenous shocks, new variants, etc.). Therefore, in calculating and reporting the ICER, our results should account for or be as invariant as possible to contextual factors. We echo Prager *et al.* [18], who highlight the "'...importance of including a broader set of causal factors to achieve more accurate estimates of the total economic impacts of not just pandemic influenza but biothreats in general."

If we consider a blanket intervention that affects the population at large (e.g., social distancing measures), the intervention's reduction in $R_e(t)$ multiplied by the estimated size of the infectious population gives the number of infections averted by the intervention on day t, as we demonstrate below. This forms the denominator of the ICER. (On the other hand, if we consider a targeted intervention, e.g., case isolation, the number of infections averted can be estimated as the reduction in $R_e(t)$ multiplied by the number of treated subjects.) Finally, the numerator of the ICER is the cost of implementing the intervention on day t.

To demonstrate, Figures S8 and S9 exhibit cumulative and weekly standardized ICERs of various strategies relative to the Open policy—in which no NPIs are used—over time in the four most populous US states. We calculate these standardized ICERs as follows. For an NPI policy u implemented on a specific day t, let

$$R_e(u,t) := R_0(u,t)S(t)$$

denote the effective reproduction number on day t under policy u, where (in an abuse of notation) $R_0(u,t)$ is the basic reproduction number defined by our NPI regression model (equation (3) in Methods) and $S(t)$ is the susceptible fraction of the population. The number of infections averted by the policy u on day t relative to Open is then

$$\nu_a(t) := NI(t)(R_e(\mathbf{0},t) - R_e(u,t)),$$

where N is the population size, $I(t)$ is the infectious fraction of the population, and $R_e(\mathbf{0}, t)$ denotes the effective reproduction number under no interventions. In a given period of days $[T_1, T_2]$, the ICER of a policy $\mathbf{u} = \{u(t) : t \in [T_1, T_2]\}$ is then

$$\text{ICER}_{\mathbf{u}}(T_1, T_2) = \frac{c_{\text{NPI}}(\mathbf{u}) \cdot N}{\sum_{t=T_1}^{T_2} \nu_a(t)},$$

where $c_{\text{NPI}}(\mathbf{u})$ is the per capita cost of the strategy. Finally, to define the standardized ICER plotted in Figures S8 and S9, we convert infections to their monetary value using the cost of an infection c_ν—defined in Methods—and take the reciprocal, such that the standardized ICER reports the ratio of the value of infections prevented to the cost of the intervention, which is a dimensionless quantity:

$$\text{SICER}_{\mathbf{u}}(T_1, T_2) := \frac{\sum_{t=T_1}^{T_2} c_\nu \nu_a(t)}{c_{\text{NPI}}(\mathbf{u}) \cdot N}. \tag{1}$$

Based on (1), in terms of net costs, a policy $\mathbf{u}$ relative to no intervention in time period $[T_1, T_2]$: breaks even if SICER $= 1$; produces a net benefit if SICER > 1; and produces a net loss if SICER < 1.

We now return to Figure S8, which displays the cumulative standardized ICER over time, $\{\text{SICER}_{\mathbf{u}}(1, t)\}_{t=1}^{T}$ for various policies $\mathbf{u}$. We see that, relative to Open, the different containment strategies are comparable in terms of cumulative cost-effectiveness early in the pandemic, with Obs. and Full becoming less cost-effective (but still producing a net benefit by the end of 2020), mainly due to the cost of school closures exceeding 16 weeks. The OC strategy is generally the most cost-effective by the end of the year, while Obs.−school is a close second. Note that, while OC and Obs.−school are nearly equally cost-effective by the end of 2020 in Florida, this does not imply that Obs.−school is also an optimal policy—this can only be determined by looking at the net benefit of each policy. Figure S9 displays the standardized ICER of each policy in each week (conditional on that policy also being implemented in all weeks prior) relative to Open. Results are similar: OC and Obs.−school are similarly cost-effective and consistently more cost-effective than Obs. and Full; all containment strategies satisfy SICER > 1 for most of the year, implying that they

produce net benefits relative to no intervention.

The OC strategy is determined by minimizing aggregate costs accrued over the year. We can solve this problem *ex post*—after we have observed the pandemic play out—but, in principle, we cannot derive an optimal policy *ex ante*, since we cannot see the future. Indeed, while susceptible-infectious-removed (SIR) models have demonstrated remarkable utility in helping us understand infectious disease [19], modeling studies carried out early in the pandemic (e.g., Ferguson *et al.* [4]) generally failed to predict the complex and stochastic dynamics of SARS-CoV-2 (e.g., multiple waves, super-spreader events) depicted in Figure 4 in Results. As we note in Results, even when we incorporate the effects of interventions into the model, we fail to explain a substantial portion of the temporal variation in transmission rates. At its face, this may seem like a disheartening realization. However, the trends in Figures S8 and S9 have important and encouraging implications for decision-making during pandemics. Specifically, they show that the OC policy—a relatively simple combination of testing, tracing, masking, social distancing, and reactive workplace closure—was consistently highly cost-effective on a weekly basis throughout the first year of the pandemic. This implies that, had we reasonable ballpark estimates of the costs and effects of interventions to work with early on, we could have determined a nearly globally optimal strategy by choosing, at each point in time, the policy that greedily minimized the cost incurred in the next time step. In our context, we could not predict long-run transmission rates and therefore we did not need to; the myopic strategy would have produced a nearly globally optimal solution. A similar phenomenon has been documented in the control theory community [20]: the performance of control algorithms can be highly sensitive to modeling errors. Hence, if a model is misspecified, or if we can only poorly understand the behavior and evolution of a control system, simple algorithms tend to be more robust.

We turn now to the cost-effectiveness of school closure in particular. In Results, we estimate that, relative to Obs.−school, Obs. saved 77,168 (12,268–235,954) lives with the cost of school closures amounting to $2 trillion, yielding an ICER of $25.9 (8.4–156.4)

million per death prevented. By comparison, in a systematic review Juneau *et al.* [21] find that school closures during the 2009 H1N1 influenza pandemic cost \$9.86 million per death prevented, which implies that the cost of preventing a death due to H1N1 is on par with the VSL—taken to be \$10.63 million, in line with Robinson *et al.* [22], in our study. In contrast, in our sensitivity analysis in S.1.2, we find that OC policies for COVID-19 involve no school closure beyond the usual 16 weeks of break per year even when the VSCD is assumed equal to the VSL and the cost of learning loss remains at the low value used in our baseline scenario.

Qualitatively, our results are consistent with the findings of Juneau *et al.* [21] in other ways. Specifically, they find that: testing, tracing, and masking are among the most cost-effective measures; workplace and school closures are effective but costly, and hence the least cost-effective interventions; combinations of NPIs are more cost-effective than single interventions; and NPIs are more cost-effective when implemented early. While Juneau *et al.* [21] conclude that school closure is among the least cost-effective interventions based on the ICER, the value of this finding is limited for a number of reasons.

Firstly, as Paulden [13] notes, it is difficult to decide based on the ICER alone if an intervention is cost-effective in a given setting, i.e., if it would be implemented in an optimal policy in conjunction with other interventions and other external factors. Indeed, as noted above, the cost-effectiveness of an NPI varies over time and across contexts, particularly as prevalence varies. (This is one factor explaining why earlier is better with regard to the timing of NPIs: as population immunity grows, NPI implementation yields diminishing returns.) As such, calculating and ranking the ICERs of various interventions is not sufficient to determine which strategies are cost-effective [13]. However, as we demonstrate in Results, we can establish that school closure is not cost-effective by deriving the optimal policy, which frames the analysis in terms of the expected total cost—or, equivalently, the net benefit—of a policy. Our methodology goes beyond quantifying the cost-effectiveness ratios of different policies by determining which NPIs should have been used and when.

Secondly, the results of Juneau *et al.* [21] are based on analysis of school closures during

the 2009 H1N1 pandemic. The biology and epidemiology of the H1N1 flu differed from SARS-CoV-2 in important ways. In particular, it was evident early in the COVID-19 pandemic that SARS-CoV-2 was more virulent and more transmissible than H1N1, which left open the possibility that school closure would be cost-effective in combating SARS-CoV-2 transmission in 2020 despite its apparent lack of cost-effectiveness in 2009, as noted by Pasquini-Descomps *et al.* [23] and Xue *et al.* [24]. Similarly, studying the cost-effectiveness of interventions in response to outbreaks of influenza, gastroenteritis, and chickenpox in France, Adda [25] finds that school closures are not cost-effective, but they "...would become beneficial for epidemics characterized by a slightly more deadly strain." Indeed, Xue *et al.* [24], Dauelsberg *et al.* [26], Perlroth *et al.* [27], Milne *et al.* [28], and Kelso *et al.* [29] find that extended school closures are cost-effective for severe pandemics. We note that Dauelsberg *et al.* [26], Perlroth *et al.* [27], Milne *et al.* [28], and Kelso *et al.* [29] do not account for costs associated to student learning loss and Xue *et al.* [24] do not consider interventions other than school closure as available tools in their model. As we show in Results, in the context of COVID-19, school closures are not cost-effective.

S.2 NPI regression model results

S.2.1 Fit to data

Regarding the fit of the NPI regression model to the estimated transmission rates, the posterior median R^2 is 0.59, indicating that a substantial proportion of the variance in transmission rates remains unexplained by NPIs or behavioral response to the fear of infections. Indeed, there are numerous factors affecting SARS-CoV-2 transmission—including super-spreader events and introduction of new infections from outside the state—accounted for by the error terms $\varepsilon^{(s)}(w)$ that are difficult to predict. Figure S10 plots the *maximum a posteriori* (MAP) trajectory of R_0 output from the epidemiological model in the four most populous states—California, Texas, Florida, and New York—against the posterior predictive distribution from the NPI model fit to this output. The model fits the data well,

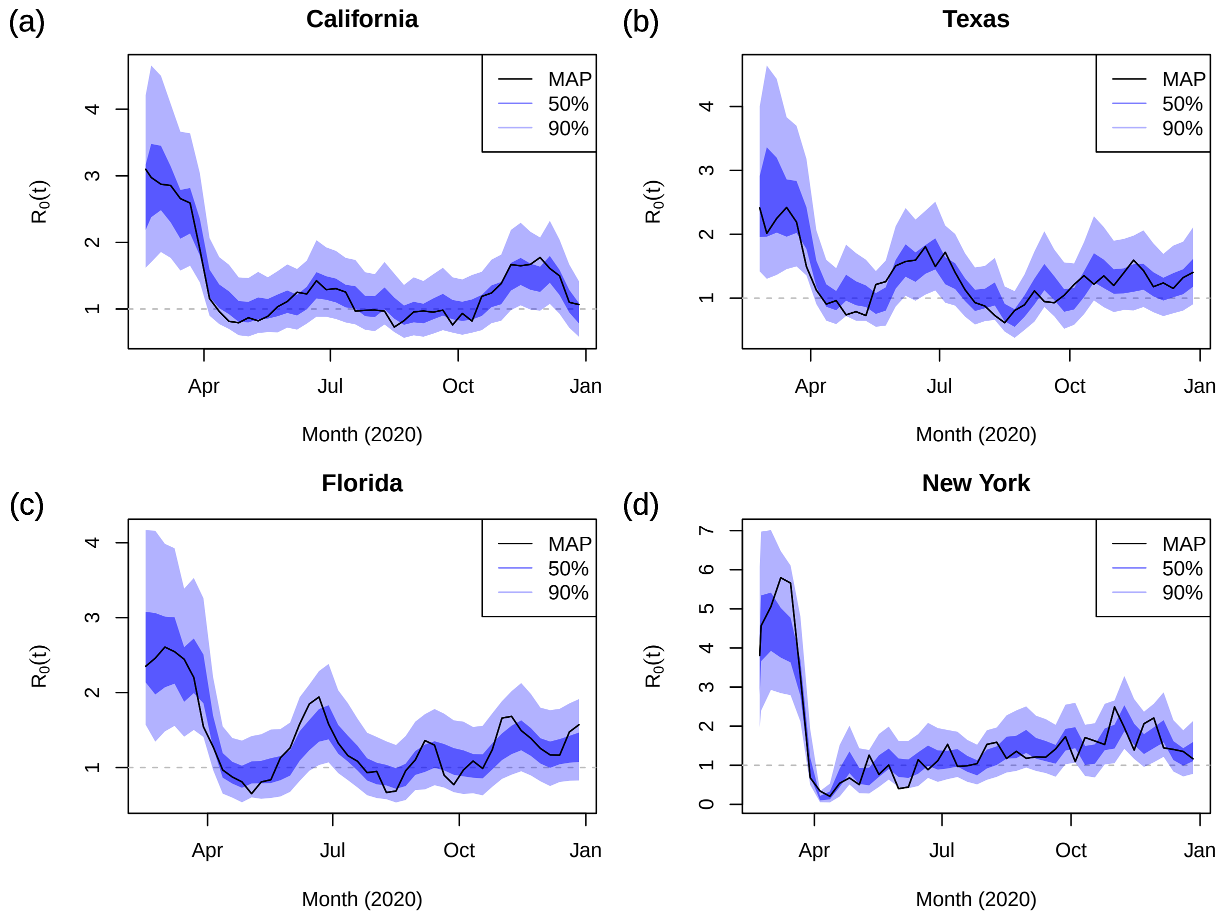

Figure S10: Time-varying transmission rates in four states. In black, the MAP trajectory output by the epidemiological model. In dark and light blue, respectively, the 50% and 90% credible intervals of the posterior predictive distribution from the fitted NPI regression model.

but cannot capture unpredictable shocks in transmission, which are reflected in future predicted values of the transmission rate through the AR(1) term. The AR(1) parameter φ is 0.76 (0.68–0.83), indicating a high degree of residual autocorrelation in the weekly-varying reproduction number $R_0^{(s)}(w)$ across states. Finally, the posterior degrees of freedom ν_ε for the Student-t distributed residuals $\varepsilon^{(s)}(w)$ is 2.9 (2.3–3.7), indicating that a heavy-tailed error distribution is appropriate for these data.

S.2.2 Total effect of NPIs

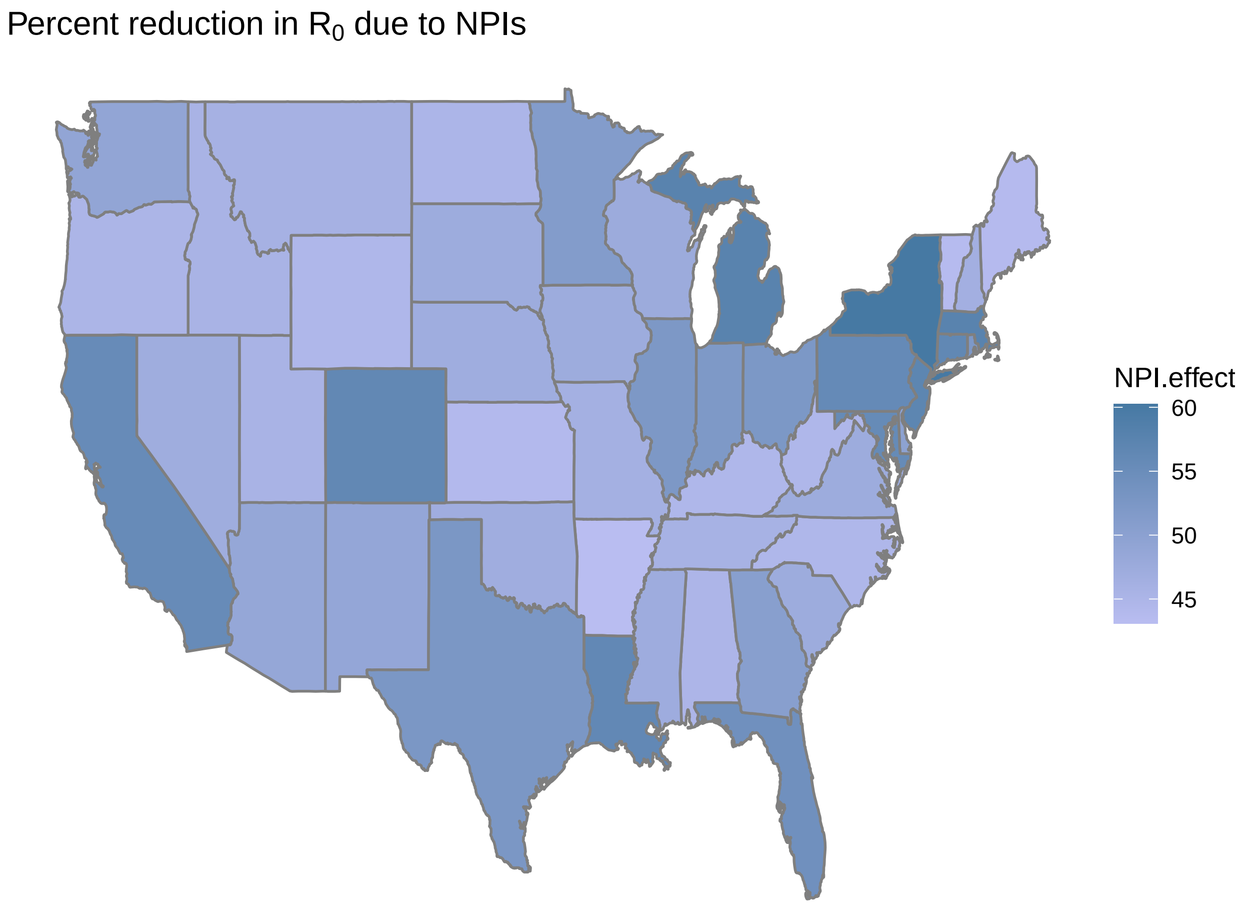

Figure S11: Posterior median total percent reduction in R_0 due to NPIs by state.

Zooming in on the state-level results, we can quantify the total effect of NPIs on transmission in state s by

$$\alpha^{(s)} := \sum_{k=1}^{p} \beta_u^{(s)}(k),$$

with $p^{(s)} = 100(1 - \exp(\alpha^{(s)}))$ representing the total percent reduction in $R_0^{(s)}$ under full lockdown. Figure S11 displays the geographic distribution of the posterior median of $p^{(s)}$ across states. Overall, NPIs tend to be more effective in more urbanized and populous states. Some of this variation may be explained by the literature on political polarization and partisan social distancing during the pandemic [30–36]. Alternatively, and related to our discussion in the previous paragraph, we note that rural states tend to have lower baseline $R_0^{(s)}$ values, possibly due to later importation of the virus and lower levels of social mixing. With a lower ceiling in these states, NPIs have less room to suppress transmission.

Finally, note that the p-vector

$$\rho^{(s)} := \beta_u^{(s)} / \alpha^{(s)}$$

consists of weights representing the proportional contribution of each NPI to the total reduction of transmission. Here $\rho^{(s)}$ can be thought of as defining a data-driven "stringency index" combining NPIs according to their strengths in a single-number summary of the stringency of government restrictions, as opposed to previously defined measures, such as OxCGRT's stringency index, which average NPIs uniformly without regard for their varying effects on transmission [37].

S.2.3 Effects of individual NPIs

Figure S12 shows the pooled effect of each NPI, $\beta_u(k), k = 1, \ldots, p$, quantified as a percent reduction in R_0, across the robust log-linear regression models defined in Methods. We focus discussion on model (ii), which includes the effects of deaths and removals (but not infections) incident in the prior week. Mask mandates are the most effective intervention, reducing R_0 by 19.0% (6.1%–28.5%). As shown in Figure S12, this effect attenuates from 19.7% (7.3%–28.5%) to 15.4% (3.3%–26.9%) as we move from model (i) to model (iii), controlling for removals and infections incident in the prior week. By comparison, Sharma *et al.* [38] estimate that mask mandates reduced transmission rates by 12% (7%–17%) in the second wave in Europe. Based on data from 190 countries between January and

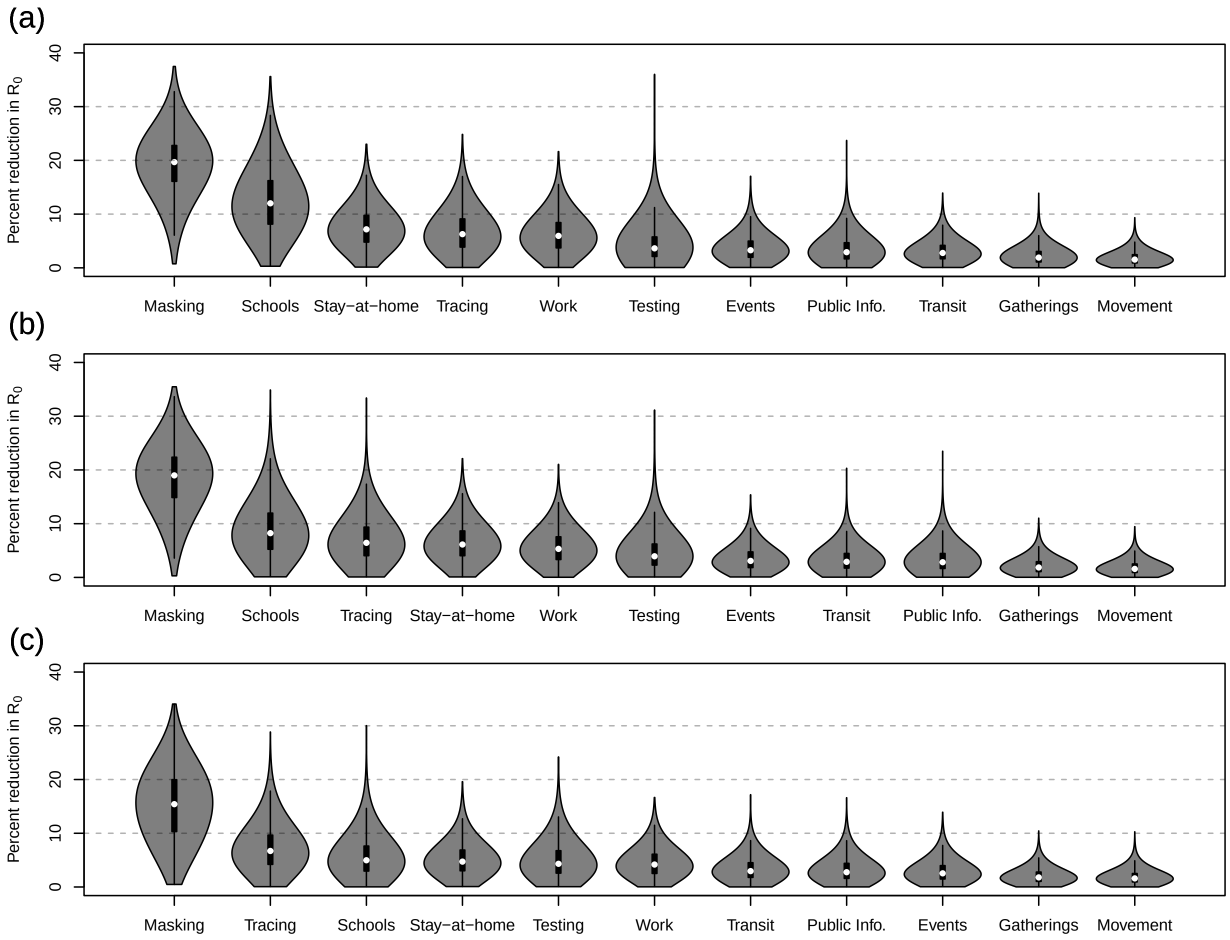

Figure S12: Posterior violin plots of global NPI effects, quantified as the percent reduction in R_0, across regression models (i)–(iii) from (a) to (c), respectively.

April 2020, Bo *et al.* [39] conclude that mask mandates were associated with a 15.1% (7.9%–21.8%) decline in transmission. Karaivanov *et al.* [40] find a 22 percent weekly reduction in new COVID-19 cases due to mask mandates in Canada in the summer of 2020. Studying the 2020 spring wave in New York City, Yang *et al.* [41] find that masking was associated with a 7% transmission reduction overall and up to 20% reduction for people over age 65. Estimating the causal effects of a number of interventions in the US, Chernozhukov *et al.* [42] demonstrate that masking policies were highly effective, leading to a reduction in the weekly growth rate of cases and deaths by more than 10 percentage points, with their conclusions holding robustly across model specifications; on the other hand, the effects of stay-at-home orders and business and school closures are much more uncertain. Qualitatively, our results are consistent with a number of other studies demonstrating the efficacy of mask mandates and the protective effects of face mask use [43–48].

Behind mask mandates, in our model (ii) school closure reduces the transmission rate by 8.2% (1.5%–20.2%). As shown in Figure S12, this effect attenuates from 12.0% (3.0%–24.8%) to 5.0% (0.8%–14.3%) as we move from model (i) to model (iii), controlling for removals and infections incident in the prior week. By comparison, Brauner *et al.* [49] and Banholzer *et al.* [50] find that school closures led to 38% (16%–54%) and 17% (-2%–36%) transmission reductions in the first wave of 2020, respectively, and Sharma *et al.* [38] estimate a 7% (4%–10%) transmission reduction due to school closures in the second wave. Studying influenza outbreaks, Cauchemez *et al.* [51] found that school holidays led to a 20–29% transmission reduction among children with no detectable effect on transmission among adults. Qualitatively, our results are consistent with a number of other studies finding school closures to be one of the NPIs most effective in reducing transmission [52–58].

As with school closures, we are unable to rule out small effects for the remaining NPIs. Workplace closure reduced transmission by 5.3% (0.9%–12.5%). By comparison, Brauner *et al.* [49], Sharma *et al.* [38], and Banholzer *et al.* [50] find that business closures led to a 27% (-3%–49%), 35% (29%–41%), and 18% (-4%–40%) reduction in R_0, respectively.

The combination of social distancing measures yields a 18.9% (10.9%–28.1%) reduction in R_0, which is on par with the individual effect of mask mandates. (As in Methods, we define social distancing measures as the combination of stay-at-home orders, restrictions on gatherings, restrictions on internal movement, public information campaigns, public transit closures, and public event cancellations.) Looking at individual social distancing measures, we estimate that stay-at-home orders reduced R_0 by 6.1% (1.2%–14.1%), which is comparable to other estimates in the literature: Bodenstein *et al.* [59] report a 6.5% transmission reduction; Brauner *et al.* [49] report a 13% (-5%–31%) reduction; and Banholzer *et al.* [50] report a 4% (-6%–17%) reduction. Restrictions on gatherings reduced R_0 by a modest 1.9% (0.3%–5.7%). However, a number of other studies estimate large effects of strict gathering restrictions: Brauner *et al.* [49] report a 42% (17%–60%) transmission reduction; Sharma *et al.* [38] report a 26% (18%–32%) reduction; Banholzer *et al.* [50] report a 37% (21%–50%) reduction; and Bo *et al.* [39] report a 42.9% (41.6%–44.2%) reduction associated to social distancing measures more broadly. (We note that these are association studies based on observational data not controlling for potential confounders.) Nevertheless, even with our relatively conservative estimates of the effects of social distancing measures, we find that they are cost-effective interventions in combination.

Finally, our estimates of the effects of testing and tracing policies—which yield 3.9% (0.6%–12.3%) and 6.4% (1.1%–16.0%) reductions in R_0, respectively—allow for the possibility of both small and large effects on transmission. Evidence on the effectiveness of contact tracing, in particular, is mixed. Rainisch *et al.* [60] concluded that case investigation and contact tracing were effective in reducing transmission based on their estimates of the number of COVID-19 cases and hospitalizations averted by these measures in the US. Wang *et al.* [61] find that testing and case isolation were effective, but the effect of contact tracing is marginal due to slow follow-up times in case investigation. They note that contact tracing can be more effective if follow-up is accelerated. In modeling studies, Hellewell *et al.* [62] and Davis *et al.* [63] find that contact tracing can be effective if carried out well—with the latter reporting a potential 15% reduction in R_0—but that its effec-

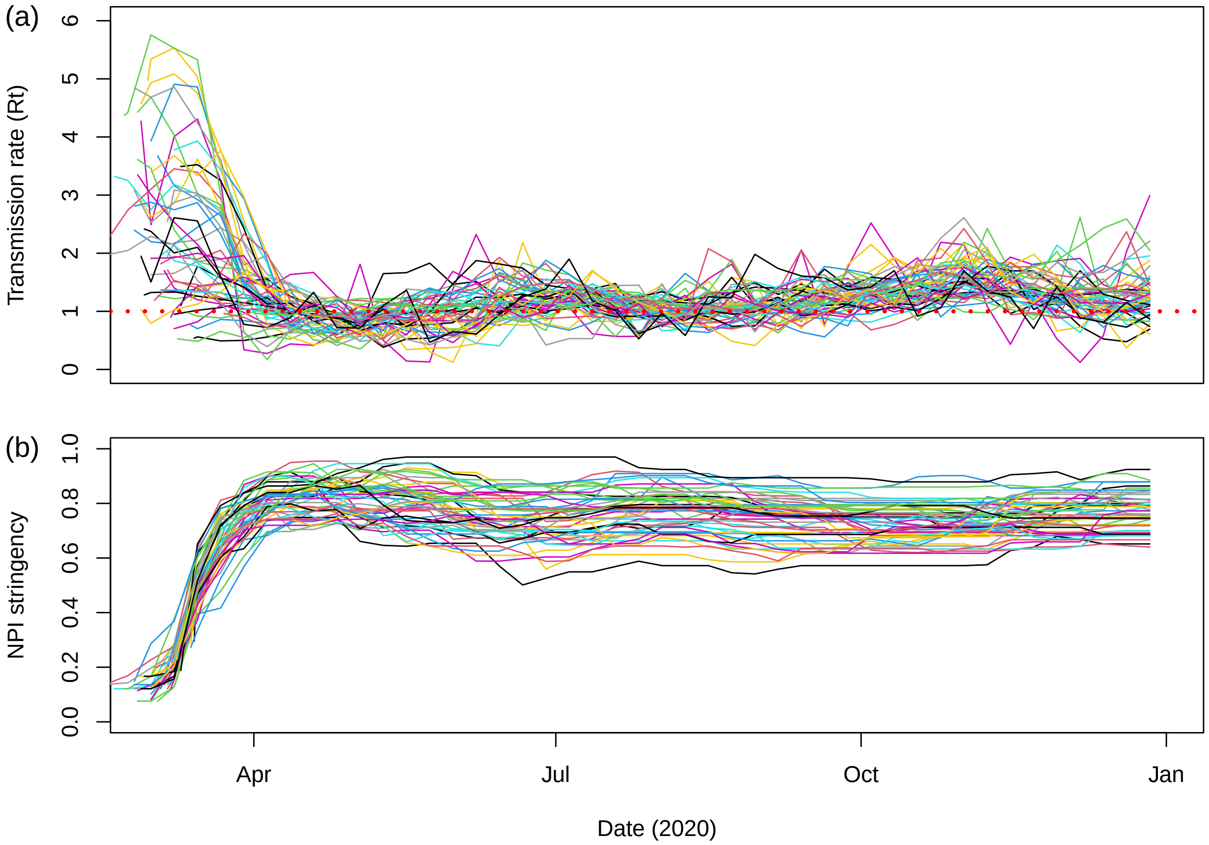

Figure S13: For each US state, a single posterior trajectory of the estimated transmission rate (a) and NPI stringency (b) over time in 2020.

tiveness is dependent on a number of epidemiological and implementation-related factors, including tracing coverage and speed. Nevertheless, even allowing for small effects, we find that testing and tracing are highly cost-effective NPIs owing to their low cost relative to other interventions.

S.2.4 Effect of lagged NPIs

We assume that the NPIs in place in week w directly affect transmission rates in week w (a zero-week lag). This is in line with a number of other studies estimating the effects of NPIs (e.g., Flaxman *et al.* [3], Sharma *et al.* [38], and Brauner *et al.* [49]), which do not account for lagged effects of NPIs. This is not an issue for closure policies (e.g., school, business, and transit closures), as they take effect immediately. For other policies, it is reasonable to assume that behavioral responses occur quickly. For example, Alexander & Karger [64]

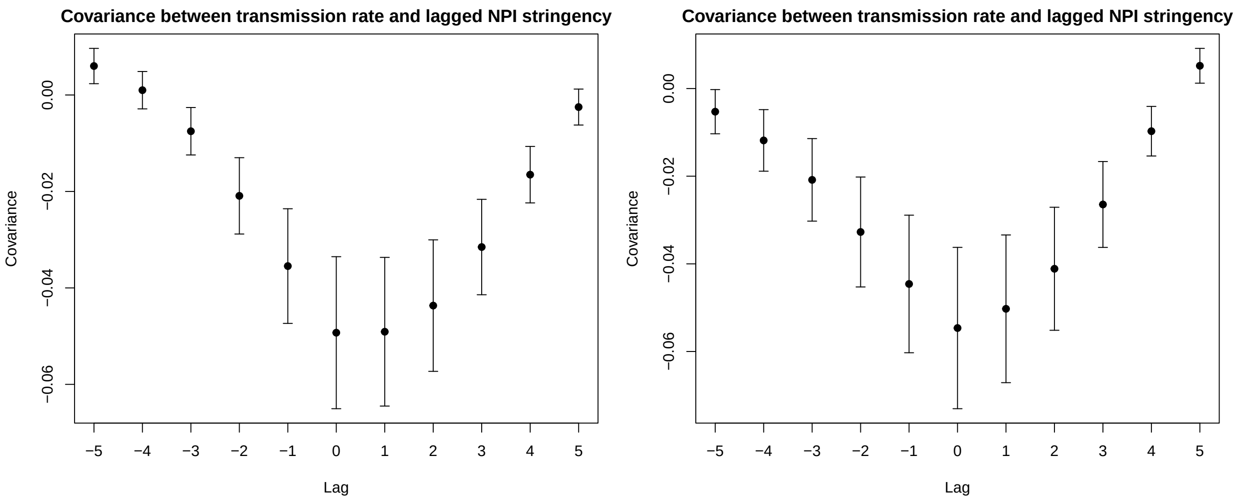

(a) Simple average (b) Weighted average

Figure S14: Estimated covariance between transmission rates and lagged NPI stringency across states, where NPI stringency is defined as (a) a simple average of NPI values, as in OxCGRT, and (b) a weighted average of NPI values based on the estimated NPI model regression coefficients.

find that mobility and consumer spending declined consistently within two days of when US counties enacted stay-at-home orders.

Figure S13 plots transmission rates and the NPI stringency index (defined as a simple average of the 11 NPI values, as in OxCGRT [37]) across US states in each week w in 2020. To assess the effect of lagging the NPIs, we conducted a covariance analysis, which is plotted in Figure S14. The x-axis shows the lag in weeks, with lag 0 corresponding to no lag (as used for the analysis in the paper), positive values implying that NPIs are being compared to future transmission rates, and negative values implying that NPIs are being compared to past transmission rates. Figure S14a shows the mean covariance across states (with 95% error bars) between transmission rates and the (lagged) NPI stringency index, defined as a simple average of NPI values. Figure S14b instead uses an NPI stringency index defined as a weighted average of NPI values based on our estimated NPI model regression coefficients.

In both plots, the covariance tends to have the largest (negative) magnitude when the lag is 0 weeks, although the difference with nearby means is not statistically significant. This suggests that using a lag of 0 weeks (as we do in the paper) is appropriate. Also, given

the similar covariance using a lag of 1 week, our results would be unlikely to change much in this case. However, using other lag values would attenuate the relationship between NPIs and transmission rates, potentially affecting our conclusions about the cost-effectiveness of interventions. For example, if workplace closures or social distancing orders were estimated to have smaller effects on transmission based on a 2- or 3-week lag, their cost-effectiveness would decline.

S.3 Epidemiological and economic parameter specification

Parameter	Value	Reference
δ^{-1}: Mean duration of latent period	5.5	Xin *et al.* [65]<br>Linton *et al.* [66]<br>Lauer *et al.* [67]<br>Gallo *et al.* [68]<br>Wu *et al.* [69]
γ^{-1}: Mean duration of infectious period	5.0	Hakki *et al.* [70]
μ^{-1}: Mean time to death after removal	10.5	Linton *et al.* [66]<br>Byrne *et al.* [71]
τ_D: Mean time from exposure to death	21.0	Ward & Johnsen [72]
ι_s: State-specific IFR	Varying	Irons & Raftery [73]
$R_0^{\max}$: Upper bound on $R_0(t)$	6.5	Liu *et al.* [74]
Mean time from case reporting to death	8.053	Jin [75]
Standard deviation in time from case reporting to death	4.116	Jin [75]

Table S1: Epidemiological parameters. All times are in days.

Parameter	Value	Reference
2019 GDP per capita by state	Varying ($39000–211000)	U.S. Bureau of Economic Analysis [76]
2019 per capita income by state	Varying ($39000–85000)	U.S. Bureau of Economic Analysis [77]
2019 US median personal income	$35980	U.S. Census Bureau [78]
2019 population by state	Varying (0.575–39.5 million)	U.S. Bureau of Economic Analysis [79]
2019 US GDP current dollar growth rate	4.1%	U.S. Bureau of Economic Analysis [80]
VSCD	Low: $4.47 million<br>High: $10.63 million	Robinson *et al.* [22]
Voluntary social distancing cost per COVID-19 infection by state	Varying ($1491–3199)	Aum *et al.* [81]
Productivity cost of COVID-19 infection	One week of state median income	U.S. Bureau of Economic Analysis [77] and Skarp *et al.* [82]
Average medical cost of COVID-19 infection	$3045	Bartsch *et al.* [83] and DeMartino *et al.* [84]
Net present value of GDP loss due to learning loss by state	Low: 9% GDP per 0.33 school-years<br>High: 69% GDP per 0.33 school-years	Psacharopoulos *et al.* [85]<br>Hanushek & Woessmann [86]
Learning loss accrued during COVID-19	0.35 school-years	Betthäuser *et al.* [87] and Fahle *et al.* [88]
Direct GDP loss due to school closure	0.2% GDP per four weeks	Lempel *et al.* [89], Sadique *et al.* [90], and Viner *et al.* [91]
Employment rate decrease due to workplace closure	Low: 2%; Med: 4%; High: 6%	Barrot *et al.* [12], Gupta *et al.* [92], and Crucini & O'Flaherty [93]
Employment rate decrease due to social distancing mandates	4%	Bodenstein *et al.* [59], Gupta *et al.* [92], Crucini & O'Flaherty [93], Baek *et al.* [94], and Coibion *et al.* [95]

Table S2: Economic parameters. All costs are in 2020 US dollars. Sensitivity analysis is conducted with respect to the VSCD, cost of learning loss, and cost of workplace closure.

Parameter	Value	Reference
Daily personal mask expenditure	\$0.32	Skarp *et al.* [82] and Bartsch *et al.* [96]
Cost of a PCR test	\$100	Skarp *et al.* [82], Lo *et al.* [97], and Sharfstein [98]
Daily rate of testing capacity increase by state	Varying (7–40 tests per million pop.)	The Atlantic [99]
Cost of contact tracing per index case	\$66.50	Skarp *et al.* [82], Fields *et al.* [100], and Spencer [101]
Weekly rate of contact tracing capacity increase	4.72 cases per 100k pop.	Rainisch *et al.* [60] and Lash *et al.* [102]

Table S3: Masking, testing, and tracing parameters. All costs are in USD2020.

S.4 State-specific epidemiological model results

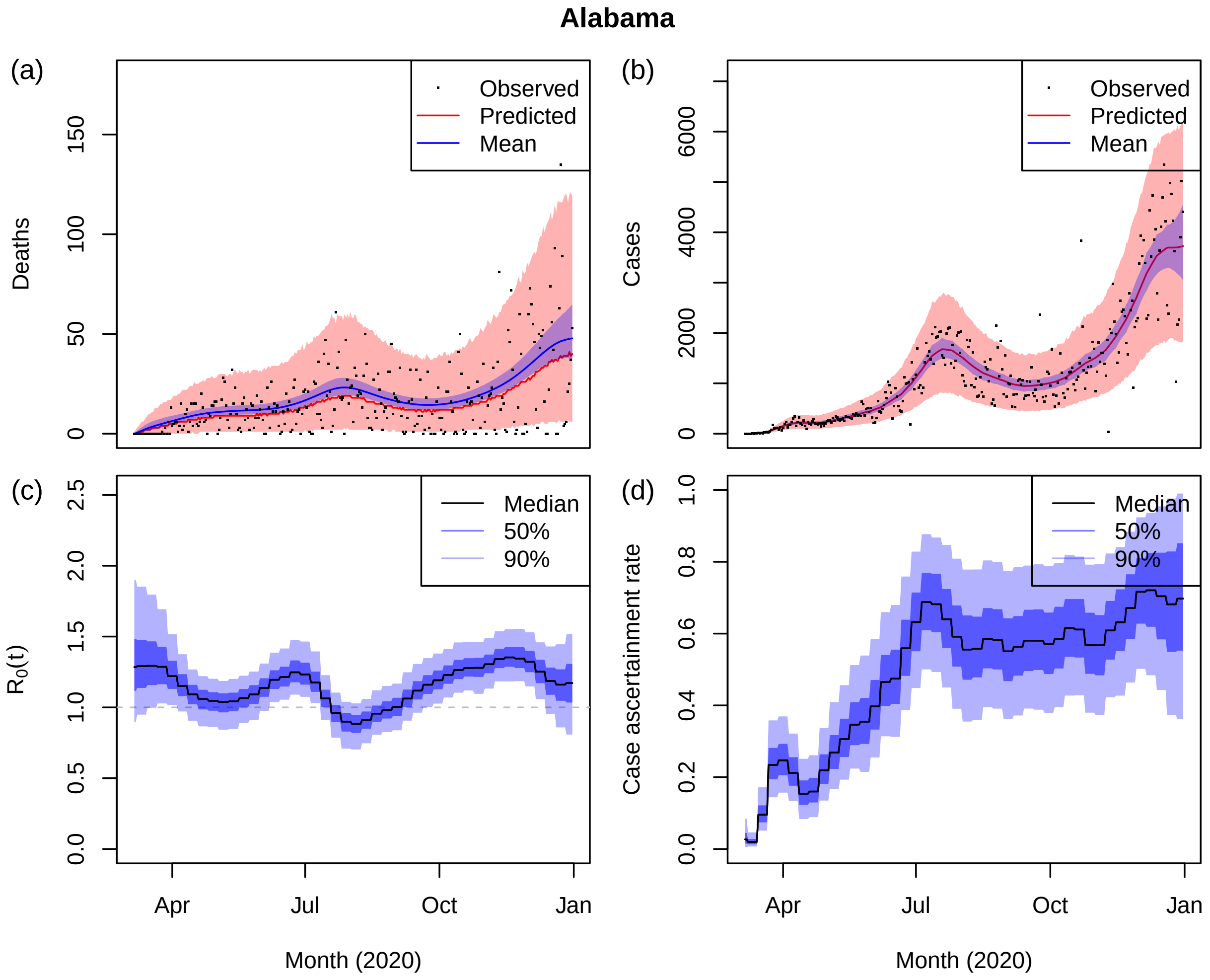

Figure S15: SEIRD model fit to COVID-19 data. **Top panels:** observed (a) deaths $d(t)$ and (b) cases $c(t)$ are plotted in black. Median and 90% credible intervals of the posterior predictive distributions of $d(t)$ and $c(t)$ are in red. Posterior median and 90% credible intervals of the underlying mean parameters $m_D(t)$ and $m_C(t)$ are in blue. **Bottom panels:** posterior median, 50%, and 90% credible intervals for (c) the basic reproduction number $R_0(t)$ and (d) the case ascertainment rate CAR(t).

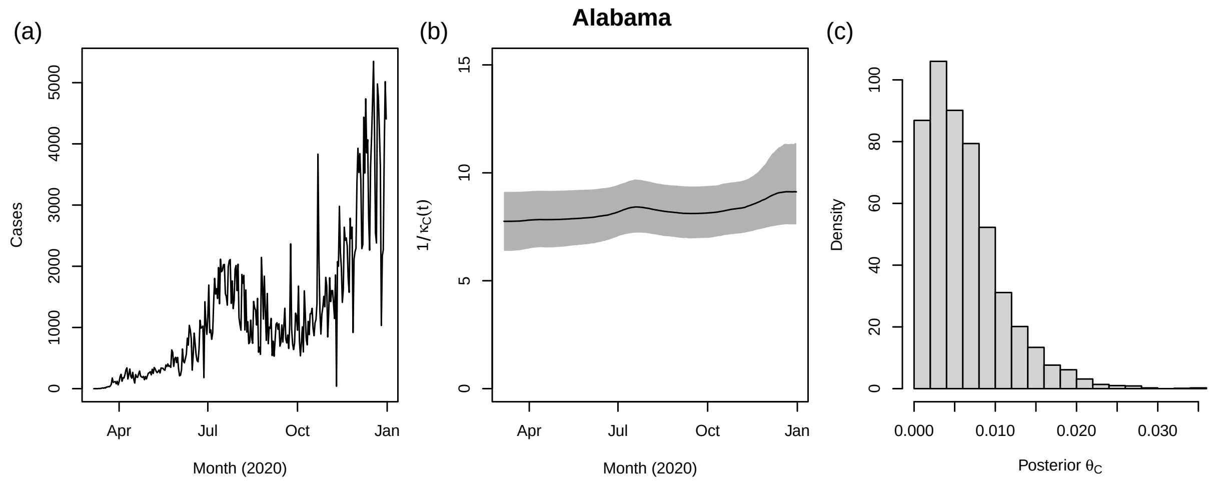

Figure S16: Overdispersion and zero-inflation in state-level clinical case data. (a) Reported cases. (b) Posterior median and 90% credible interval for the time-varying negative binomial overdispersion parameter $\kappa_C(t)^{-1}$. (c) Posterior histogram for the zero-inflation parameter θ_C.

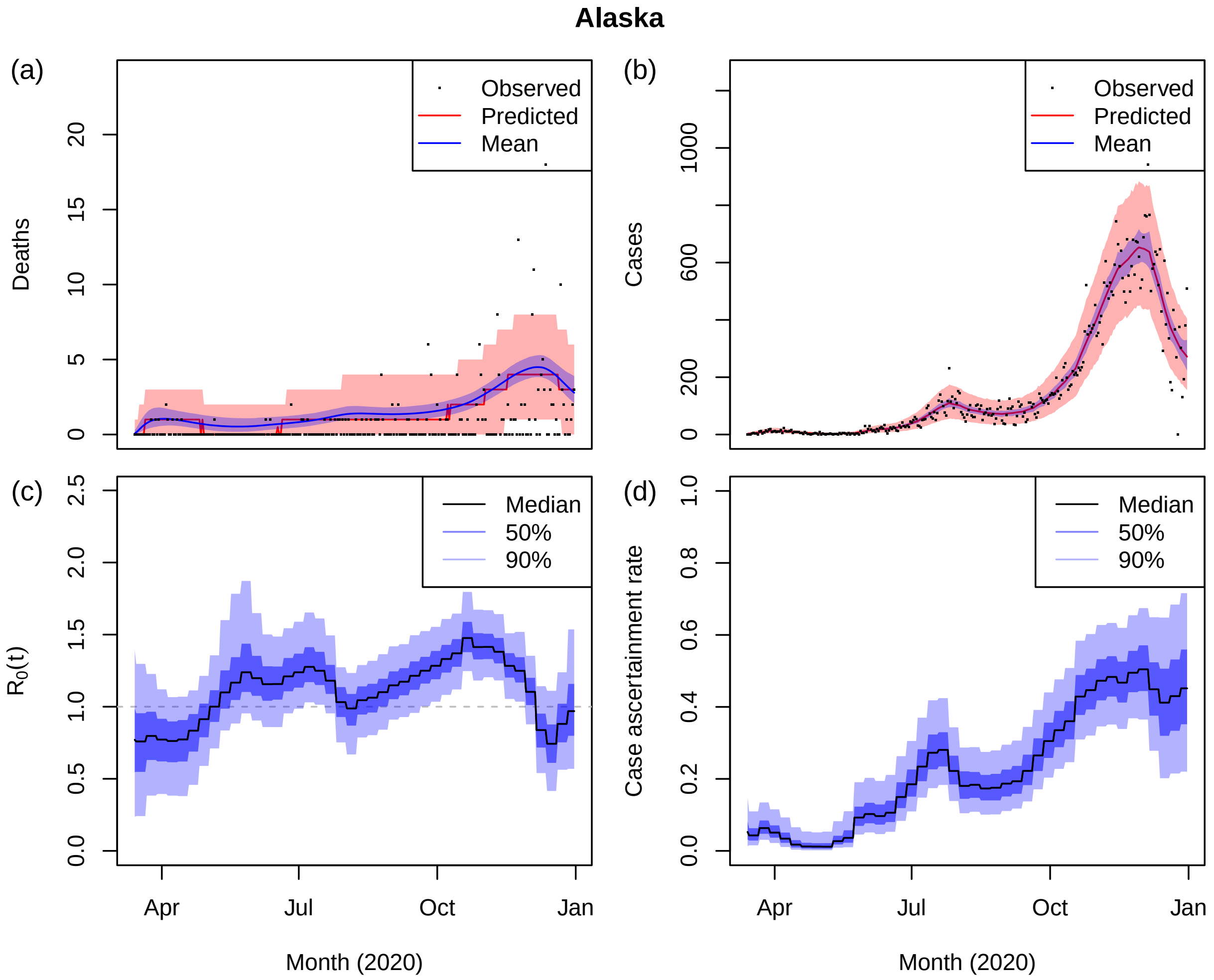

Figure S17: SEIRD model fit to COVID-19 data. **Top panels:** observed (a) deaths $d(t)$ and (b) cases $c(t)$ are plotted in black. Median and 90% credible intervals of the posterior predictive distributions of $d(t)$ and $c(t)$ are in red. Posterior median and 90% credible intervals of the underlying mean parameters $m_D(t)$ and $m_C(t)$ are in blue. **Bottom panels:** posterior median, 50%, and 90% credible intervals for (c) the basic reproduction number $R_0(t)$ and (d) the case ascertainment rate $\mathrm{CAR}(t)$.

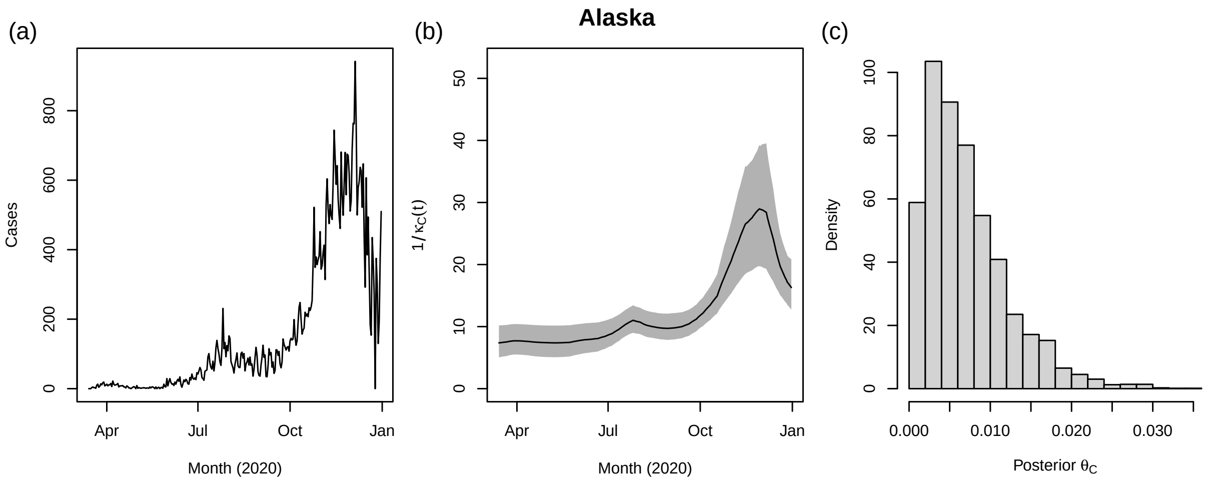

Figure S18: Overdispersion and zero-inflation in state-level clinical case data. (a) Reported cases. (b) Posterior median and 90% credible interval for the time-varying negative binomial overdispersion parameter $\kappa_C(t)^{-1}$. (c) Posterior histogram for the zero-inflation parameter θ_C.

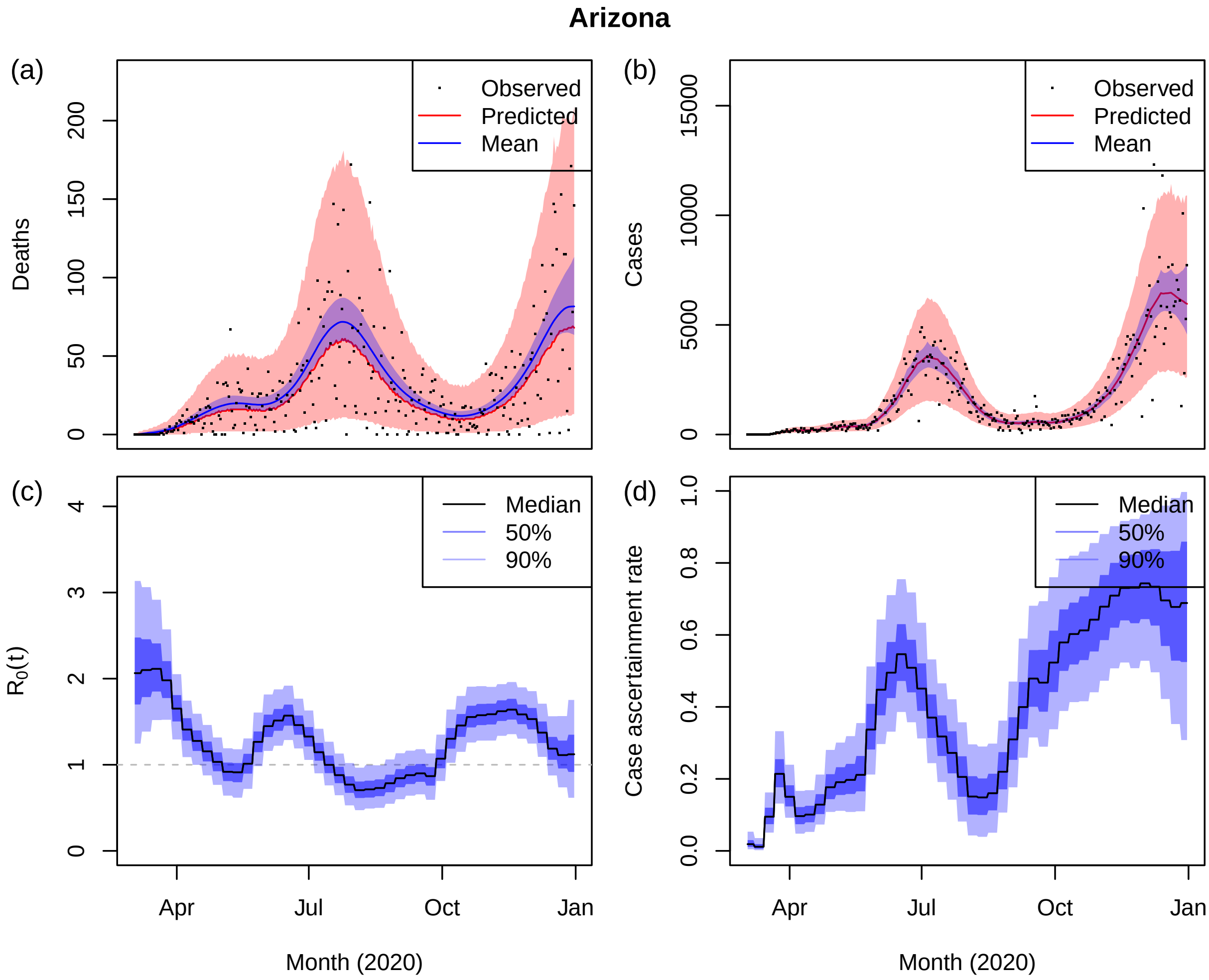

Figure S19: SEIRD model fit to COVID-19 data. **Top panels:** observed (a) deaths $d(t)$ and (b) cases $c(t)$ are plotted in black. Median and 90% credible intervals of the posterior predictive distributions of $d(t)$ and $c(t)$ are in red. Posterior median and 90% credible intervals of the underlying mean parameters $m_D(t)$ and $m_C(t)$ are in blue. **Bottom panels:** posterior median, 50%, and 90% credible intervals for (c) the basic reproduction number $R_0(t)$ and (d) the case ascertainment rate CAR(t).

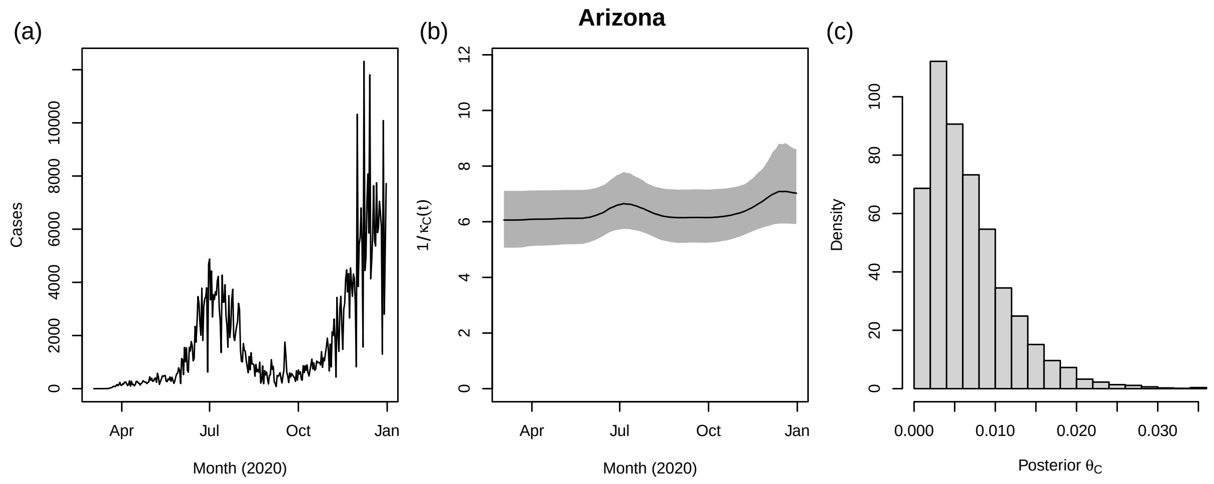

Figure S20: Overdispersion and zero-inflation in state-level clinical case data. (a) Reported cases. (b) Posterior median and 90% credible interval for the time-varying negative binomial overdispersion parameter $\kappa_C(t)^{-1}$. (c) Posterior histogram for the zero-inflation parameter θ_C.

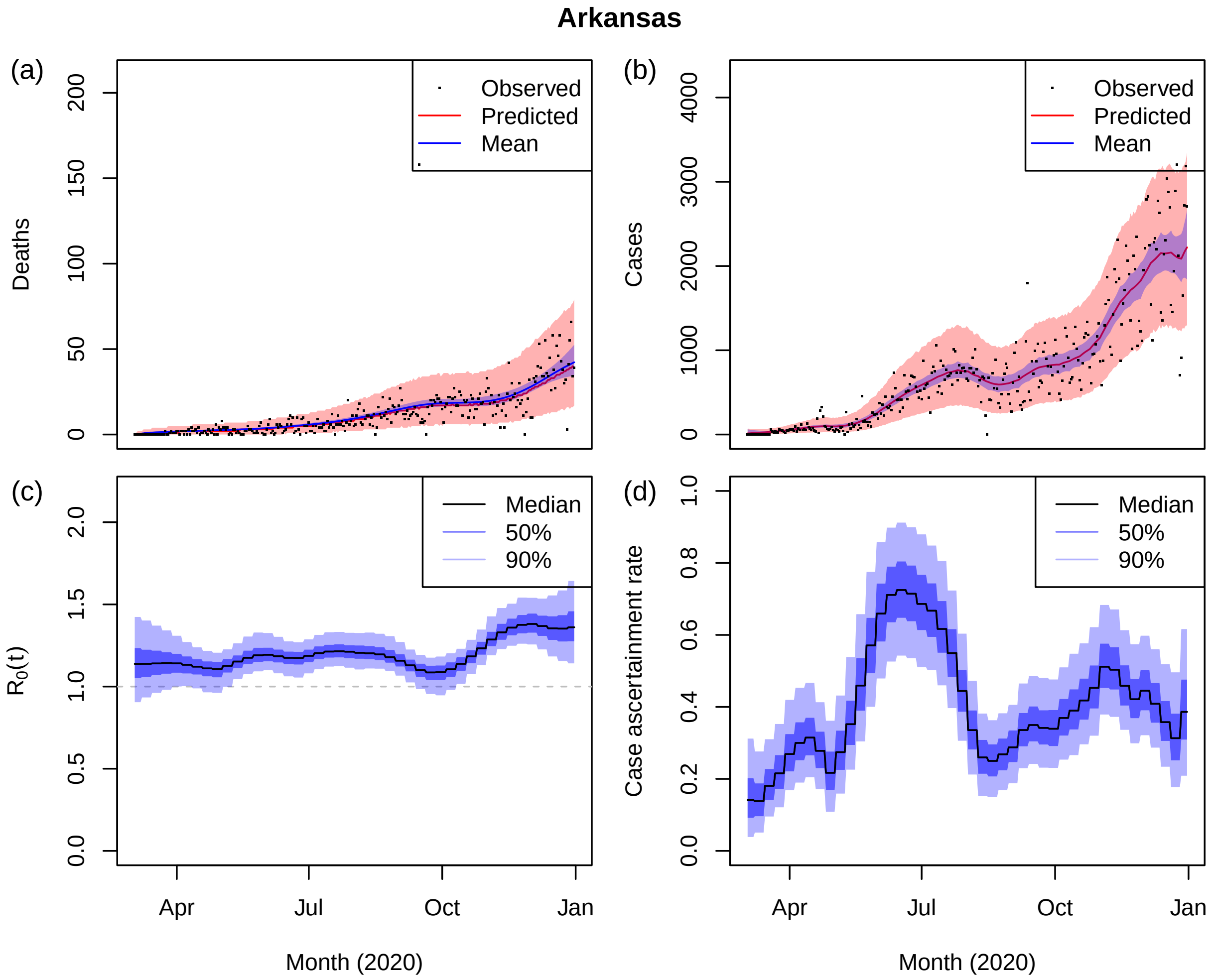

Figure S21: SEIRD model fit to COVID-19 data. **Top panels:** observed (a) deaths $d(t)$ and (b) cases $c(t)$ are plotted in black. Median and 90% credible intervals of the posterior predictive distributions of $d(t)$ and $c(t)$ are in red. Posterior median and 90% credible intervals of the underlying mean parameters $m_D(t)$ and $m_C(t)$ are in blue. **Bottom panels:** posterior median, 50%, and 90% credible intervals for (c) the basic reproduction number $R_0(t)$ and (d) the case ascertainment rate $\text{CAR}(t)$.

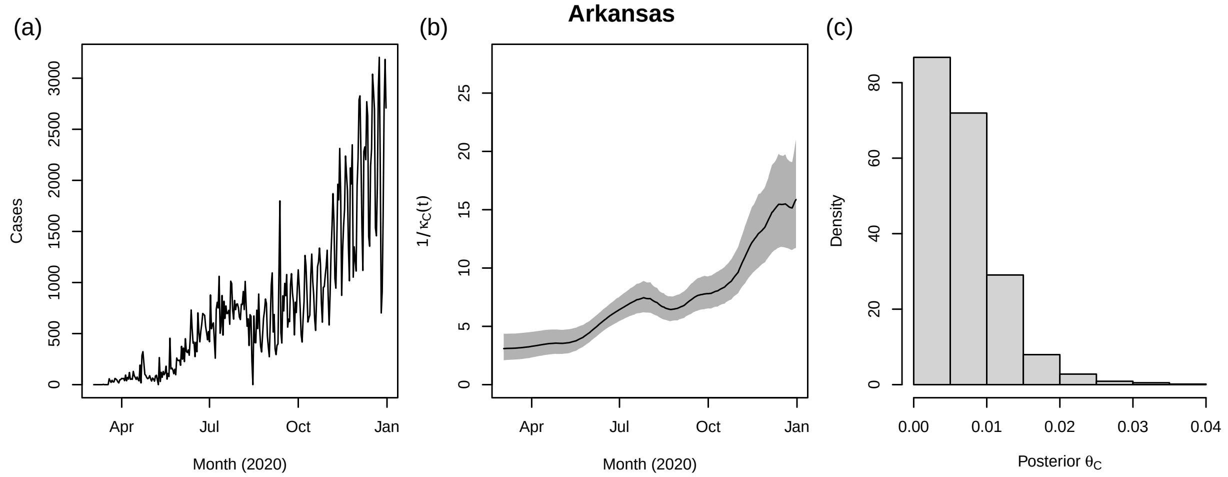

Figure S22: Overdispersion and zero-inflation in state-level clinical case data. (a) Reported cases. (b) Posterior median and 90% credible interval for the time-varying negative binomial overdispersion parameter $\kappa_C(t)^{-1}$. (c) Posterior histogram for the zero-inflation parameter θ_C.

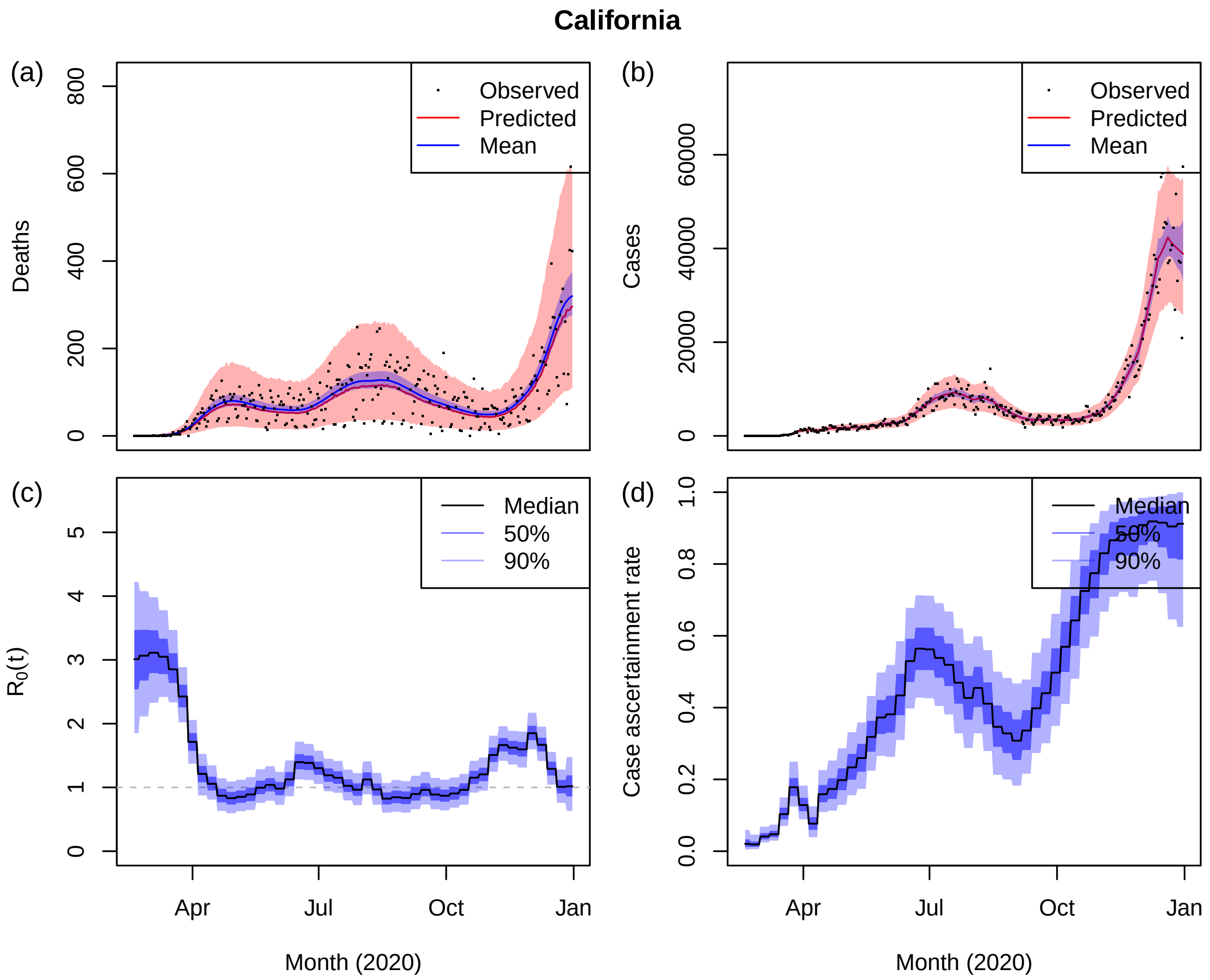

Figure S23: SEIRD model fit to COVID-19 data. **Top panels:** observed (a) deaths $d(t)$ and (b) cases $c(t)$ are plotted in black. Median and 90% credible intervals of the posterior predictive distributions of $d(t)$ and $c(t)$ are in red. Posterior median and 90% credible intervals of the underlying mean parameters $m_D(t)$ and $m_C(t)$ are in blue. **Bottom panels:** posterior median, 50%, and 90% credible intervals for (c) the basic reproduction number $R_0(t)$ and (d) the case ascertainment rate $\mathrm{CAR}(t)$.

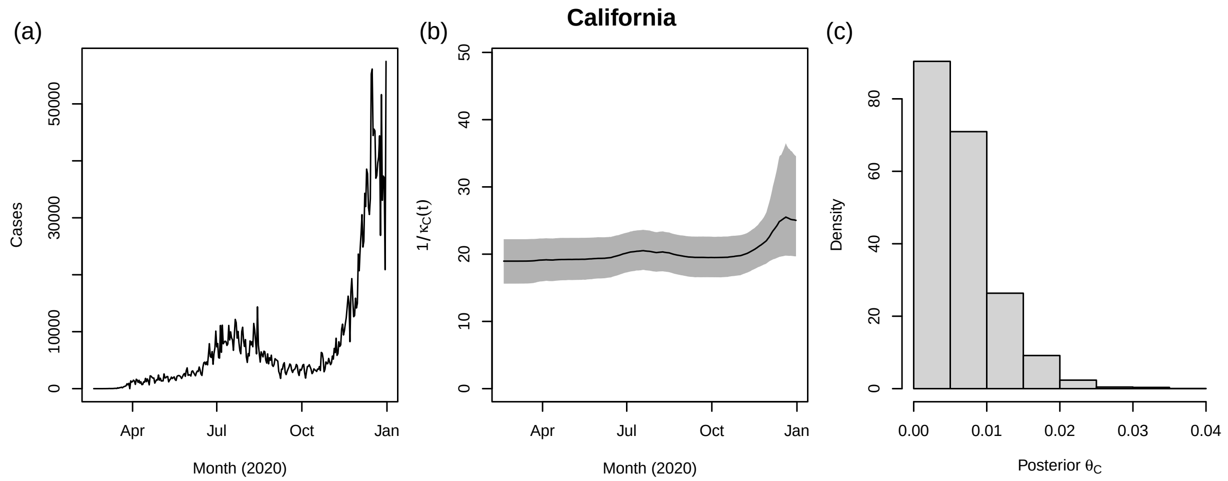

Figure S24: Overdispersion and zero-inflation in state-level clinical case data. (a) Reported cases. (b) Posterior median and 90% credible interval for the time-varying negative binomial overdispersion parameter $\kappa_C(t)^{-1}$. (c) Posterior histogram for the zero-inflation parameter θ_C.

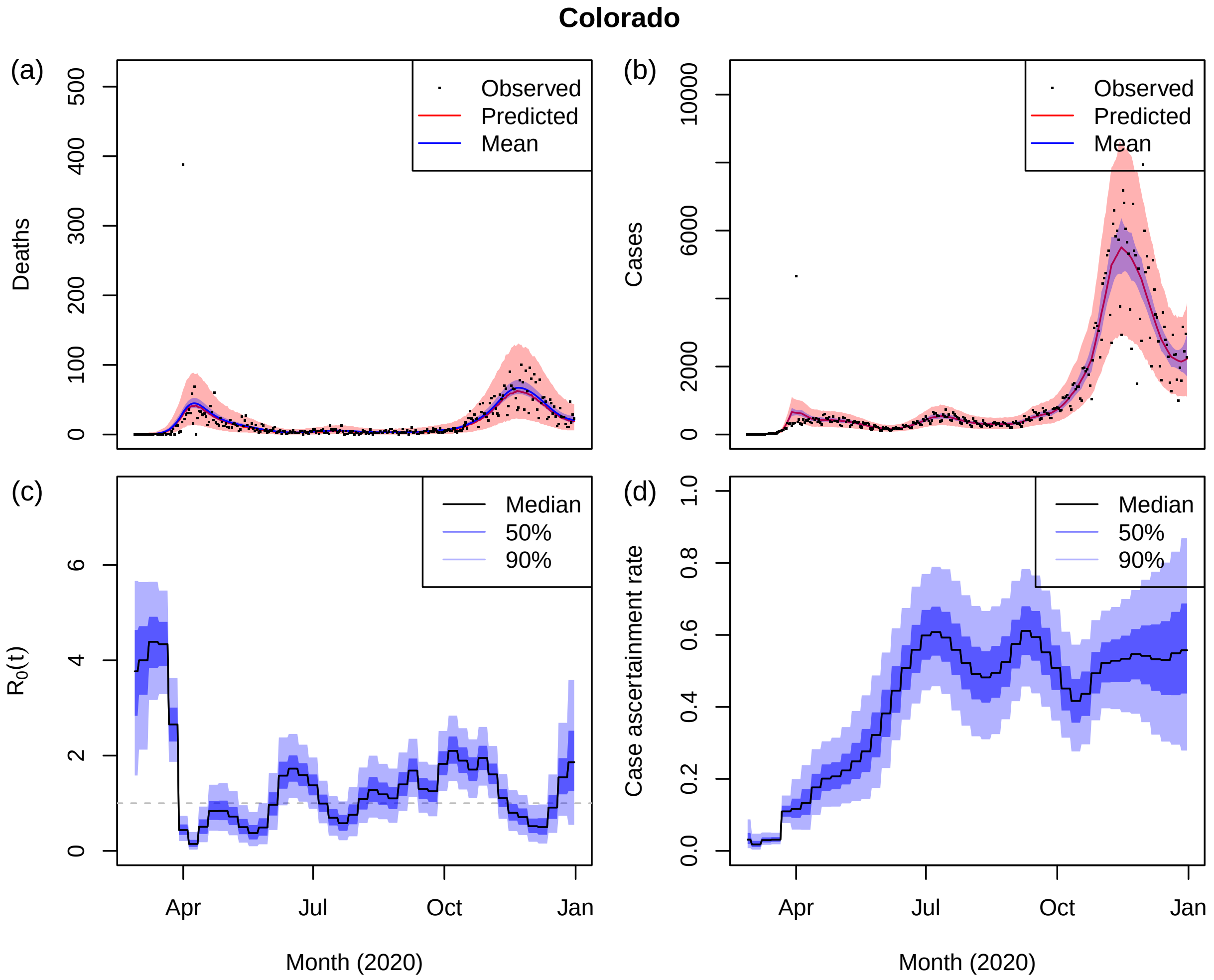

Figure S25: SEIRD model fit to COVID-19 data. **Top panels:** observed (a) deaths $d(t)$ and (b) cases $c(t)$ are plotted in black. Median and 90% credible intervals of the posterior predictive distributions of $d(t)$ and $c(t)$ are in red. Posterior median and 90% credible intervals of the underlying mean parameters $m_D(t)$ and $m_C(t)$ are in blue. **Bottom panels:** posterior median, 50%, and 90% credible intervals for (c) the basic reproduction number $R_0(t)$ and (d) the case ascertainment rate $\text{CAR}(t)$.

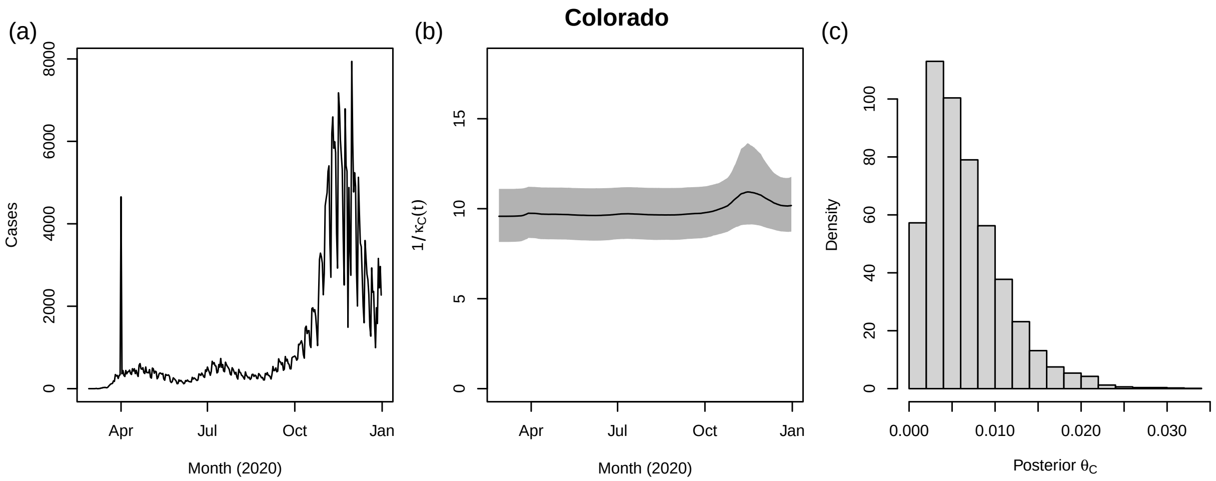

Figure S26: Overdispersion and zero-inflation in state-level clinical case data. (a) Reported cases. (b) Posterior median and 90% credible interval for the time-varying negative binomial overdispersion parameter $\kappa_C(t)^{-1}$. (c) Posterior histogram for the zero-inflation parameter θ_C.

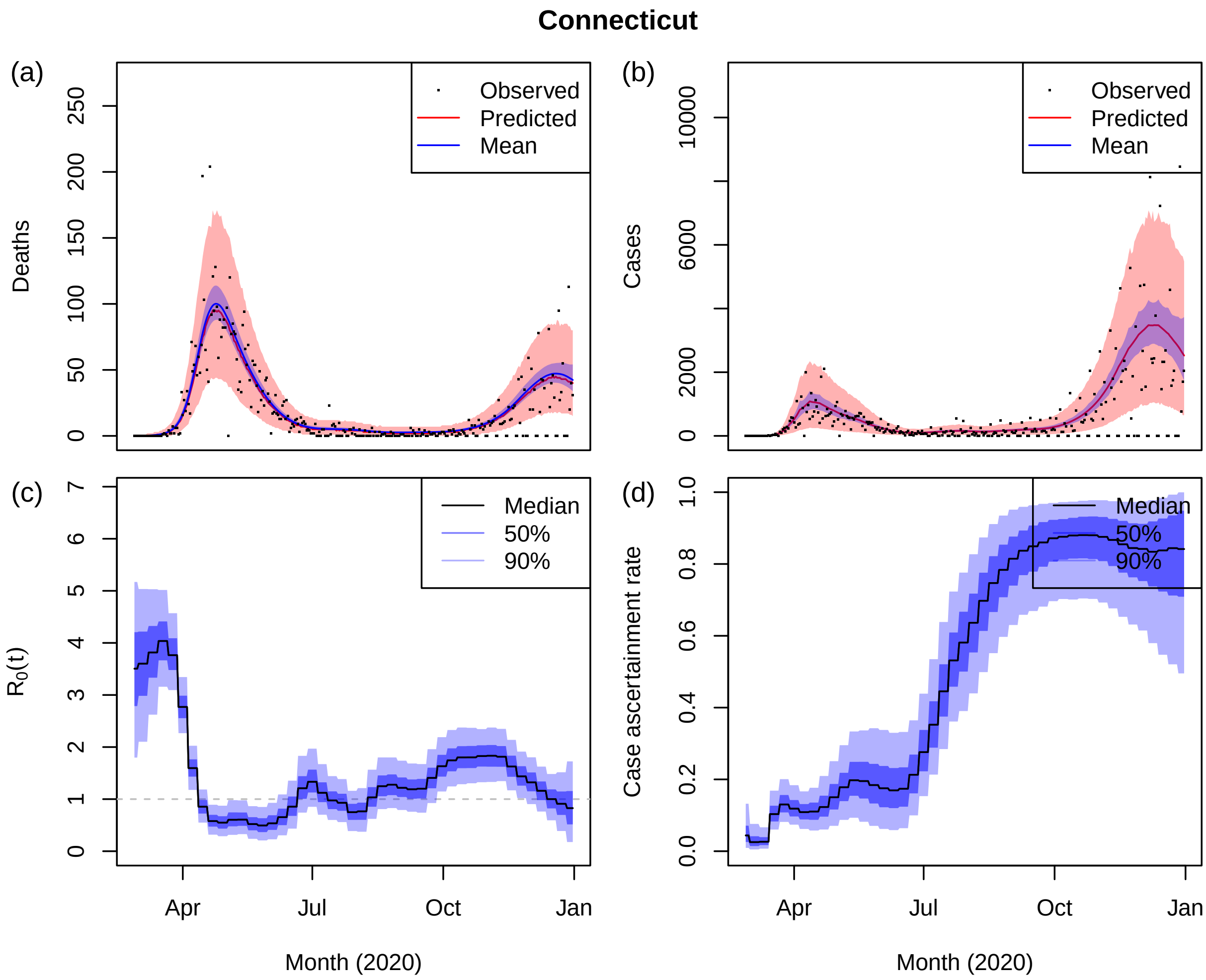

Figure S27: SEIRD model fit to COVID-19 data. **Top panels:** observed (a) deaths $d(t)$ and (b) cases $c(t)$ are plotted in black. Median and 90% credible intervals of the posterior predictive distributions of $d(t)$ and $c(t)$ are in red. Posterior median and 90% credible intervals of the underlying mean parameters $m_D(t)$ and $m_C(t)$ are in blue. **Bottom panels:** posterior median, 50%, and 90% credible intervals for (c) the basic reproduction number $R_0(t)$ and (d) the case ascertainment rate $\text{CAR}(t)$.

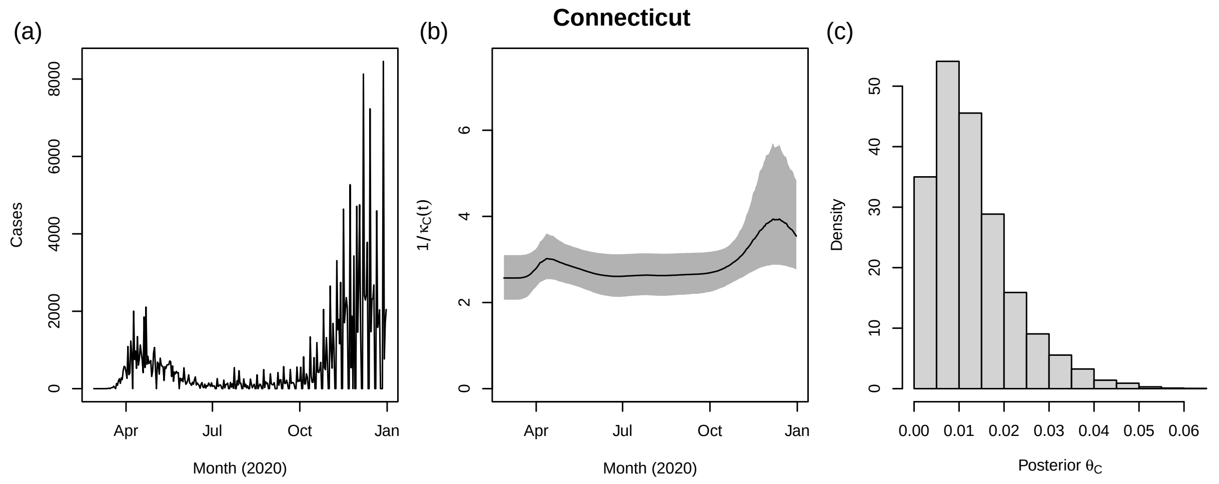

Figure S28: Overdispersion and zero-inflation in state-level clinical case data. (a) Reported cases. (b) Posterior median and 90% credible interval for the time-varying negative binomial overdispersion parameter $\kappa_C(t)^{-1}$. (c) Posterior histogram for the zero-inflation parameter θ_C.

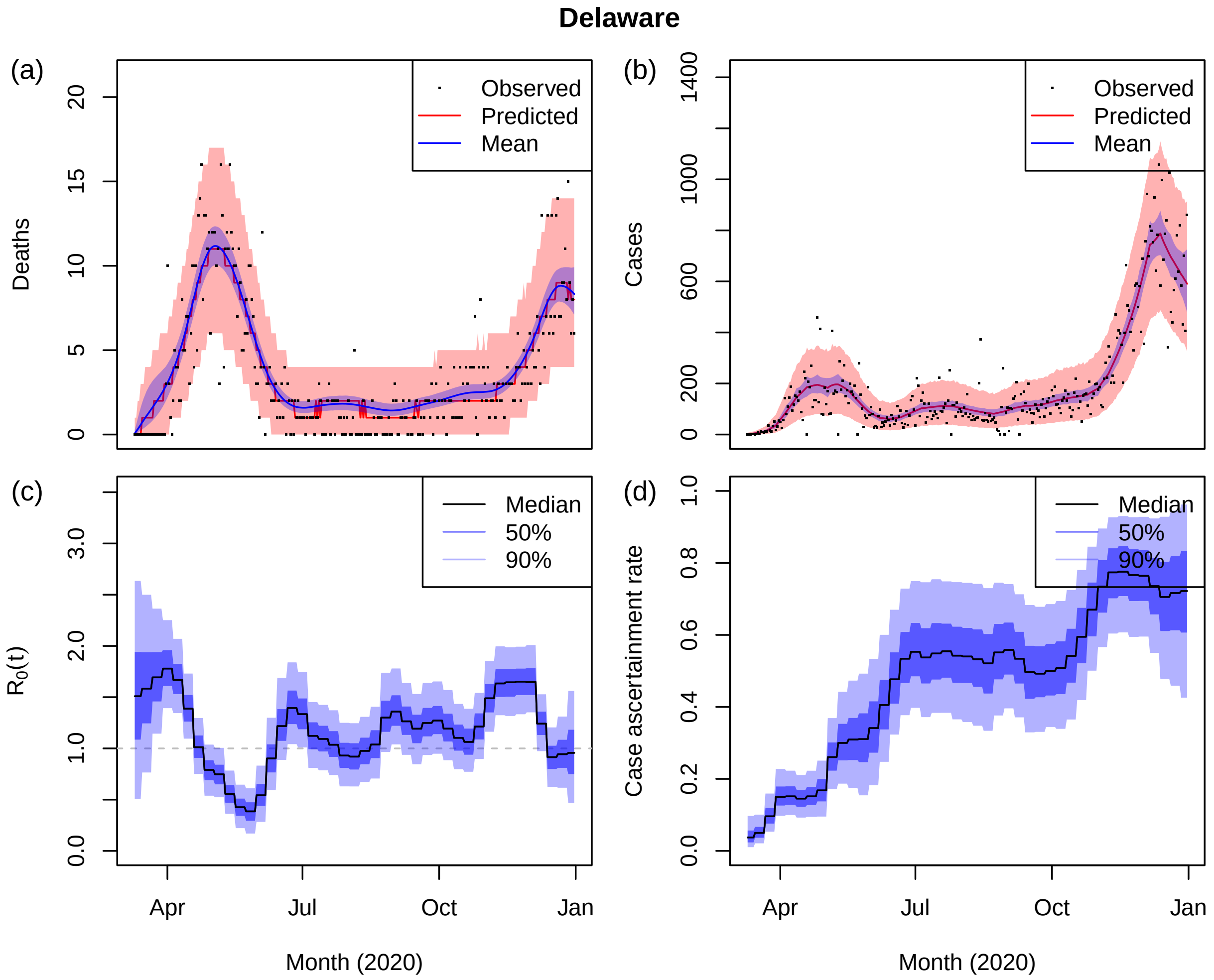

Figure S29: SEIRD model fit to COVID-19 data. **Top panels:** observed (a) deaths $d(t)$ and (b) cases $c(t)$ are plotted in black. Median and 90% credible intervals of the posterior predictive distributions of $d(t)$ and $c(t)$ are in red. Posterior median and 90% credible intervals of the underlying mean parameters $m_D(t)$ and $m_C(t)$ are in blue. **Bottom panels:** posterior median, 50%, and 90% credible intervals for (c) the basic reproduction number $R_0(t)$ and (d) the case ascertainment rate $\mathrm{CAR}(t)$.

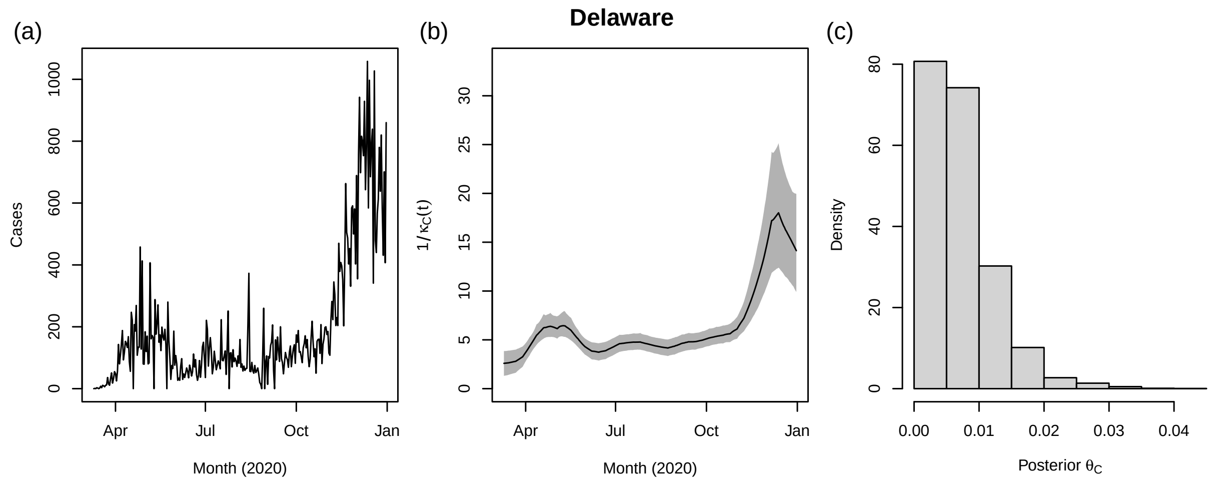

Figure S30: Overdispersion and zero-inflation in state-level clinical case data. (a) Reported cases. (b) Posterior median and 90% credible interval for the time-varying negative binomial overdispersion parameter $\kappa_C(t)^{-1}$. (c) Posterior histogram for the zero-inflation parameter θ_C.

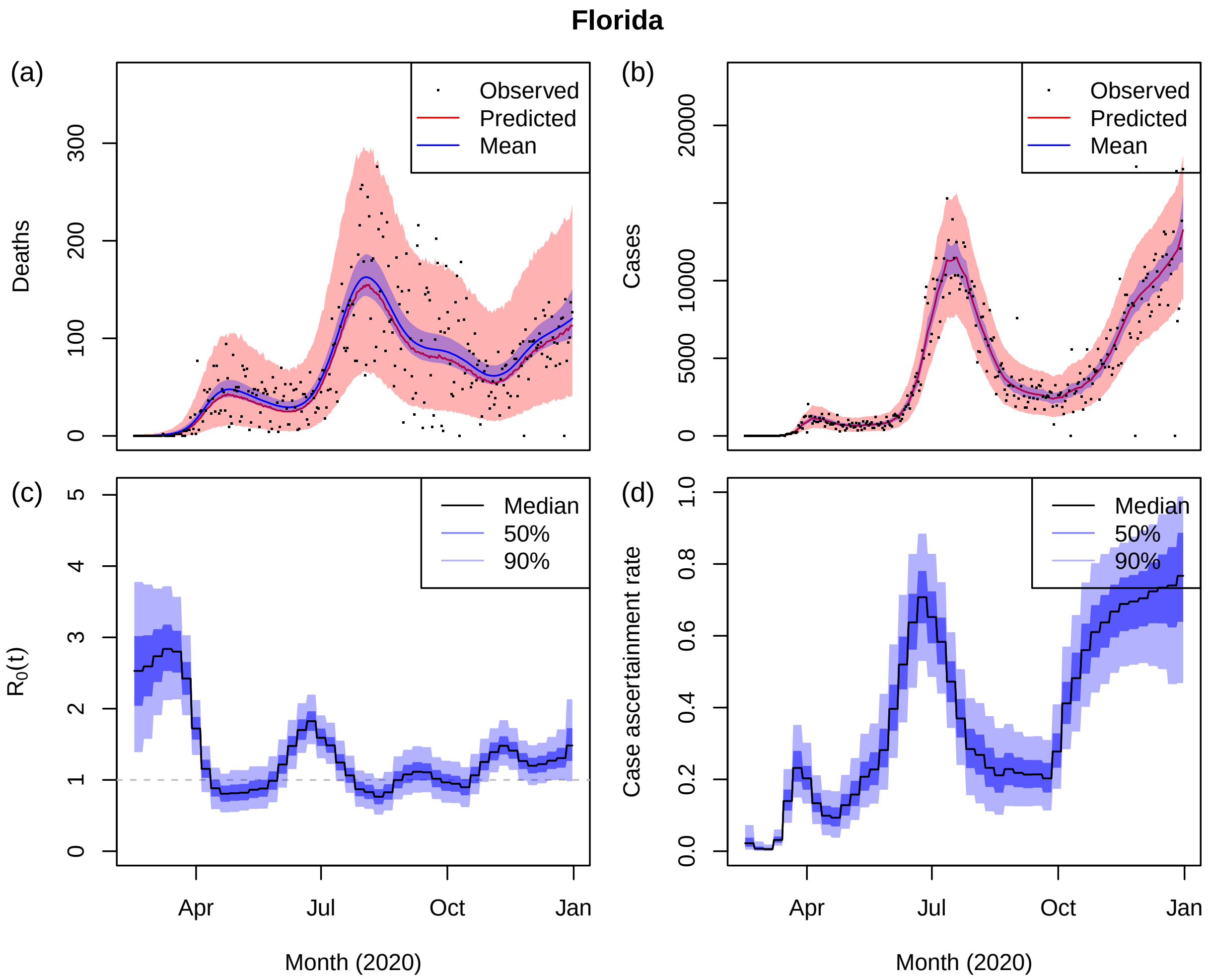

Figure S31: SEIRD model fit to COVID-19 data. **Top panels:** observed (a) deaths $d(t)$ and (b) cases $c(t)$ are plotted in black. Median and 90% credible intervals of the posterior predictive distributions of $d(t)$ and $c(t)$ are in red. Posterior median and 90% credible intervals of the underlying mean parameters $m_D(t)$ and $m_C(t)$ are in blue. **Bottom panels:** posterior median, 50%, and 90% credible intervals for (c) the basic reproduction number $R_0(t)$ and (d) the case ascertainment rate CAR(t).

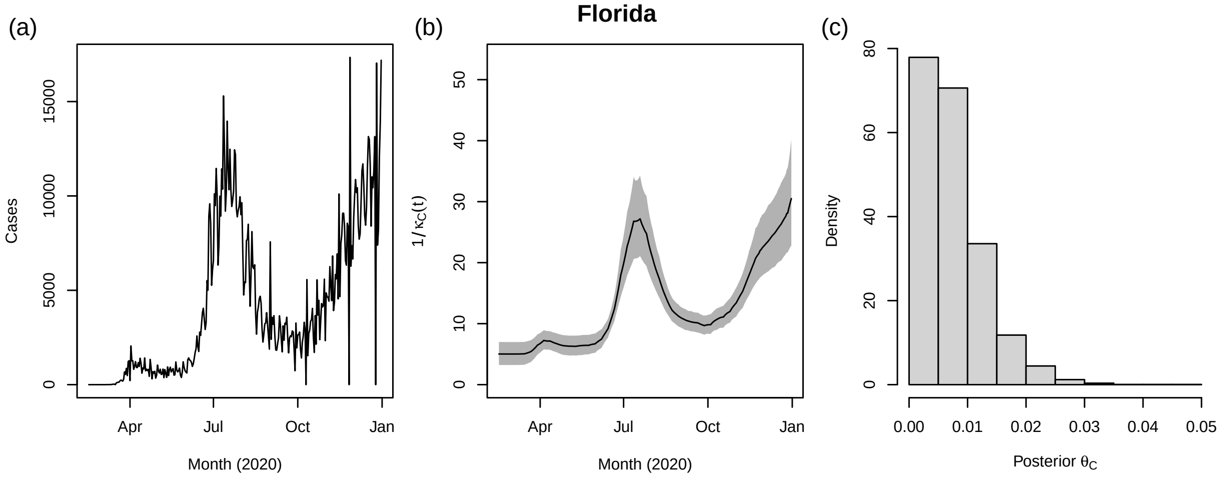

Figure S32: Overdispersion and zero-inflation in state-level clinical case data. (a) Reported cases. (b) Posterior median and 90% credible interval for the time-varying negative binomial overdispersion parameter $\kappa_C(t)^{-1}$. (c) Posterior histogram for the zero-inflation parameter θ_C.

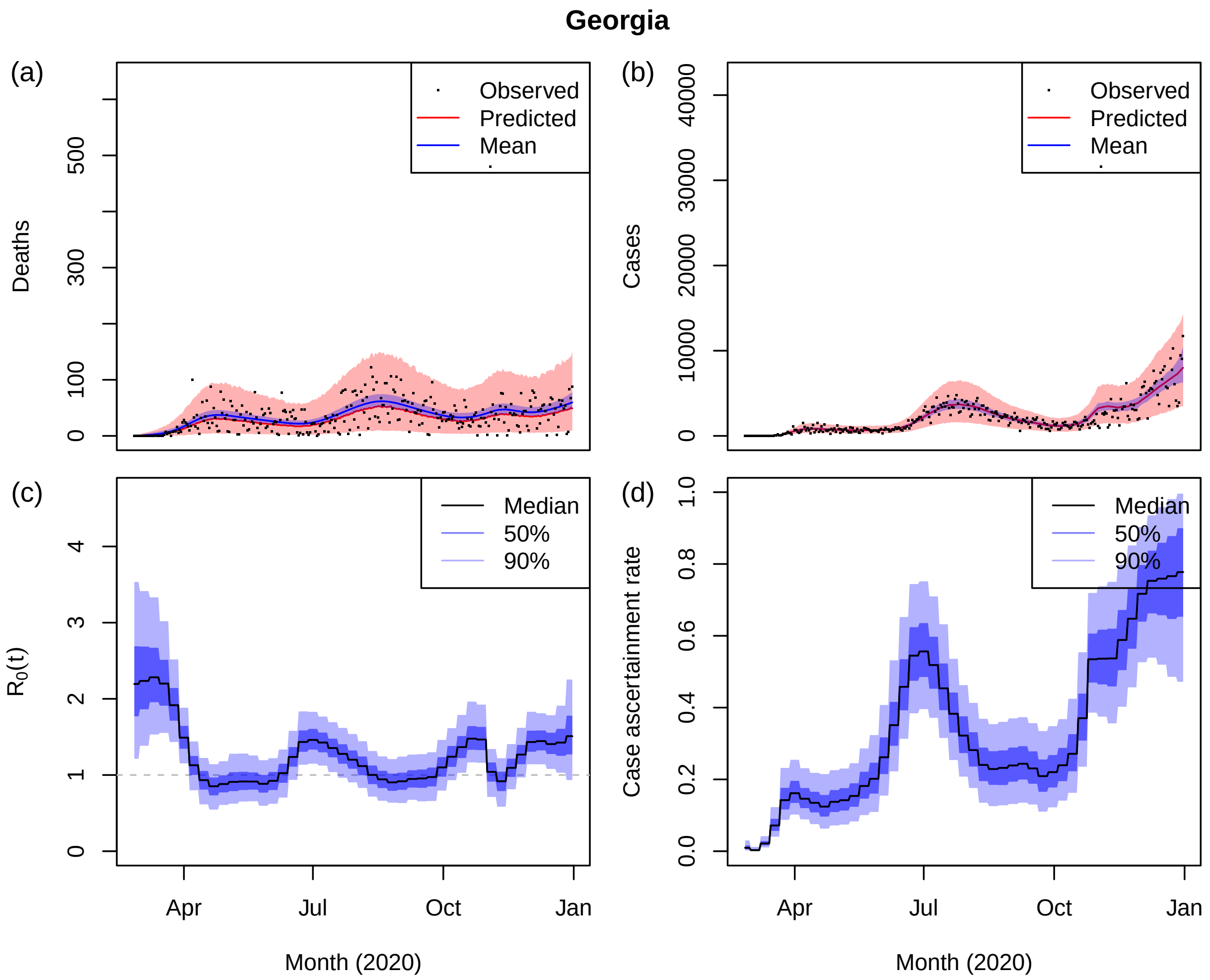

Figure S33: SEIRD model fit to COVID-19 data. **Top panels:** observed (a) deaths $d(t)$ and (b) cases $c(t)$ are plotted in black. Median and 90% credible intervals of the posterior predictive distributions of $d(t)$ and $c(t)$ are in red. Posterior median and 90% credible intervals of the underlying mean parameters $m_D(t)$ and $m_C(t)$ are in blue. **Bottom panels:** posterior median, 50%, and 90% credible intervals for (c) the basic reproduction number $R_0(t)$ and (d) the case ascertainment rate $\text{CAR}(t)$.

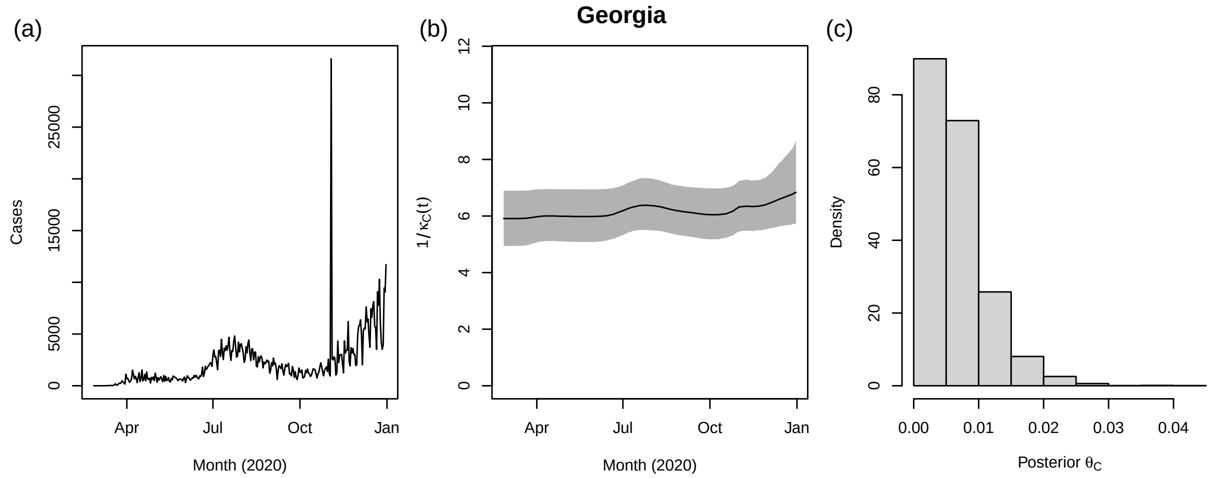

Figure S34: Overdispersion and zero-inflation in state-level clinical case data. (a) Reported cases. (b) Posterior median and 90% credible interval for the time-varying negative binomial overdispersion parameter $\kappa_C(t)^{-1}$. (c) Posterior histogram for the zero-inflation parameter θ_C.

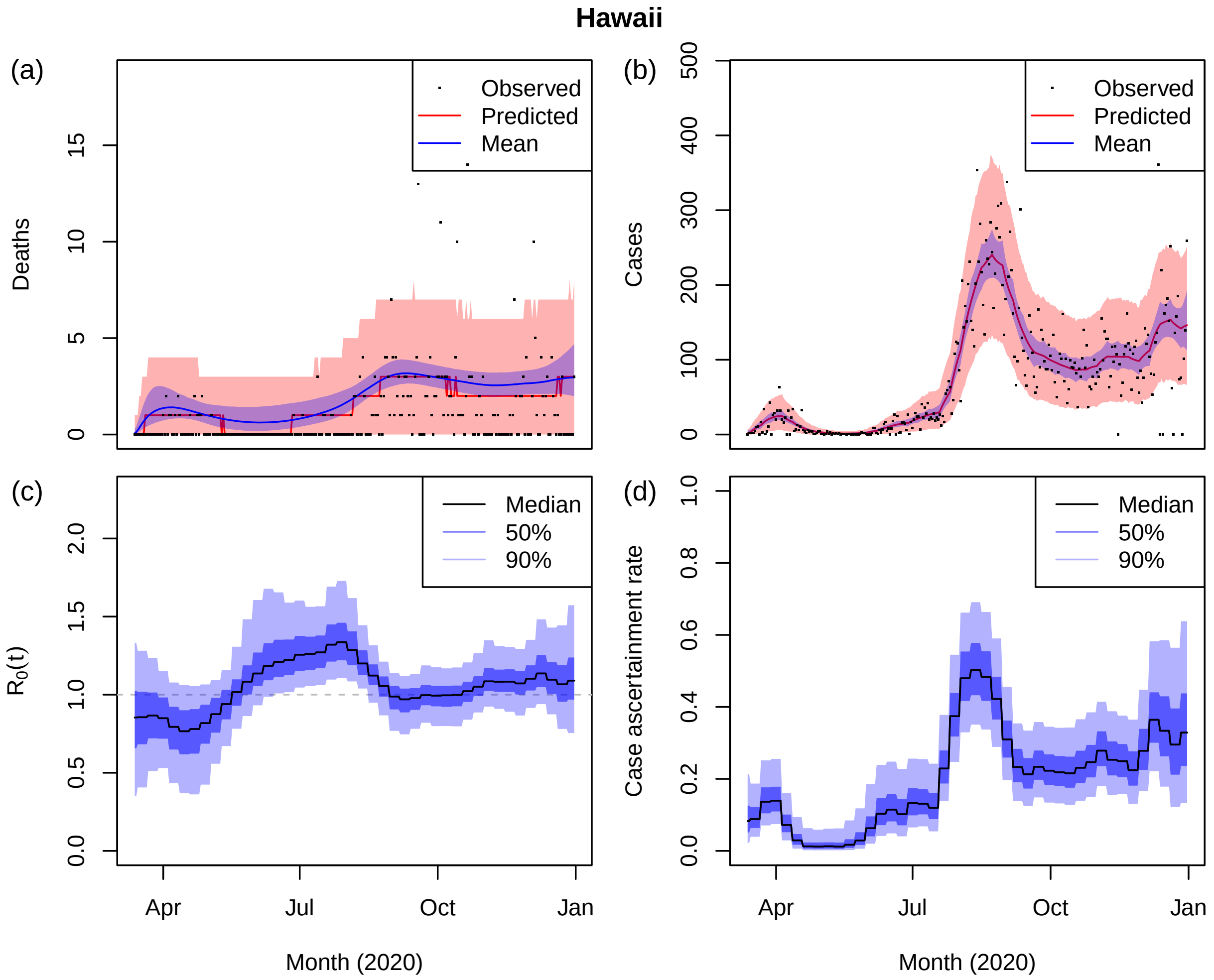

Figure S35: SEIRD model fit to COVID-19 data. **Top panels:** observed (a) deaths $d(t)$ and (b) cases $c(t)$ are plotted in black. Median and 90% credible intervals of the posterior predictive distributions of $d(t)$ and $c(t)$ are in red. Posterior median and 90% credible intervals of the underlying mean parameters $m_D(t)$ and $m_C(t)$ are in blue. **Bottom panels:** posterior median, 50%, and 90% credible intervals for (c) the basic reproduction number $R_0(t)$ and (d) the case ascertainment rate $\mathrm{CAR}(t)$.

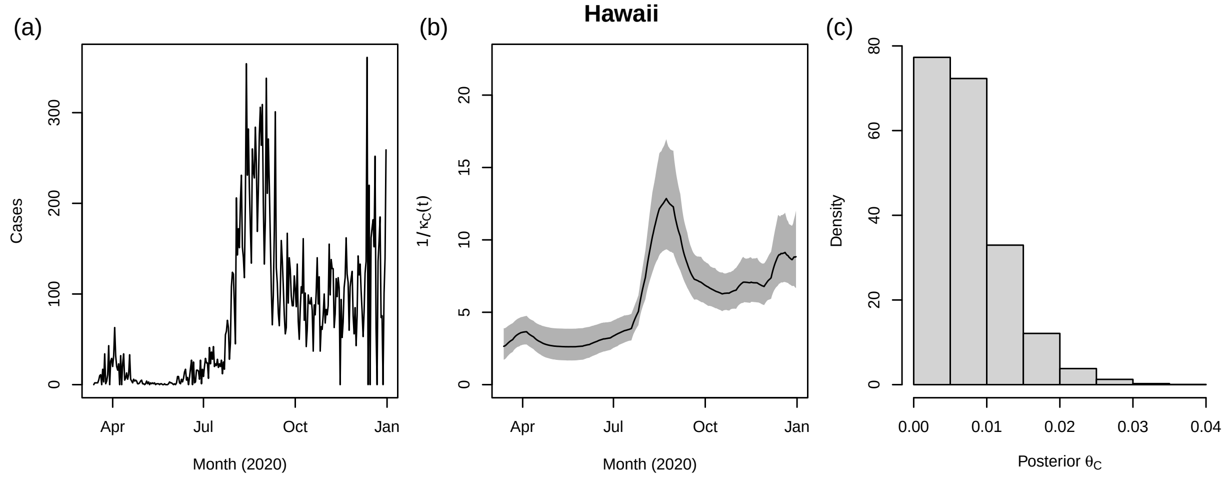

Figure S36: Overdispersion and zero-inflation in state-level clinical case data. (a) Reported cases. (b) Posterior median and 90% credible interval for the time-varying negative binomial overdispersion parameter $\kappa_C(t)^{-1}$. (c) Posterior histogram for the zero-inflation parameter θ_C.

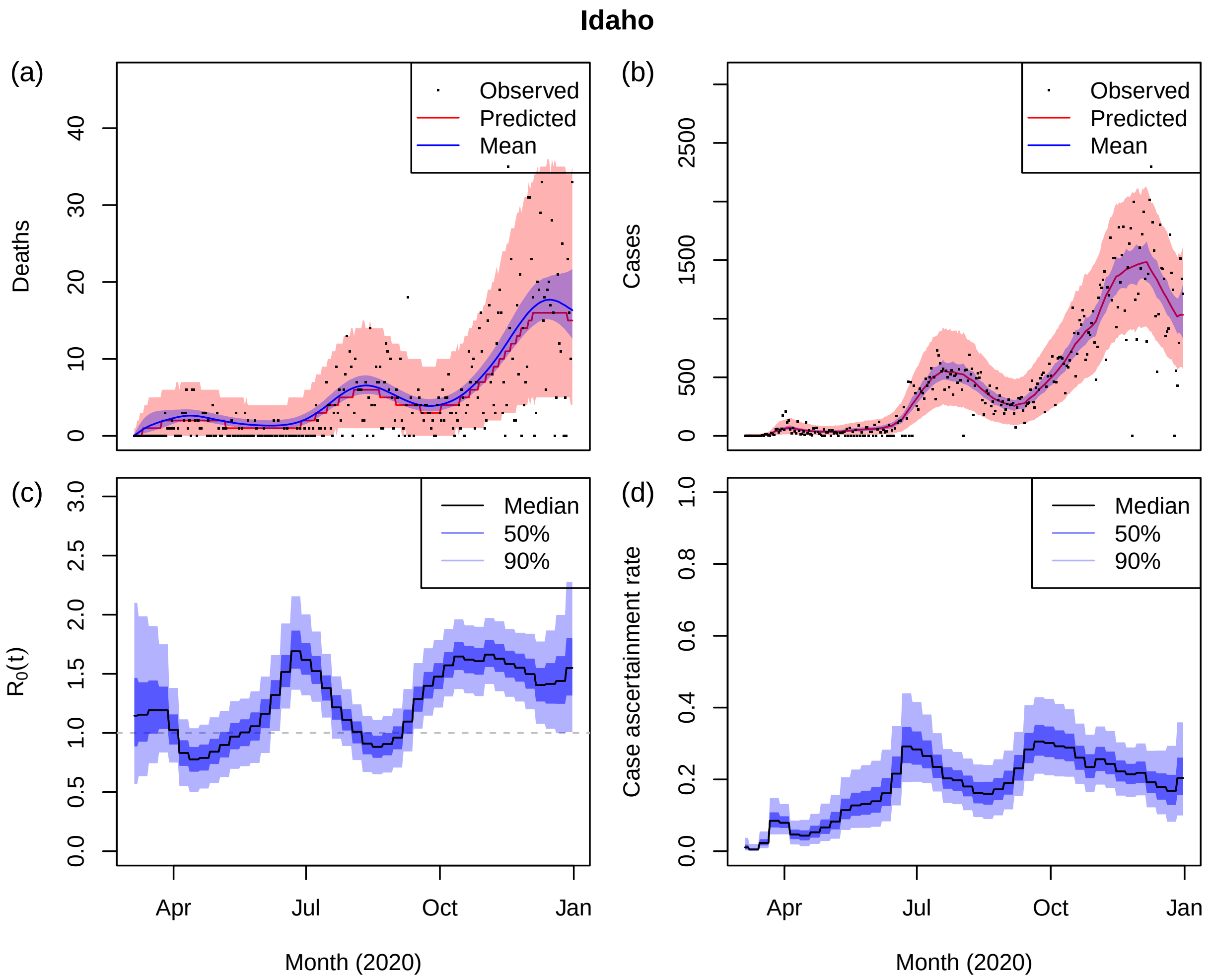

Figure S37: SEIRD model fit to COVID-19 data. **Top panels:** observed (a) deaths $d(t)$ and (b) cases $c(t)$ are plotted in black. Median and 90% credible intervals of the posterior predictive distributions of $d(t)$ and $c(t)$ are in red. Posterior median and 90% credible intervals of the underlying mean parameters $m_D(t)$ and $m_C(t)$ are in blue. **Bottom panels:** posterior median, 50%, and 90% credible intervals for (c) the basic reproduction number $R_0(t)$ and (d) the case ascertainment rate CAR(t).

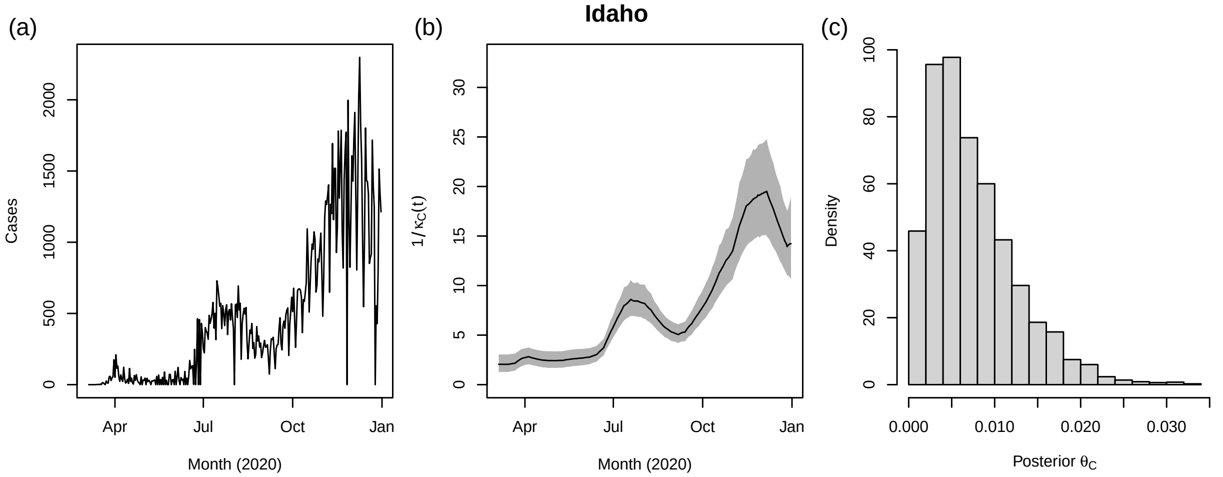

Figure S38: Overdispersion and zero-inflation in state-level clinical case data. (a) Reported cases. (b) Posterior median and 90% credible interval for the time-varying negative binomial overdispersion parameter $\kappa_C(t)^{-1}$. (c) Posterior histogram for the zero-inflation parameter θ_C.

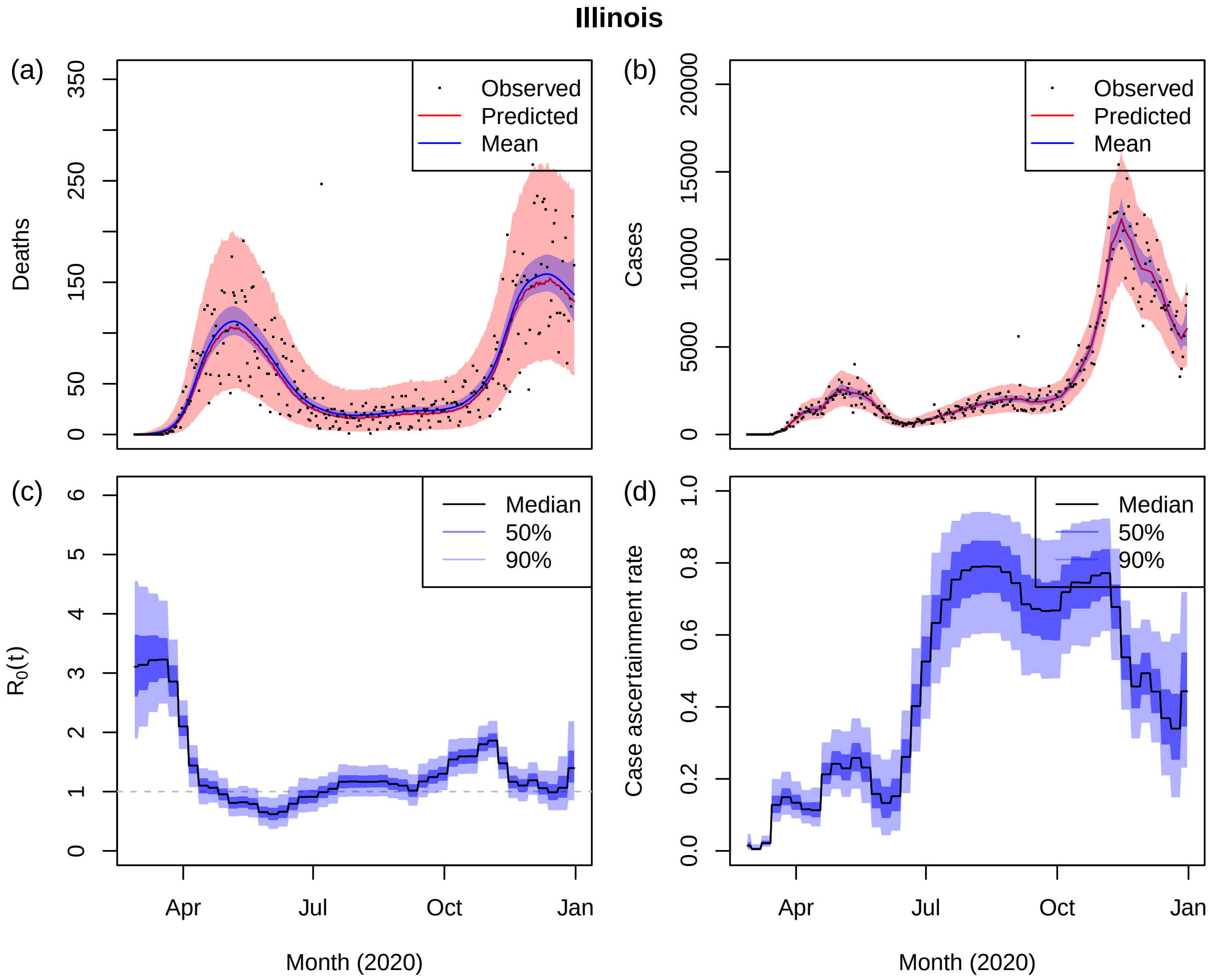

Figure S39: SEIRD model fit to COVID-19 data. **Top panels:** observed (a) deaths $d(t)$ and (b) cases $c(t)$ are plotted in black. Median and 90% credible intervals of the posterior predictive distributions of $d(t)$ and $c(t)$ are in red. Posterior median and 90% credible intervals of the underlying mean parameters $m_D(t)$ and $m_C(t)$ are in blue. **Bottom panels:** posterior median, 50%, and 90% credible intervals for (c) the basic reproduction number $R_0(t)$ and (d) the case ascertainment rate $\mathrm{CAR}(t)$.

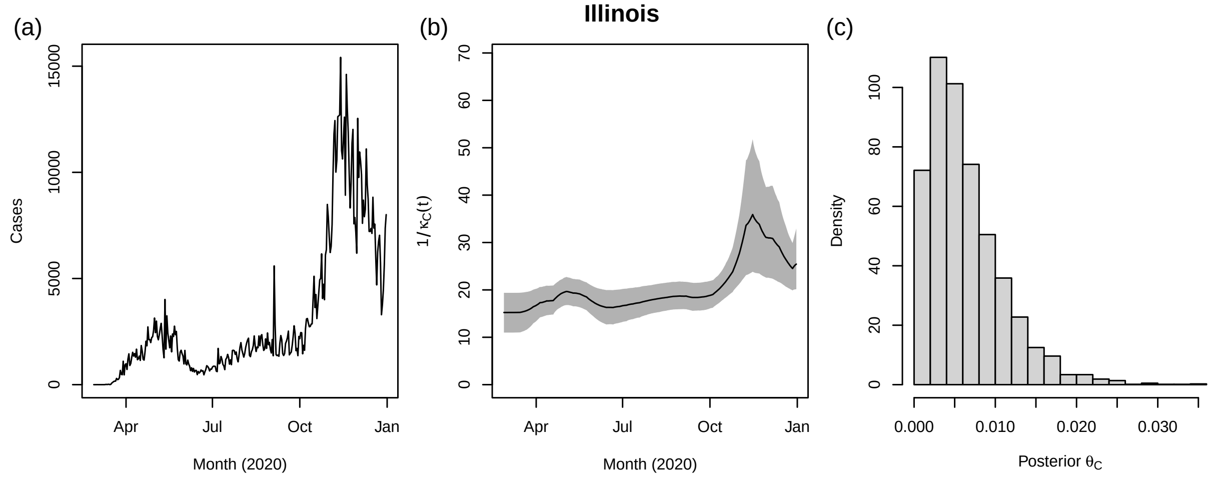

Figure S40: Overdispersion and zero-inflation in state-level clinical case data. (a) Reported cases. (b) Posterior median and 90% credible interval for the time-varying negative binomial overdispersion parameter $\kappa_C(t)^{-1}$. (c) Posterior histogram for the zero-inflation parameter θ_C.

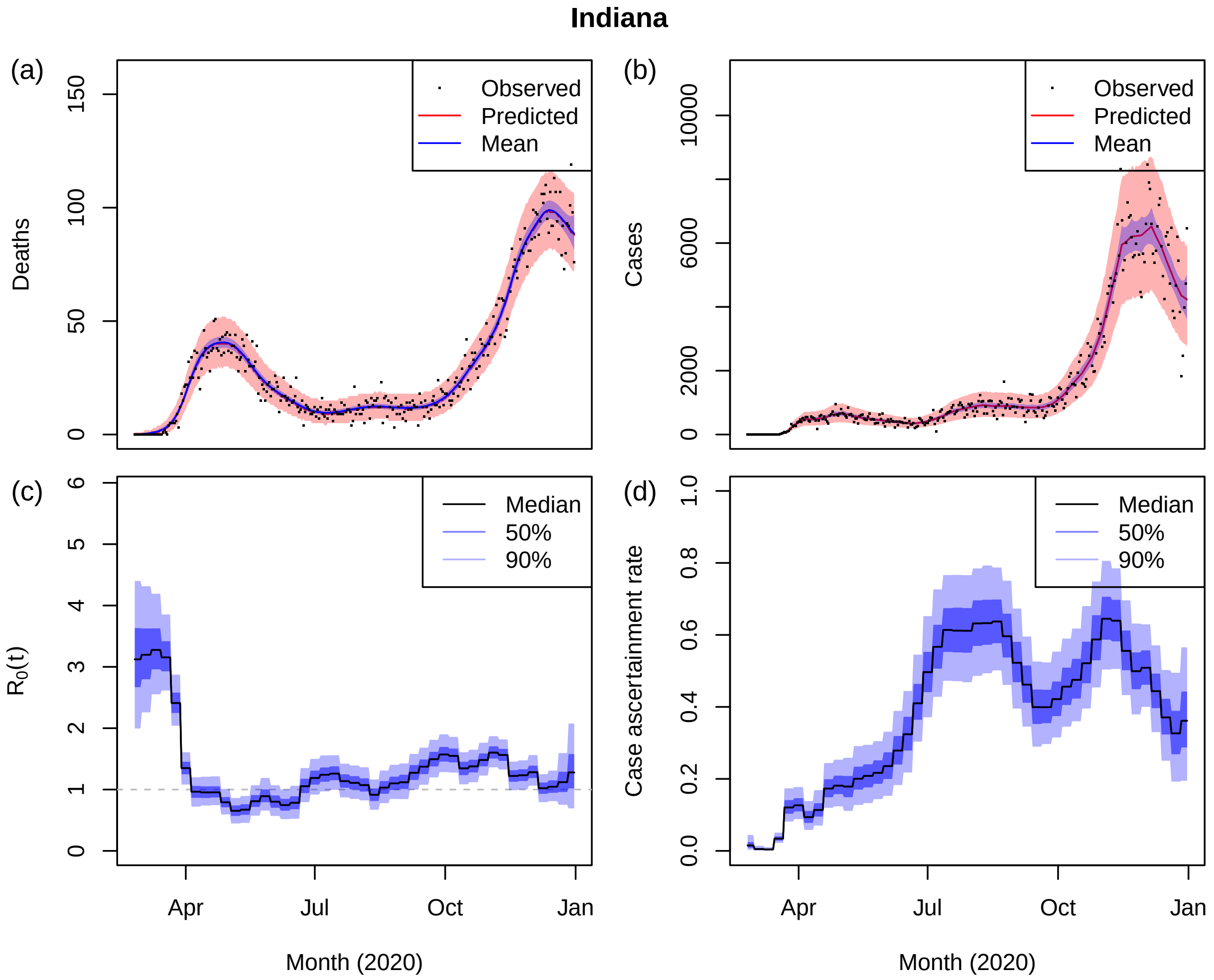

Figure S41: SEIRD model fit to COVID-19 data. **Top panels:** observed (a) deaths $d(t)$ and (b) cases $c(t)$ are plotted in black. Median and 90% credible intervals of the posterior predictive distributions of $d(t)$ and $c(t)$ are in red. Posterior median and 90% credible intervals of the underlying mean parameters $m_D(t)$ and $m_C(t)$ are in blue. **Bottom panels:** posterior median, 50%, and 90% credible intervals for (c) the basic reproduction number $R_0(t)$ and (d) the case ascertainment rate CAR(t).

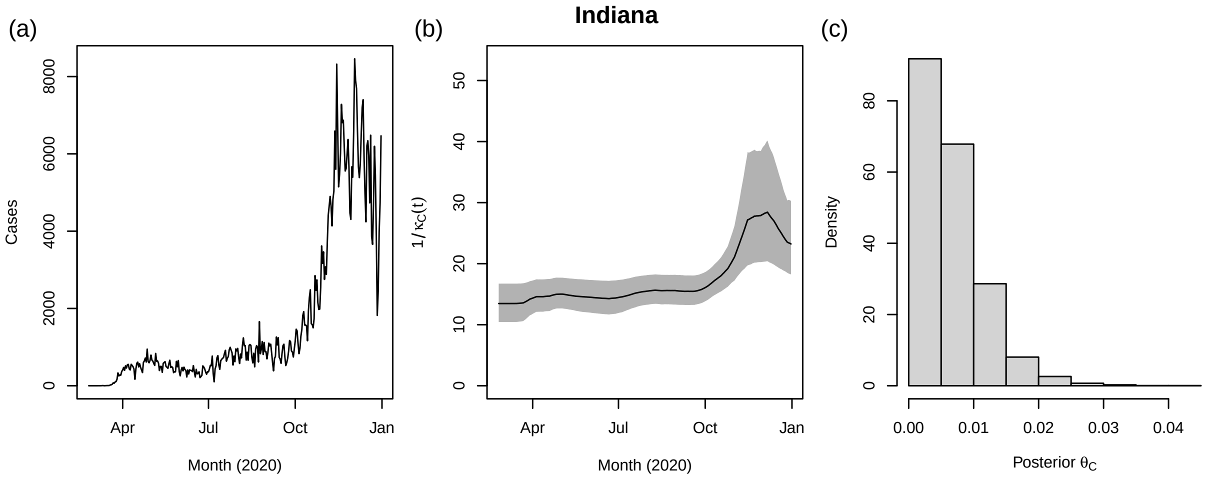

Figure S42: Overdispersion and zero-inflation in state-level clinical case data. (a) Reported cases. (b) Posterior median and 90% credible interval for the time-varying negative binomial overdispersion parameter $\kappa_C(t)^{-1}$. (c) Posterior histogram for the zero-inflation parameter θ_C.

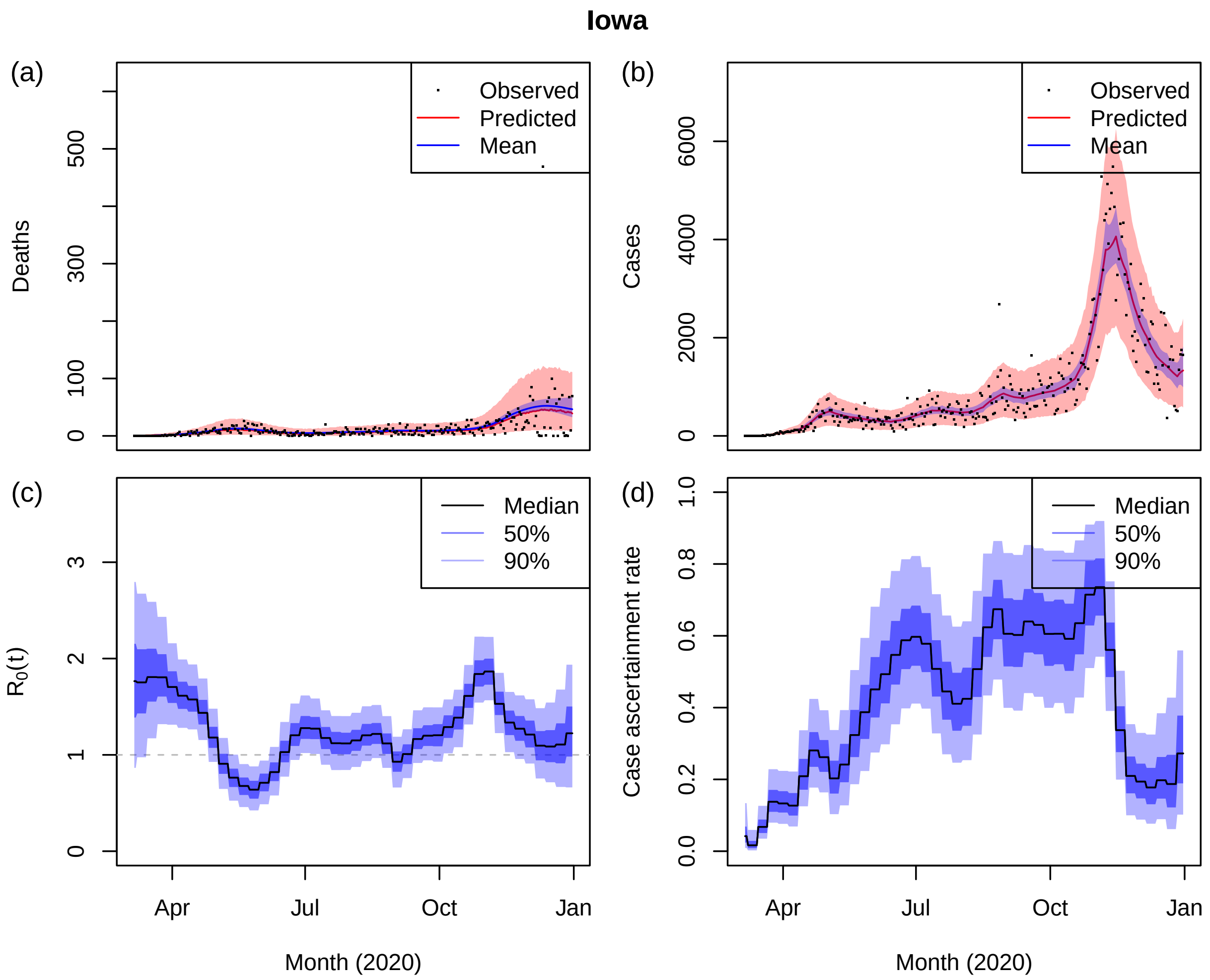

Figure S43: SEIRD model fit to COVID-19 data. **Top panels:** observed (a) deaths $d(t)$ and (b) cases $c(t)$ are plotted in black. Median and 90% credible intervals of the posterior predictive distributions of $d(t)$ and $c(t)$ are in red. Posterior median and 90% credible intervals of the underlying mean parameters $m_D(t)$ and $m_C(t)$ are in blue. **Bottom panels:** posterior median, 50%, and 90% credible intervals for (c) the basic reproduction number $R_0(t)$ and (d) the case ascertainment rate $\mathrm{CAR}(t)$.

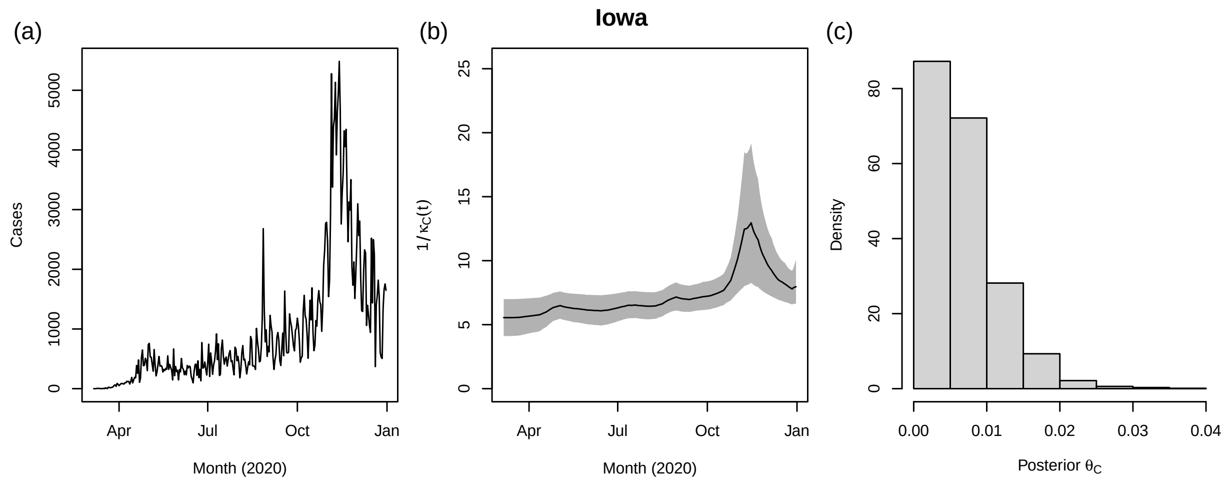

Figure S44: Overdispersion and zero-inflation in state-level clinical case data. (a) Reported cases. (b) Posterior median and 90% credible interval for the time-varying negative binomial overdispersion parameter $\kappa_C(t)^{-1}$. (c) Posterior histogram for the zero-inflation parameter θ_C.

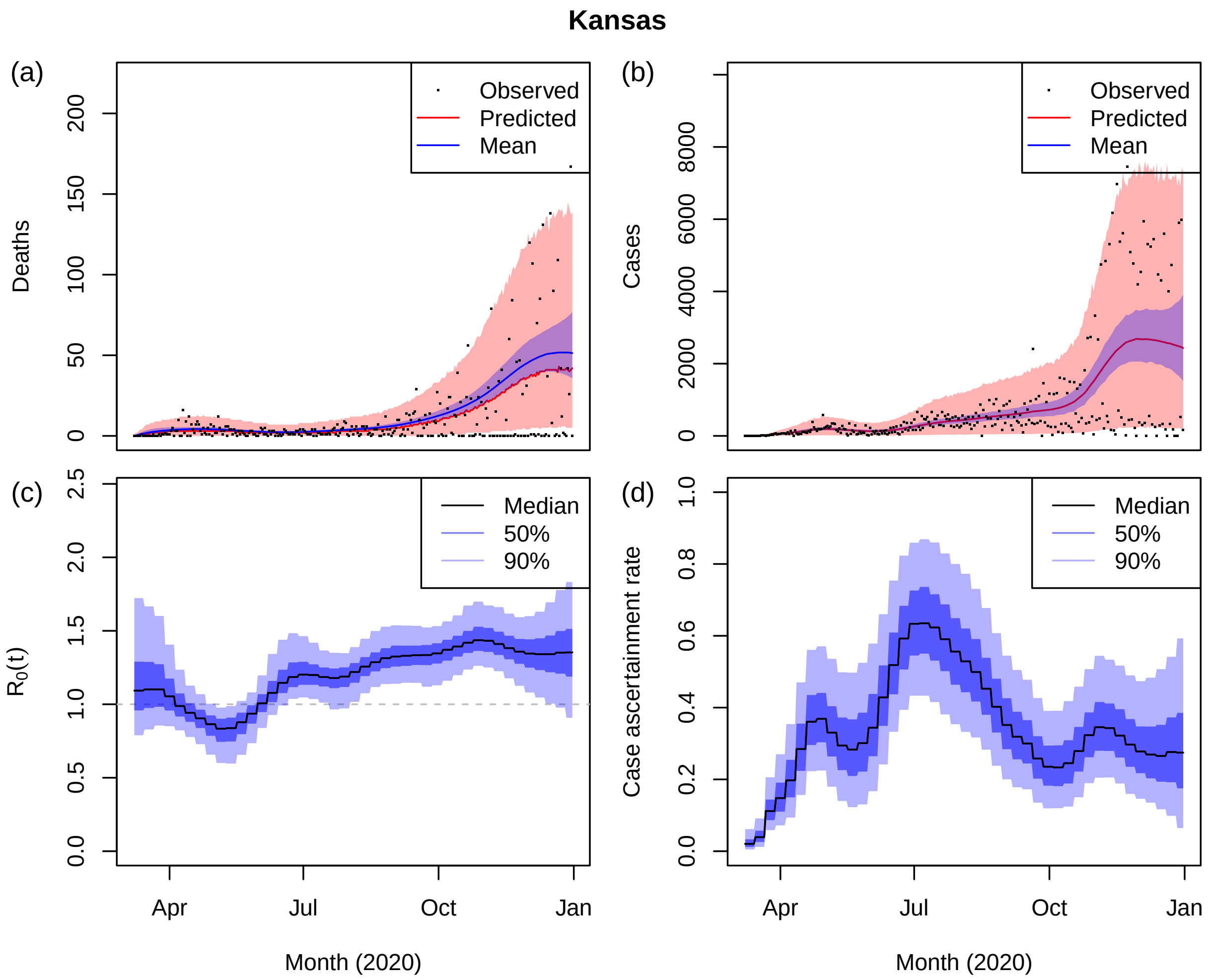

Figure S45: SEIRD model fit to COVID-19 data. **Top panels:** observed (a) deaths $d(t)$ and (b) cases $c(t)$ are plotted in black. Median and 90% credible intervals of the posterior predictive distributions of $d(t)$ and $c(t)$ are in red. Posterior median and 90% credible intervals of the underlying mean parameters $m_D(t)$ and $m_C(t)$ are in blue. **Bottom panels:** posterior median, 50%, and 90% credible intervals for (c) the basic reproduction number $R_0(t)$ and (d) the case ascertainment rate CAR(t).

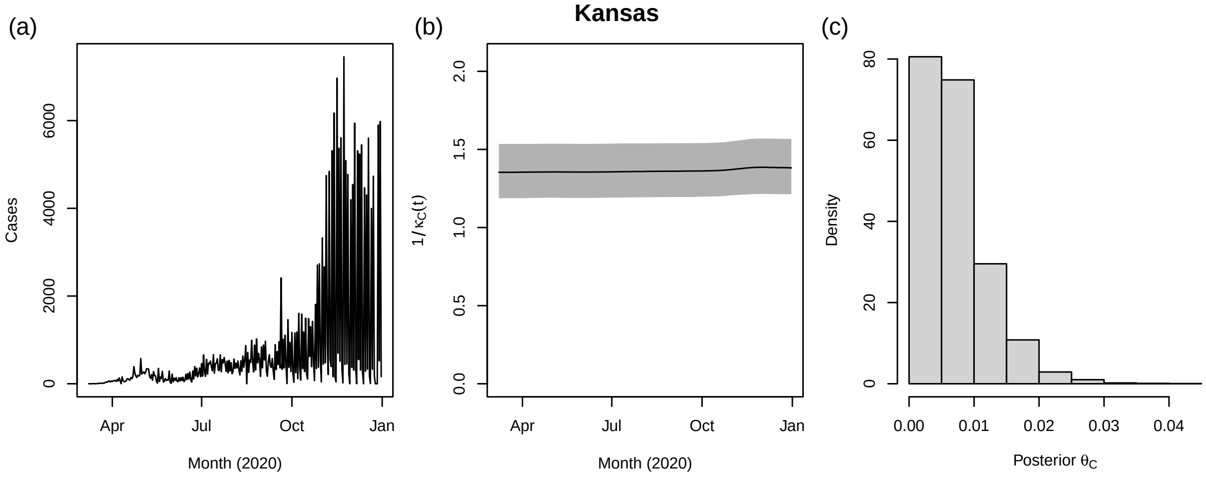

Figure S46: Overdispersion and zero-inflation in state-level clinical case data. (a) Reported cases. (b) Posterior median and 90% credible interval for the time-varying negative binomial overdispersion parameter $\kappa_C(t)^{-1}$. (c) Posterior histogram for the zero-inflation parameter θ_C.

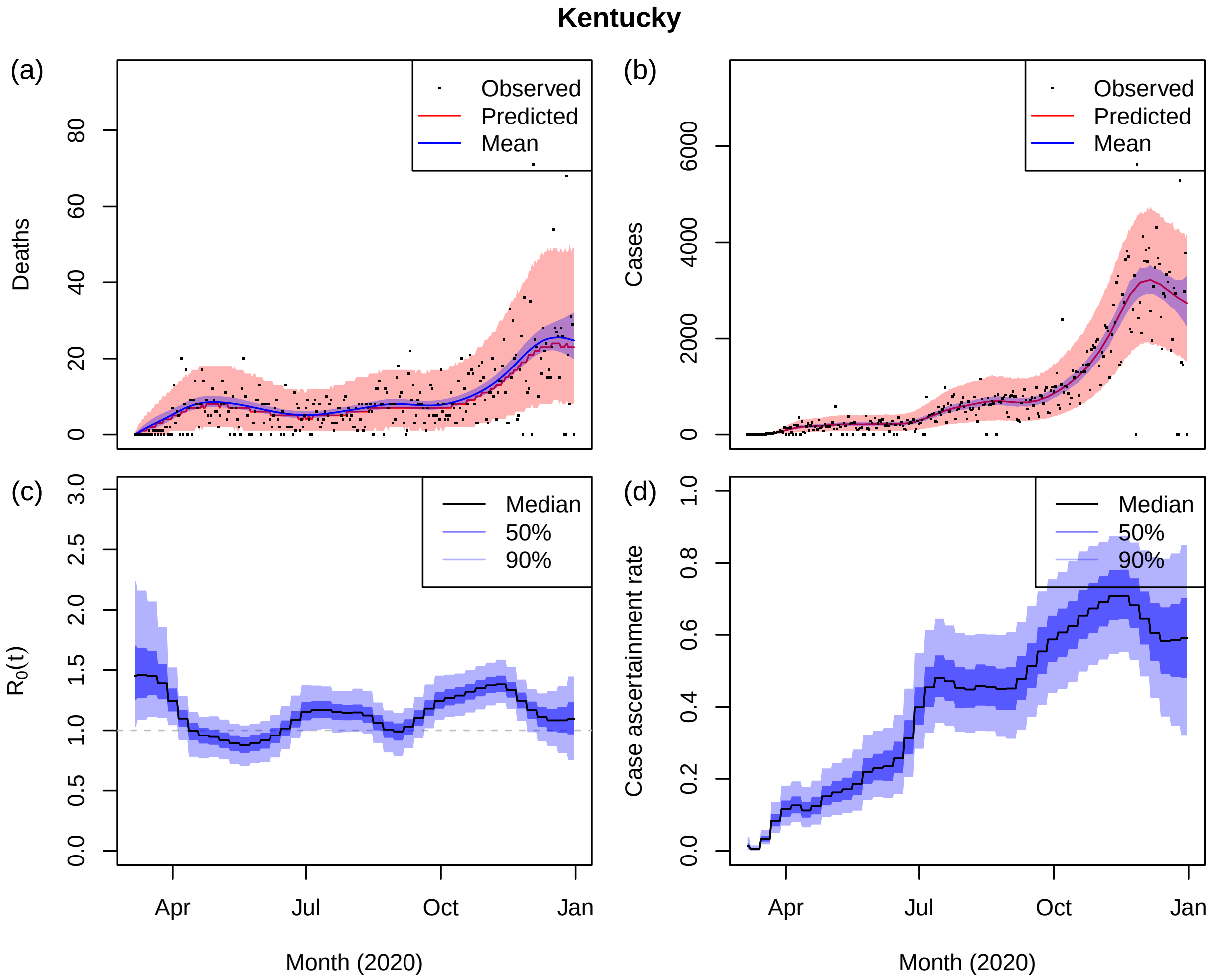

Figure S47: SEIRD model fit to COVID-19 data. **Top panels:** observed (a) deaths $d(t)$ and (b) cases $c(t)$ are plotted in black. Median and 90% credible intervals of the posterior predictive distributions of $d(t)$ and $c(t)$ are in red. Posterior median and 90% credible intervals of the underlying mean parameters $m_D(t)$ and $m_C(t)$ are in blue. **Bottom panels:** posterior median, 50%, and 90% credible intervals for (c) the basic reproduction number $R_0(t)$ and (d) the case ascertainment rate CAR(t).

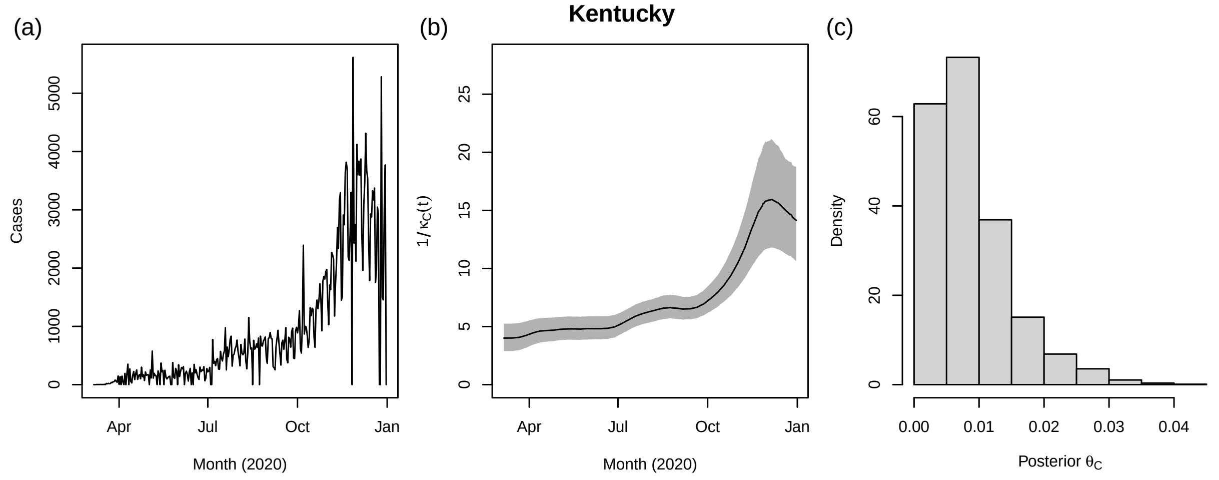

Figure S48: Overdispersion and zero-inflation in state-level clinical case data. (a) Reported cases. (b) Posterior median and 90% credible interval for the time-varying negative binomial overdispersion parameter $\kappa_C(t)^{-1}$. (c) Posterior histogram for the zero-inflation parameter θ_C.

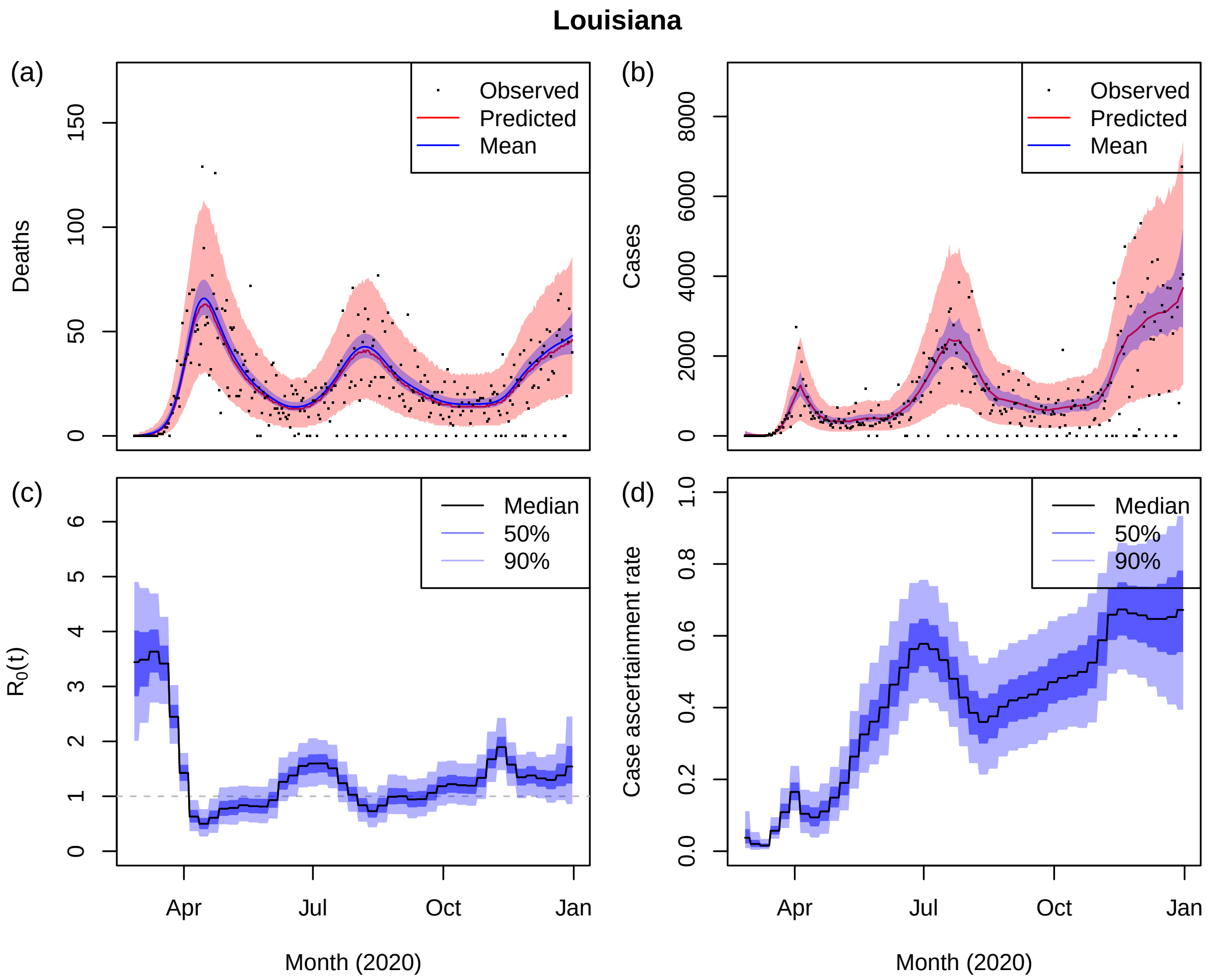

Figure S49: SEIRD model fit to COVID-19 data. **Top panels:** observed (a) deaths $d(t)$ and (b) cases $c(t)$ are plotted in black. Median and 90% credible intervals of the posterior predictive distributions of $d(t)$ and $c(t)$ are in red. Posterior median and 90% credible intervals of the underlying mean parameters $m_D(t)$ and $m_C(t)$ are in blue. **Bottom panels:** posterior median, 50%, and 90% credible intervals for (c) the basic reproduction number $R_0(t)$ and (d) the case ascertainment rate $\text{CAR}(t)$.

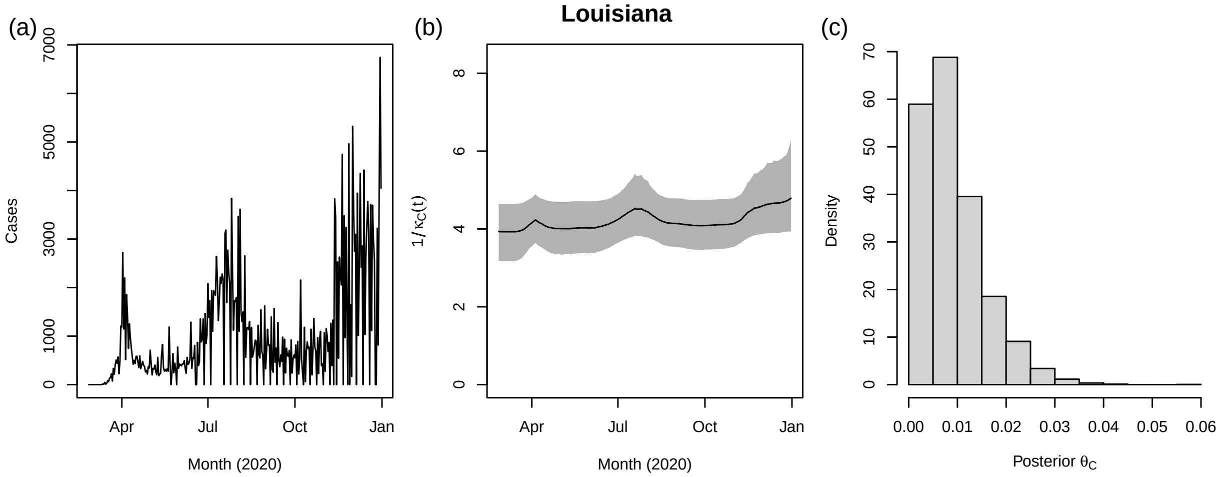

Figure S50: Overdispersion and zero-inflation in state-level clinical case data. (a) Reported cases. (b) Posterior median and 90% credible interval for the time-varying negative binomial overdispersion parameter $\kappa_C(t)^{-1}$. (c) Posterior histogram for the zero-inflation parameter θ_C.

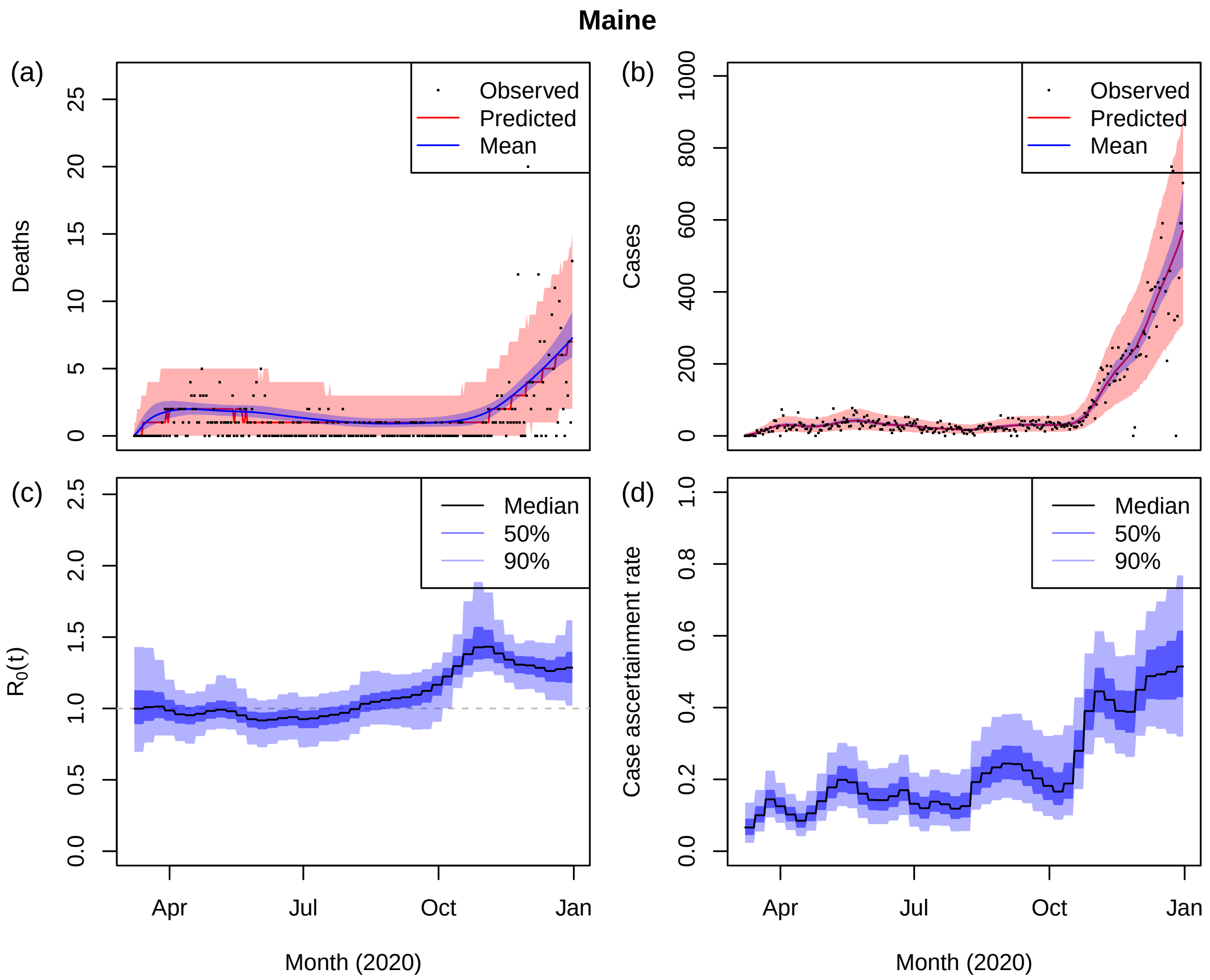

Figure S51: SEIRD model fit to COVID-19 data. **Top panels:** observed (a) deaths $d(t)$ and (b) cases $c(t)$ are plotted in black. Median and 90% credible intervals of the posterior predictive distributions of $d(t)$ and $c(t)$ are in red. Posterior median and 90% credible intervals of the underlying mean parameters $m_D(t)$ and $m_C(t)$ are in blue. **Bottom panels:** posterior median, 50%, and 90% credible intervals for (c) the basic reproduction number $R_0(t)$ and (d) the case ascertainment rate $\text{CAR}(t)$.

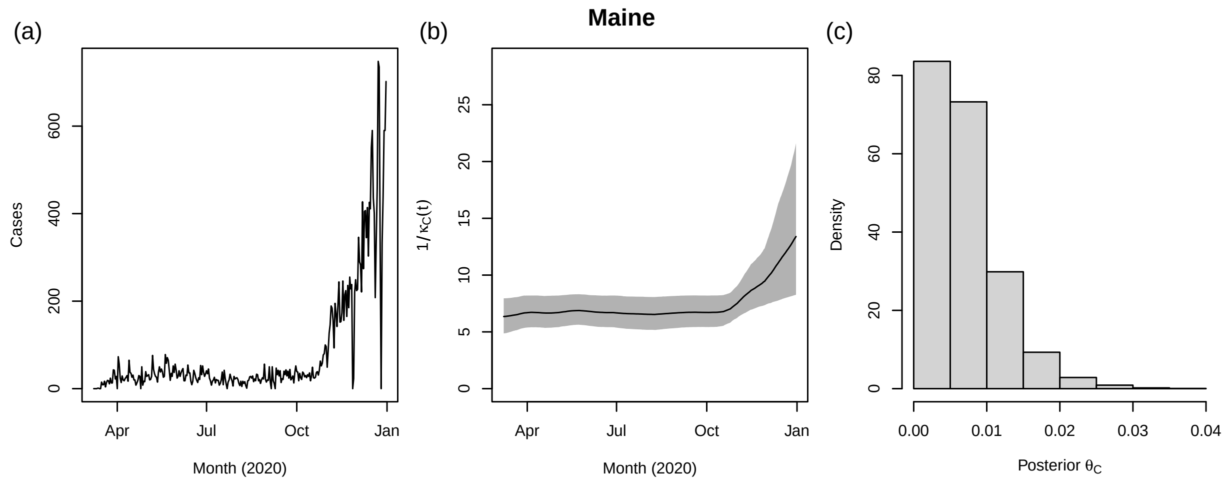

Figure S52: Overdispersion and zero-inflation in state-level clinical case data. (a) Reported cases. (b) Posterior median and 90% credible interval for the time-varying negative binomial overdispersion parameter $\kappa_C(t)^{-1}$. (c) Posterior histogram for the zero-inflation parameter θ_C.

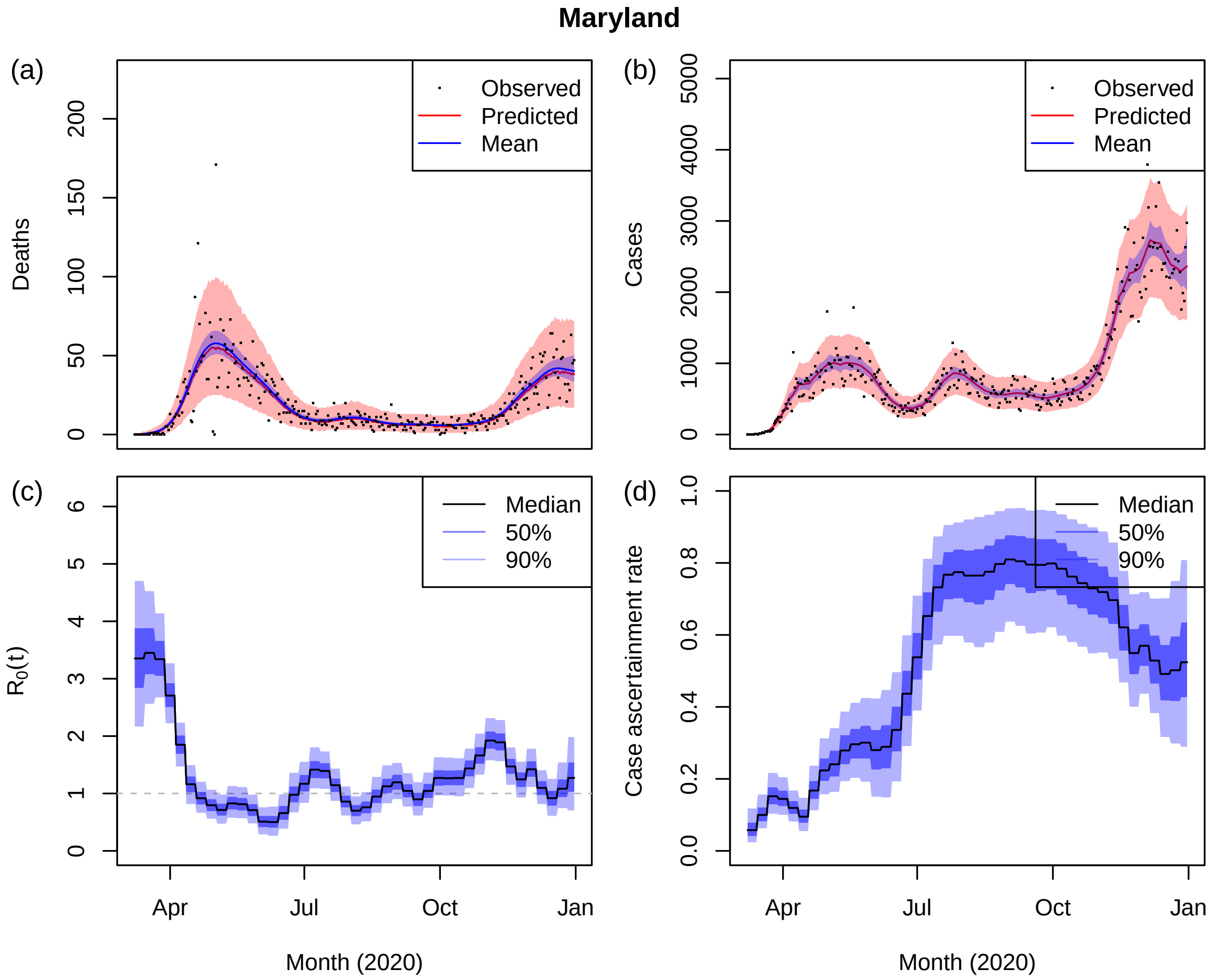

Figure S53: SEIRD model fit to COVID-19 data. **Top panels:** observed (a) deaths $d(t)$ and (b) cases $c(t)$ are plotted in black. Median and 90% credible intervals of the posterior predictive distributions of $d(t)$ and $c(t)$ are in red. Posterior median and 90% credible intervals of the underlying mean parameters $m_D(t)$ and $m_C(t)$ are in blue. **Bottom panels:** posterior median, 50%, and 90% credible intervals for (c) the basic reproduction number $R_0(t)$ and (d) the case ascertainment rate CAR(t).

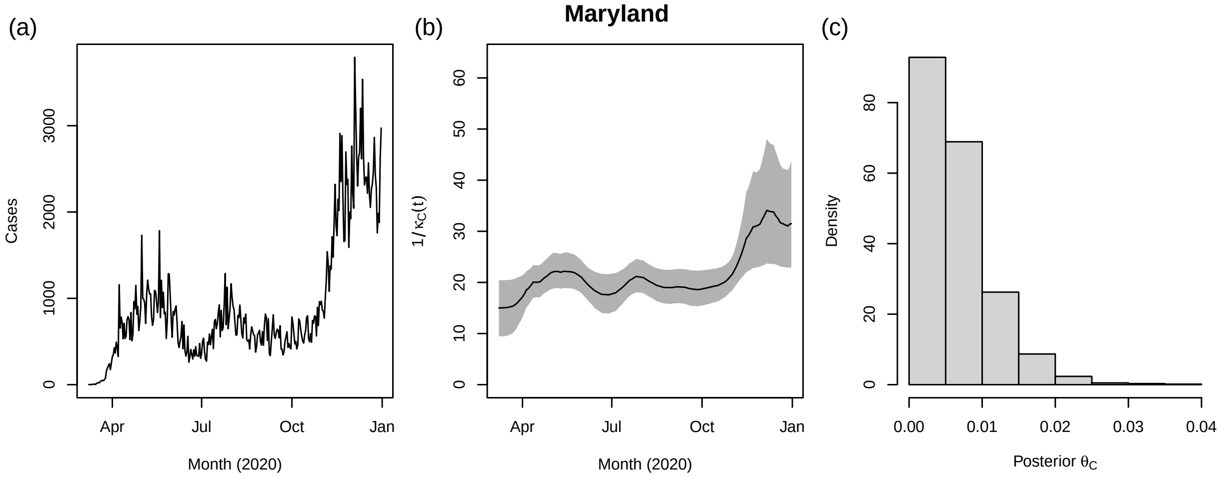

Figure S54: Overdispersion and zero-inflation in state-level clinical case data. (a) Reported cases. (b) Posterior median and 90% credible interval for the time-varying negative binomial overdispersion parameter $\kappa_C(t)^{-1}$. (c) Posterior histogram for the zero-inflation parameter θ_C.

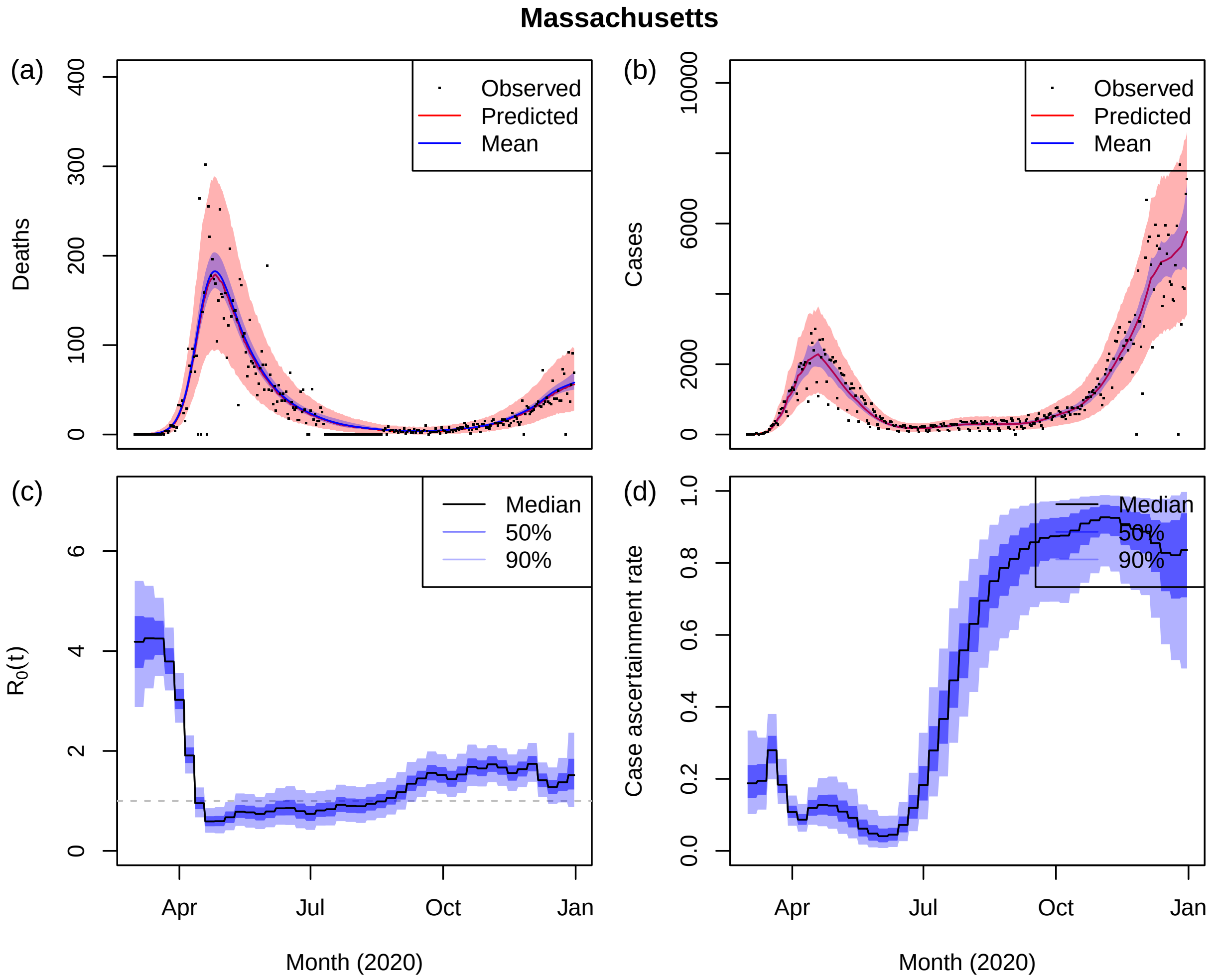

Figure S55: SEIRD model fit to COVID-19 data. **Top panels:** observed (a) deaths $d(t)$ and (b) cases $c(t)$ are plotted in black. Median and 90% credible intervals of the posterior predictive distributions of $d(t)$ and $c(t)$ are in red. Posterior median and 90% credible intervals of the underlying mean parameters $m_D(t)$ and $m_C(t)$ are in blue. **Bottom panels:** posterior median, 50%, and 90% credible intervals for (c) the basic reproduction number $R_0(t)$ and (d) the case ascertainment rate $\text{CAR}(t)$.

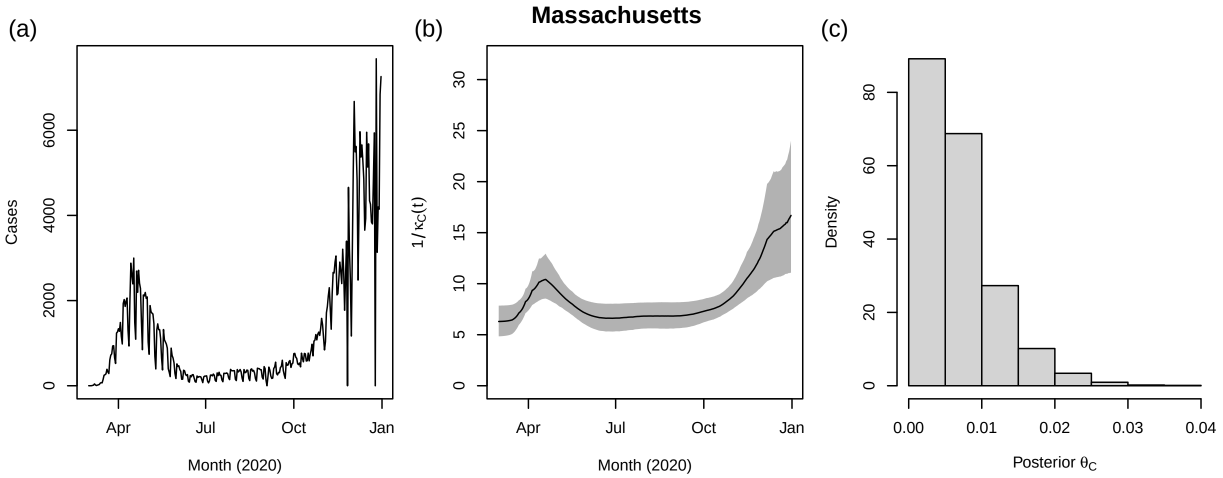

Figure S56: Overdispersion and zero-inflation in state-level clinical case data. (a) Reported cases. (b) Posterior median and 90% credible interval for the time-varying negative binomial overdispersion parameter $\kappa_C(t)^{-1}$. (c) Posterior histogram for the zero-inflation parameter θ_C.

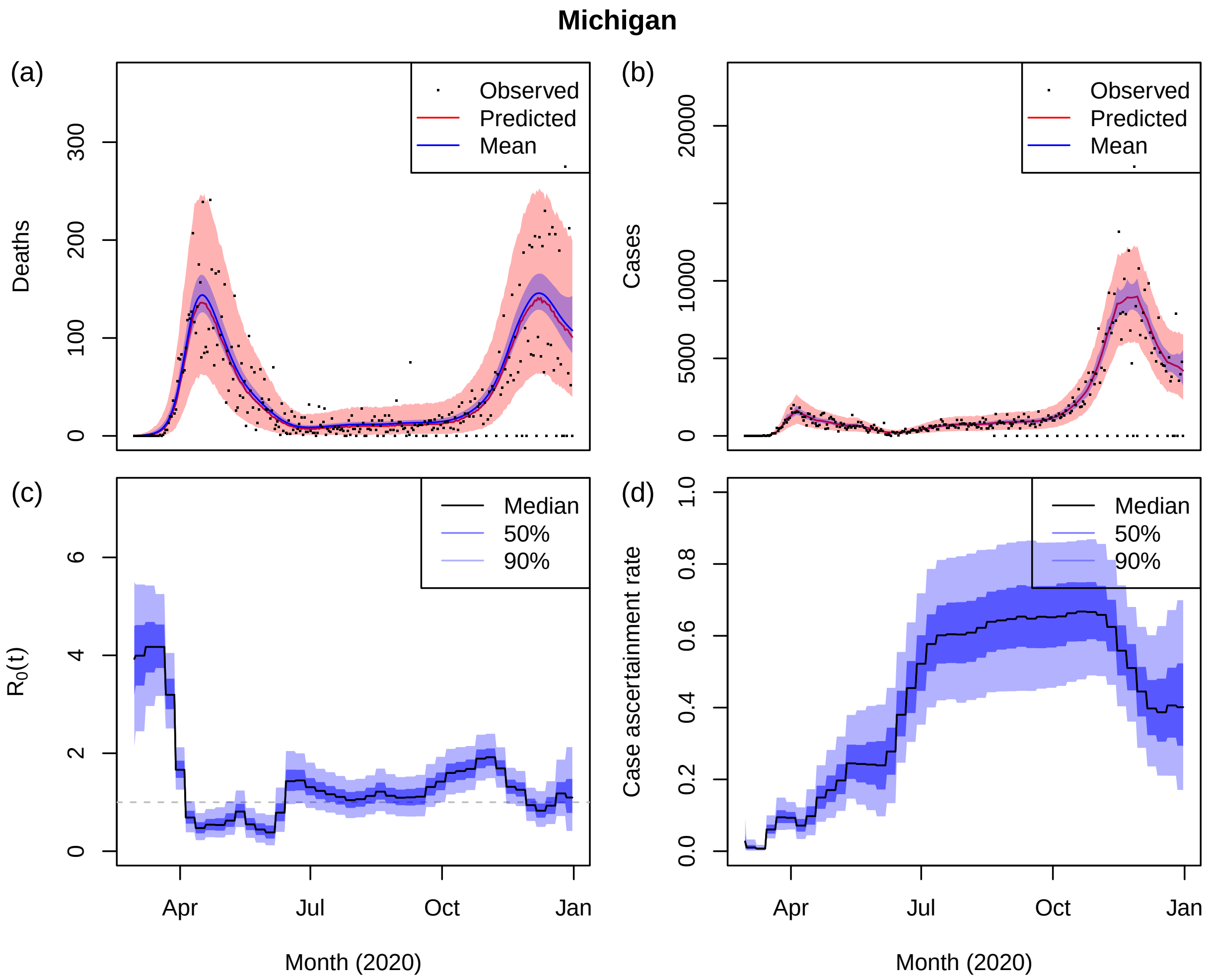

Figure S57: SEIRD model fit to COVID-19 data. **Top panels:** observed (a) deaths $d(t)$ and (b) cases $c(t)$ are plotted in black. Median and 90% credible intervals of the posterior predictive distributions of $d(t)$ and $c(t)$ are in red. Posterior median and 90% credible intervals of the underlying mean parameters $m_D(t)$ and $m_C(t)$ are in blue. **Bottom panels:** posterior median, 50%, and 90% credible intervals for (c) the basic reproduction number $R_0(t)$ and (d) the case ascertainment rate CAR(t).

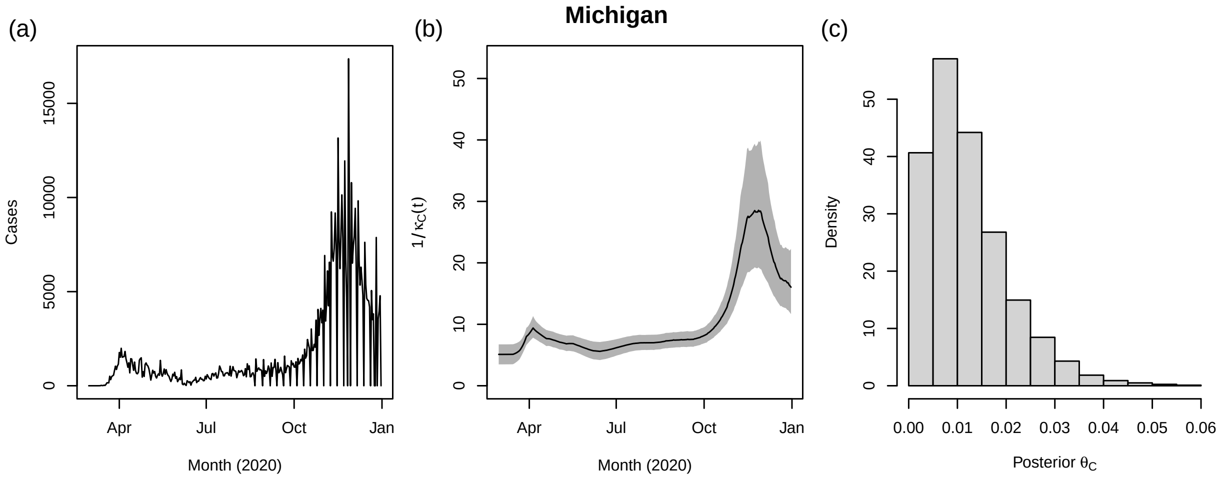

Figure S58: Overdispersion and zero-inflation in state-level clinical case data. (a) Reported cases. (b) Posterior median and 90% credible interval for the time-varying negative binomial overdispersion parameter $\kappa_C(t)^{-1}$. (c) Posterior histogram for the zero-inflation parameter θ_C.

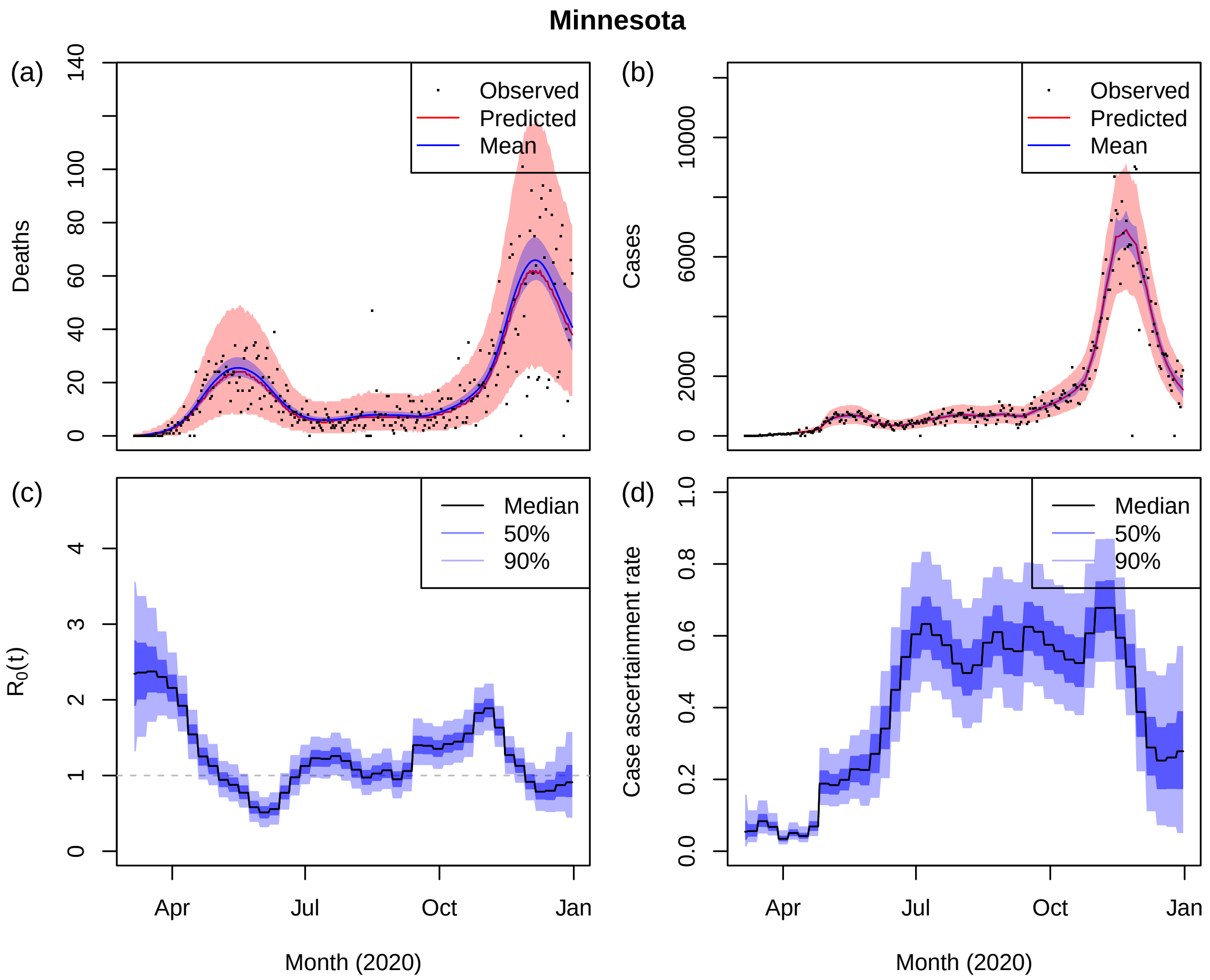

Figure S59: SEIRD model fit to COVID-19 data. **Top panels:** observed (a) deaths $d(t)$ and (b) cases $c(t)$ are plotted in black. Median and 90% credible intervals of the posterior predictive distributions of $d(t)$ and $c(t)$ are in red. Posterior median and 90% credible intervals of the underlying mean parameters $m_D(t)$ and $m_C(t)$ are in blue. **Bottom panels:** posterior median, 50%, and 90% credible intervals for (c) the basic reproduction number $R_0(t)$ and (d) the case ascertainment rate $\mathrm{CAR}(t)$.

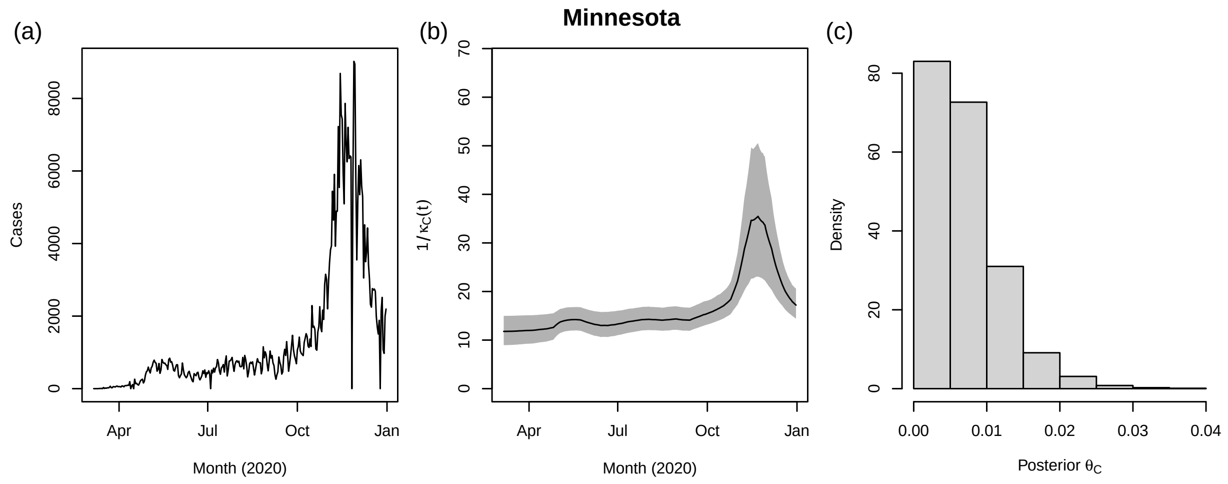

Figure S60: Overdispersion and zero-inflation in state-level clinical case data. (a) Reported cases. (b) Posterior median and 90% credible interval for the time-varying negative binomial overdispersion parameter $\kappa_C(t)^{-1}$. (c) Posterior histogram for the zero-inflation parameter θ_C.

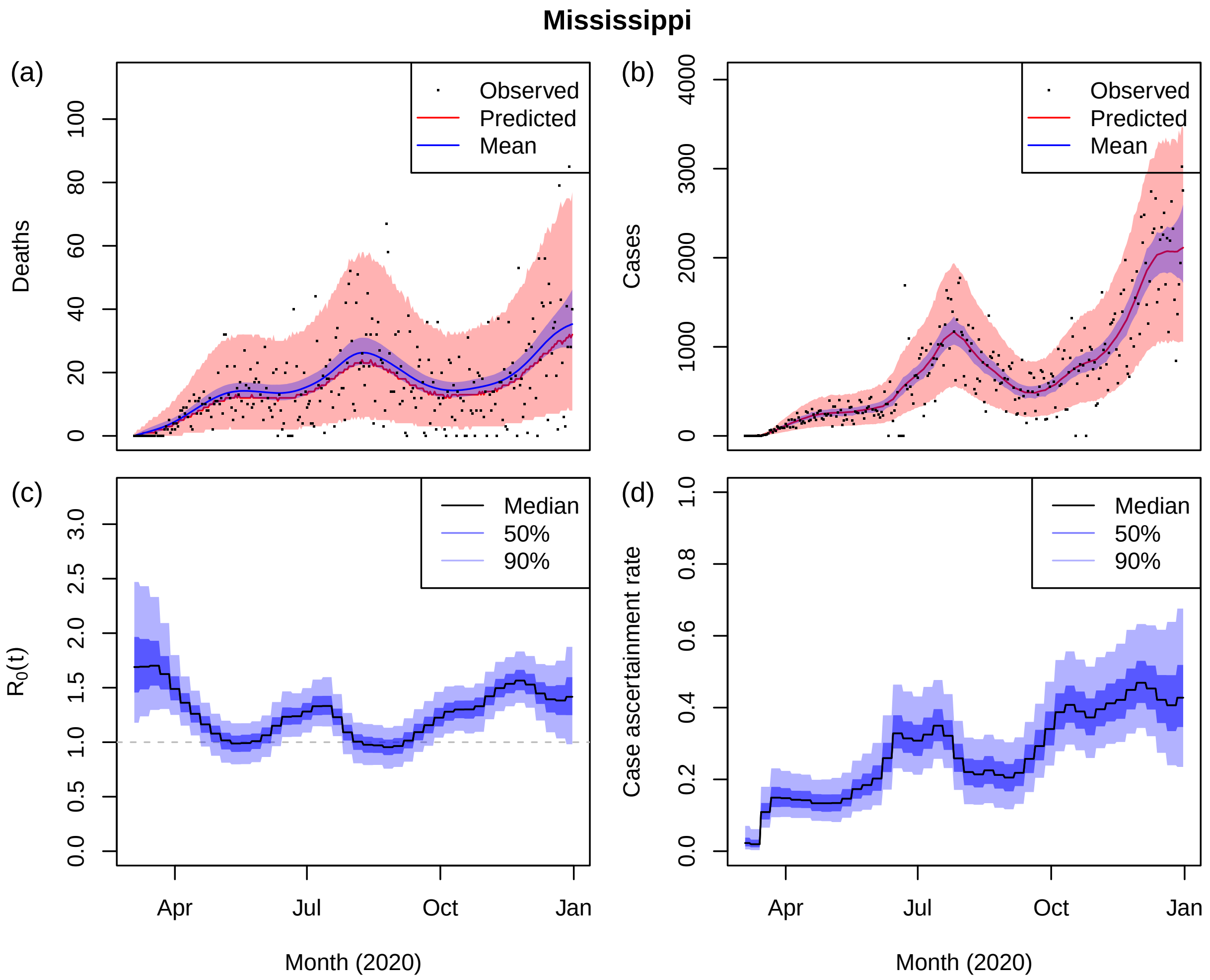

Figure S61: SEIRD model fit to COVID-19 data. **Top panels:** observed (a) deaths $d(t)$ and (b) cases $c(t)$ are plotted in black. Median and 90% credible intervals of the posterior predictive distributions of $d(t)$ and $c(t)$ are in red. Posterior median and 90% credible intervals of the underlying mean parameters $m_D(t)$ and $m_C(t)$ are in blue. **Bottom panels:** posterior median, 50%, and 90% credible intervals for (c) the basic reproduction number $R_0(t)$ and (d) the case ascertainment rate $\text{CAR}(t)$.

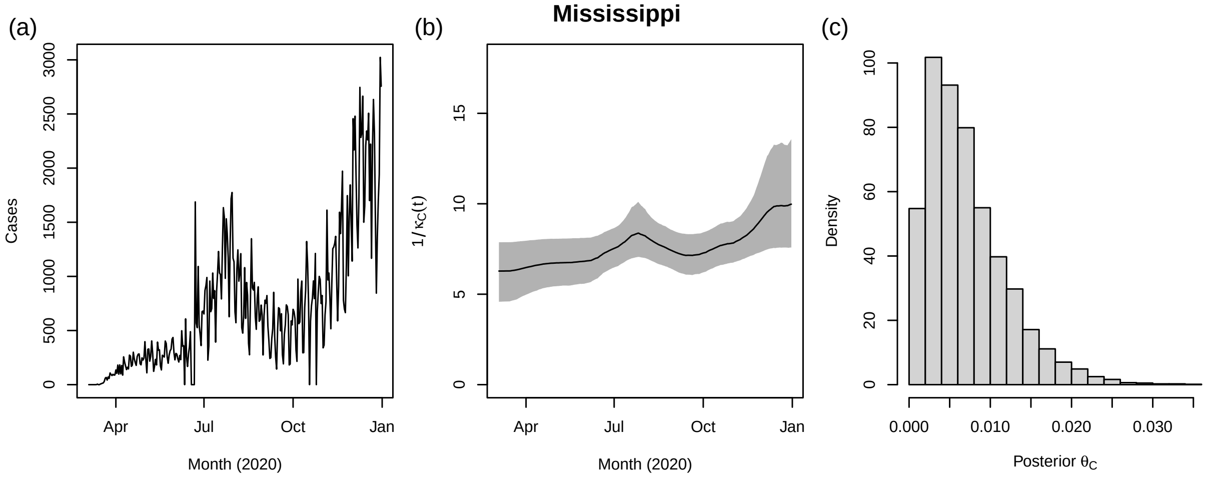

Figure S62: Overdispersion and zero-inflation in state-level clinical case data. (a) Reported cases. (b) Posterior median and 90% credible interval for the time-varying negative binomial overdispersion parameter $\kappa_C(t)^{-1}$. (c) Posterior histogram for the zero-inflation parameter θ_C.

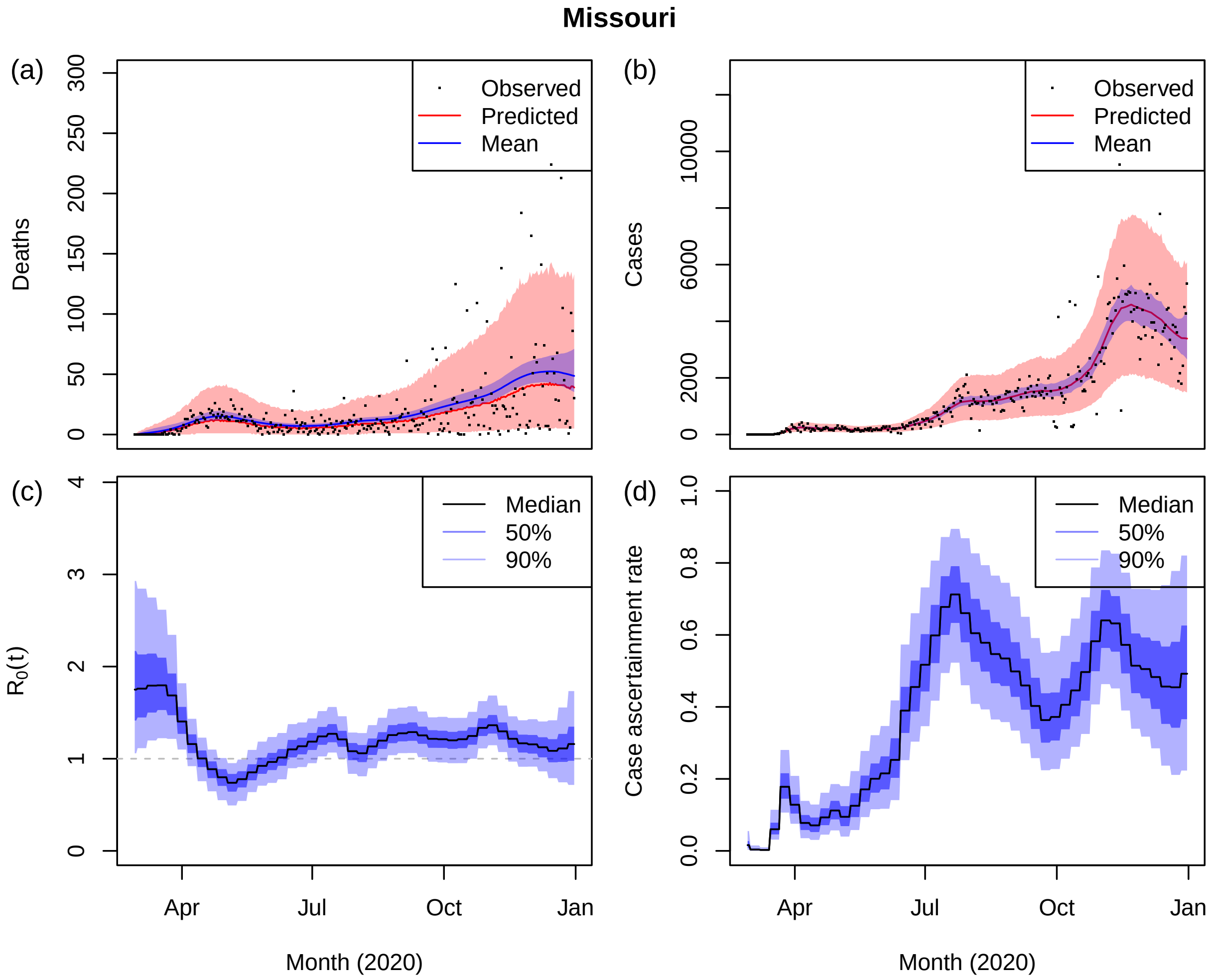

Figure S63: SEIRD model fit to COVID-19 data. **Top panels:** observed (a) deaths $d(t)$ and (b) cases $c(t)$ are plotted in black. Median and 90% credible intervals of the posterior predictive distributions of $d(t)$ and $c(t)$ are in red. Posterior median and 90% credible intervals of the underlying mean parameters $m_D(t)$ and $m_C(t)$ are in blue. **Bottom panels:** posterior median, 50%, and 90% credible intervals for (c) the basic reproduction number $R_0(t)$ and (d) the case ascertainment rate CAR(t).

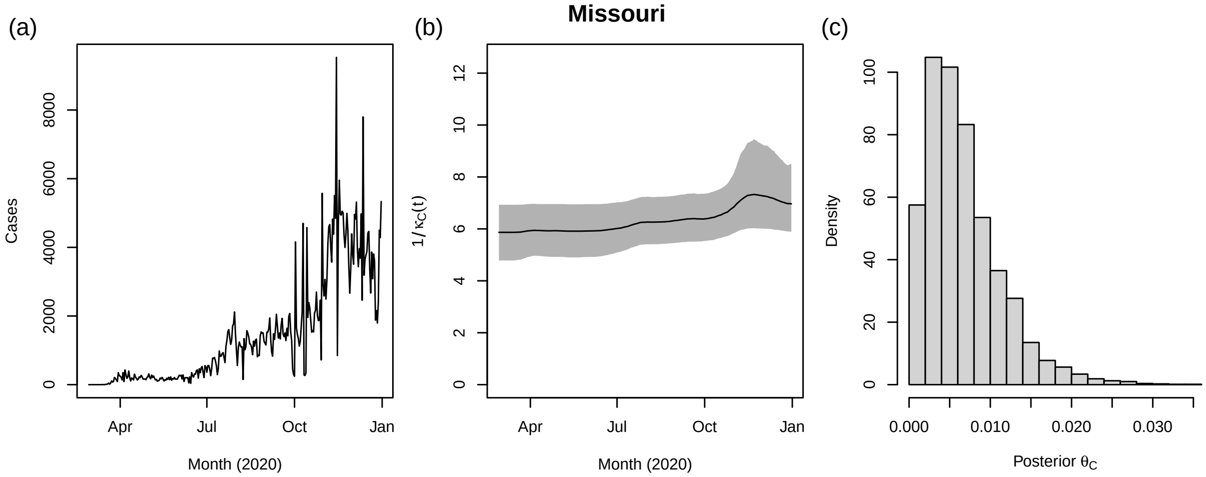

Figure S64: Overdispersion and zero-inflation in state-level clinical case data. (a) Reported cases. (b) Posterior median and 90% credible interval for the time-varying negative binomial overdispersion parameter $\kappa_C(t)^{-1}$. (c) Posterior histogram for the zero-inflation parameter θ_C.

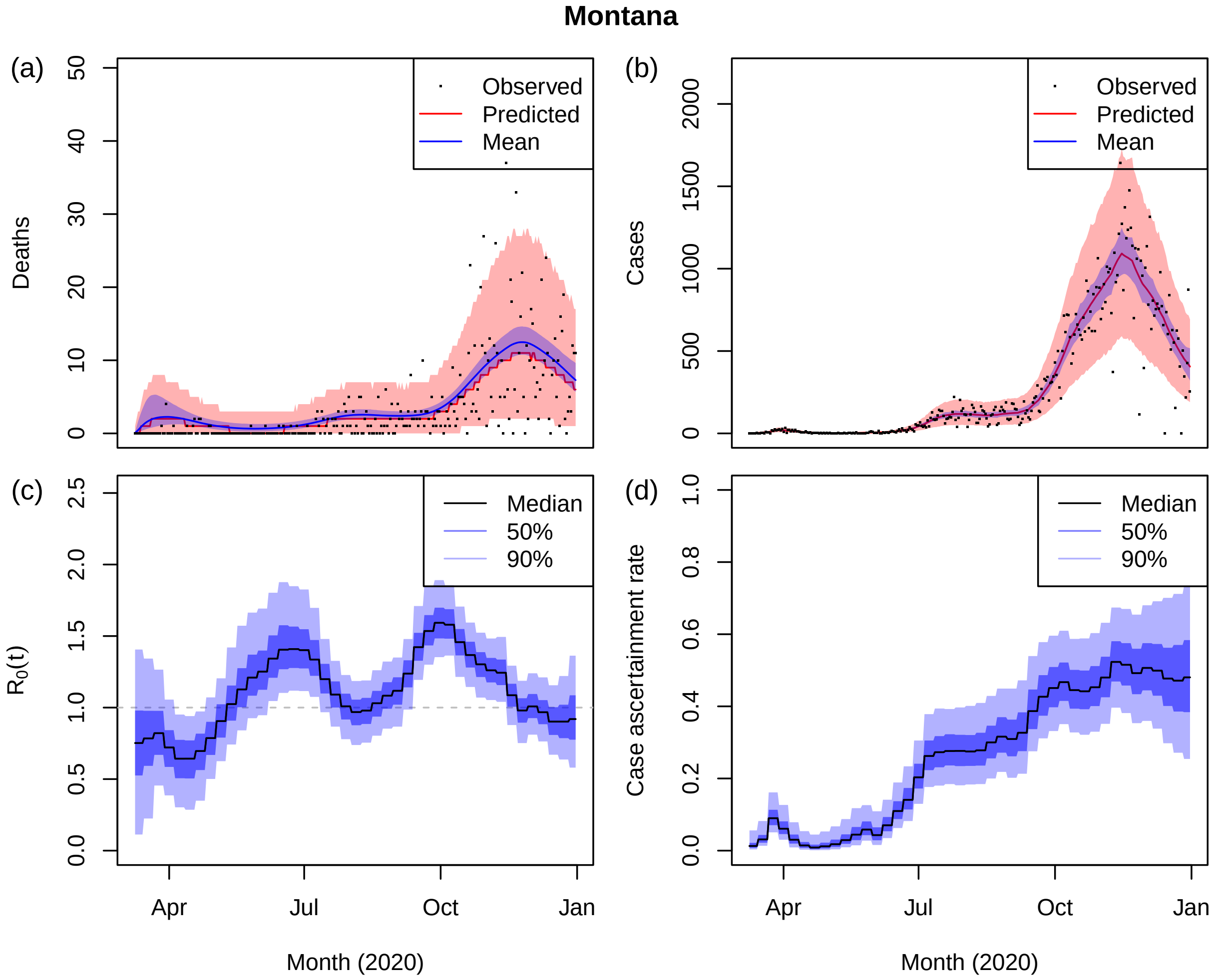

Figure S65: SEIRD model fit to COVID-19 data. **Top panels:** observed (a) deaths $d(t)$ and (b) cases $c(t)$ are plotted in black. Median and 90% credible intervals of the posterior predictive distributions of $d(t)$ and $c(t)$ are in red. Posterior median and 90% credible intervals of the underlying mean parameters $m_D(t)$ and $m_C(t)$ are in blue. **Bottom panels:** posterior median, 50%, and 90% credible intervals for (c) the basic reproduction number $R_0(t)$ and (d) the case ascertainment rate $\mathrm{CAR}(t)$.

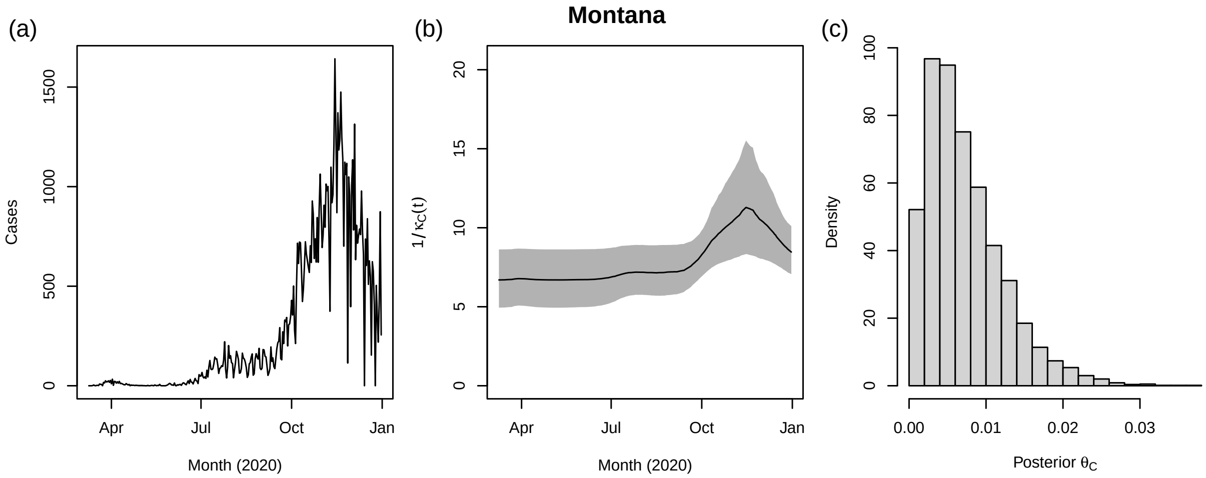

Figure S66: Overdispersion and zero-inflation in state-level clinical case data. (a) Reported cases. (b) Posterior median and 90% credible interval for the time-varying negative binomial overdispersion parameter $\kappa_C(t)^{-1}$. (c) Posterior histogram for the zero-inflation parameter θ_C.

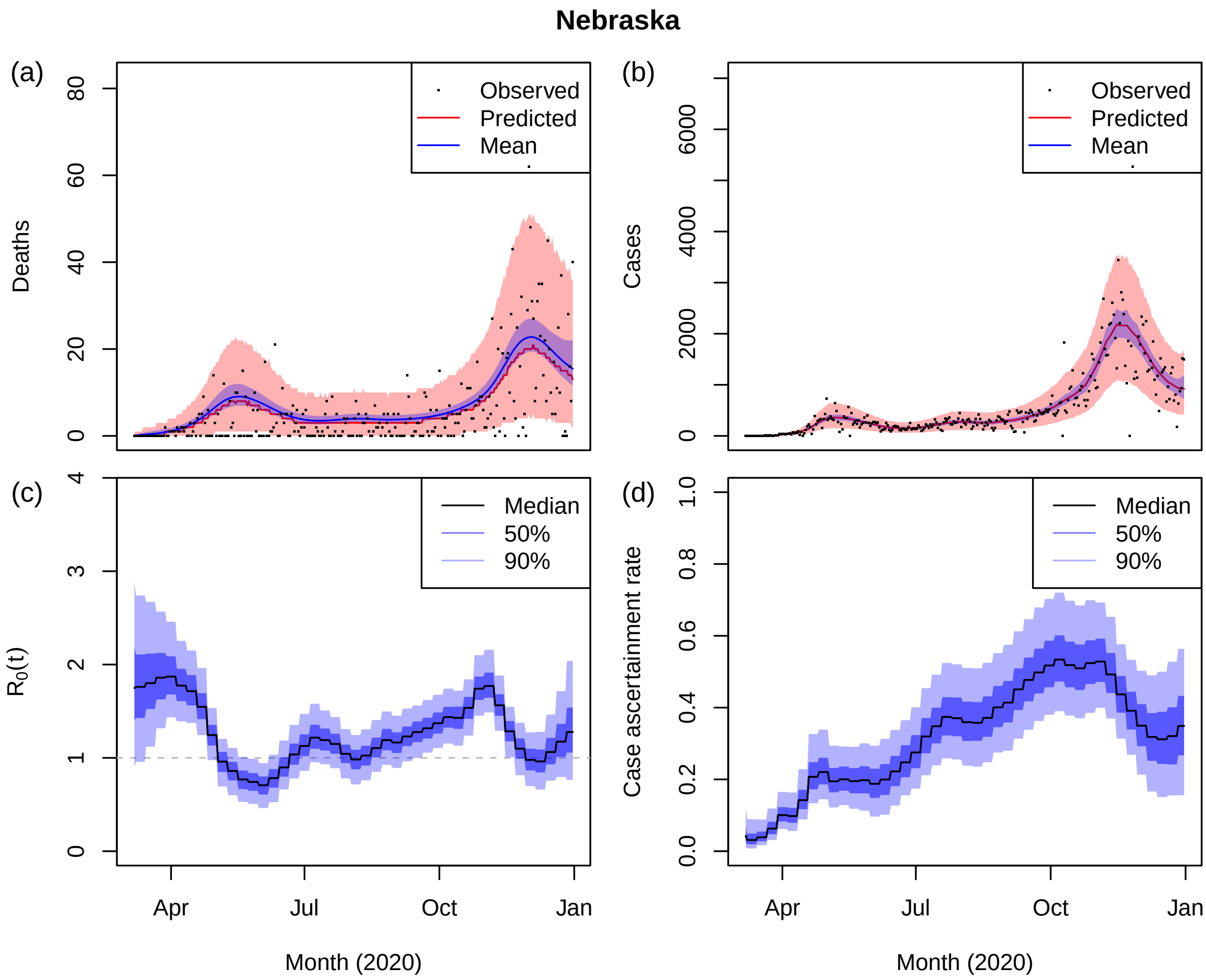

Figure S67: SEIRD model fit to COVID-19 data. **Top panels:** observed (a) deaths $d(t)$ and (b) cases $c(t)$ are plotted in black. Median and 90% credible intervals of the posterior predictive distributions of $d(t)$ and $c(t)$ are in red. Posterior median and 90% credible intervals of the underlying mean parameters $m_D(t)$ and $m_C(t)$ are in blue. **Bottom panels:** posterior median, 50%, and 90% credible intervals for (c) the basic reproduction number $R_0(t)$ and (d) the case ascertainment rate $\text{CAR}(t)$.

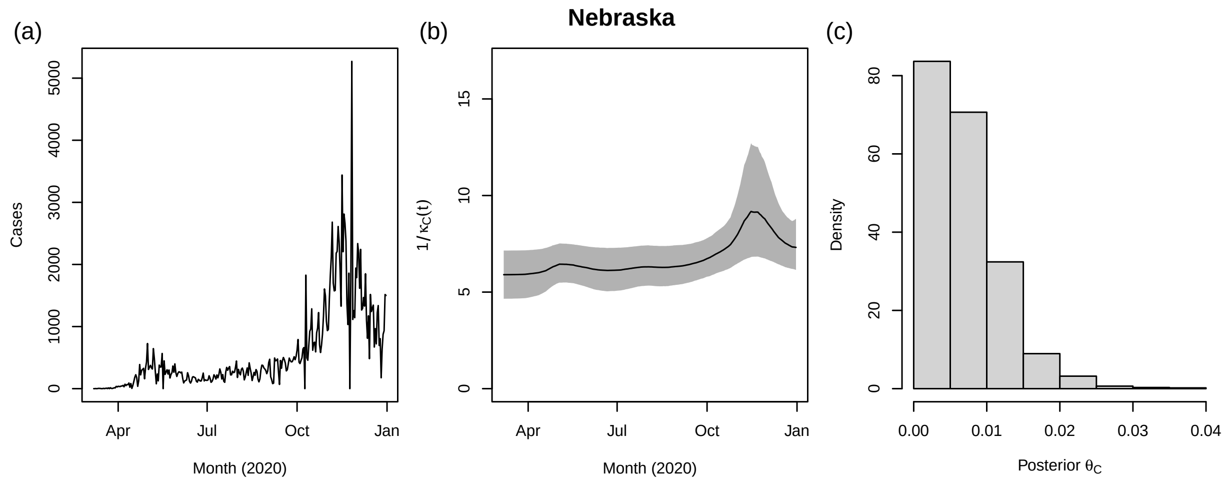

Figure S68: Overdispersion and zero-inflation in state-level clinical case data. (a) Reported cases. (b) Posterior median and 90% credible interval for the time-varying negative binomial overdispersion parameter $\kappa_C(t)^{-1}$. (c) Posterior histogram for the zero-inflation parameter θ_C.

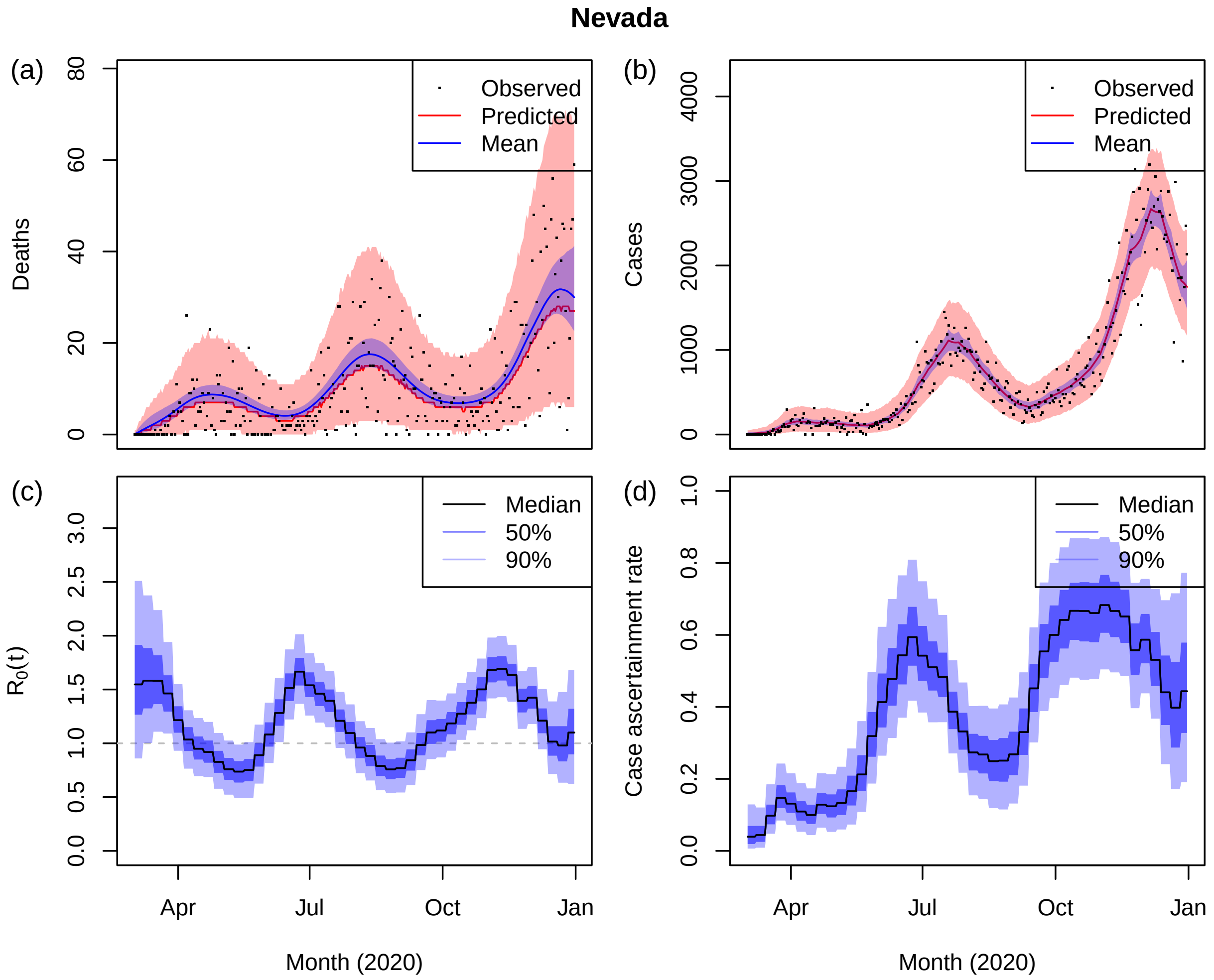

Figure S69: SEIRD model fit to COVID-19 data. **Top panels:** observed (a) deaths $d(t)$ and (b) cases $c(t)$ are plotted in black. Median and 90% credible intervals of the posterior predictive distributions of $d(t)$ and $c(t)$ are in red. Posterior median and 90% credible intervals of the underlying mean parameters $m_D(t)$ and $m_C(t)$ are in blue. **Bottom panels:** posterior median, 50%, and 90% credible intervals for (c) the basic reproduction number $R_0(t)$ and (d) the case ascertainment rate CAR(t).

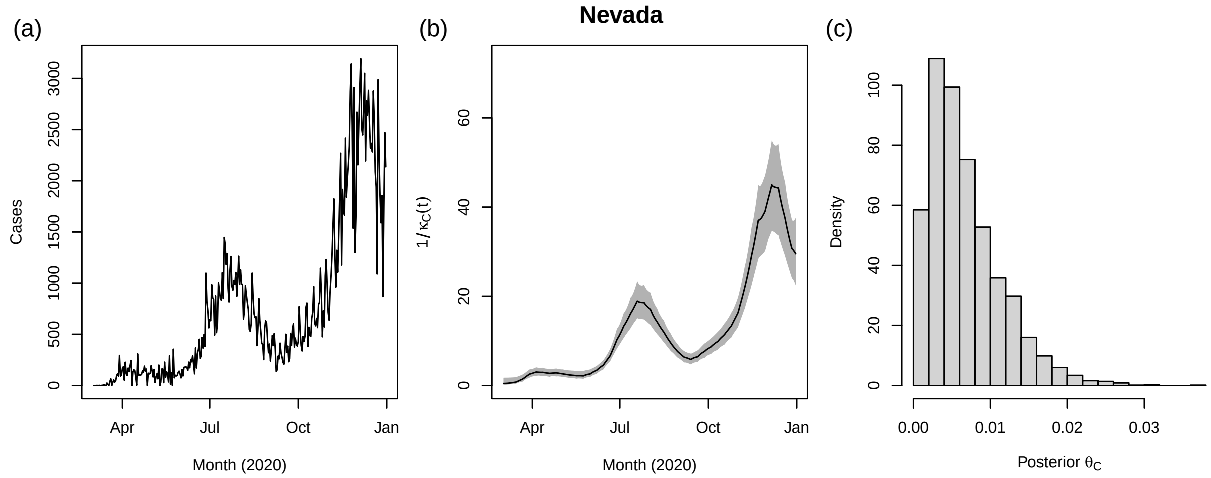

Figure S70: Overdispersion and zero-inflation in state-level clinical case data. (a) Reported cases. (b) Posterior median and 90% credible interval for the time-varying negative binomial overdispersion parameter $\kappa_C(t)^{-1}$. (c) Posterior histogram for the zero-inflation parameter θ_C.

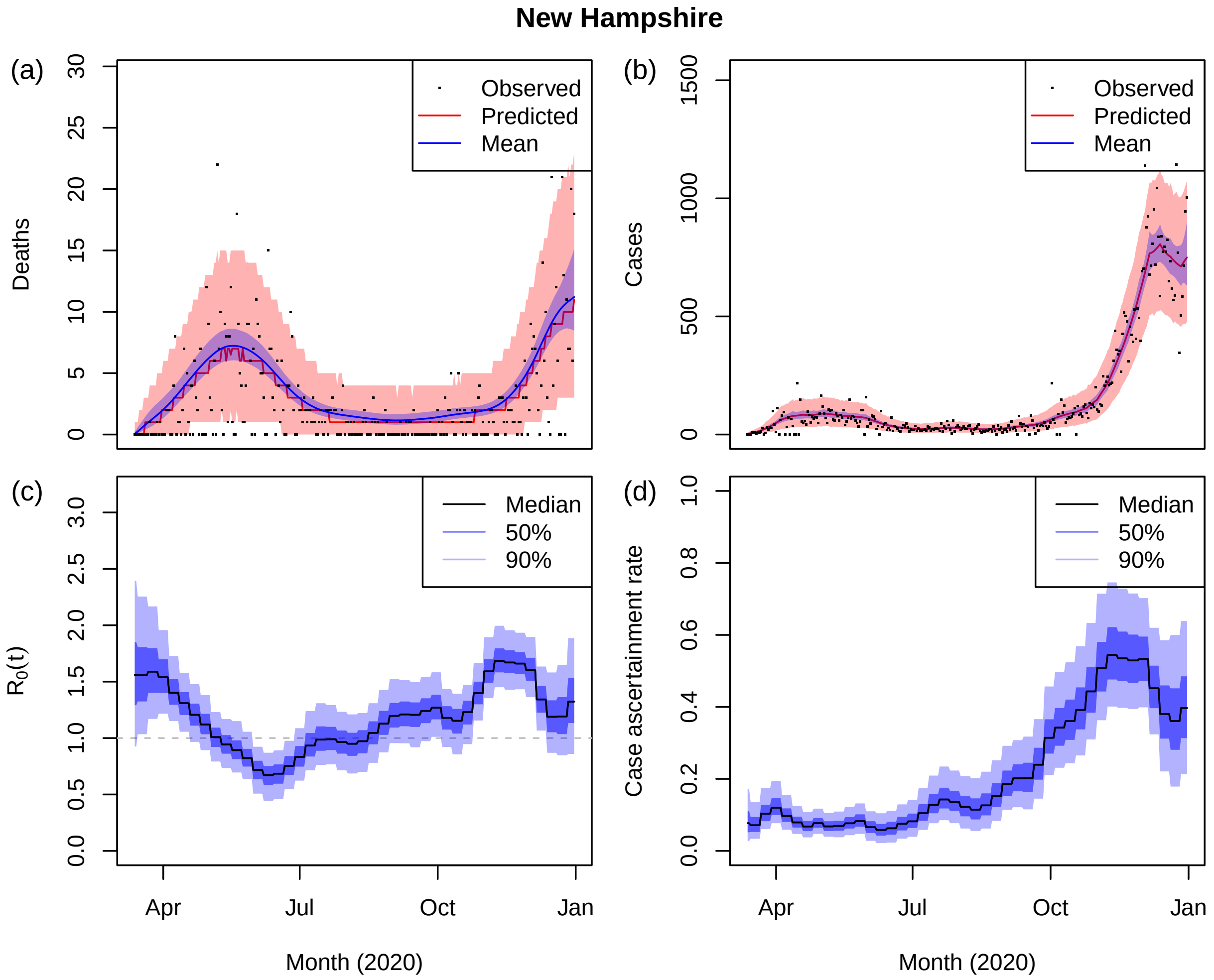

Figure S71: SEIRD model fit to COVID-19 data. **Top panels:** observed (a) deaths $d(t)$ and (b) cases $c(t)$ are plotted in black. Median and 90% credible intervals of the posterior predictive distributions of $d(t)$ and $c(t)$ are in red. Posterior median and 90% credible intervals of the underlying mean parameters $m_D(t)$ and $m_C(t)$ are in blue. **Bottom panels:** posterior median, 50%, and 90% credible intervals for (c) the basic reproduction number $R_0(t)$ and (d) the case ascertainment rate $\text{CAR}(t)$.

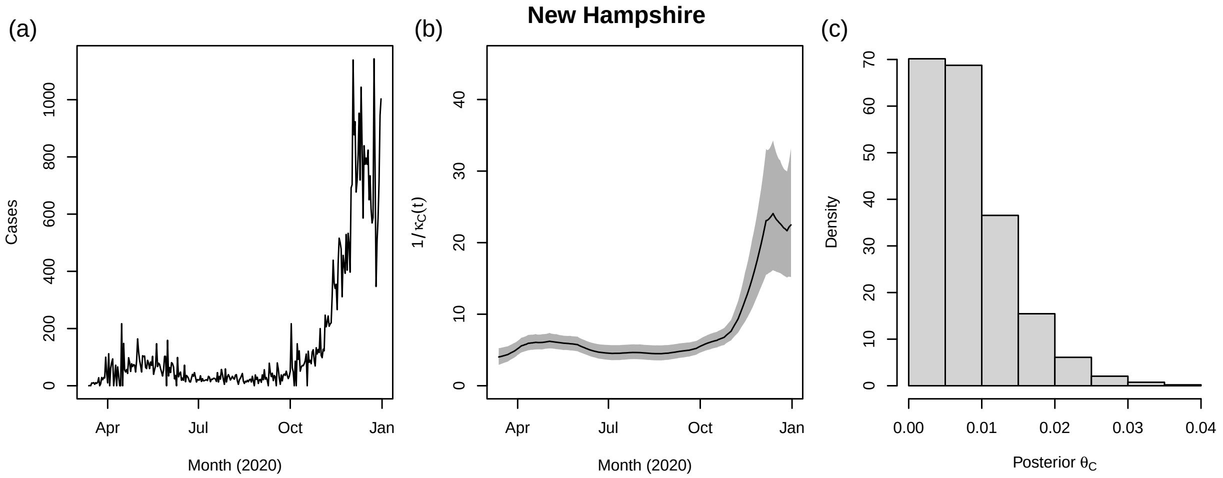

Figure S72: Overdispersion and zero-inflation in state-level clinical case data. (a) Reported cases. (b) Posterior median and 90% credible interval for the time-varying negative binomial overdispersion parameter $\kappa_C(t)^{-1}$. (c) Posterior histogram for the zero-inflation parameter θ_C.

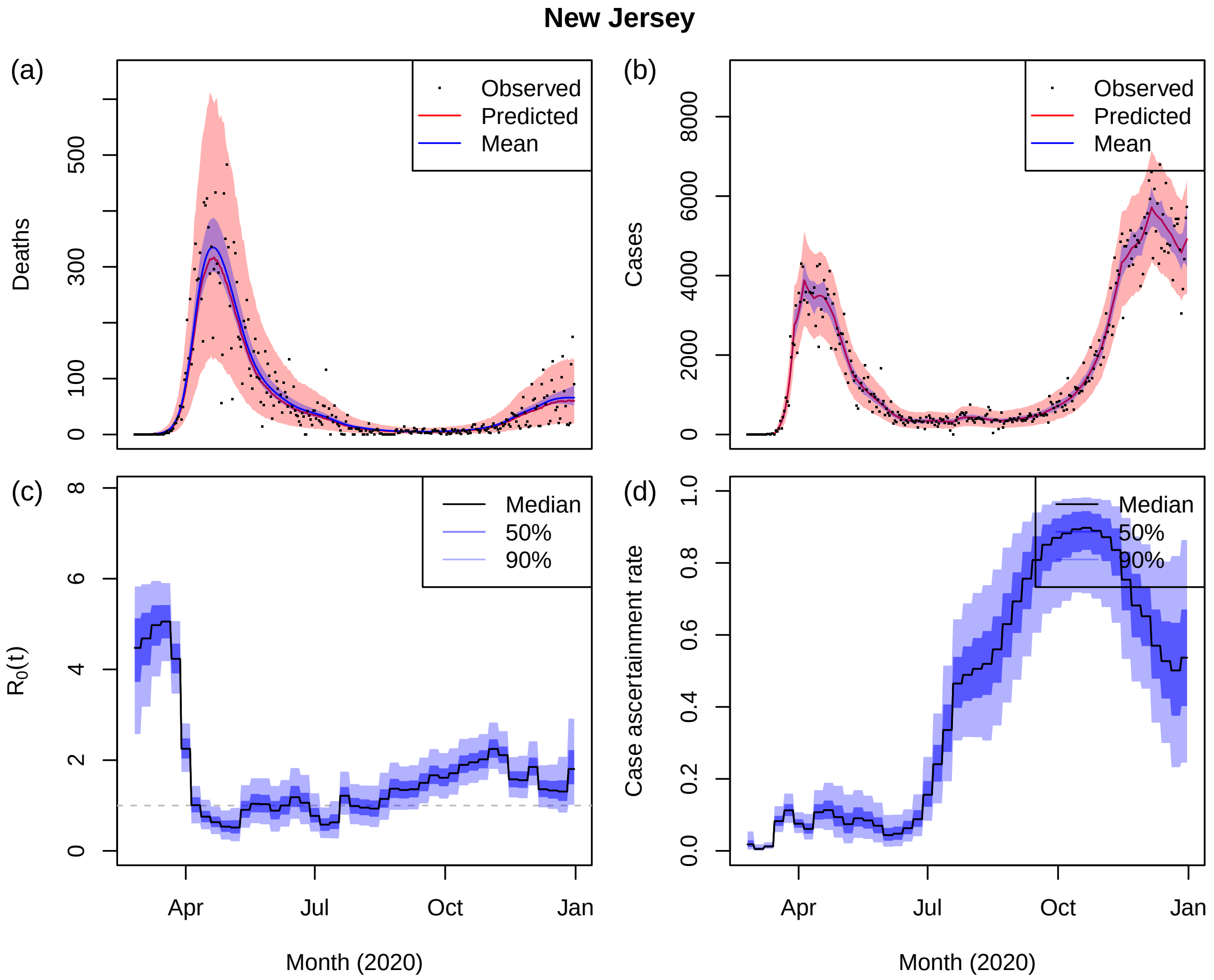

Figure S73: SEIRD model fit to COVID-19 data. **Top panels:** observed (a) deaths $d(t)$ and (b) cases $c(t)$ are plotted in black. Median and 90% credible intervals of the posterior predictive distributions of $d(t)$ and $c(t)$ are in red. Posterior median and 90% credible intervals of the underlying mean parameters $m_D(t)$ and $m_C(t)$ are in blue. **Bottom panels:** posterior median, 50%, and 90% credible intervals for (c) the basic reproduction number $R_0(t)$ and (d) the case ascertainment rate $\mathrm{CAR}(t)$.

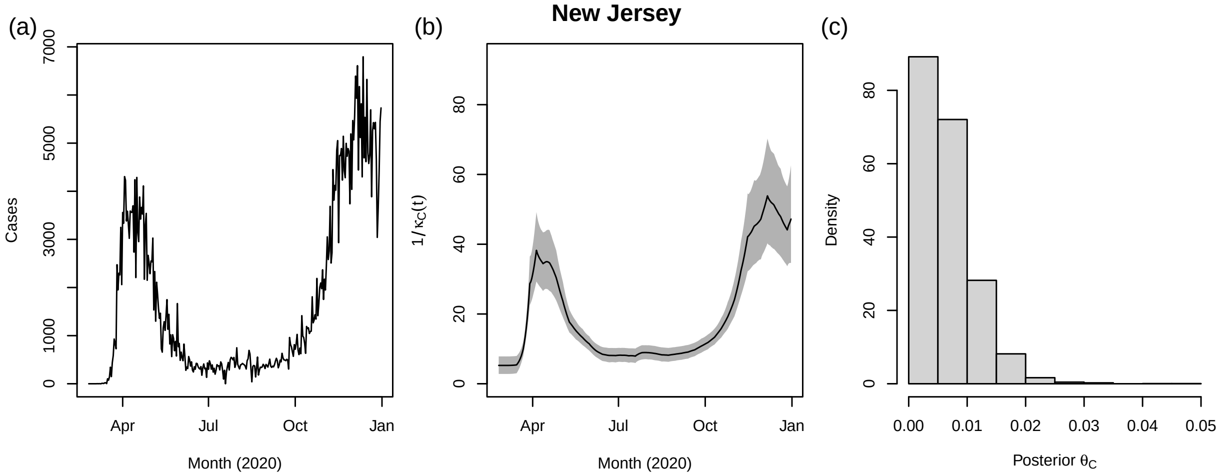

Figure S74: Overdispersion and zero-inflation in state-level clinical case data. (a) Reported cases. (b) Posterior median and 90% credible interval for the time-varying negative binomial overdispersion parameter $\kappa_C(t)^{-1}$. (c) Posterior histogram for the zero-inflation parameter θ_C.

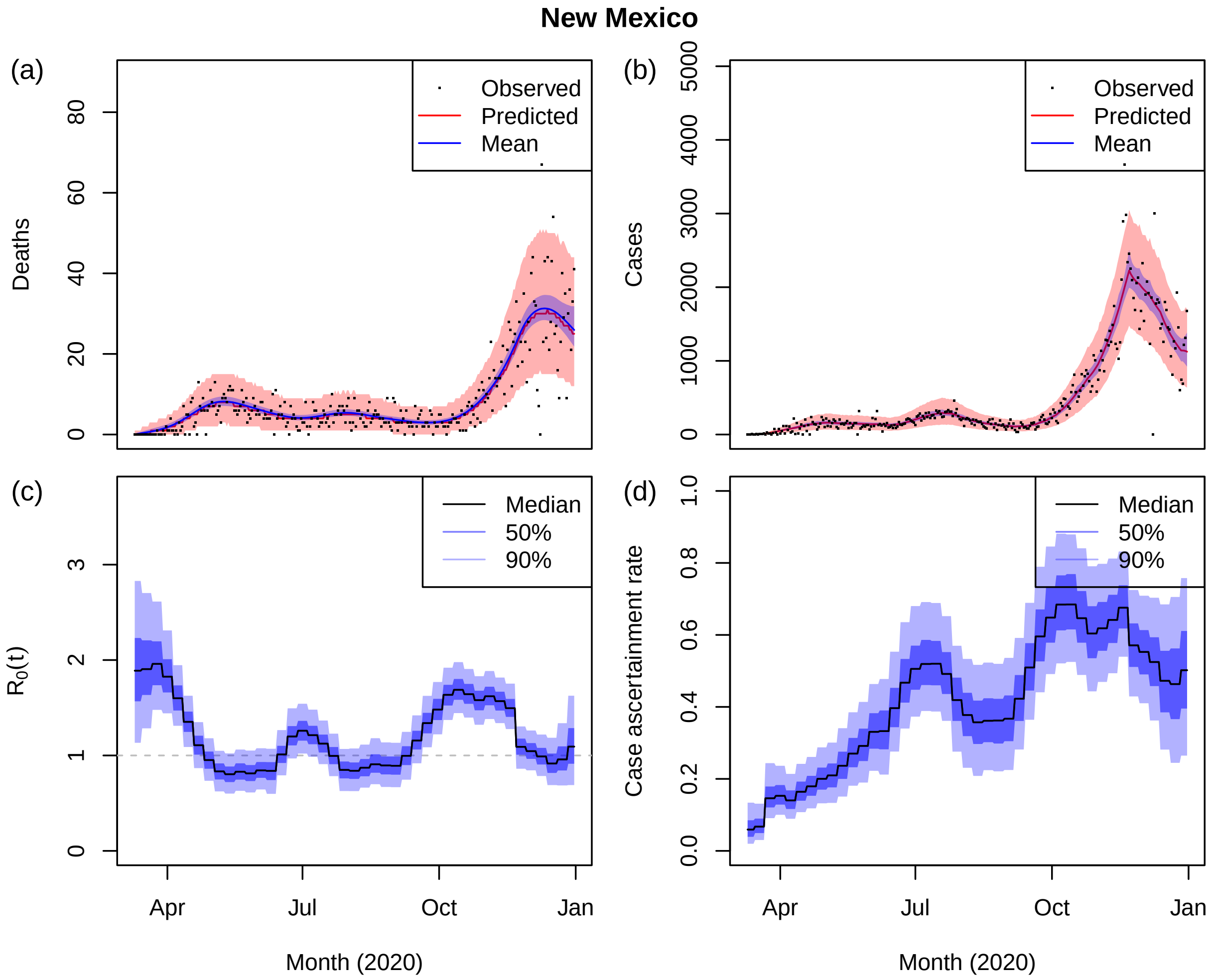

Figure S75: SEIRD model fit to COVID-19 data. **Top panels:** observed (a) deaths $d(t)$ and (b) cases $c(t)$ are plotted in black. Median and 90% credible intervals of the posterior predictive distributions of $d(t)$ and $c(t)$ are in red. Posterior median and 90% credible intervals of the underlying mean parameters $m_D(t)$ and $m_C(t)$ are in blue. **Bottom panels:** posterior median, 50%, and 90% credible intervals for (c) the basic reproduction number $R_0(t)$ and (d) the case ascertainment rate $\mathrm{CAR}(t)$.

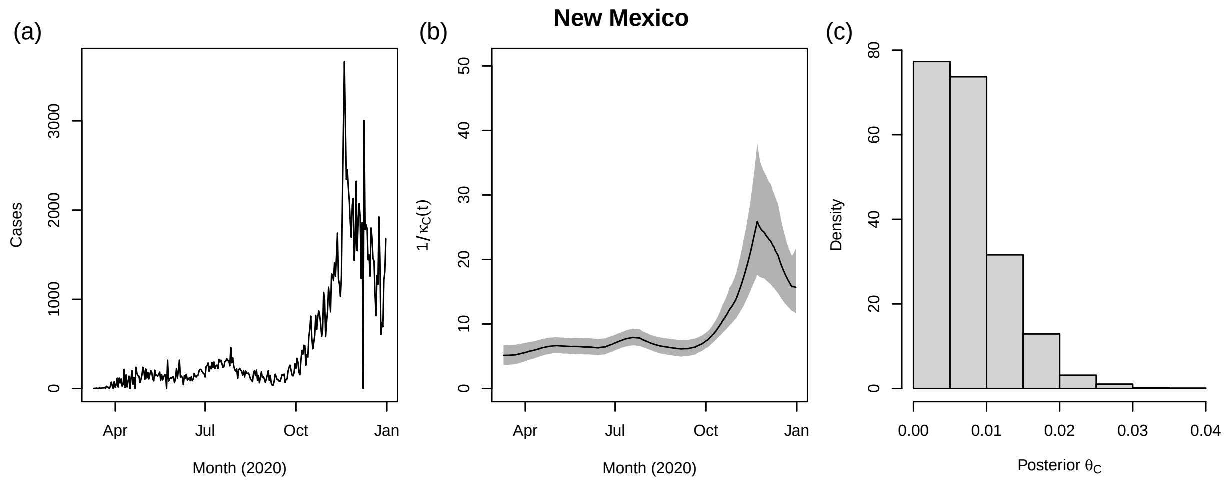

Figure S76: Overdispersion and zero-inflation in state-level clinical case data. (a) Reported cases. (b) Posterior median and 90% credible interval for the time-varying negative binomial overdispersion parameter $\kappa_C(t)^{-1}$. (c) Posterior histogram for the zero-inflation parameter θ_C.

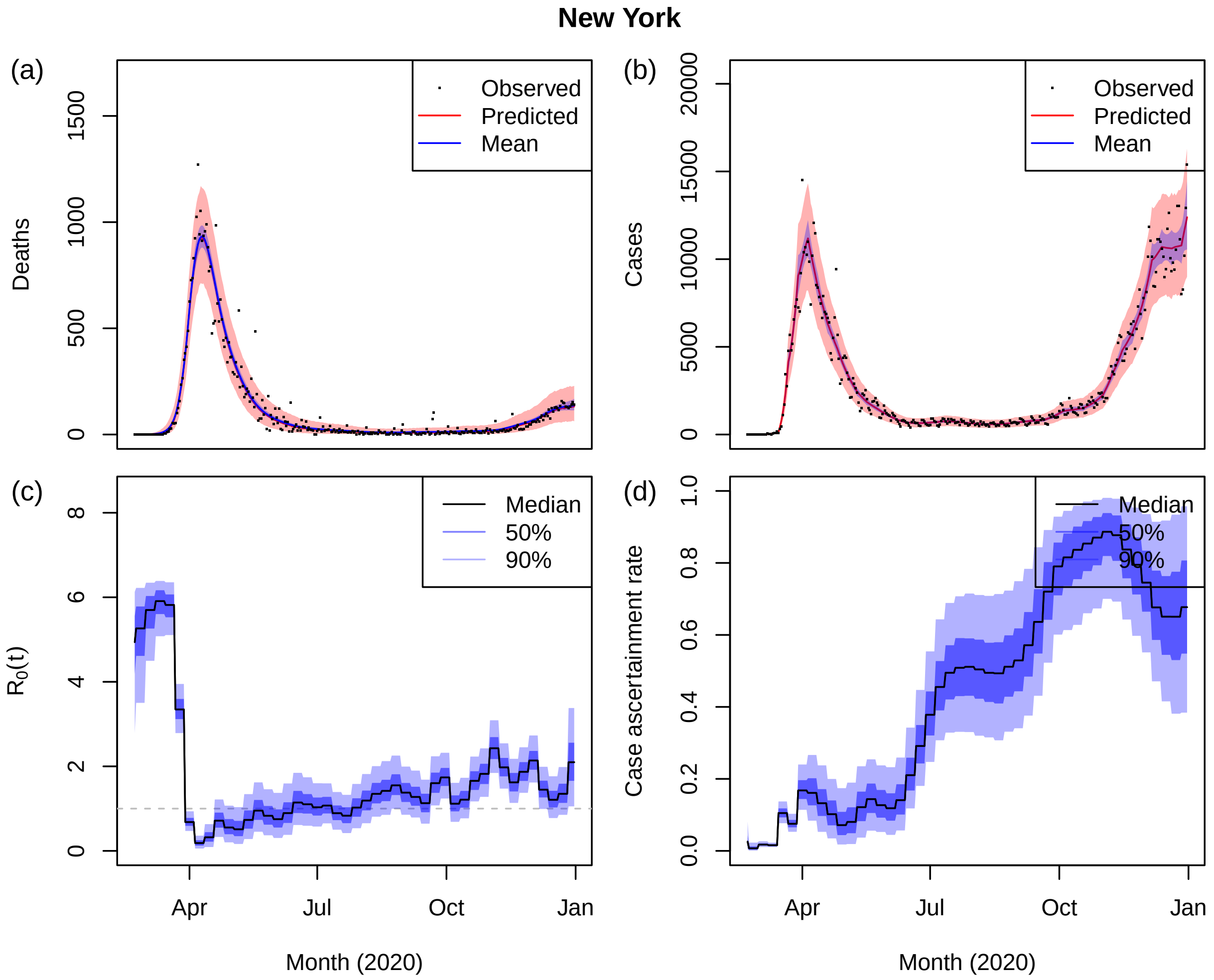

Figure S77: SEIRD model fit to COVID-19 data. **Top panels:** observed (a) deaths $d(t)$ and (b) cases $c(t)$ are plotted in black. Median and 90% credible intervals of the posterior predictive distributions of $d(t)$ and $c(t)$ are in red. Posterior median and 90% credible intervals of the underlying mean parameters $m_D(t)$ and $m_C(t)$ are in blue. **Bottom panels:** posterior median, 50%, and 90% credible intervals for (c) the basic reproduction number $R_0(t)$ and (d) the case ascertainment rate CAR(t).

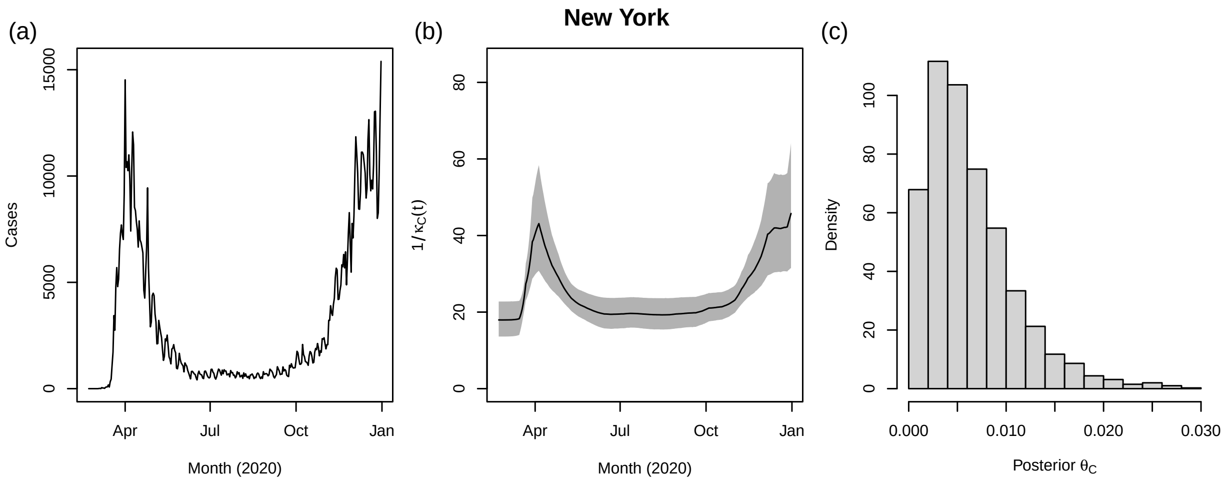

Figure S78: Overdispersion and zero-inflation in state-level clinical case data. (a) Reported cases. (b) Posterior median and 90% credible interval for the time-varying negative binomial overdispersion parameter $\kappa_C(t)^{-1}$. (c) Posterior histogram for the zero-inflation parameter θ_C.

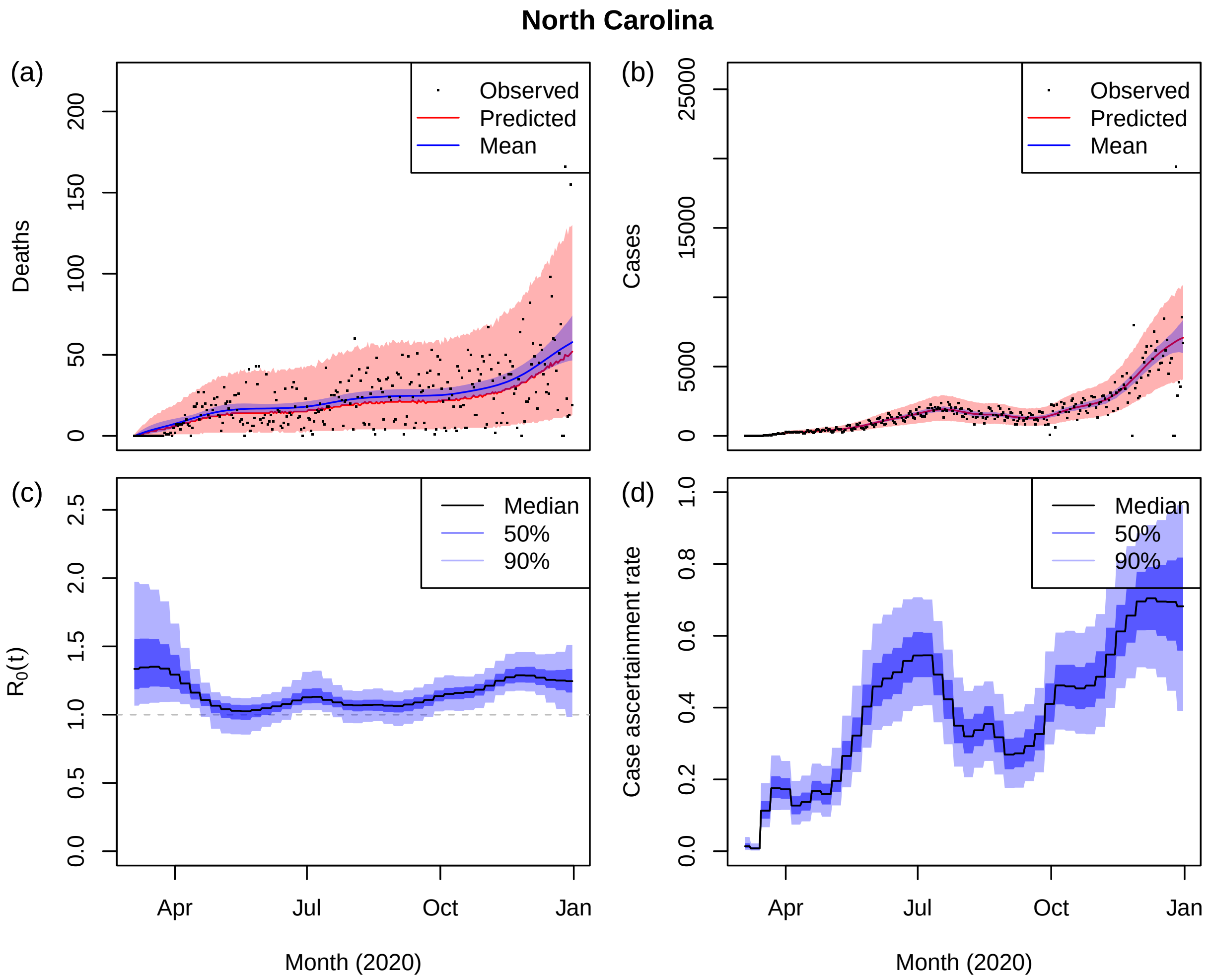

Figure S79: SEIRD model fit to COVID-19 data. **Top panels:** observed (a) deaths $d(t)$ and (b) cases $c(t)$ are plotted in black. Median and 90% credible intervals of the posterior predictive distributions of $d(t)$ and $c(t)$ are in red. Posterior median and 90% credible intervals of the underlying mean parameters $m_D(t)$ and $m_C(t)$ are in blue. **Bottom panels:** posterior median, 50%, and 90% credible intervals for (c) the basic reproduction number $R_0(t)$ and (d) the case ascertainment rate $\text{CAR}(t)$.

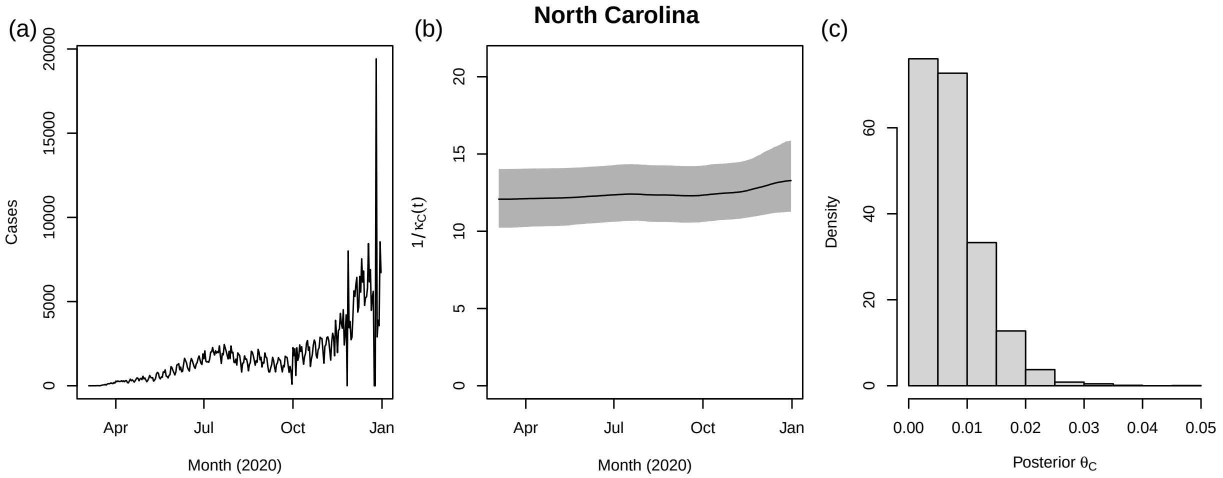

Figure S80: Overdispersion and zero-inflation in state-level clinical case data. (a) Reported cases. (b) Posterior median and 90% credible interval for the time-varying negative binomial overdispersion parameter $\kappa_C(t)^{-1}$. (c) Posterior histogram for the zero-inflation parameter θ_C.

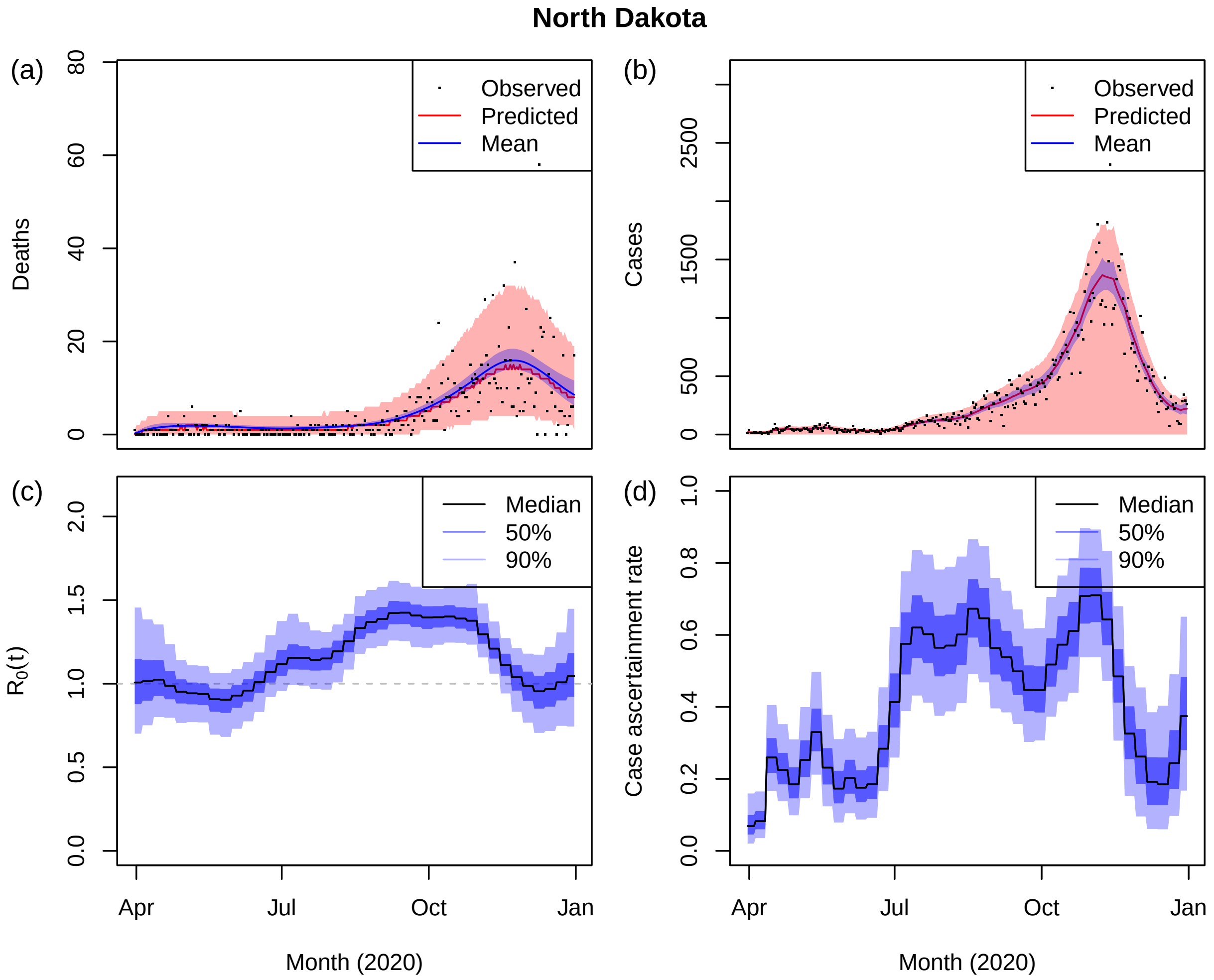

Figure S81: SEIRD model fit to COVID-19 data. **Top panels:** observed (a) deaths $d(t)$ and (b) cases $c(t)$ are plotted in black. Median and 90% credible intervals of the posterior predictive distributions of $d(t)$ and $c(t)$ are in red. Posterior median and 90% credible intervals of the underlying mean parameters $m_D(t)$ and $m_C(t)$ are in blue. **Bottom panels:** posterior median, 50%, and 90% credible intervals for (c) the basic reproduction number $R_0(t)$ and (d) the case ascertainment rate $\text{CAR}(t)$.

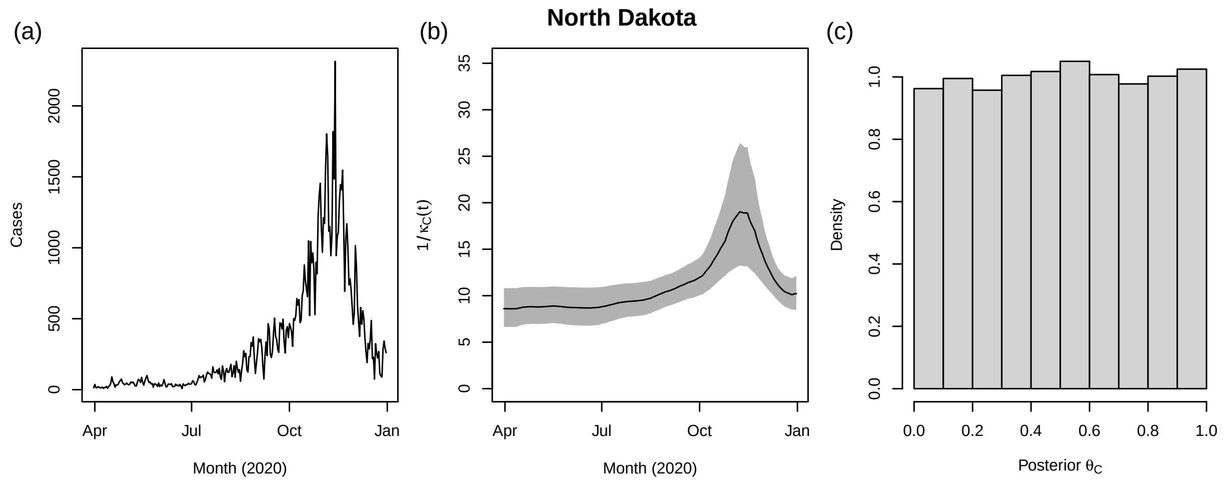

Figure S82: Overdispersion and zero-inflation in state-level clinical case data. (a) Reported cases. (b) Posterior median and 90% credible interval for the time-varying negative binomial overdispersion parameter $\kappa_C(t)^{-1}$. (c) Posterior histogram for the zero-inflation parameter θ_C.

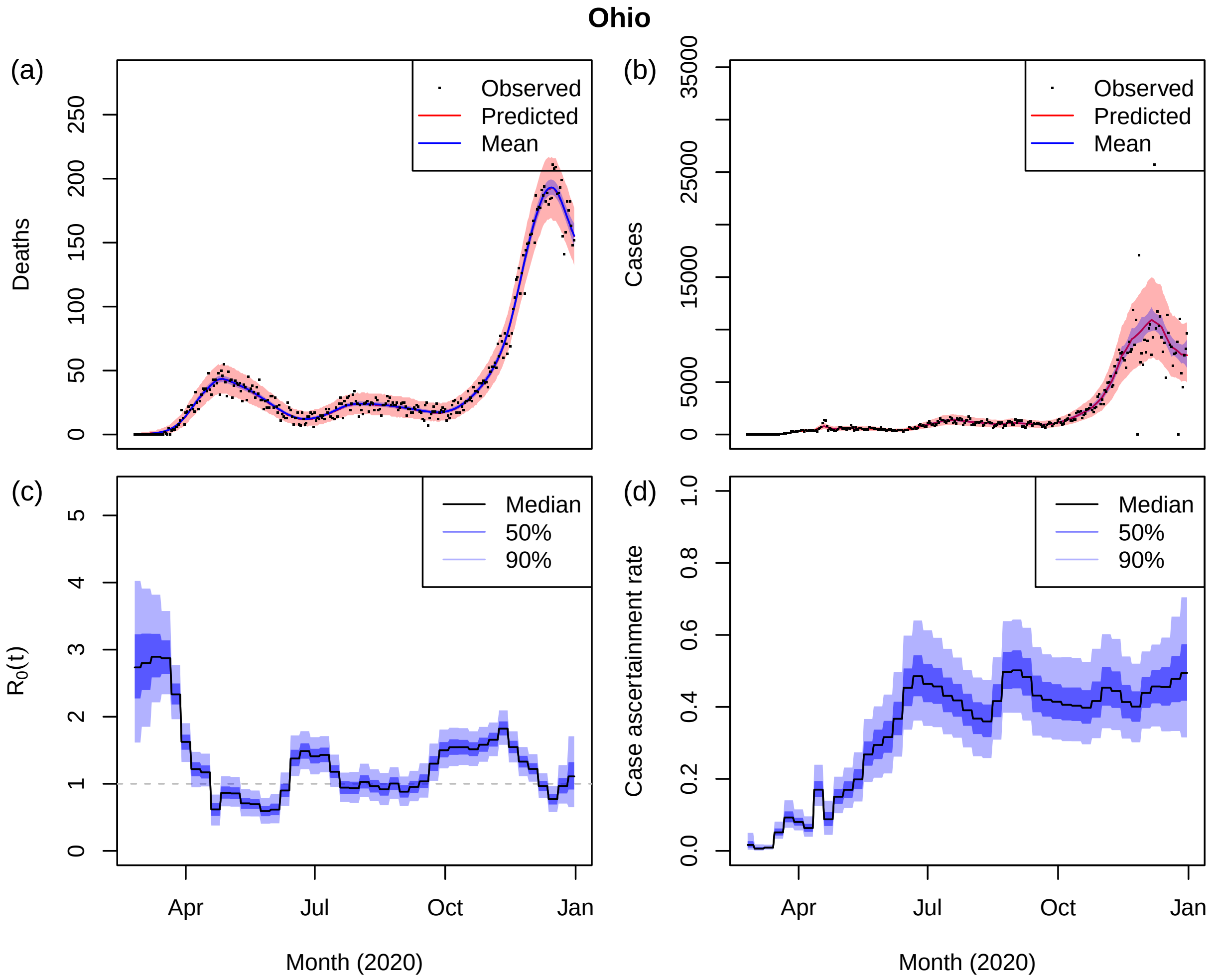

Figure S83: SEIRD model fit to COVID-19 data. **Top panels:** observed (a) deaths $d(t)$ and (b) cases $c(t)$ are plotted in black. Median and 90% credible intervals of the posterior predictive distributions of $d(t)$ and $c(t)$ are in red. Posterior median and 90% credible intervals of the underlying mean parameters $m_D(t)$ and $m_C(t)$ are in blue. **Bottom panels:** posterior median, 50%, and 90% credible intervals for (c) the basic reproduction number $R_0(t)$ and (d) the case ascertainment rate $\text{CAR}(t)$.

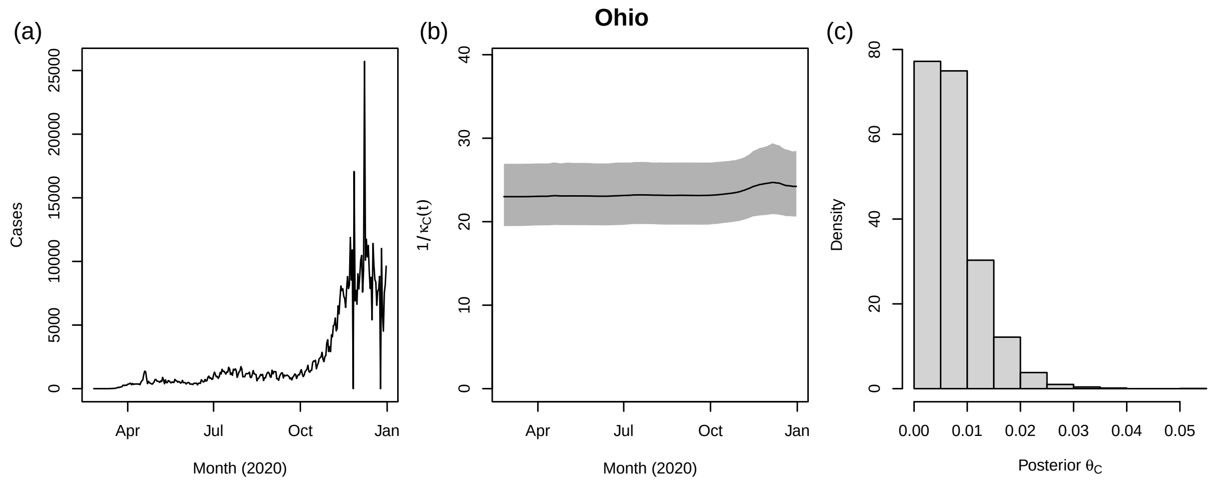

Figure S84: Overdispersion and zero-inflation in state-level clinical case data. (a) Reported cases. (b) Posterior median and 90% credible interval for the time-varying negative binomial overdispersion parameter $\kappa_C(t)^{-1}$. (c) Posterior histogram for the zero-inflation parameter θ_C.

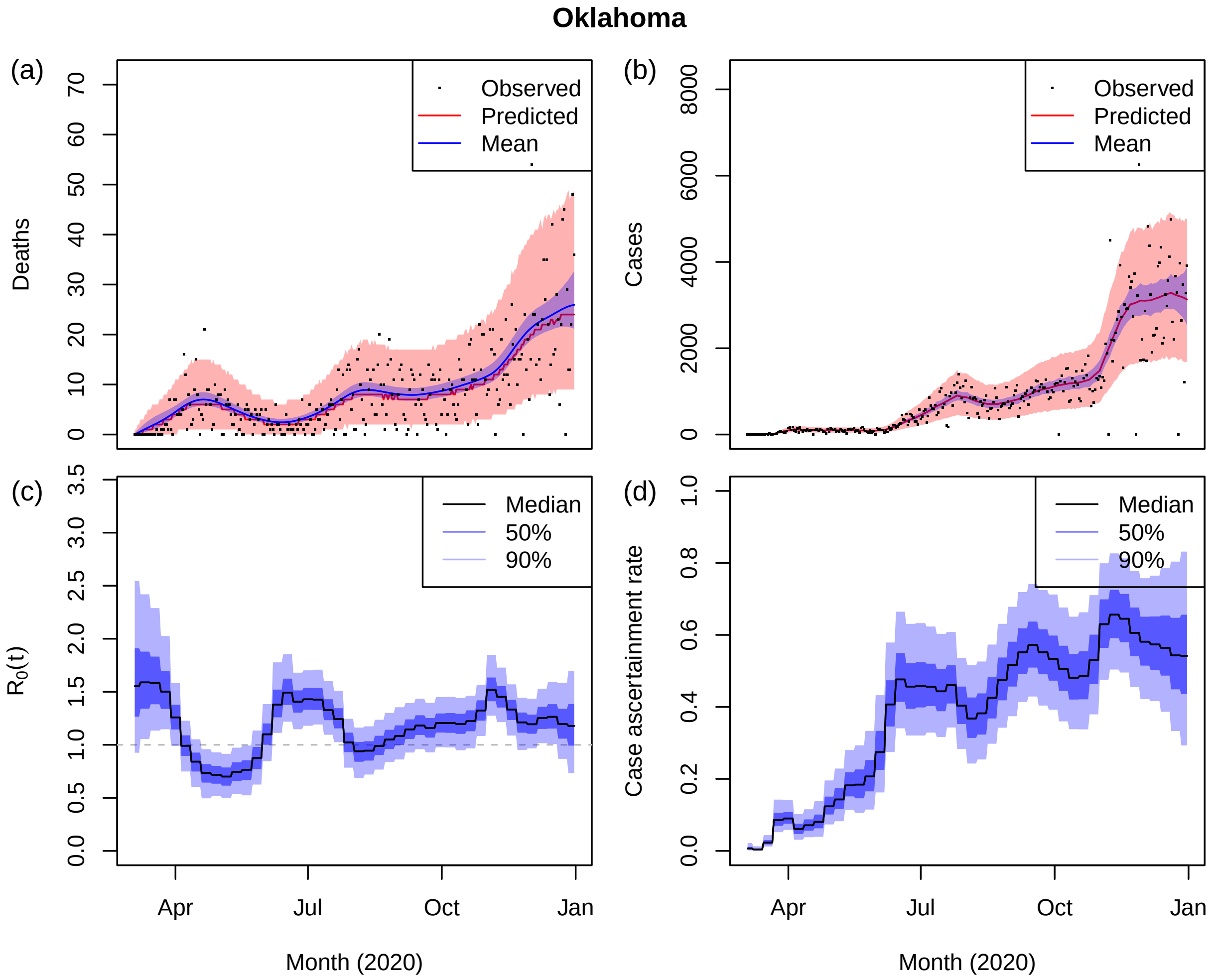

Figure S85: SEIRD model fit to COVID-19 data. **Top panels:** observed (a) deaths $d(t)$ and (b) cases $c(t)$ are plotted in black. Median and 90% credible intervals of the posterior predictive distributions of $d(t)$ and $c(t)$ are in red. Posterior median and 90% credible intervals of the underlying mean parameters $m_D(t)$ and $m_C(t)$ are in blue. **Bottom panels:** posterior median, 50%, and 90% credible intervals for (c) the basic reproduction number $R_0(t)$ and (d) the case ascertainment rate CAR(t).

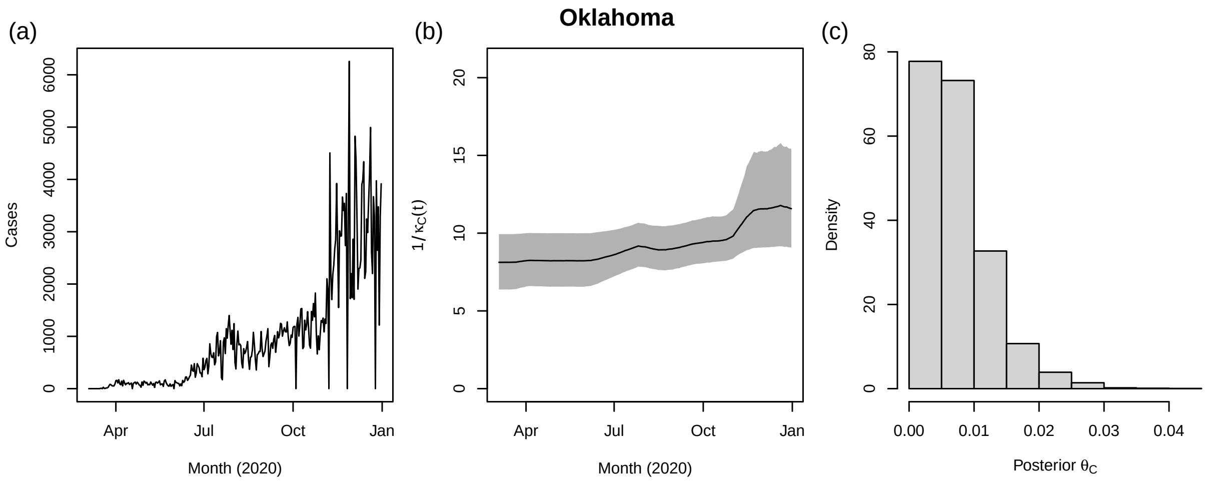

Figure S86: Overdispersion and zero-inflation in state-level clinical case data. (a) Reported cases. (b) Posterior median and 90% credible interval for the time-varying negative binomial overdispersion parameter $\kappa_C(t)^{-1}$. (c) Posterior histogram for the zero-inflation parameter θ_C.

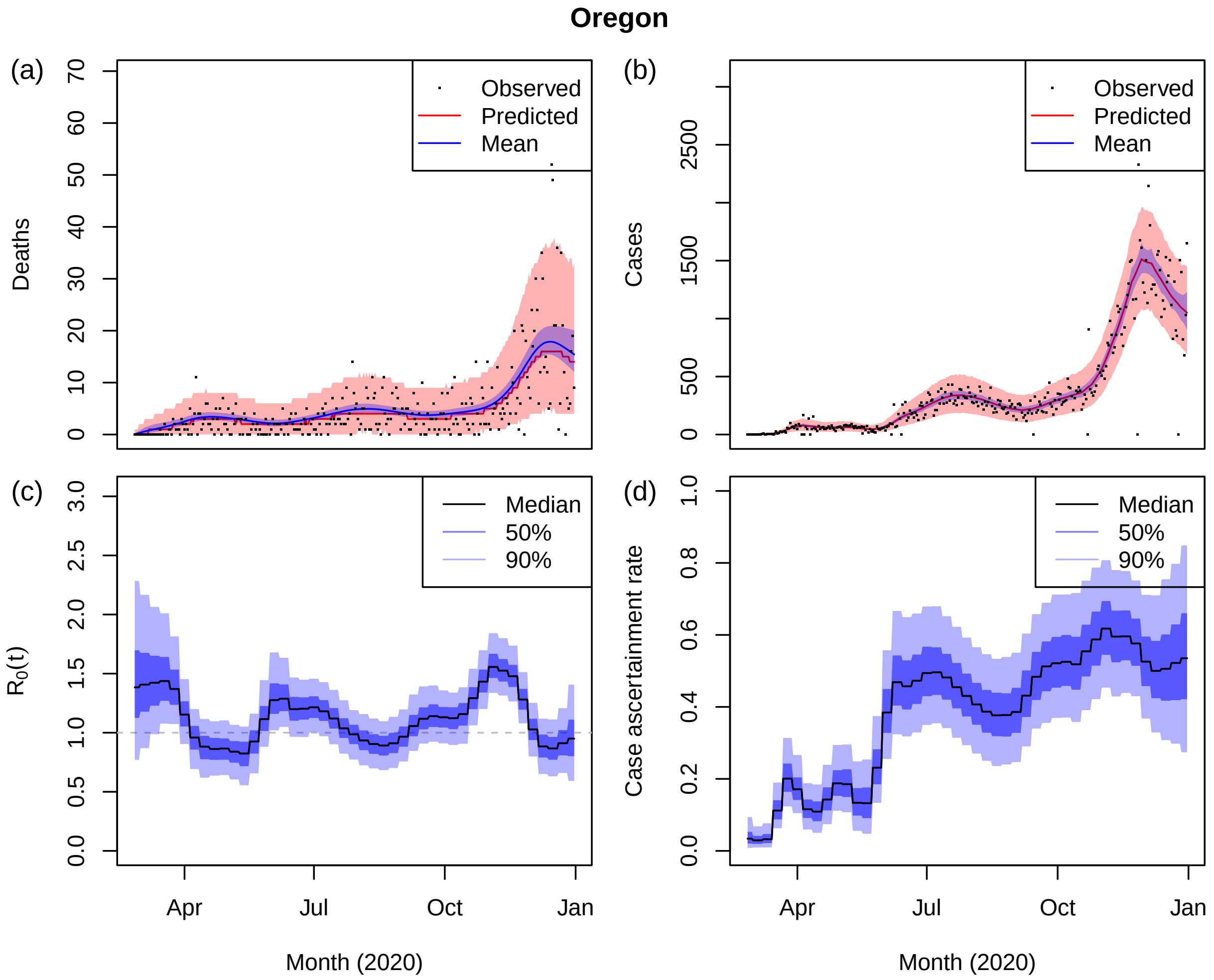

Figure S87: SEIRD model fit to COVID-19 data. **Top panels:** observed (a) deaths $d(t)$ and (b) cases $c(t)$ are plotted in black. Median and 90% credible intervals of the posterior predictive distributions of $d(t)$ and $c(t)$ are in red. Posterior median and 90% credible intervals of the underlying mean parameters $m_D(t)$ and $m_C(t)$ are in blue. **Bottom panels:** posterior median, 50%, and 90% credible intervals for (c) the basic reproduction number $R_0(t)$ and (d) the case ascertainment rate CAR(t).

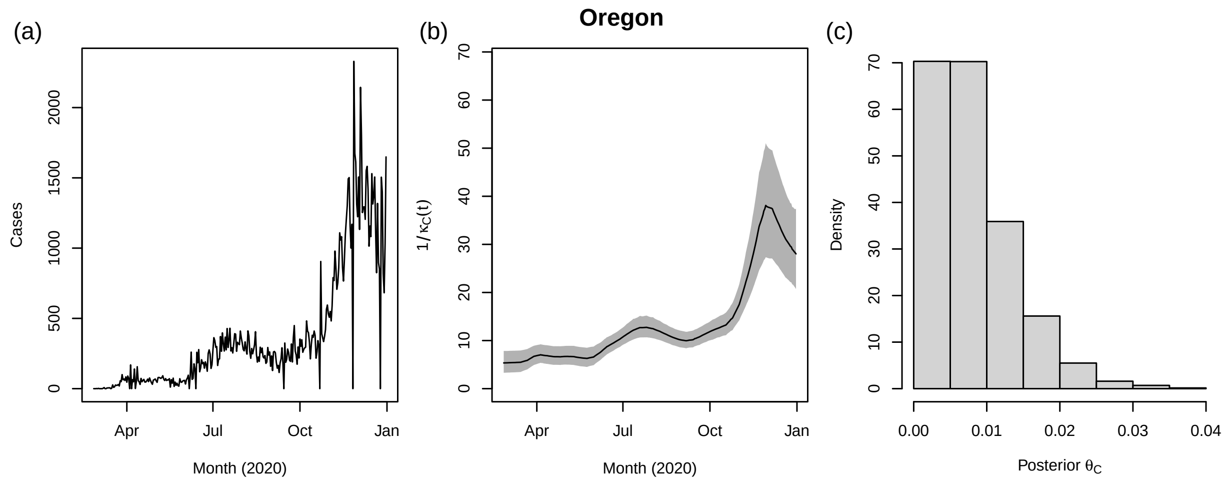

Figure S88: Overdispersion and zero-inflation in state-level clinical case data. (a) Reported cases. (b) Posterior median and 90% credible interval for the time-varying negative binomial overdispersion parameter $\kappa_C(t)^{-1}$. (c) Posterior histogram for the zero-inflation parameter θ_C.

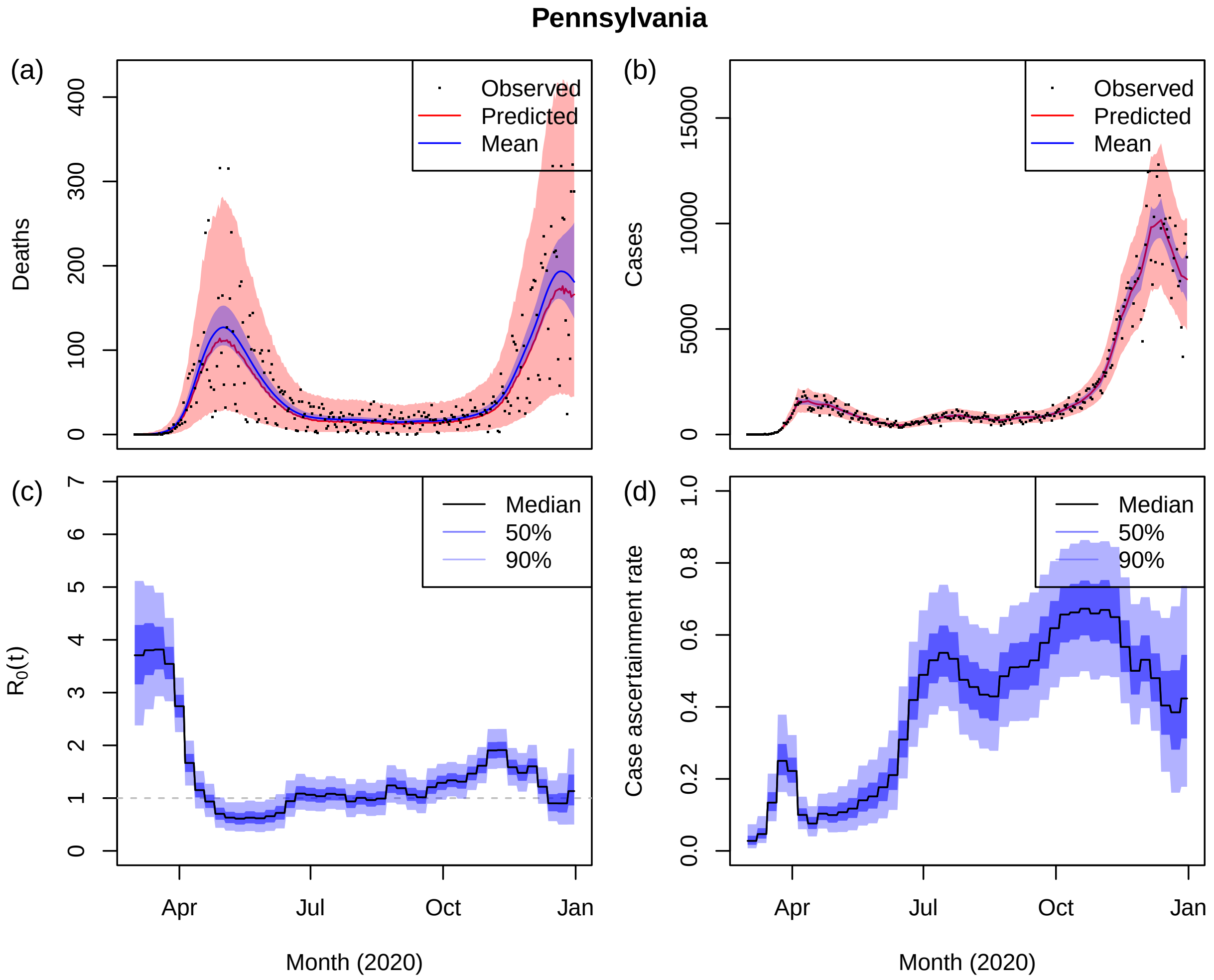

Figure S89: SEIRD model fit to COVID-19 data. **Top panels:** observed (a) deaths $d(t)$ and (b) cases $c(t)$ are plotted in black. Median and 90% credible intervals of the posterior predictive distributions of $d(t)$ and $c(t)$ are in red. Posterior median and 90% credible intervals of the underlying mean parameters $m_D(t)$ and $m_C(t)$ are in blue. **Bottom panels:** posterior median, 50%, and 90% credible intervals for (c) the basic reproduction number $R_0(t)$ and (d) the case ascertainment rate $\text{CAR}(t)$.

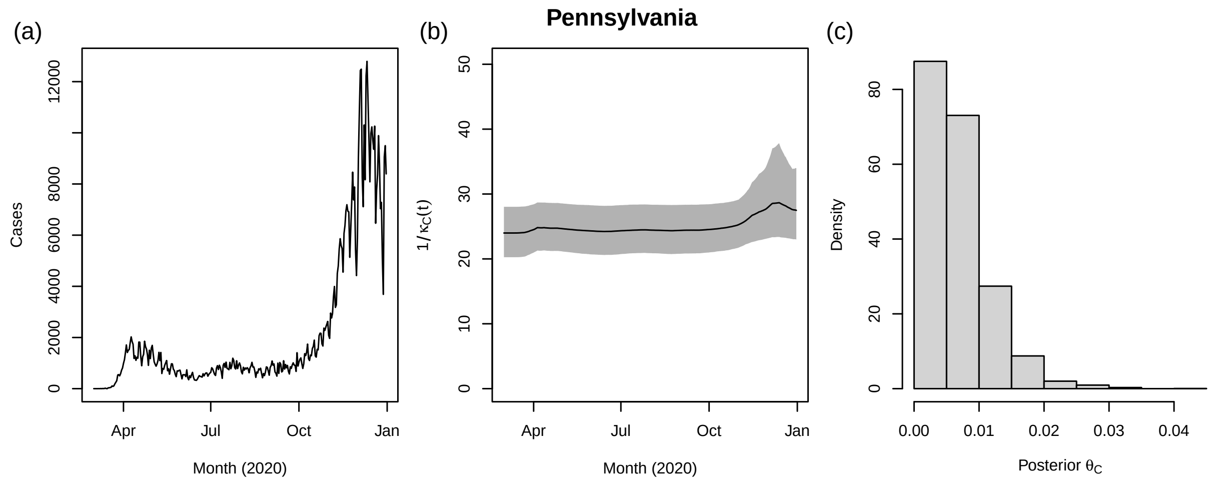

Figure S90: Overdispersion and zero-inflation in state-level clinical case data. (a) Reported cases. (b) Posterior median and 90% credible interval for the time-varying negative binomial overdispersion parameter $\kappa_C(t)^{-1}$. (c) Posterior histogram for the zero-inflation parameter θ_C.

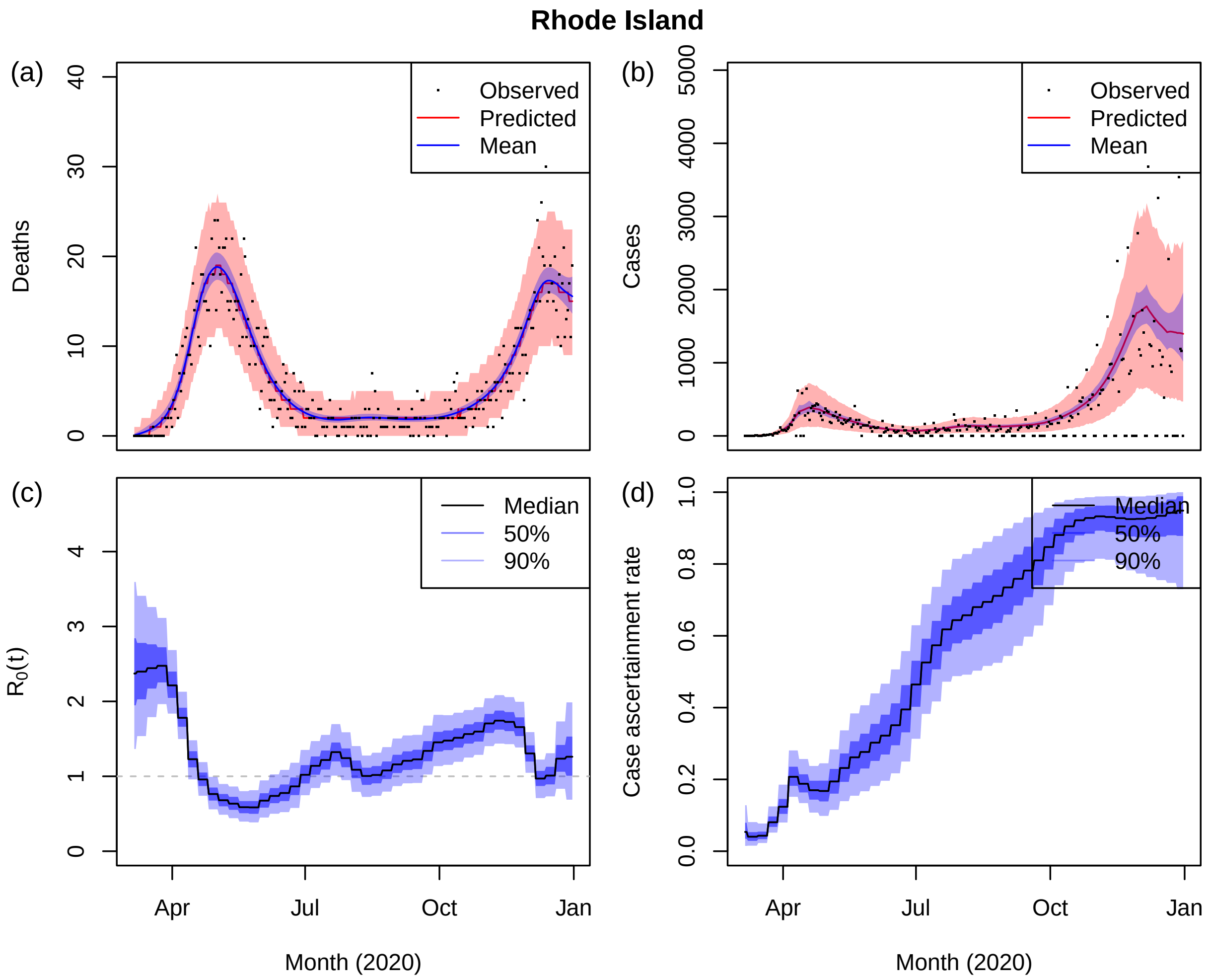

Figure S91: SEIRD model fit to COVID-19 data. **Top panels:** observed (a) deaths $d(t)$ and (b) cases $c(t)$ are plotted in black. Median and 90% credible intervals of the posterior predictive distributions of $d(t)$ and $c(t)$ are in red. Posterior median and 90% credible intervals of the underlying mean parameters $m_D(t)$ and $m_C(t)$ are in blue. **Bottom panels:** posterior median, 50%, and 90% credible intervals for (c) the basic reproduction number $R_0(t)$ and (d) the case ascertainment rate $\text{CAR}(t)$.

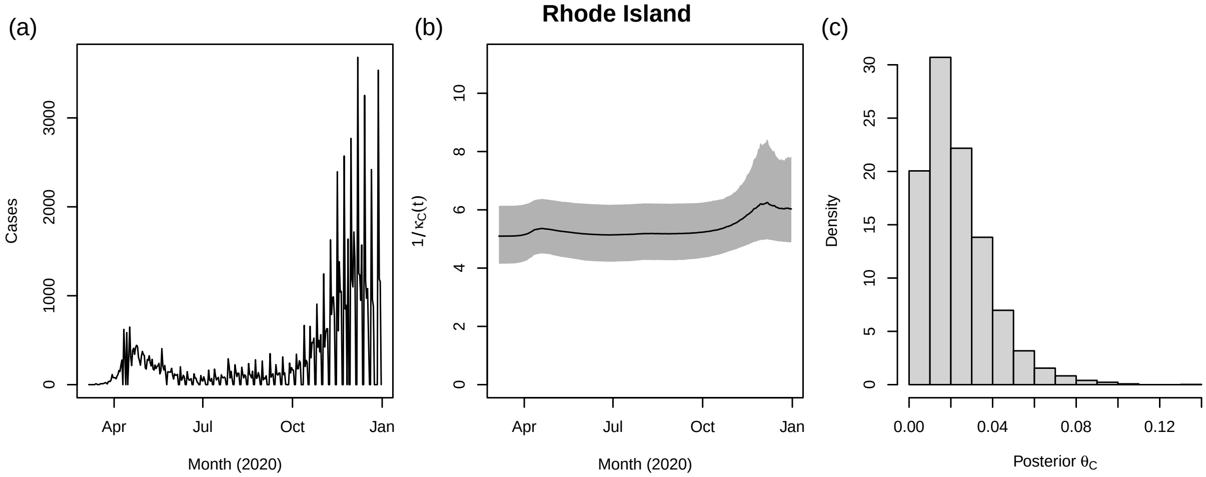

Figure S92: Overdispersion and zero-inflation in state-level clinical case data. (a) Reported cases. (b) Posterior median and 90% credible interval for the time-varying negative binomial overdispersion parameter $\kappa_C(t)^{-1}$. (c) Posterior histogram for the zero-inflation parameter θ_C.

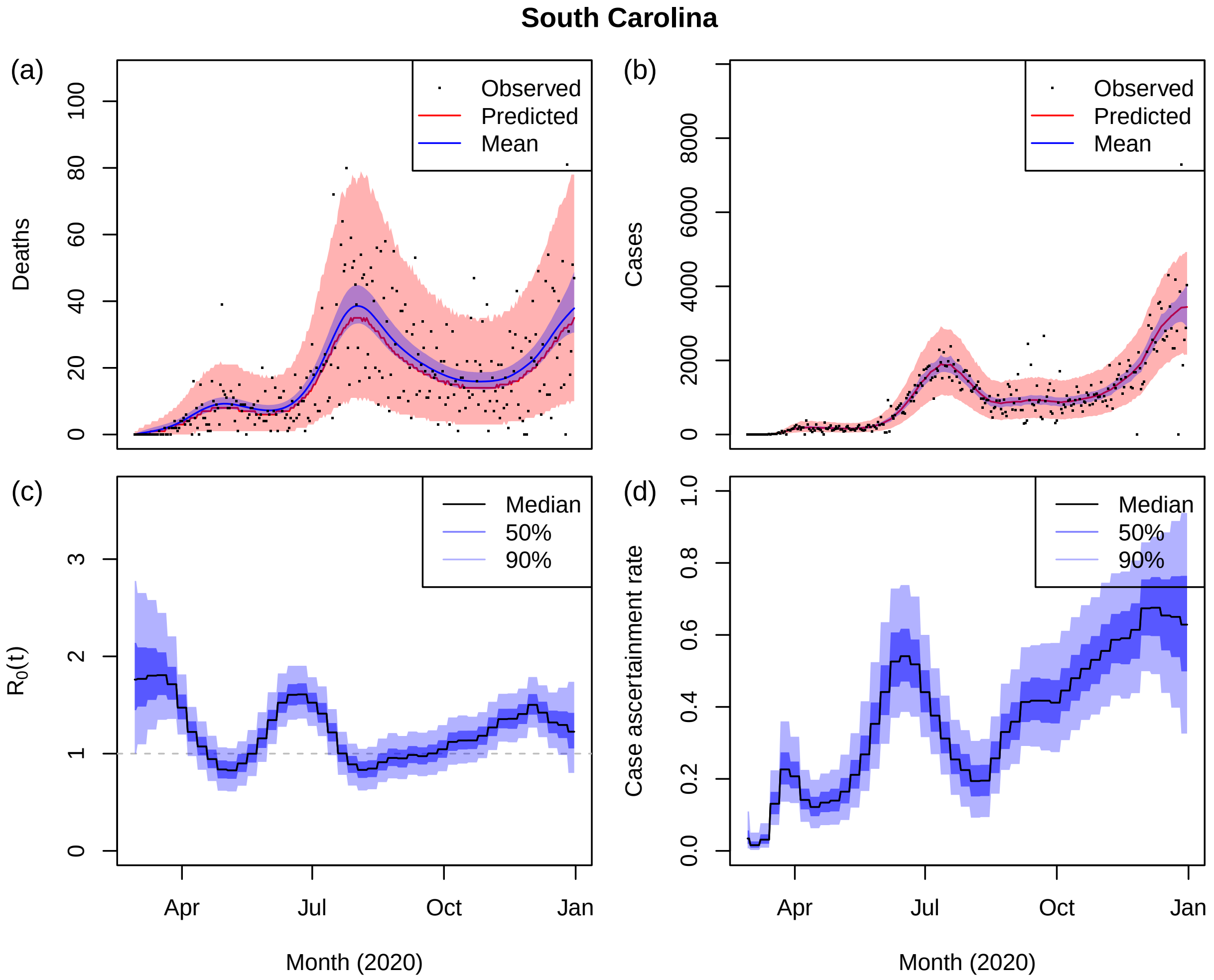

Figure S93: SEIRD model fit to COVID-19 data. **Top panels:** observed (a) deaths $d(t)$ and (b) cases $c(t)$ are plotted in black. Median and 90% credible intervals of the posterior predictive distributions of $d(t)$ and $c(t)$ are in red. Posterior median and 90% credible intervals of the underlying mean parameters $m_D(t)$ and $m_C(t)$ are in blue. **Bottom panels:** posterior median, 50%, and 90% credible intervals for (c) the basic reproduction number $R_0(t)$ and (d) the case ascertainment rate CAR(t).

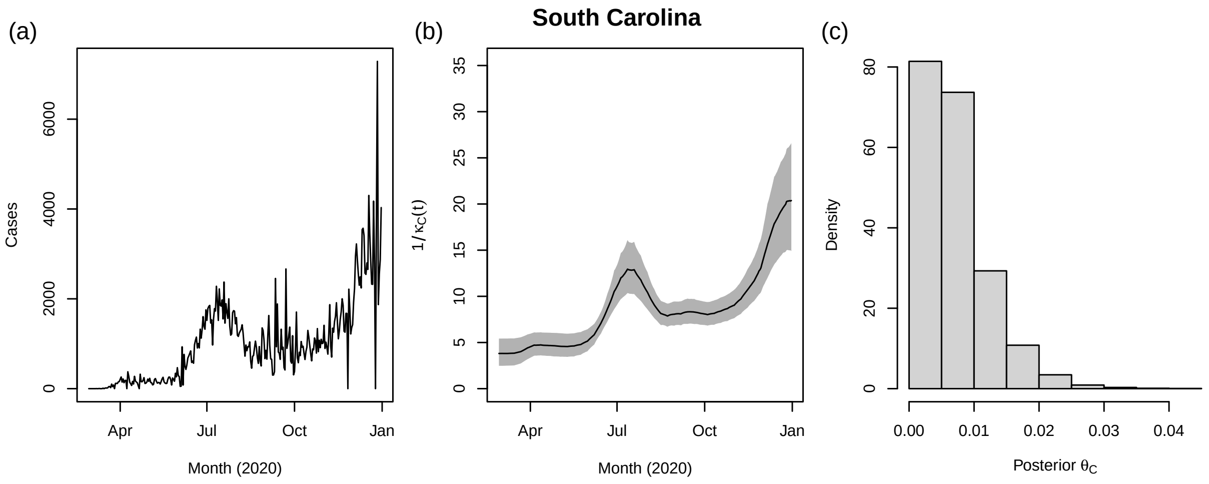

Figure S94: Overdispersion and zero-inflation in state-level clinical case data. (a) Reported cases. (b) Posterior median and 90% credible interval for the time-varying negative binomial overdispersion parameter $\kappa_C(t)^{-1}$. (c) Posterior histogram for the zero-inflation parameter θ_C.

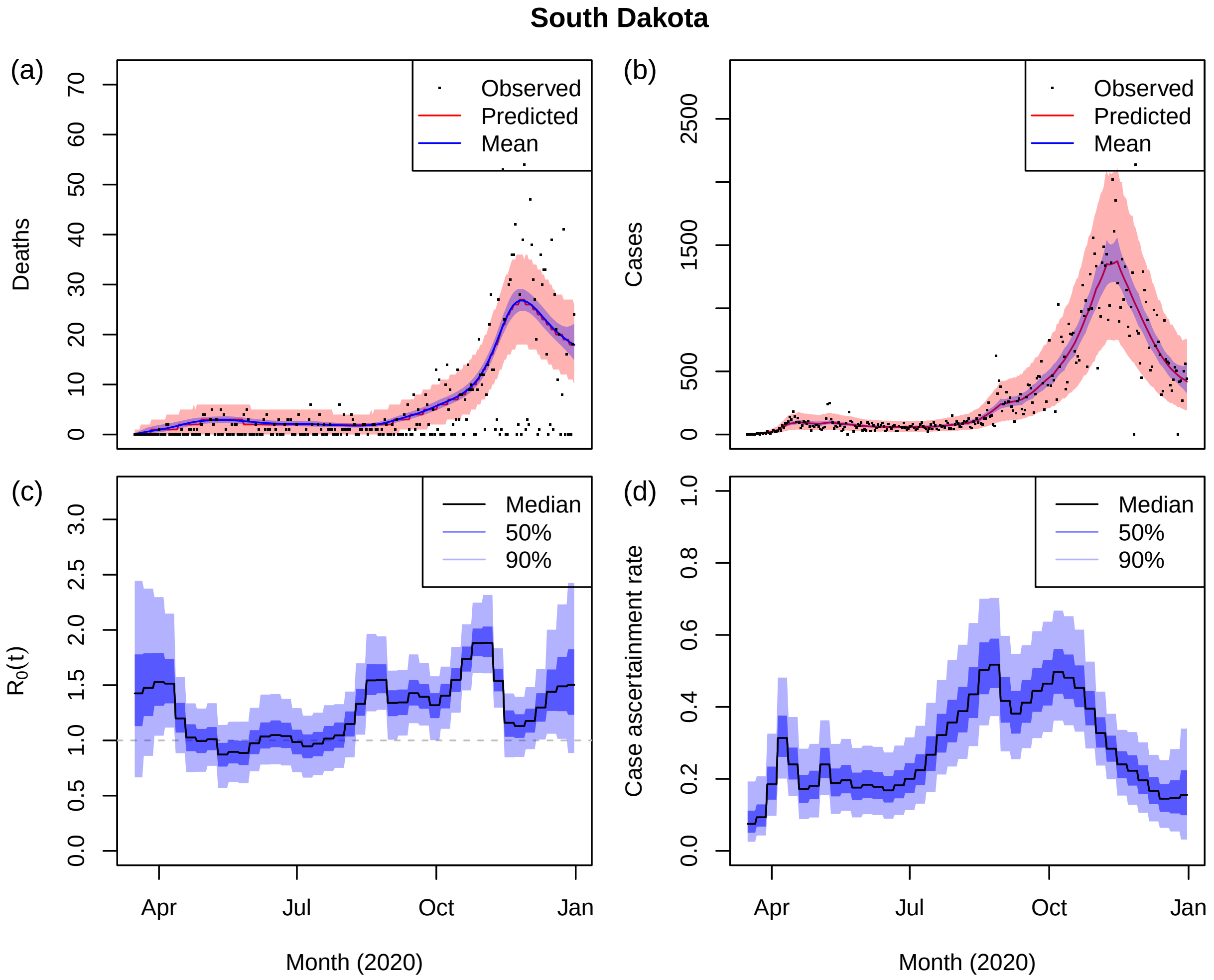

Figure S95: SEIRD model fit to COVID-19 data. **Top panels:** observed (a) deaths $d(t)$ and (b) cases $c(t)$ are plotted in black. Median and 90% credible intervals of the posterior predictive distributions of $d(t)$ and $c(t)$ are in red. Posterior median and 90% credible intervals of the underlying mean parameters $m_D(t)$ and $m_C(t)$ are in blue. **Bottom panels:** posterior median, 50%, and 90% credible intervals for (c) the basic reproduction number $R_0(t)$ and (d) the case ascertainment rate CAR(t).

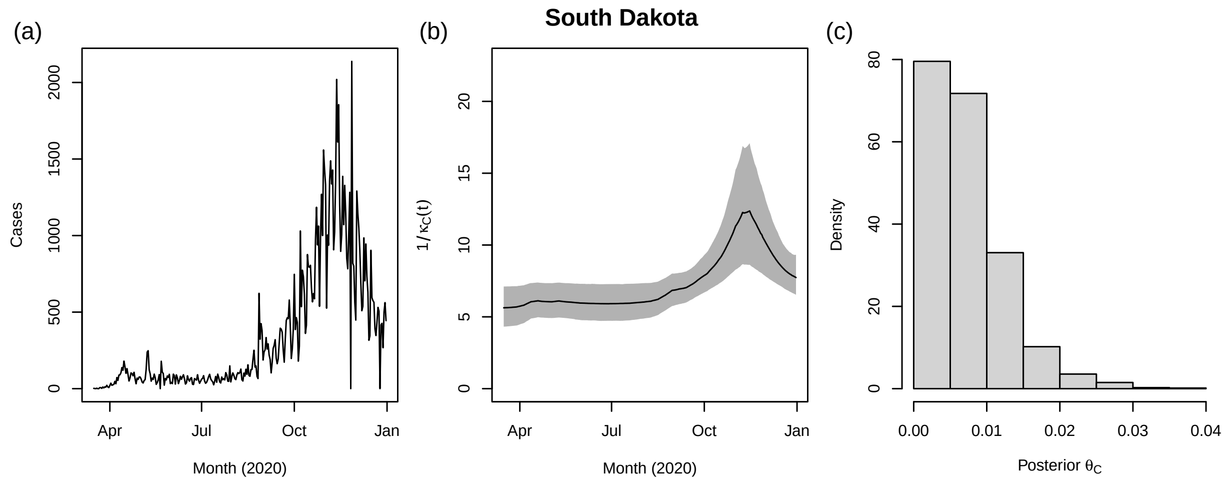

Figure S96: Overdispersion and zero-inflation in state-level clinical case data. (a) Reported cases. (b) Posterior median and 90% credible interval for the time-varying negative binomial overdispersion parameter $\kappa_C(t)^{-1}$. (c) Posterior histogram for the zero-inflation parameter θ_C.

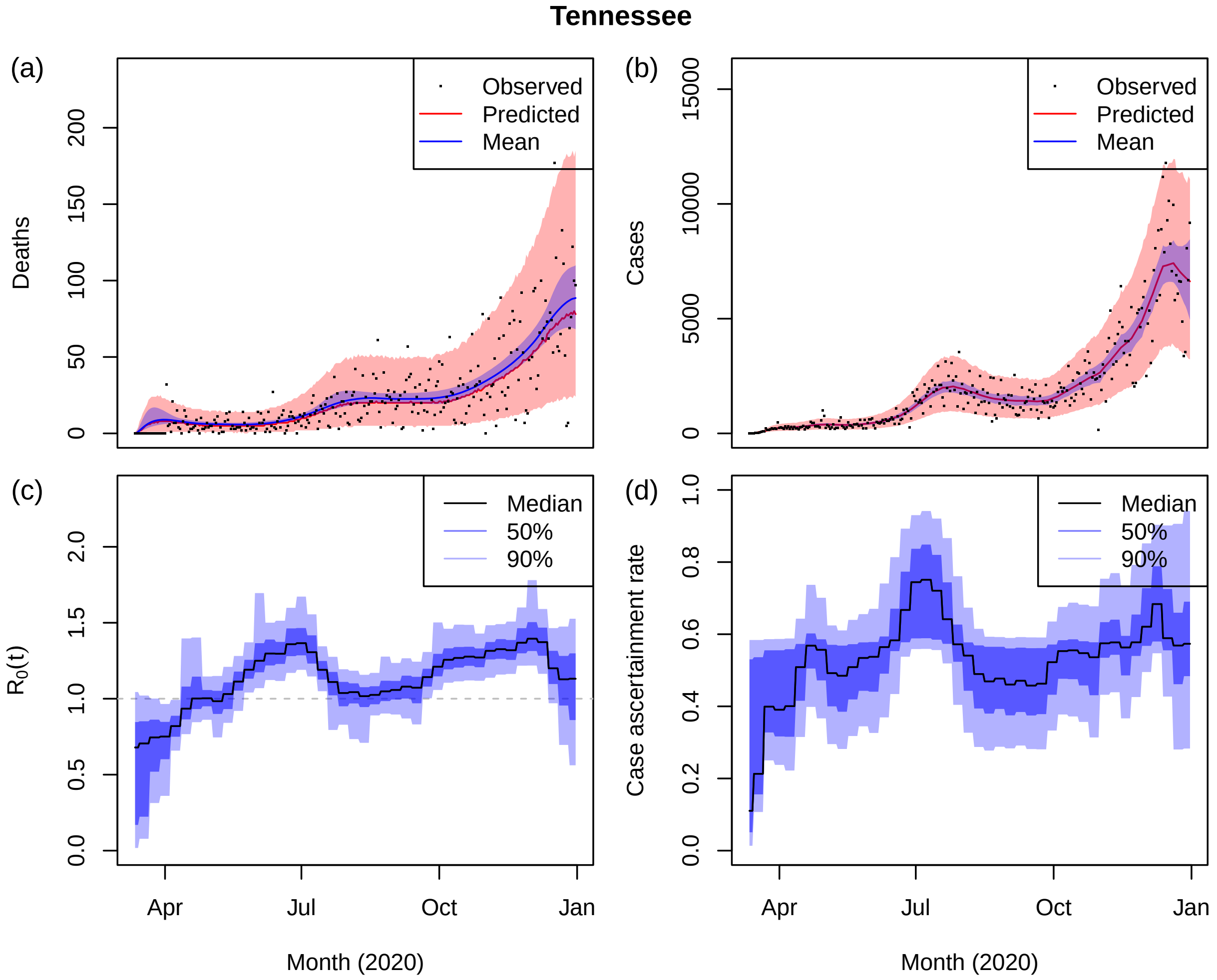

Figure S97: SEIRD model fit to COVID-19 data. **Top panels:** observed (a) deaths $d(t)$ and (b) cases $c(t)$ are plotted in black. Median and 90% credible intervals of the posterior predictive distributions of $d(t)$ and $c(t)$ are in red. Posterior median and 90% credible intervals of the underlying mean parameters $m_D(t)$ and $m_C(t)$ are in blue. **Bottom panels:** posterior median, 50%, and 90% credible intervals for (c) the basic reproduction number $R_0(t)$ and (d) the case ascertainment rate CAR(t).

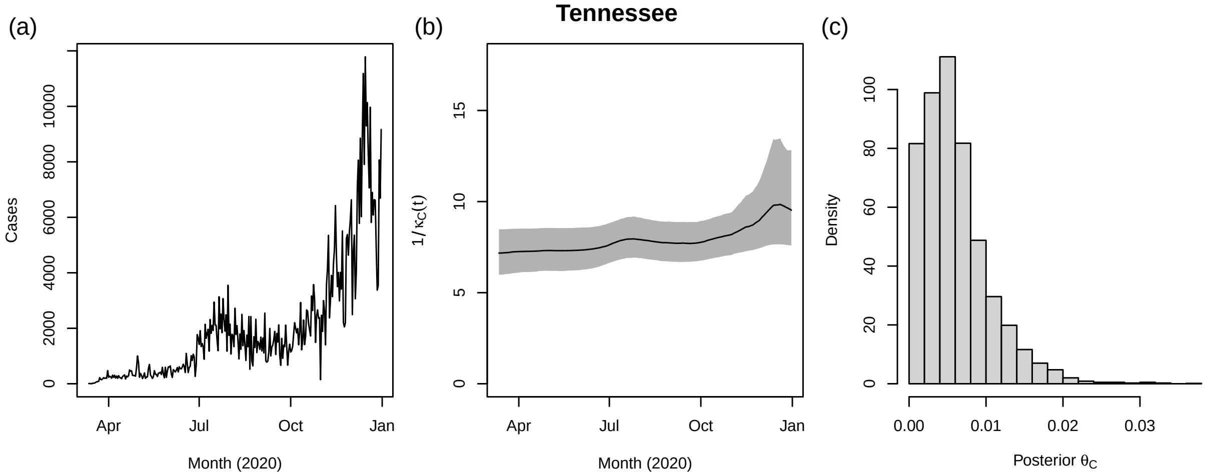

Figure S98: Overdispersion and zero-inflation in state-level clinical case data. (a) Reported cases. (b) Posterior median and 90% credible interval for the time-varying negative binomial overdispersion parameter $\kappa_C(t)^{-1}$. (c) Posterior histogram for the zero-inflation parameter θ_C.

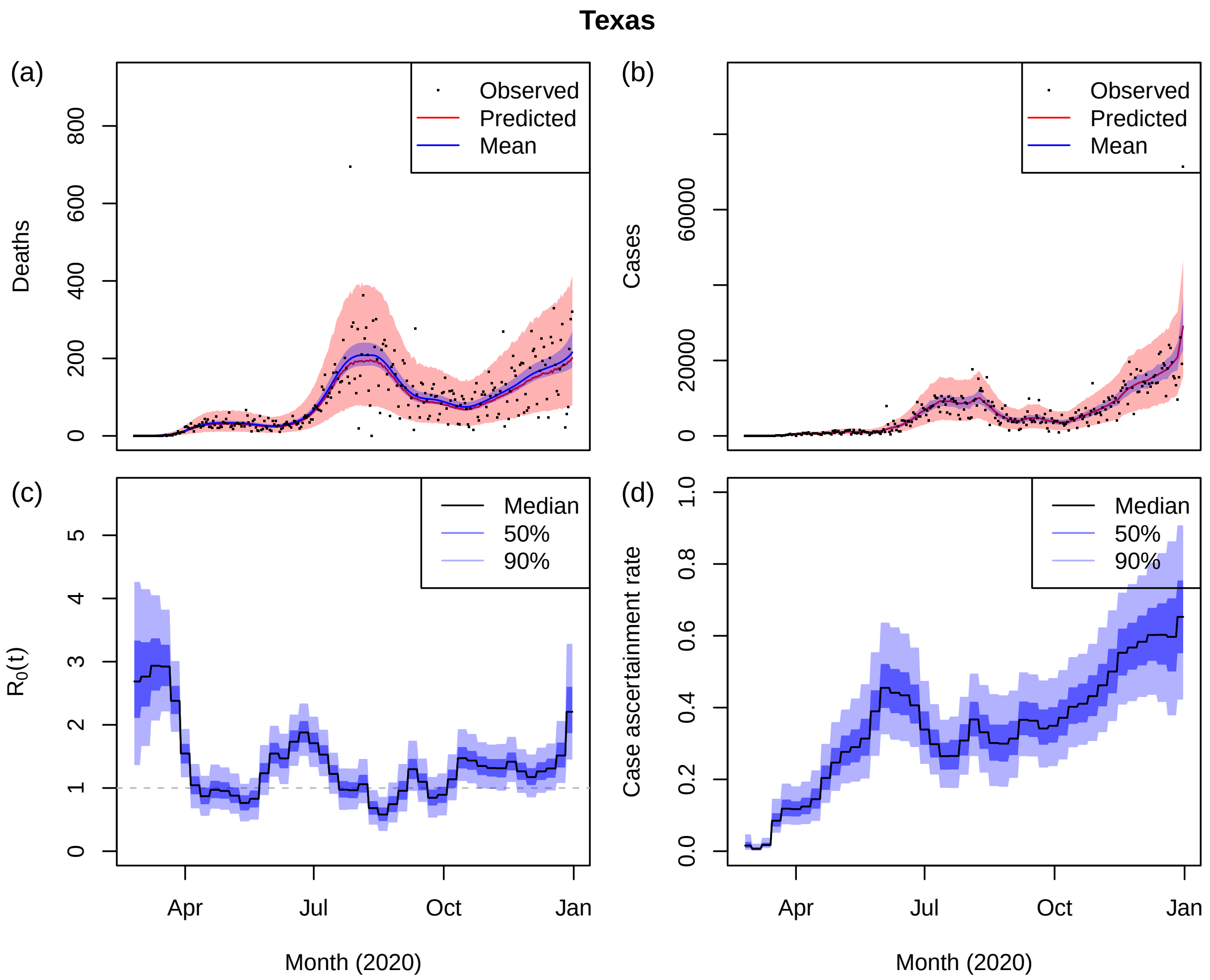

Figure S99: SEIRD model fit to COVID-19 data. **Top panels:** observed (a) deaths $d(t)$ and (b) cases $c(t)$ are plotted in black. Median and 90% credible intervals of the posterior predictive distributions of $d(t)$ and $c(t)$ are in red. Posterior median and 90% credible intervals of the underlying mean parameters $m_D(t)$ and $m_C(t)$ are in blue. **Bottom panels:** posterior median, 50%, and 90% credible intervals for (c) the basic reproduction number $R_0(t)$ and (d) the case ascertainment rate CAR(t).

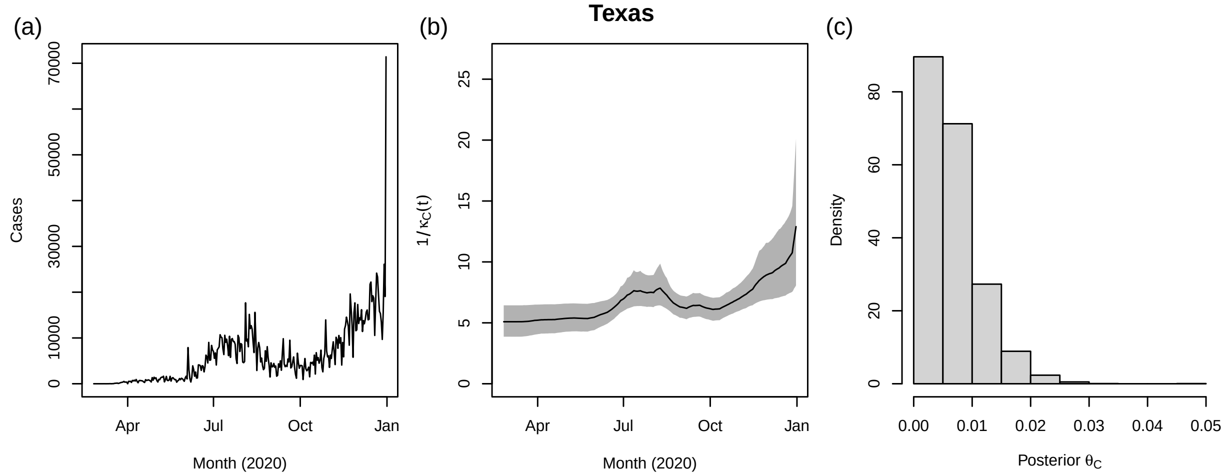

Figure S100: Overdispersion and zero-inflation in state-level clinical case data. (a) Reported cases. (b) Posterior median and 90% credible interval for the time-varying negative binomial overdispersion parameter $\kappa_C(t)^{-1}$. (c) Posterior histogram for the zero-inflation parameter θ_C.

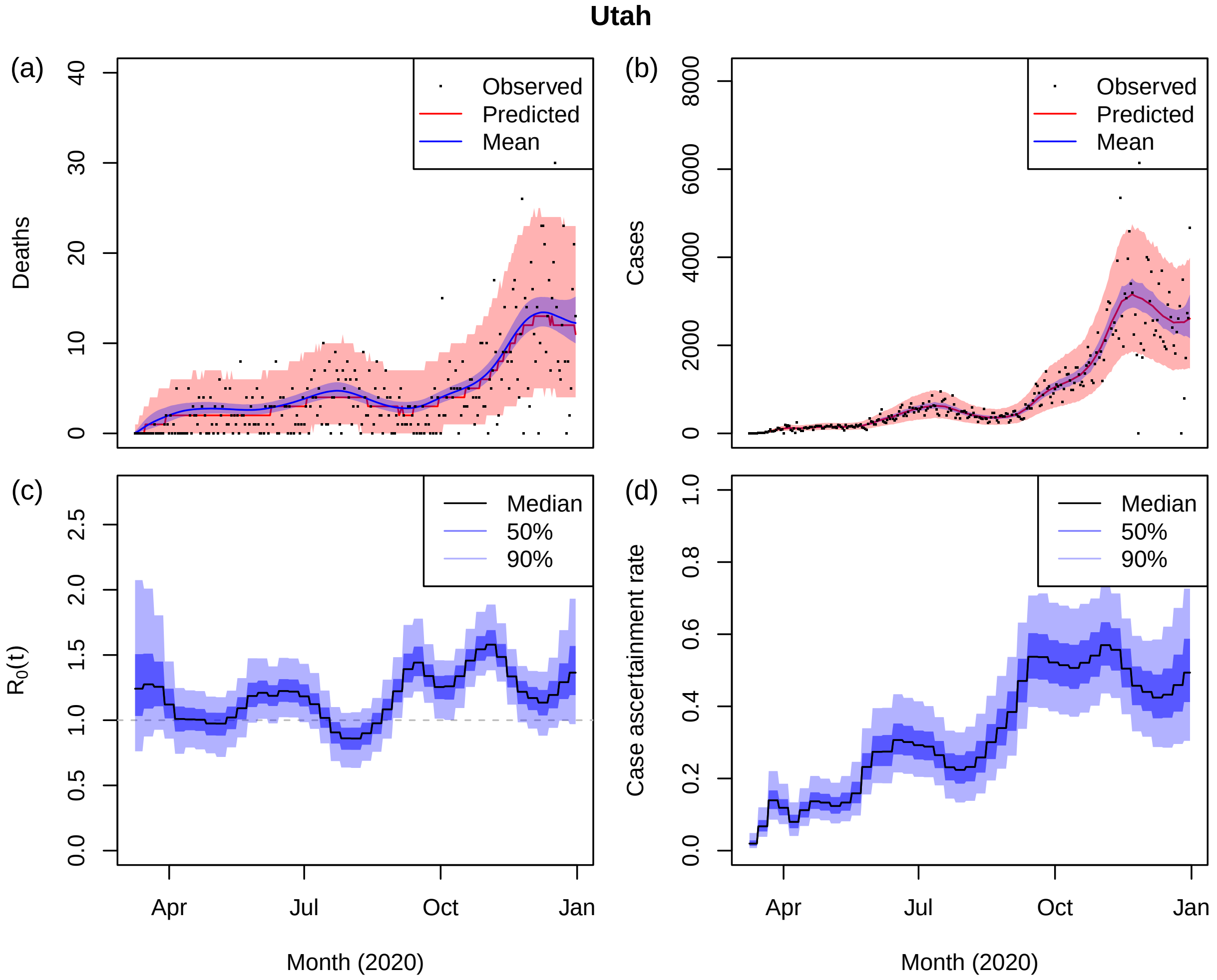

Figure S101: SEIRD model fit to COVID-19 data. **Top panels:** observed (a) deaths $d(t)$ and (b) cases $c(t)$ are plotted in black. Median and 90% credible intervals of the posterior predictive distributions of $d(t)$ and $c(t)$ are in red. Posterior median and 90% credible intervals of the underlying mean parameters $m_D(t)$ and $m_C(t)$ are in blue. **Bottom panels:** posterior median, 50%, and 90% credible intervals for (c) the basic reproduction number $R_0(t)$ and (d) the case ascertainment rate $\text{CAR}(t)$.

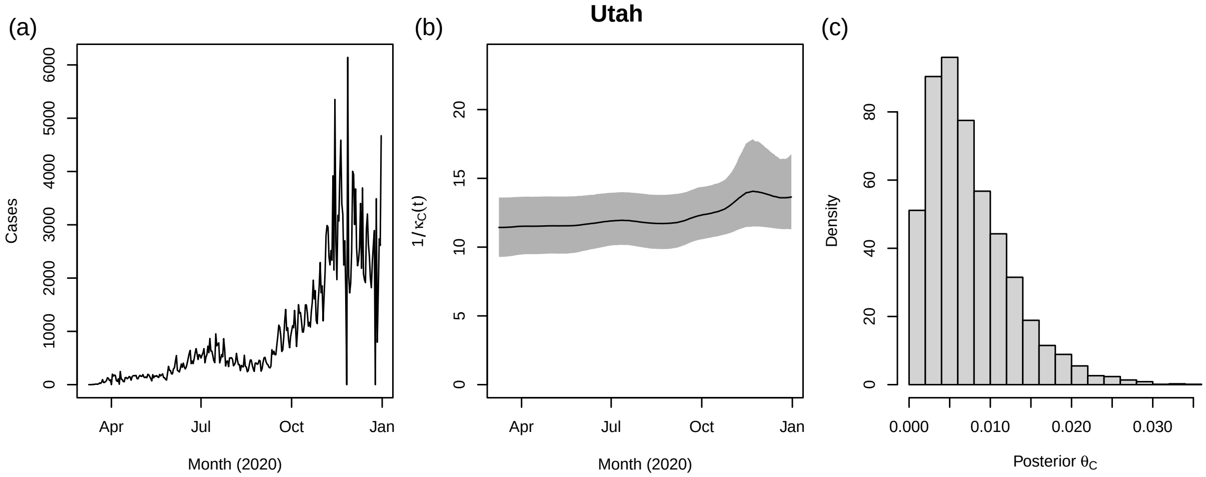

Figure S102: Overdispersion and zero-inflation in state-level clinical case data. (a) Reported cases. (b) Posterior median and 90% credible interval for the time-varying negative binomial overdispersion parameter $\kappa_C(t)^{-1}$. (c) Posterior histogram for the zero-inflation parameter θ_C.

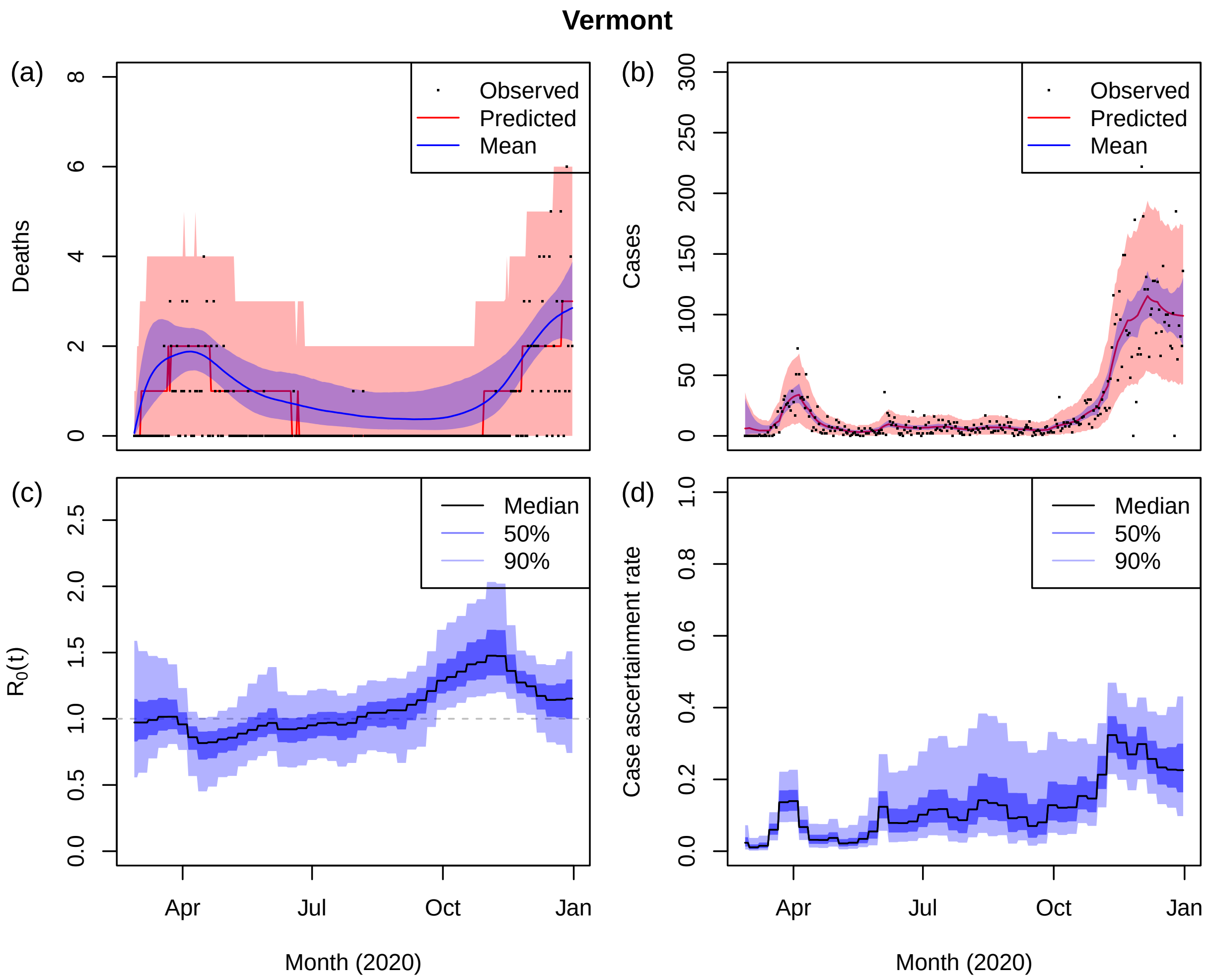

Figure S103: SEIRD model fit to COVID-19 data. **Top panels:** observed (a) deaths $d(t)$ and (b) cases $c(t)$ are plotted in black. Median and 90% credible intervals of the posterior predictive distributions of $d(t)$ and $c(t)$ are in red. Posterior median and 90% credible intervals of the underlying mean parameters $m_D(t)$ and $m_C(t)$ are in blue. **Bottom panels:** posterior median, 50%, and 90% credible intervals for (c) the basic reproduction number $R_0(t)$ and (d) the case ascertainment rate $\mathrm{CAR}(t)$.

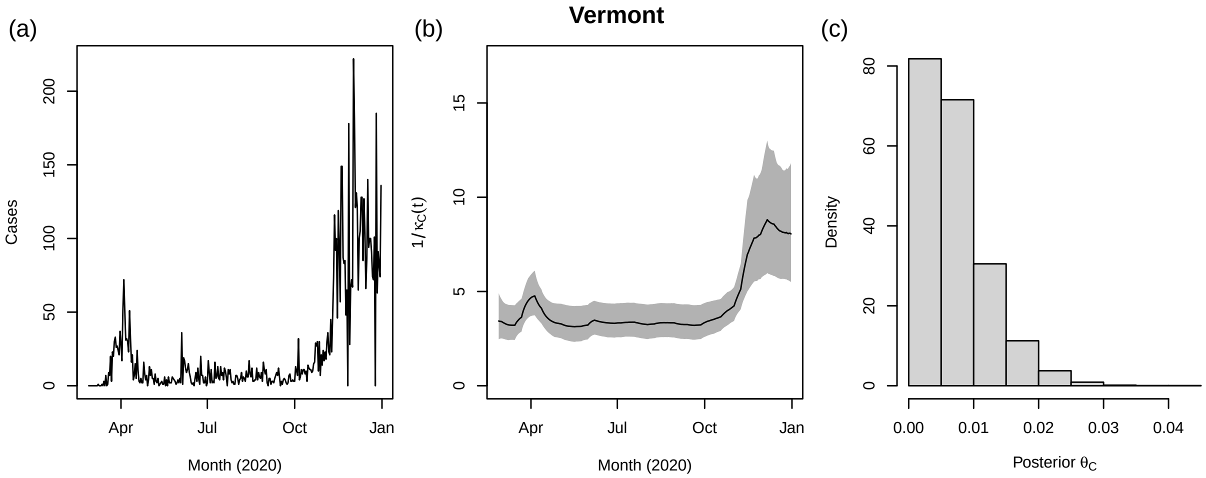

Figure S104: Overdispersion and zero-inflation in state-level clinical case data. (a) Reported cases. (b) Posterior median and 90% credible interval for the time-varying negative binomial overdispersion parameter $\kappa_C(t)^{-1}$. (c) Posterior histogram for the zero-inflation parameter θ_C.

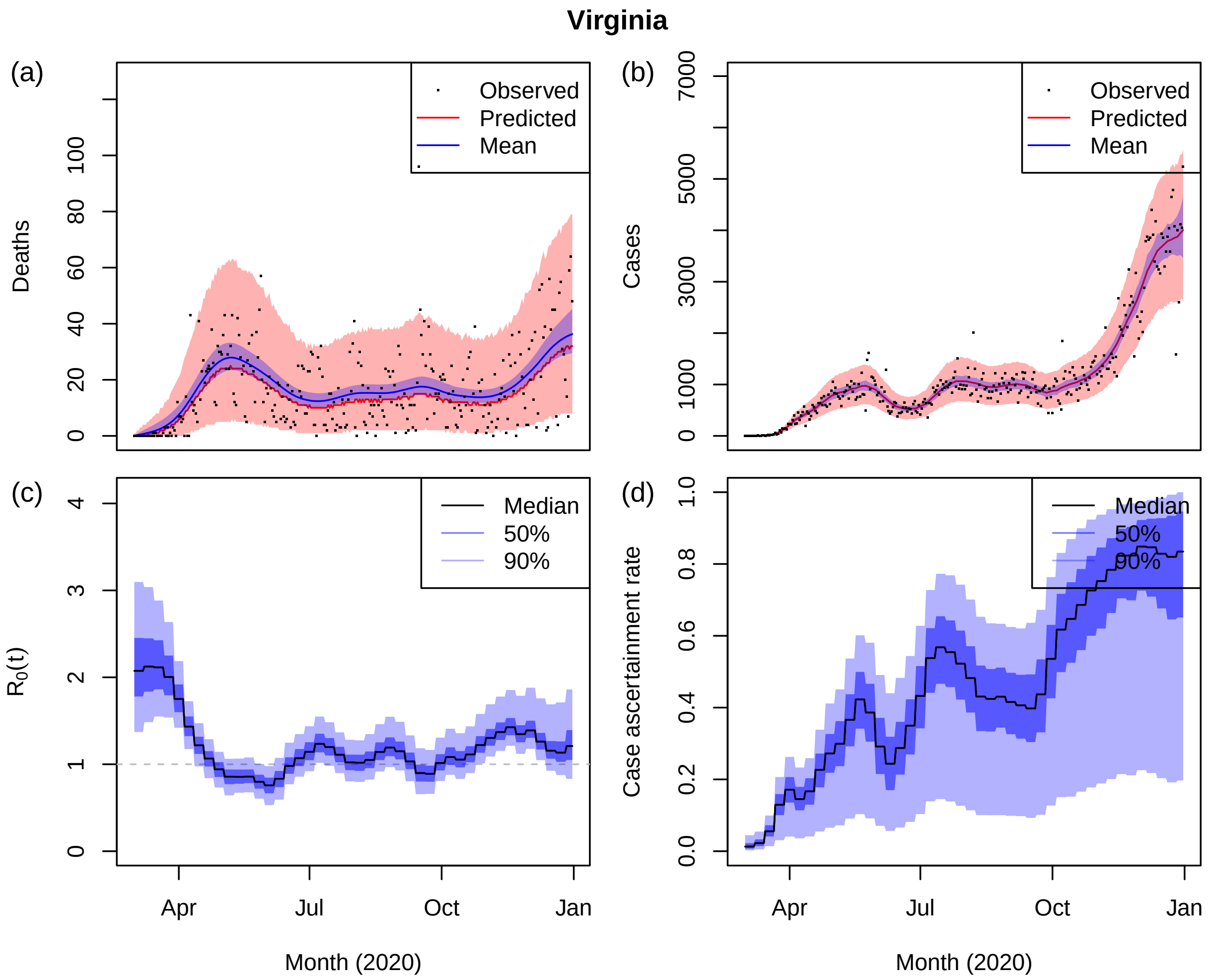

Figure S105: SEIRD model fit to COVID-19 data. **Top panels:** observed (a) deaths $d(t)$ and (b) cases $c(t)$ are plotted in black. Median and 90% credible intervals of the posterior predictive distributions of $d(t)$ and $c(t)$ are in red. Posterior median and 90% credible intervals of the underlying mean parameters $m_D(t)$ and $m_C(t)$ are in blue. **Bottom panels:** posterior median, 50%, and 90% credible intervals for (c) the basic reproduction number $R_0(t)$ and (d) the case ascertainment rate CAR(t).

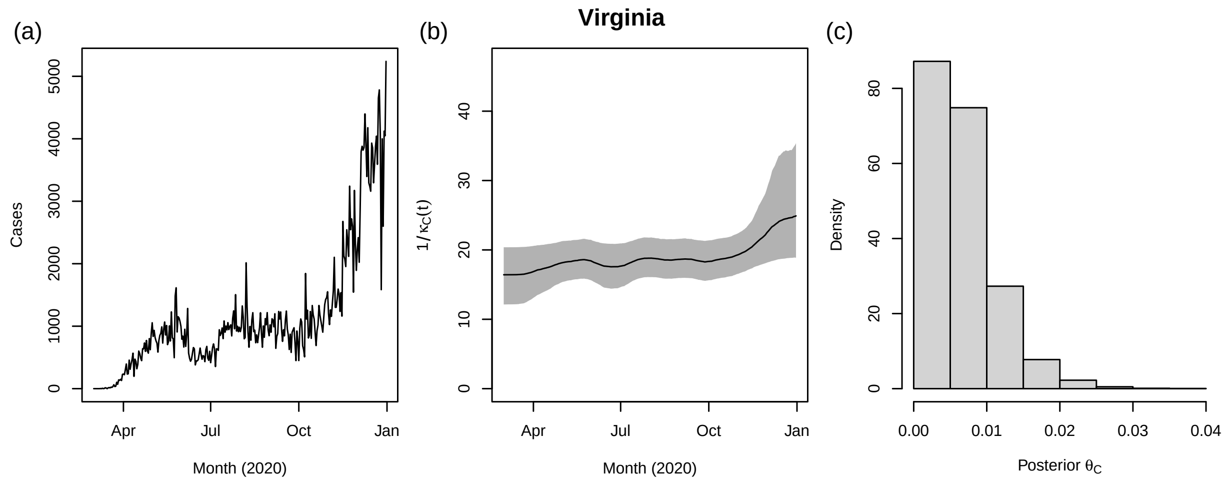

Figure S106: Overdispersion and zero-inflation in state-level clinical case data. (a) Reported cases. (b) Posterior median and 90% credible interval for the time-varying negative binomial overdispersion parameter $\kappa_C(t)^{-1}$. (c) Posterior histogram for the zero-inflation parameter θ_C.

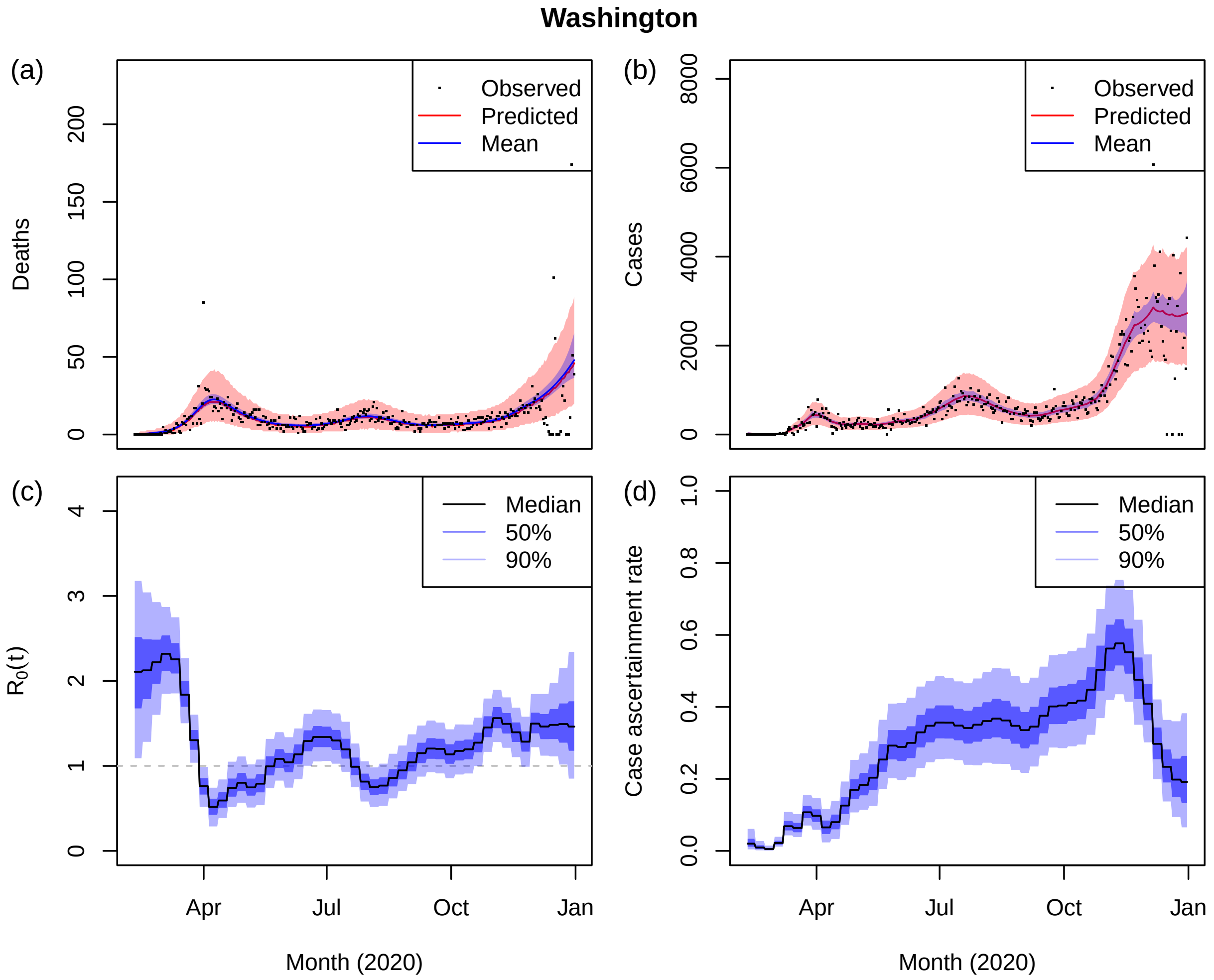

Figure S107: SEIRD model fit to COVID-19 data. **Top panels:** observed (a) deaths $d(t)$ and (b) cases $c(t)$ are plotted in black. Median and 90% credible intervals of the posterior predictive distributions of $d(t)$ and $c(t)$ are in red. Posterior median and 90% credible intervals of the underlying mean parameters $m_D(t)$ and $m_C(t)$ are in blue. **Bottom panels:** posterior median, 50%, and 90% credible intervals for (c) the basic reproduction number $R_0(t)$ and (d) the case ascertainment rate $\mathrm{CAR}(t)$.

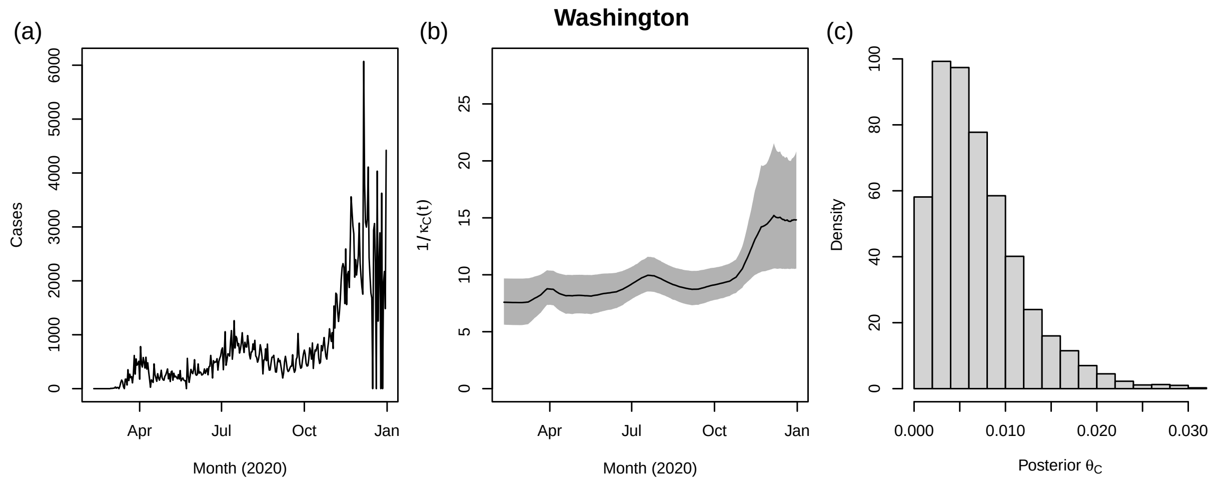

Figure S108: Overdispersion and zero-inflation in state-level clinical case data. (a) Reported cases. (b) Posterior median and 90% credible interval for the time-varying negative binomial overdispersion parameter $\kappa_C(t)^{-1}$. (c) Posterior histogram for the zero-inflation parameter θ_C.

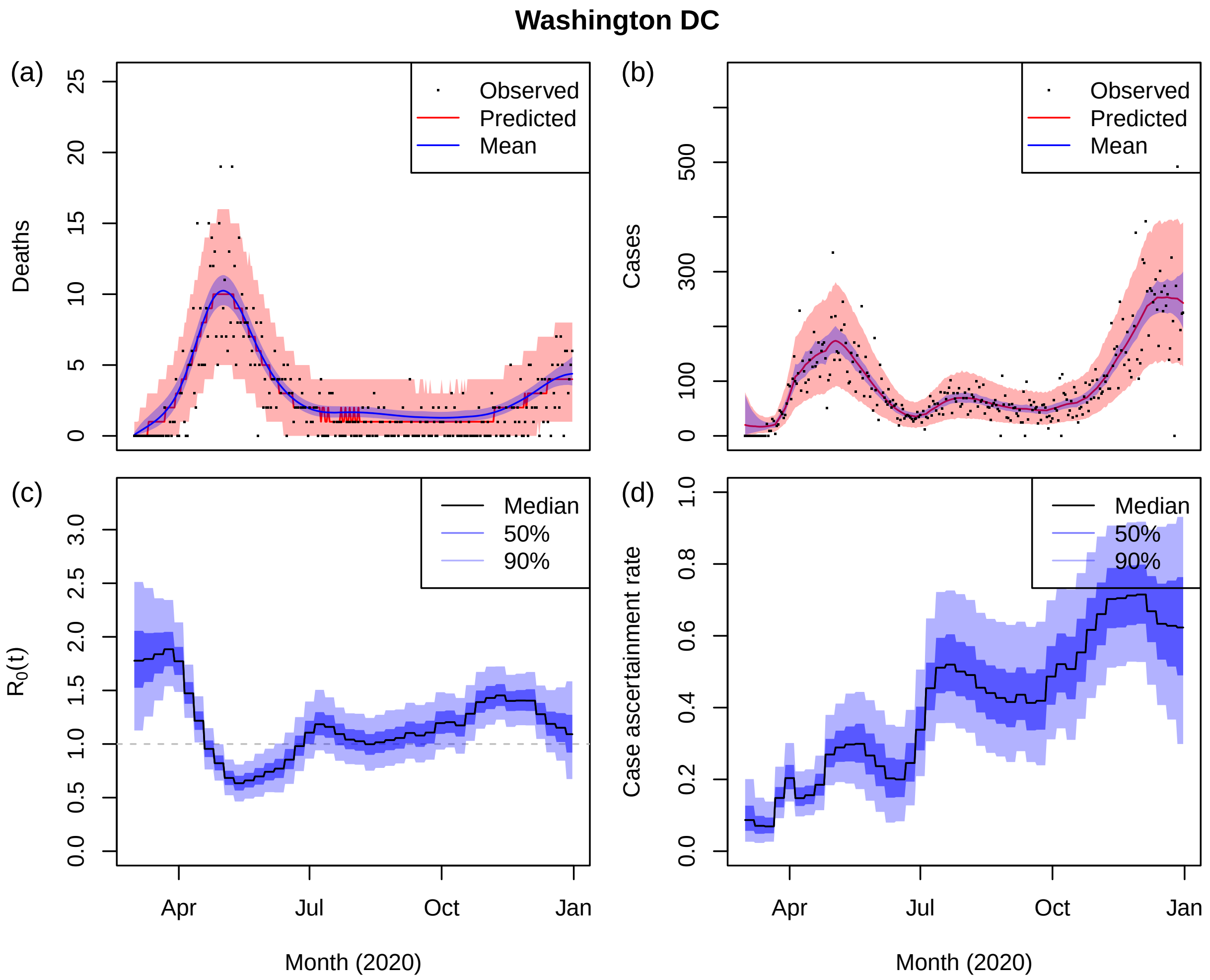

Figure S109: SEIRD model fit to COVID-19 data. **Top panels:** observed (a) deaths $d(t)$ and (b) cases $c(t)$ are plotted in black. Median and 90% credible intervals of the posterior predictive distributions of $d(t)$ and $c(t)$ are in red. Posterior median and 90% credible intervals of the underlying mean parameters $m_D(t)$ and $m_C(t)$ are in blue. **Bottom panels:** posterior median, 50%, and 90% credible intervals for (c) the basic reproduction number $R_0(t)$ and (d) the case ascertainment rate $\text{CAR}(t)$.

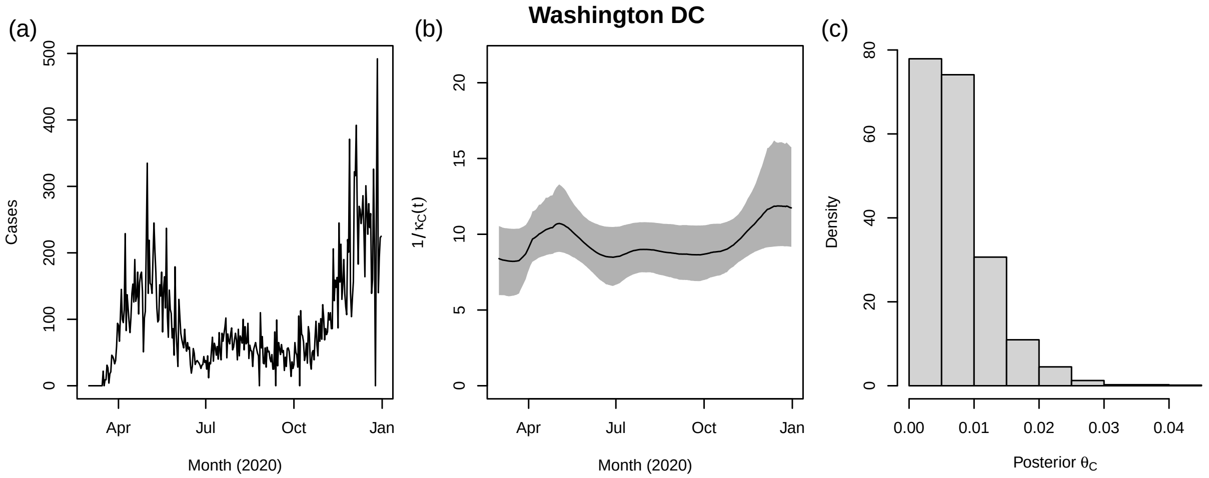

Figure S110: Overdispersion and zero-inflation in state-level clinical case data. (a) Reported cases. (b) Posterior median and 90% credible interval for the time-varying negative binomial overdispersion parameter $\kappa_C(t)^{-1}$. (c) Posterior histogram for the zero-inflation parameter θ_C.

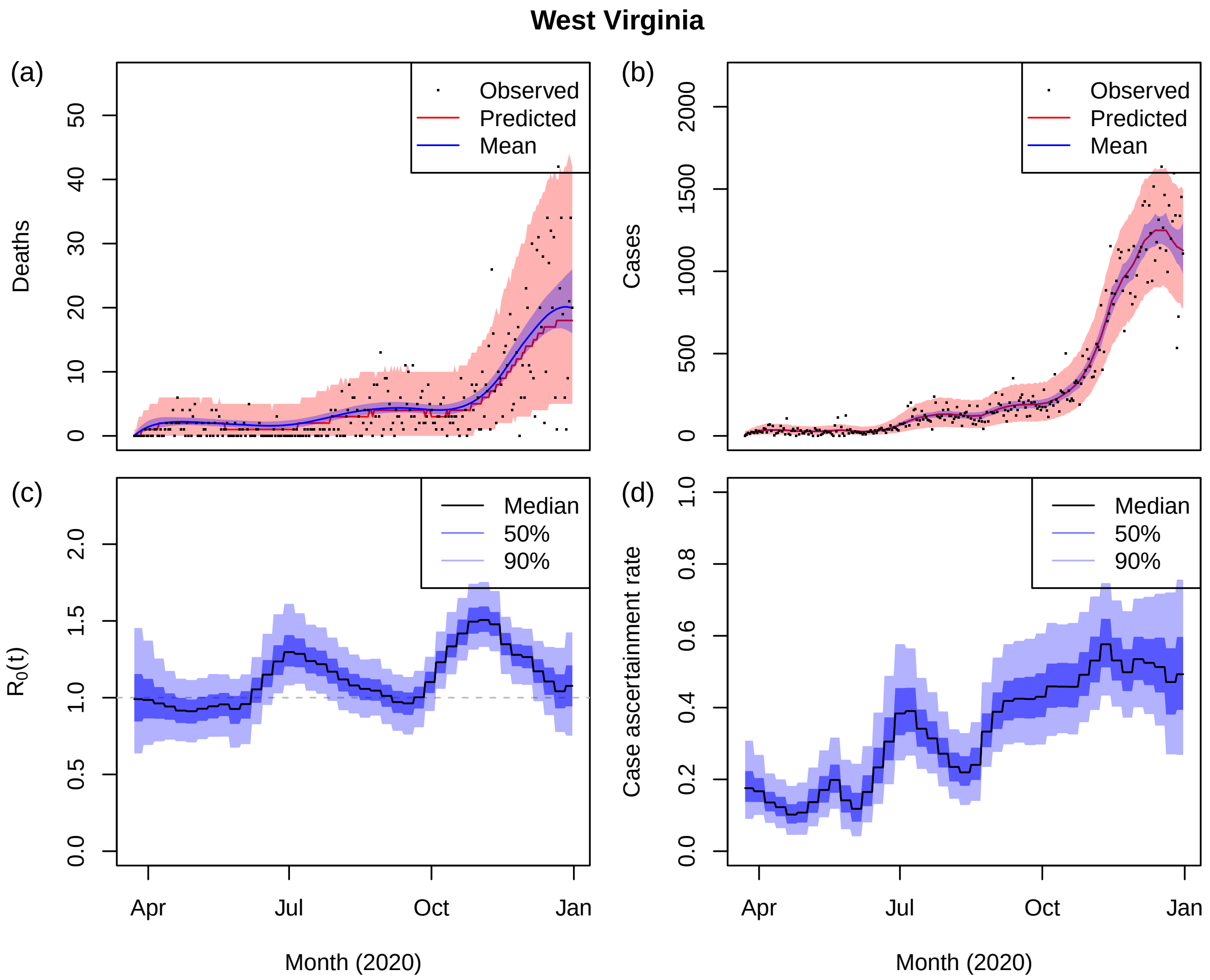

Figure S111: SEIRD model fit to COVID-19 data. **Top panels:** observed (a) deaths $d(t)$ and (b) cases $c(t)$ are plotted in black. Median and 90% credible intervals of the posterior predictive distributions of $d(t)$ and $c(t)$ are in red. Posterior median and 90% credible intervals of the underlying mean parameters $m_D(t)$ and $m_C(t)$ are in blue. **Bottom panels:** posterior median, 50%, and 90% credible intervals for (c) the basic reproduction number $R_0(t)$ and (d) the case ascertainment rate CAR(t).

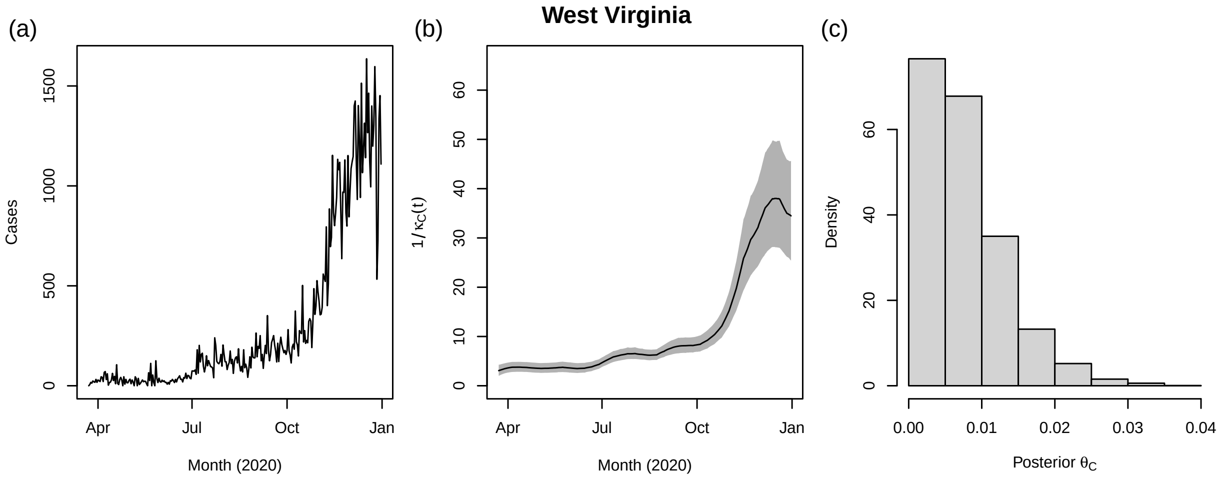

Figure S112: Overdispersion and zero-inflation in state-level clinical case data. (a) Reported cases. (b) Posterior median and 90% credible interval for the time-varying negative binomial overdispersion parameter $\kappa_C(t)^{-1}$. (c) Posterior histogram for the zero-inflation parameter θ_C.

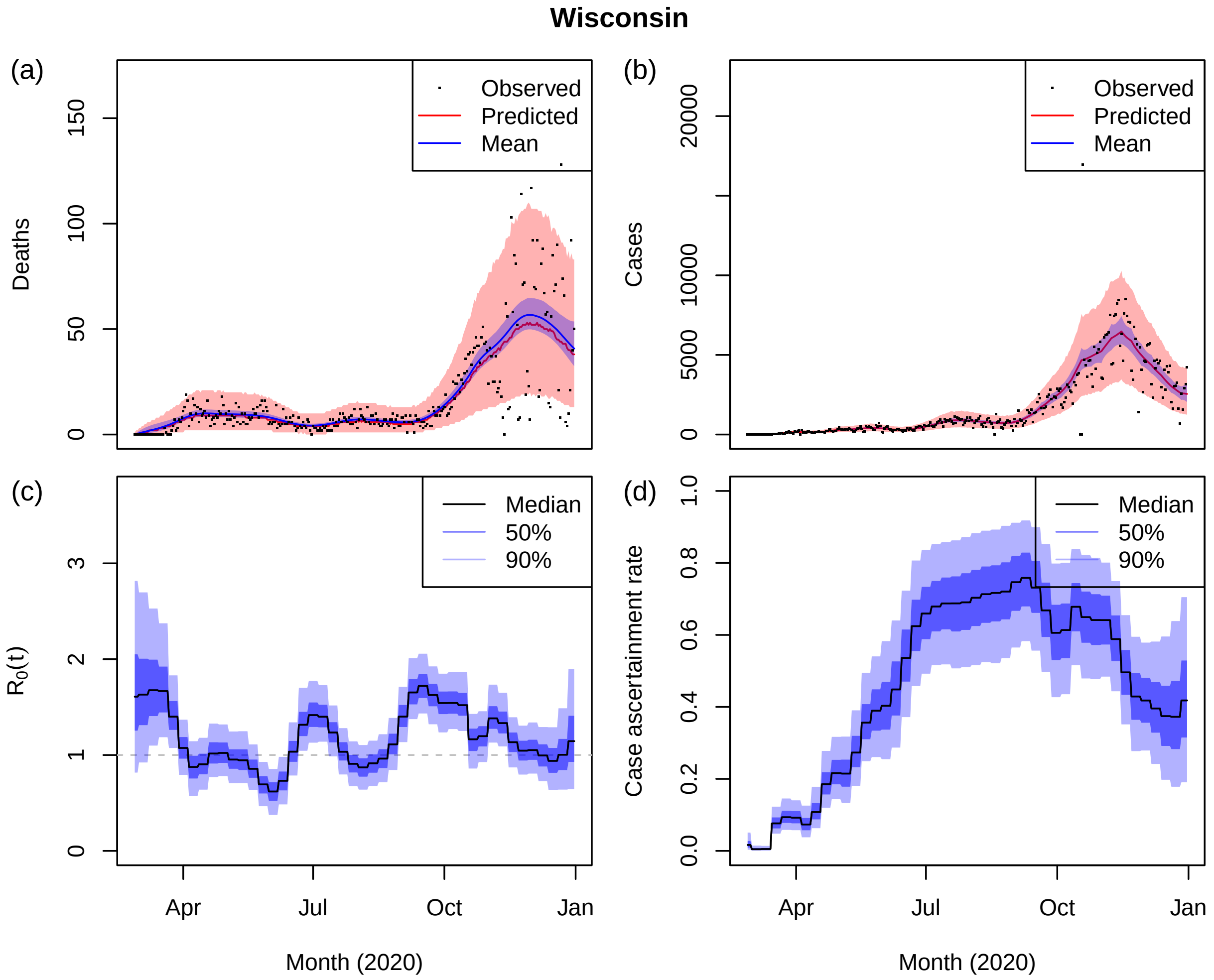

Figure S113: SEIRD model fit to COVID-19 data. **Top panels:** observed (a) deaths $d(t)$ and (b) cases $c(t)$ are plotted in black. Median and 90% credible intervals of the posterior predictive distributions of $d(t)$ and $c(t)$ are in red. Posterior median and 90% credible intervals of the underlying mean parameters $m_D(t)$ and $m_C(t)$ are in blue. **Bottom panels:** posterior median, 50%, and 90% credible intervals for (c) the basic reproduction number $R_0(t)$ and (d) the case ascertainment rate $\text{CAR}(t)$.

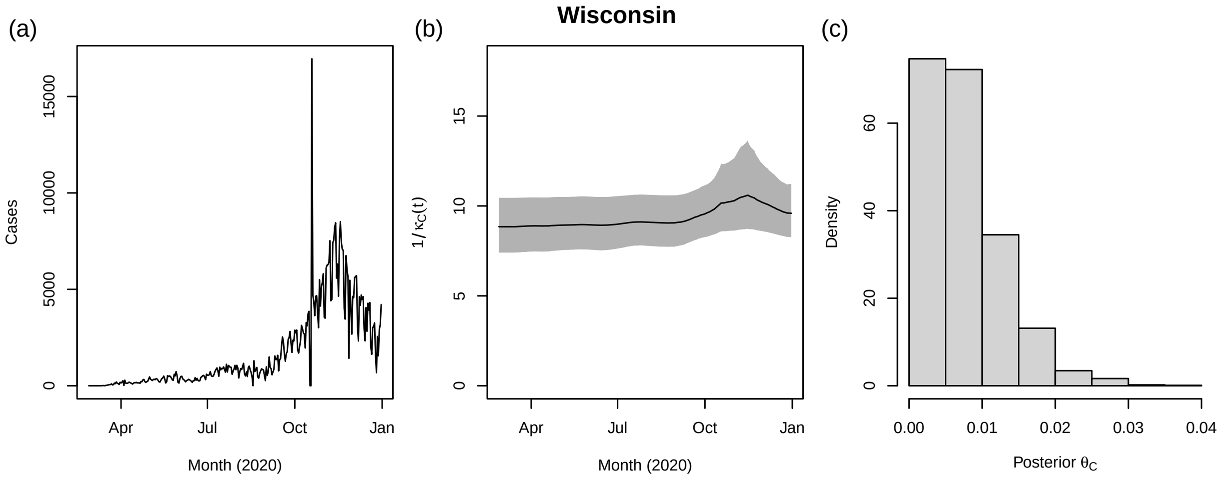

Figure S114: Overdispersion and zero-inflation in state-level clinical case data. (a) Reported cases. (b) Posterior median and 90% credible interval for the time-varying negative binomial overdispersion parameter $\kappa_C(t)^{-1}$. (c) Posterior histogram for the zero-inflation parameter θ_C.

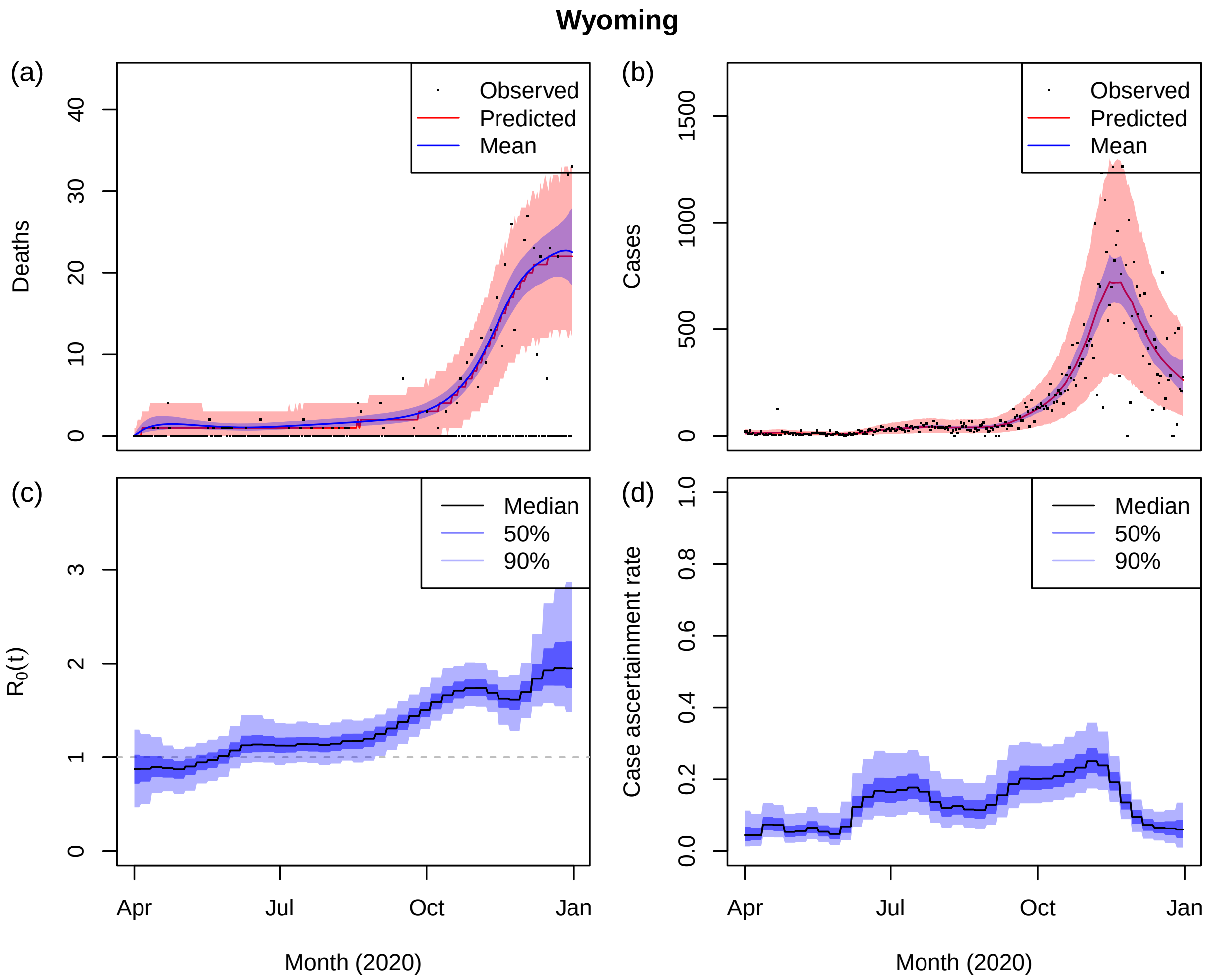

Figure S115: SEIRD model fit to COVID-19 data. **Top panels:** observed (a) deaths $d(t)$ and (b) cases $c(t)$ are plotted in black. Median and 90% credible intervals of the posterior predictive distributions of $d(t)$ and $c(t)$ are in red. Posterior median and 90% credible intervals of the underlying mean parameters $m_D(t)$ and $m_C(t)$ are in blue. **Bottom panels:** posterior median, 50%, and 90% credible intervals for (c) the basic reproduction number $R_0(t)$ and (d) the case ascertainment rate CAR(t).

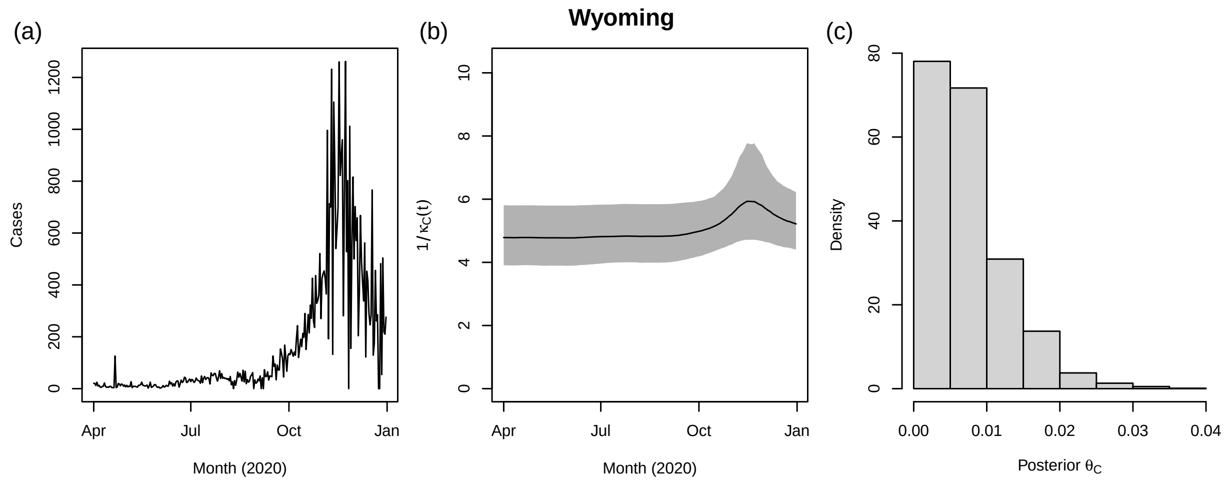

Figure S116: Overdispersion and zero-inflation in state-level clinical case data. (a) Reported cases. (b) Posterior median and 90% credible interval for the time-varying negative binomial overdispersion parameter $\kappa_C(t)^{-1}$. (c) Posterior histogram for the zero-inflation parameter θ_C.

References

1. Cutler, D. M. & Summers, L. H. The COVID-19 pandemic and the $16 trillion virus. *Jama* **324,** 1495–1496 (2020).

2. Bruns, R. & Teran, N. *Weighing the Cost of the Pandemic: Knowing what we know now, how much damage did COVID-19 cause in the United States?* `https://ifp.org/weighing-the-cost-of-the-pandemic/`. Accessed 2024/05/19.

3. Flaxman, S. *et al.* Estimating the effects of non-pharmaceutical interventions on COVID-19 in Europe. *Nature* **584,** 257–261 (2020).

4. Ferguson, N. M. *et al. Impact of non-pharmaceutical interventions (NPIs) to reduce COVID-19 mortality and healthcare demand. Imperial College COVID-19 Response Team* tech. rep. doi: https://doi.org/10.25561/77482 (Imperial College COVID-19 Response Team, Mar. 2020).

5. Greenstone, M. & Nigam, V. Does social distancing matter? *University of Chicago, Becker Friedman Institute for economics working paper* (Mar. 2020).

6. Eichenbaum, M. S., Rebelo, S. & Trabandt, M. The Macroeconomics of Epidemics. *The Review of Financial Studies* **34,** 5149–5187 (2021).

7. Thunström, L., Newbold, S. C., Finnoff, D., Ashworth, M. & Shogren, J. F. The Benefits and Costs of Using Social Distancing to Flatten the Curve for COVID-19. *Journal of Benefit-Cost Analysis* **11,** 179–195 (2020).

8. Farboodi, M., Jarosch, G. & Shimer, R. Internal and external effects of social distancing in a pandemic. *Journal of Economic Theory* **196,** 105293. ISSN: 0022-0531. `https://www.sciencedirect.com/science/article/pii/S0022053121001101` (2021).

9. Gollier, C. Cost–benefit analysis of age-specific deconfinement strategies. *Journal of Public Economic Theory* **22,** 1746–1771 (2020).

10. Jones, C., Philippon, T. & Venkateswaran, V. Optimal Mitigation Policies in a Pandemic: Social Distancing and Working from Home. *The Review of Financial Studies* **34,** 5188–5223. ISSN: 0893-9454. eprint: https://academic.oup.com/rfs/article-pdf/34/11/5188/40724209/hhab076.pdf. https://doi.org/10.1093/rfs/hhab076 (Sept. 2021).

11. Keogh-Brown, M. R., Jensen, H. T., Edmunds, W. J. & Smith, R. D. The impact of Covid-19, associated behaviours and policies on the UK economy: A computable general equilibrium model. *SSM-population health* **12,** 100651 (2020).

12. Barrot, J.-N., Bonelli, M., Grassi, B. & Sauvagnat, J. Causal effects of closing businesses in a pandemic. *Journal of Financial Economics* **154,** 103794. ISSN: 0304-405X. https://www.sciencedirect.com/science/article/pii/S0304405X24000175 (2024).

13. Paulden, M. Why it's time to abandon the ICER. *Pharmacoeconomics* **38,** 781–784 (2020).

14. Weinstein, M. C. Principles of cost-effective resource allocation in health care organizations. *International journal of technology assessment in health care* **6,** 93–103 (1990).

15. Podolsky, M. I., Present, I., Neumann, P. J. & Kim, D. D. A Systematic Review of Economic Evaluations of COVID-19 Interventions: Considerations of Non-Health Impacts and Distributional Issues. *Value in Health* **25,** 1298–1306. ISSN: 1098-3015. https://www.sciencedirect.com/science/article/pii/S1098301522001024 (2022).

16. Craig, B. A. & Black, M. A. Incremental cost-effectiveness ratio and incremental net-health benefit: two sides of the same coin. *Expert Review of Pharmacoeconomics & Outcomes Research* **1,** 37–46 (2001).

17. Stinnett, A. A. & Mullahy, J. Net health benefits: a new framework for the analysis of uncertainty in cost-effectiveness analysis. *Medical decision making* **18,** S68–S80 (1998).

18. Prager, F., Wei, D. & Rose, A. Total economic consequences of an influenza outbreak in the United States. *Risk Analysis* **37,** 4–19 (2017).

19. Kermack, W. O. & McKendrick, A. G. A contribution to the mathematical theory of epidemics. *Proceedings of the royal society of london. Series A, Containing papers of a mathematical and physical character* **115,** 700–721 (1927).

20. Recht, B. *All models are wrong, but some are dangerous.* Accessed 2024/06/17. `https://www.argmin.net/p/all-models-are-wrong-but-some-are`.

21. Juneau, C.-E. *et al.* Lessons from past pandemics: a systematic review of evidence-based, cost-effective interventions to suppress COVID-19. *Systematic reviews* **11,** 90 (2022).

22. Robinson, L. A., Sullivan, R. & Shogren, J. F. Do the Benefits of COVID-19 Policies Exceed the Costs? Exploring Uncertainties in the Age–VSL Relationship. *Risk Analysis* **41,** 761–770. eprint: `https://onlinelibrary.wiley.com/doi/pdf/10.1111/risa.13561`. `https://onlinelibrary.wiley.com/doi/abs/10.1111/risa.13561` (2021).

23. Pasquini-Descomps, H., Brender, N. & Maradan, D. Value for money in H1N1 influenza: a systematic review of the cost-effectiveness of pandemic interventions. *Value in Health* **20,** 819–827 (2017).

24. Xue, Y., Kristiansen, I. S. & de Blasio, B. F. Dynamic modelling of costs and health consequences of school closure during an influenza pandemic. *BMC public health* **12,** 1–17 (2012).

25. Adda, J. Economic Activity and the Spread of Viral Diseases: Evidence from High Frequency Data. *The Quarterly Journal of Economics* **131,** 891–941. ISSN: 0033-

5533. eprint: https://academic.oup.com/qje/article-pdf/131/2/891/30636376/qjw005.pdf. https://doi.org/10.1093/qje/qjw005 (Feb. 2016).

26. Dauelsberg, L. R. *et al.* Cost effectiveness of preemptive school closures to mitigate pandemic influenza outbreaks of differing severity in the United States. *BMC Public Health* **24,** 200 (2024).

27. Perlroth, D. J. *et al.* Health outcomes and costs of community mitigation strategies for an influenza pandemic in the United States. *Clinical infectious diseases* **50,** 165–174 (2010).

28. Milne, G. J., Halder, N. & Kelso, J. K. The cost effectiveness of pandemic influenza interventions: a pandemic severity based analysis. *PloS one* **8,** e61504 (2013).

29. Kelso, J. K., Halder, N., Postma, M. J. & Milne, G. J. Economic analysis of pandemic influenza mitigation strategies for five pandemic severity categories. *BMC public health* **13,** 1–17 (2013).

30. Allcott, H. *et al.* Polarization and public health: Partisan differences in social distancing during the coronavirus pandemic. *Journal of Public Economics* **191,** 104254. ISSN: 0047-2727. https://www.sciencedirect.com/science/article/pii/S0047272720301183 (2020).

31. Barrios, J. M. & Hochberg, Y. V. Risk perceptions and politics: Evidence from the COVID-19 pandemic. *Journal of Financial Economics* **142,** 862–879. ISSN: 0304-405X. https://www.sciencedirect.com/science/article/pii/S0304405X21002324 (2021).

32. Painter, M. & Qiu, T. Political beliefs affect compliance with government mandates. *Journal of Economic Behavior and Organization* **185,** 688–701. ISSN: 0167-2681. https://www.sciencedirect.com/science/article/pii/S016726812100113X (2021).

33. Brodeur, A., Gray, D., Islam, A. & Bhuiyan, S. A literature review of the economics of COVID-19. *Journal of Economic Surveys* **35,** 1007–1044. eprint: https://onlinelibrary.wiley.com/doi/pdf/10.1111/joes.12423. https://onlinelibrary.wiley.com/doi/abs/10.1111/joes.12423 (2021).

34. Adolph, C. *et al.* Governor partisanship explains the adoption of statewide mask mandates in response to COVID-19. *State Politics & Policy Quarterly* **22,** 24–49 (2022).

35. Adolph, C. *et al.* The pandemic policy U-turn: Partisanship, public health, and race in decisions to ease COVID-19 social distancing policies in the United States. *Perspectives on Politics* **20,** 595–617 (2022).

36. Adolph, C., Amano, K., Bang-Jensen, B., Fullman, N. & Wilkerson, J. Pandemic politics: Timing state-level social distancing responses to COVID-19. *Journal of Health Politics, Policy and Law* **46,** 211–233 (2021).

37. Hale, T. *et al.* A global panel database of pandemic policies (Oxford COVID-19 Government Response Tracker). *Nature Human Behaviour* (2021).

38. Sharma, M. *et al.* Understanding the effectiveness of government interventions against the resurgence of COVID-19 in Europe. *Nature communications* **12,** 5820 (2021).

39. Bo, Y. *et al.* Effectiveness of non-pharmaceutical interventions on COVID-19 transmission in 190 countries from 23 January to 13 April 2020. *International Journal of Infectious Diseases* **102,** 247–253 (2021).

40. Karaivanov, A., Lu, S. E., Shigeoka, H., Chen, C. & Pamplona, S. Face masks, public policies and slowing the spread of COVID-19: Evidence from Canada. *Journal of Health Economics* **78,** 102475. ISSN: 0167-6296. https://www.sciencedirect.com/science/article/pii/S0167629621000606 (2021).

41. Yang, W., Shaff, J. & Shaman, J. Effectiveness of non-pharmaceutical interventions to contain COVID-19: a case study of the 2020 spring pandemic wave in New York City. *Journal of the Royal Society Interface* **18,** 20200822 (2021).

42. Chernozhukov, V., Kasahara, H. & Schrimpf, P. Causal impact of masks, policies, behavior on early covid-19 pandemic in the U.S. *Journal of Econometrics* **220,** 23–62 (2021).

43. Jamison, J. C., Bundy, D., Jamison, D. T., Spitz, J. & Verguet, S. Comparing the impact on COVID-19 mortality of self-imposed behavior change and of government regulations across 13 countries. *Health services research* **56,** 874–884 (2021).

44. Talic, S. *et al.* Effectiveness of public health measures in reducing the incidence of covid-19, SARS-CoV-2 transmission, and covid-19 mortality: systematic review and meta-analysis. *BMJ* **375** (2021).

45. Li, H. *et al.* Efficacy and practice of facemask use in general population: a systematic review and meta-analysis. *Translational psychiatry* **12,** 49 (2022).

46. Lyu, W. & Wehby, G. L. Community Use Of Face Masks And COVID-19: Evidence From A Natural Experiment Of State Mandates In The US: Study examines impact on COVID-19 growth rates associated with state government mandates requiring face mask use in public. *Health affairs* **39,** 1419–1425 (2020).

47. Rader, B. *et al.* Mask-wearing and control of SARS-CoV-2 transmission in the USA: a cross-sectional study. *The Lancet Digital Health* **3,** e148–e157 (2021).

48. Greenhalgh, T. *et al.* Masks and respirators for prevention of respiratory infections: a state of the science review. *Clinical microbiology reviews,* e00124–23 (2024).

49. Brauner, J. M. *et al.* Inferring the effectiveness of government interventions against COVID-19. *Science* **371,** eabd9338 (2021).

50. Banholzer, N. *et al.* Estimating the effects of non-pharmaceutical interventions on the number of new infections with COVID-19 during the first epidemic wave. *PLoS one* **16,** e0252827 (2021).

51. Cauchemez, S., Valleron, A.-J., Boelle, P.-Y., Flahault, A. & Ferguson, N. M. Estimating the impact of school closure on influenza transmission from Sentinel data. *Nature* **452,** 750–754 (2008).

52. Markel, H. *et al.* Nonpharmaceutical Interventions Implemented by US Cities During the 1918-1919 Influenza Pandemic. *JAMA* **298,** 644–654. ISSN: 0098-7484. eprint: `https://jamanetwork.com/journals/jama/articlepdf/208354/joc70085\_644\_654.pdf`. `https://doi.org/10.1001/jama.298.6.644` (Aug. 2007).

53. Auger, K. A. *et al.* Association between statewide school closure and COVID-19 incidence and mortality in the US. *Jama* **324,** 859–870 (2020).

54. Liu, Y., Morgenstern, C., Kelly, J., Lowe, R. & Jit, M. The impact of non-pharmaceutical interventions on SARS-CoV-2 transmission across 130 countries and territories. *BMC medicine* **19,** 1–12 (2021).

55. Stokes, J., Turner, A. J., Anselmi, L., Morciano, M. & Hone, T. The relative effects of non-pharmaceutical interventions on wave one Covid-19 mortality: natural experiment in 130 countries. *BMC Public Health* **22,** 1113 (2022).

56. Li, Y., Campbell, H. & Kulkarni, D. e. a. The temporal association of introducing and lifting non-pharmaceutical interventions with the time-varying reproduction number (R) of SARS-CoV-2: a modelling study across 131 countries. *The Lancet Infectious Diseases* **21,** 193–202 (2021).

57. Haug, N. *et al.* Ranking the effectiveness of worldwide COVID-19 government interventions. *Nature human behaviour* **4,** 1303–1312 (2020).

58. Ferguson, N. M. *et al.* Strategies for mitigating an influenza pandemic. *Nature* **442,** 448–452 (2006).

59. Bodenstein, M., Corsetti, G. & Guerrieri, L. Social distancing and supply disruptions in a pandemic. *Quantitative Economics* **13,** 681–721 (2022).

60. Rainisch, G. *et al.* Estimated COVID-19 Cases and Hospitalizations Averted by Case Investigation and Contact Tracing in the US. *JAMA Network Open* **5,** e224042–e224042. ISSN: 2574-3805. eprint: `https://jamanetwork.com/journals/jamanetworkopen/articlepdf/2790518/rainisch\_2022\_oi\_220145\_1652374295.93757.pdf`. `https://doi.org/10.1001/jamanetworkopen.2022.4042` (Mar. 2022).

61. Wang, X. *et al.* The effectiveness of COVID-19 testing and contact tracing in a US city. *Proceedings of the National Academy of Sciences* **119,** e2200652119 (2022).

62. Hellewell, J. *et al.* Feasibility of controlling COVID-19 outbreaks by isolation of cases and contacts. *The Lancet Global Health* **8,** e488–e496 (2020).

63. Davis, E. L. *et al.* Contact tracing is an imperfect tool for controlling COVID-19 transmission and relies on population adherence. *Nature communications* **12,** 5412 (2021).

64. Alexander, D. & Karger, E. Do Stay-at-Home Orders Cause People to Stay at Home? Effects of Stay-at-Home Orders on Consumer Behavior. *The Review of Economics and Statistics* **105,** 1017–1027. ISSN: 0034-6535. eprint: `https://direct.mit.edu/rest/article-pdf/105/4/1017/2142564/rest\_a\_01108.pdf`. `https://doi.org/10.1162/rest%5C_a%5C_01108` (July 2023).

65. Xin, H. *et al.* Estimating the latent period of coronavirus disease 2019 (COVID-19). *Clinical Infectious Diseases* **74,** 1678–1681 (2022).

66. Linton, N. M. *et al.* Incubation period and other epidemiological characteristics of 2019 novel coronavirus infections with right truncation: a statistical analysis of publicly available case data. *Journal of clinical medicine* **9,** 538 (2020).

67. Lauer, S. A. *et al.* The incubation period of coronavirus disease 2019 (COVID-19) from publicly reported confirmed cases: estimation and application. *Annals of internal medicine* **172,** 577–582 (2020).

68. Gallo, L. G. *et al.* Ten epidemiological parameters of COVID-19: use of rapid literature review to inform predictive models during the pandemic. *Frontiers in public health* **8,** 598547 (2020).

69. Wu, Y. *et al.* Incubation period of COVID-19 caused by unique SARS-CoV-2 strains: a systematic review and meta-analysis. *JAMA network open* **5,** e2228008–e2228008 (2022).

70. Hakki, S. *et al.* Onset and window of SARS-CoV-2 infectiousness and temporal correlation with symptom onset: a prospective, longitudinal, community cohort study. *The Lancet Respiratory Medicine* **10,** 1061–1073 (2022).

71. Byrne, A. W. *et al.* Inferred duration of infectious period of SARS-CoV-2: rapid scoping review and analysis of available evidence for asymptomatic and symptomatic COVID-19 cases. *BMJ open* **10,** e039856 (2020).

72. Ward, T. & Johnsen, A. Understanding an evolving pandemic: An analysis of the clinical time delay distributions of COVID-19 in the United Kingdom. *Plos one* **16,** e0257978 (2021).

73. Irons, N. J. & Raftery, A. E. Estimating SARS-CoV-2 infections from deaths, confirmed cases, tests, and random surveys. *Proceedings of the National Academy of Sciences* **118,** e2103272118 (2021).

74. Liu, Y., Gayle, A. A., Wilder-Smith, A. & Rocklöv, J. The reproductive number of COVID-19 is higher compared to SARS coronavirus. *Journal of Travel Medicine* **27,** taaa021. ISSN: 1708-8305. eprint: https://academic.oup.com/jtm/article-pdf/27/2/taaa021/32902430/taaa021.pdf. https://doi.org/10.1093/jtm/taaa021 (Feb. 2020).

75. Jin, R. The lag between daily reported Covid-19 cases and deaths and its relationship to age. *Journal of public health research* **10,** jphr–2021 (2021).

76. U.S. Bureau of Economic Analysis. *Gross Domestic Product by State, Annual 2017-2022.* Accessed 2024/05/28. https://apps.bea.gov/regional/histdata/releases/0923gdpstate/SAGDP.zip.

77. U.S. Bureau of Economic Analysis. *Personal Consumption Expenditures by State, 2022.* Accessed 2024/05/28. https://apps.bea.gov/regional/zip/SAPCE.zip.

78. U.S. Census Bureau. *Median Personal Income in the United States [MEPAINUSA646N]* Retrieved from FRED, Federal Reserve Bank of St. Louis; 2024/05/28. https://fred.stlouisfed.org/series/MEPAINUSA646N.

79. U.S. Bureau of Economic Analysis. *State Personal Income: 2nd Quarter 2022 and Annual 2021.* Accessed 2024/05/28. https://www.bea.gov/sites/default/files/2022-09/covid-workbook-ann.xlsx.

80. U.S. Bureau of Economic Analysis. *Gross Domestic Product, Fourth Quarter and Year 2019 (Third Estimate); Corporate Profits, Fourth Quarter and Year 2019.* Accessed 2024/05/28. https://www.bea.gov/news/2020/gross-domestic-product-fourth-quarter-and-year-2019-third-estimate-corporate-profits.

81. Aum, S., Lee, S. Y. (& Shin, Y. COVID-19 doesn't need lockdowns to destroy jobs: The effect of local outbreaks in Korea. *Labour Economics* **70,** 101993. ISSN: 0927-5371. https://www.sciencedirect.com/science/article/pii/S0927537121000282 (2021).

82. Skarp, J. E. *et al.* A systematic review of the costs relating to non-pharmaceutical interventions against infectious disease outbreaks. *Applied Health Economics and Health Policy* **19,** 673–697 (2021).

83. Bartsch, S. M. *et al.* The Potential Health Care Costs And Resource Use Associated With COVID-19 In The United States: A simulation estimate of the direct medical costs and health care resource use associated with COVID-19 infections in the United States. *Health affairs* **39,** 927–935 (2020).

84. DeMartino, J. K. *et al.* Direct health care costs associated with COVID-19 in the United States. *Journal of Managed Care & Specialty Pharmacy* **28.** PMID: 35722829, 936–947. eprint: https://doi.org/10.18553/jmcp.2022.22050. https://doi.org/10.18553/jmcp.2022.22050 (2022).

85. Psacharopoulos, G., Collis, V., Patrinos, H. A. & Vegas, E. The COVID-19 Cost of School Closures in Earnings and Income across the World. *Comparative Education Review* **65,** 271–287. eprint: https://doi.org/10.1086/713540. https://doi.org/10.1086/713540 (2021).

86. Hanushek, E. A. & Woessmann, L. The economic impacts of learning losses. *OECD publishing Paris.* Accessed 2025/07/20. https://www.moe.gov.mm/sites/default/files/COVID-19_The_Economic_Impacts_of_Learning_Losses.pdf (2020).

87. Betthäuser, B. A., Bach-Mortensen, A. M. & Engzell, P. A systematic review and meta-analysis of the evidence on learning during the COVID-19 pandemic. *Nature Human Behaviour* **7,** 375–385 (2023).

88. Fahle, E. M. *et al.* School district and community factors associated with learning loss during the COVID-19 pandemic. *Center for Education Policy Research at Harvard University: Cambridge, MA, USA* (2023).

89. Lempel, H., Epstein, J. M. & Hammond, R. A. Economic cost and health care workforce effects of school closures in the US. *PLoS currents* **1** (2009).

90. Sadique, M. Z., Adams, E. J. & Edmunds, W. J. Estimating the costs of school closure for mitigating an influenza pandemic. *BMC public health* **8,** 1–7 (2008).

91. Viner, R. M. *et al.* School closure and management practices during coronavirus outbreaks including COVID-19: a rapid systematic review. *The Lancet Child & Adolescent Health* **4,** 397–404 (2020).

92. Gupta, S. *et al.* Effects of social distancing policy on labor market outcomes. *Contemporary Economic Policy* **41,** 166–193. eprint: https://onlinelibrary.wiley.

com/doi/pdf/10.1111/coep.12582. https://onlinelibrary.wiley.com/doi/abs/10.1111/coep.12582 (2023).

93. Crucini, M. J. & O'Flaherty, O. *Stay-at-Home Orders in a Fiscal Union.* Working Paper 28182 (National Bureau of Economic Research, Dec. 2020). http://www.nber.org/papers/w28182.

94. Baek, C., McCrory, P. B., Messer, T. & Mui, P. Unemployment Effects of Stay-at-Home Orders: Evidence from High-Frequency Claims Data. *The Review of Economics and Statistics* **103,** 979–993. ISSN: 0034-6535. eprint: https://direct.mit.edu/rest/article-pdf/103/5/979/1975726/rest_a_00996.pdf. https://doi.org/10.1162/rest%5C_a%5C_00996 (Nov. 2021).

95. Coibion, O., Gorodnichenko, Y. & Weber, M. The cost of the COVID-19 crisis: Lockdowns, macroeconomic expectations, and consumer spending. *Journal of Economic Behavior & Organization* **229,** 106846 (2025).

96. Bartsch, S. M. *et al.* Maintaining face mask use before and after achieving different COVID-19 vaccination coverage levels: a modelling study. *The Lancet Public Health* **7,** e356–e365 (2022).

97. Lo, J. *et al. Health Spending: Prices for COVID-19 testing.* Accessed 2024/05/28. https://www.healthsystemtracker.org/brief/prices-for-covid-19-testing/.

98. Sharfstein, J. *Q&A: HOW MUCH DOES IT COST TO GET A COVID-19 TEST? IT DEPENDS.* Accessed 2024/05/28. https://coronavirus.jhu.edu/from-our-experts/q-and-a-how-much-does-it-cost-to-get-a-covid-19-test-it-depends.

99. The Atlantic. *The COVID Tracking Project.* Accessed 2025/07/20. covidtracking.com.

100. Fields, V. L. *et al.* Coronavirus Disease Contact Tracing Outcomes and Cost, Salt Lake County, Utah, USA, March–May 2020. *Emerging Infectious Diseases* **27,** 2999 (2021).

101. Spencer, K. D. COVID-19 case investigation and contact tracing efforts from health departments—United States, June 25–July 24, 2020. *MMWR. Morbidity and Mortality Weekly Report* **70** (2021).

102. Lash, R. R. *et al.* COVID-19 Case Investigation and Contact Tracing in the US, 2020. *JAMA Network Open* **4,** e2115850–e2115850. ISSN: 2574-3805. eprint: `https://jamanetwork.com/journals/jamanetworkopen/articlepdf/2780568/lash\_2021\_oi\_210475\_1622057707.87431.pdf`. `https://doi.org/10.1001/jamanetworkopen.2021.15850` (June 2021).
